# Supplementary figures and images for: EROS is a selective chaperone regulating the phagocyte NADPH oxidase and purinergic signalling
Source: eLife. 2022 Nov 24;11:e76387. doi: 10.7554/eLife.76387 (PMC9767466; doi:10.7554/eLife.76387)

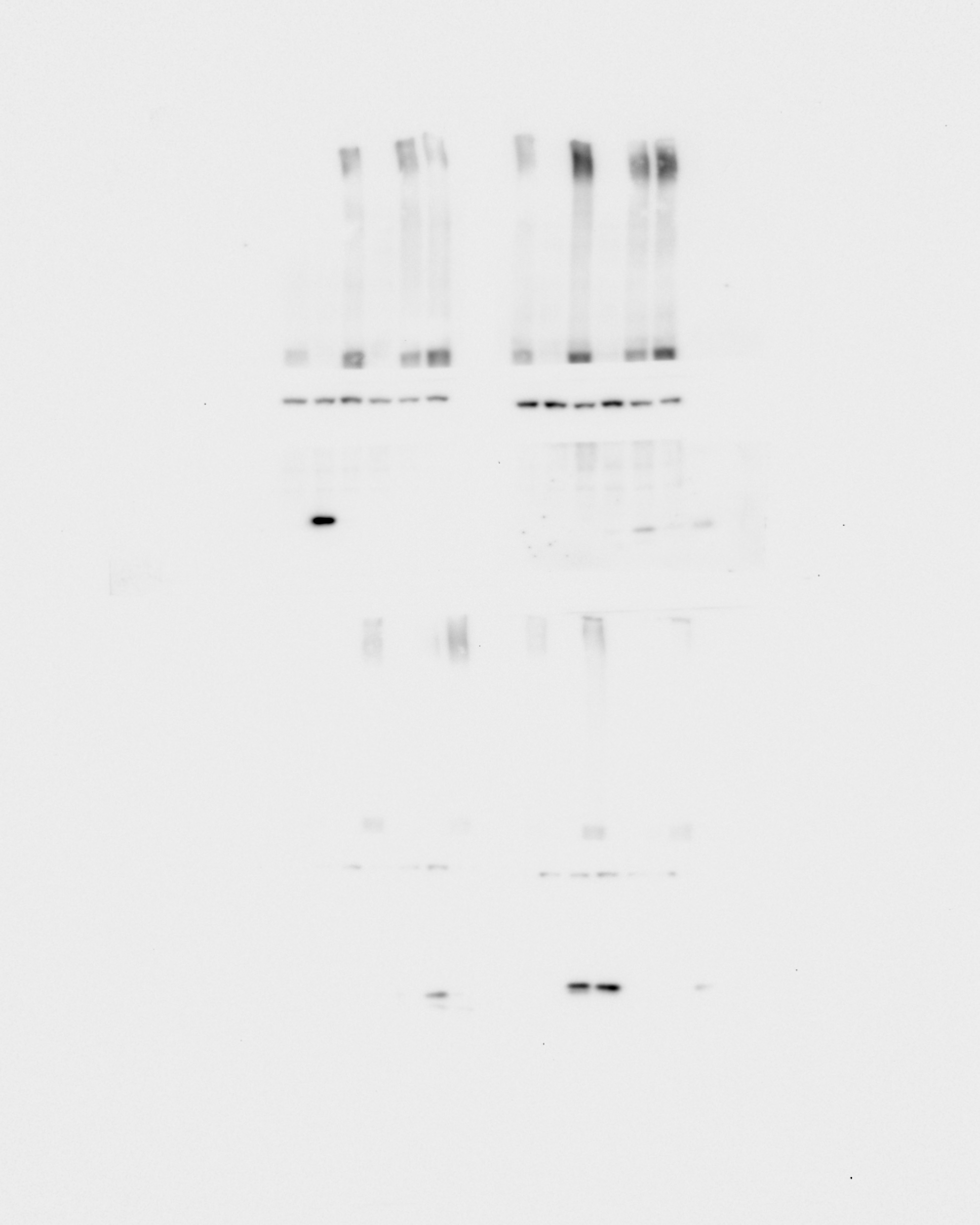

Supplement: Figure 1—source data 1. [file elife-76387-fig1-data1.zip › Figure 1- source data 1/2020-02-05 13h03m29s Chemiluminescence 67.291s HEK293 eros.tif]

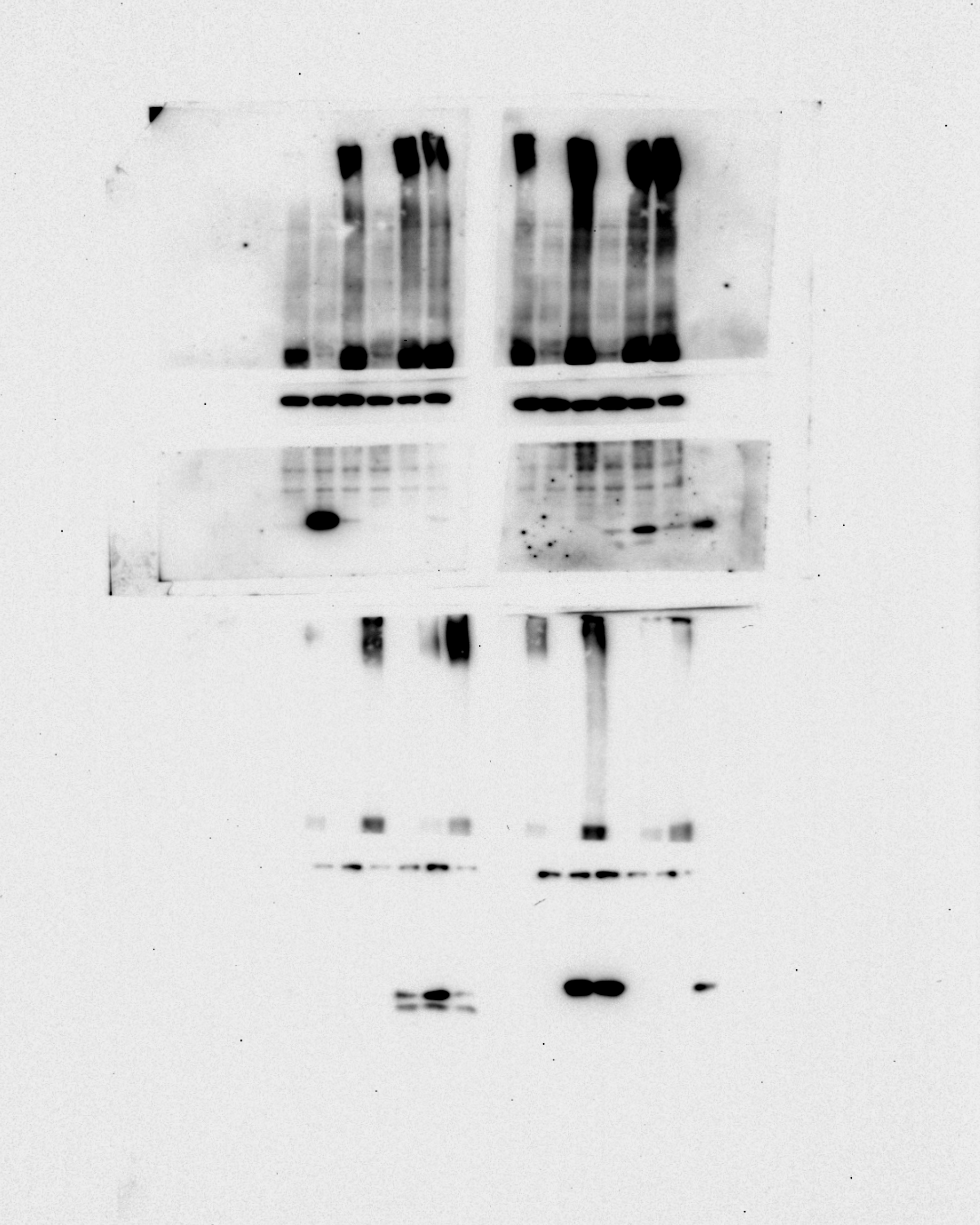

Supplement: Figure 1—source data 1. [file elife-76387-fig1-data1.zip › Figure 1- source data 1/2020-02-05 13h29m08s Chemiluminescence 1499.984s HEK293 actin.tif]

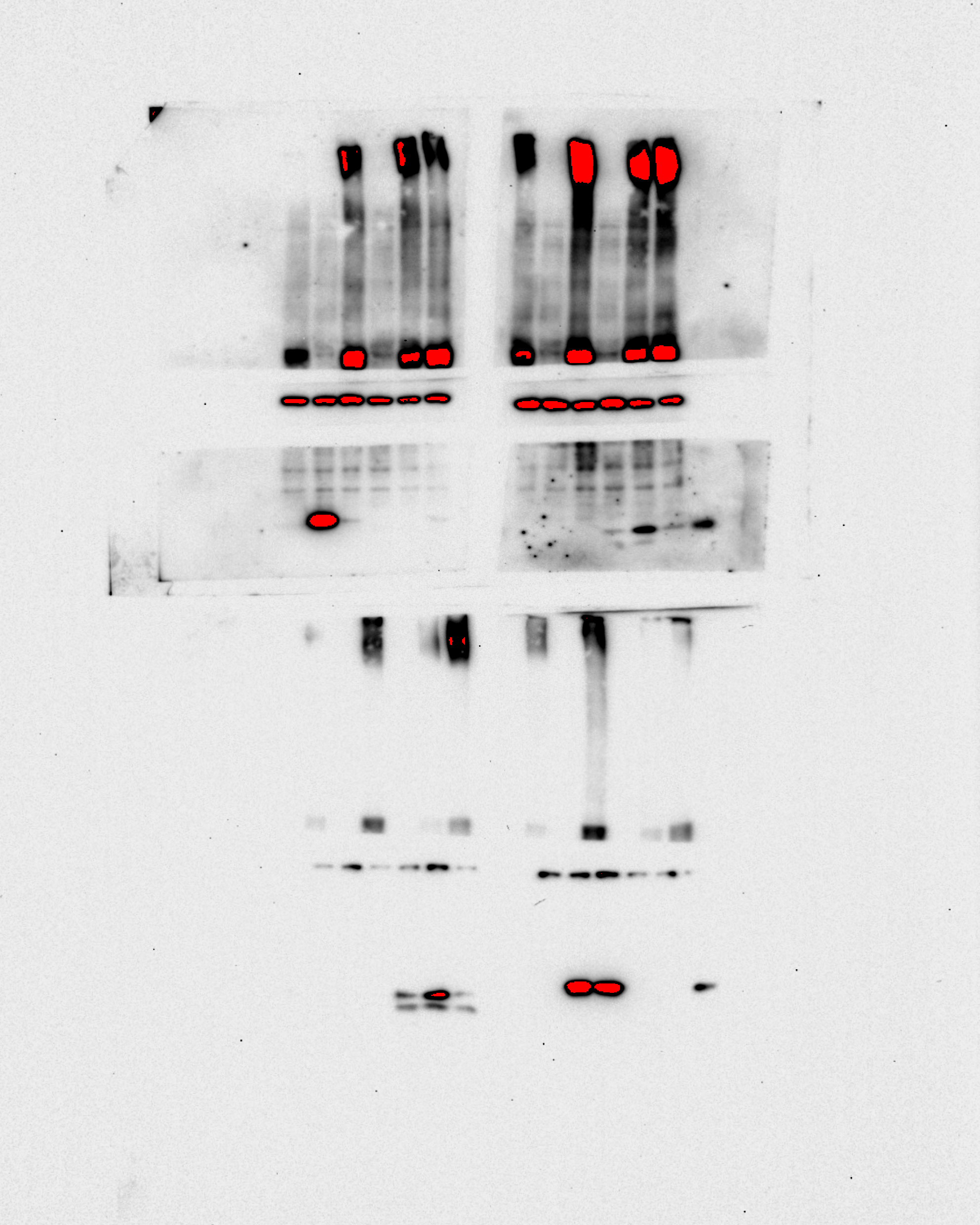

Supplement: Figure 1—source data 1. [file elife-76387-fig1-data1.zip › Figure 1- source data 1/2020-02-05 13h29m08s Chemiluminescence 1499.984s HEK293 gp91phox.tif]

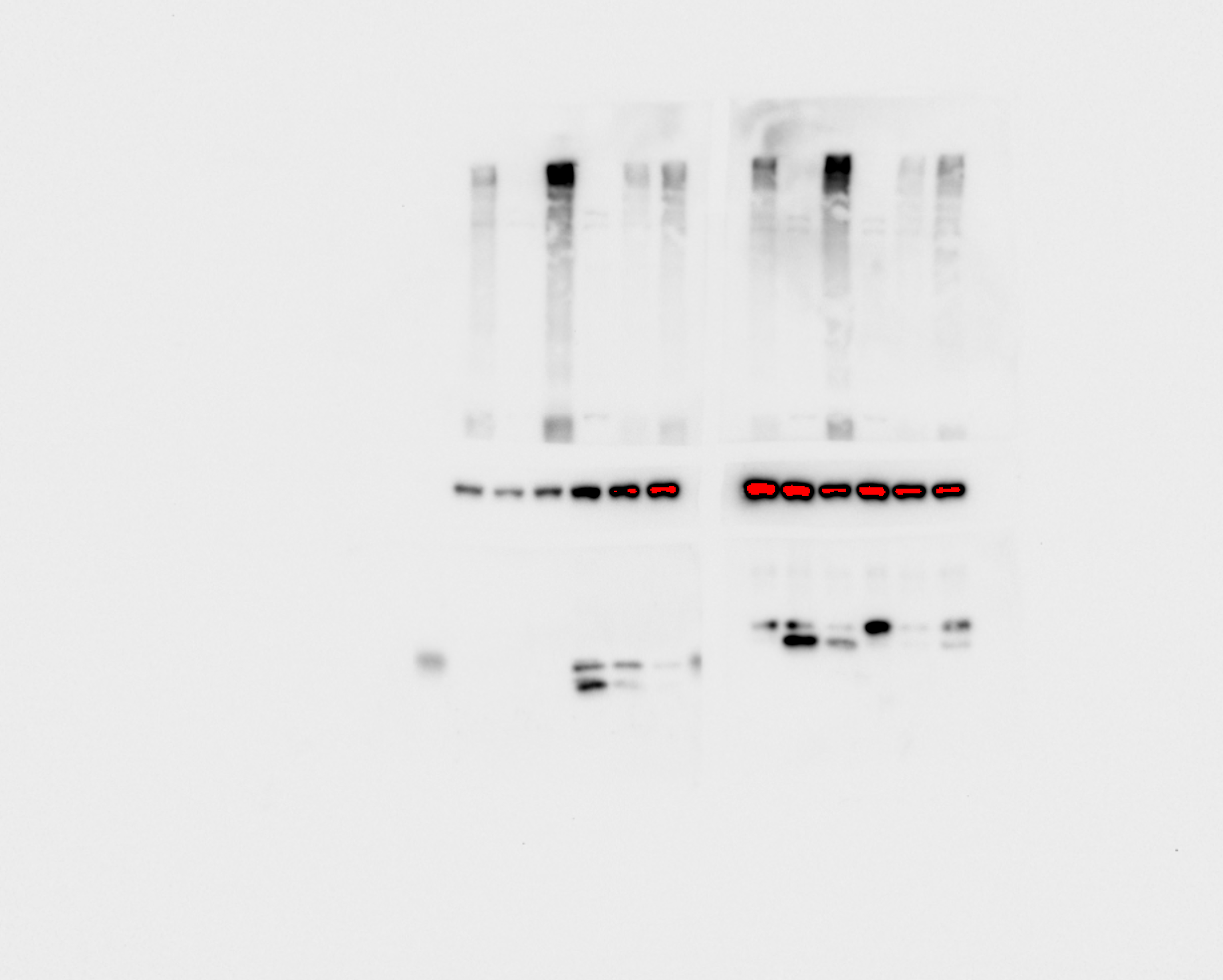

Supplement: Figure 1—source data 1. [file elife-76387-fig1-data1.zip › Figure 1- source data 1/2020-02-28 12h14m51s Chemiluminescence 10.000s 3T3 Eros.tif]

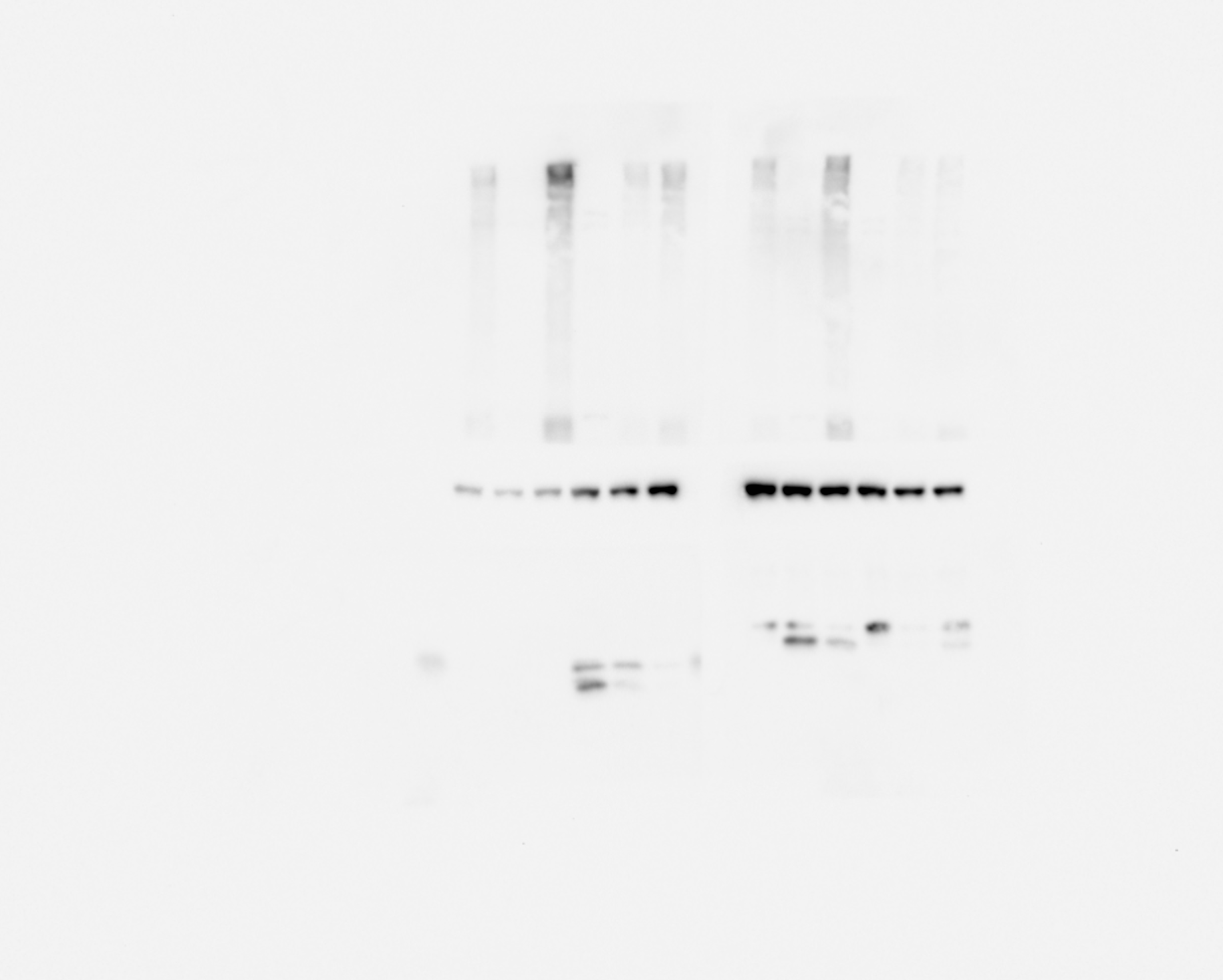

Supplement: Figure 1—source data 1. [file elife-76387-fig1-data1.zip › Figure 1- source data 1/2020-02-28 12h39m09s Chemiluminescence 10.000s 3T3 actin.tif]

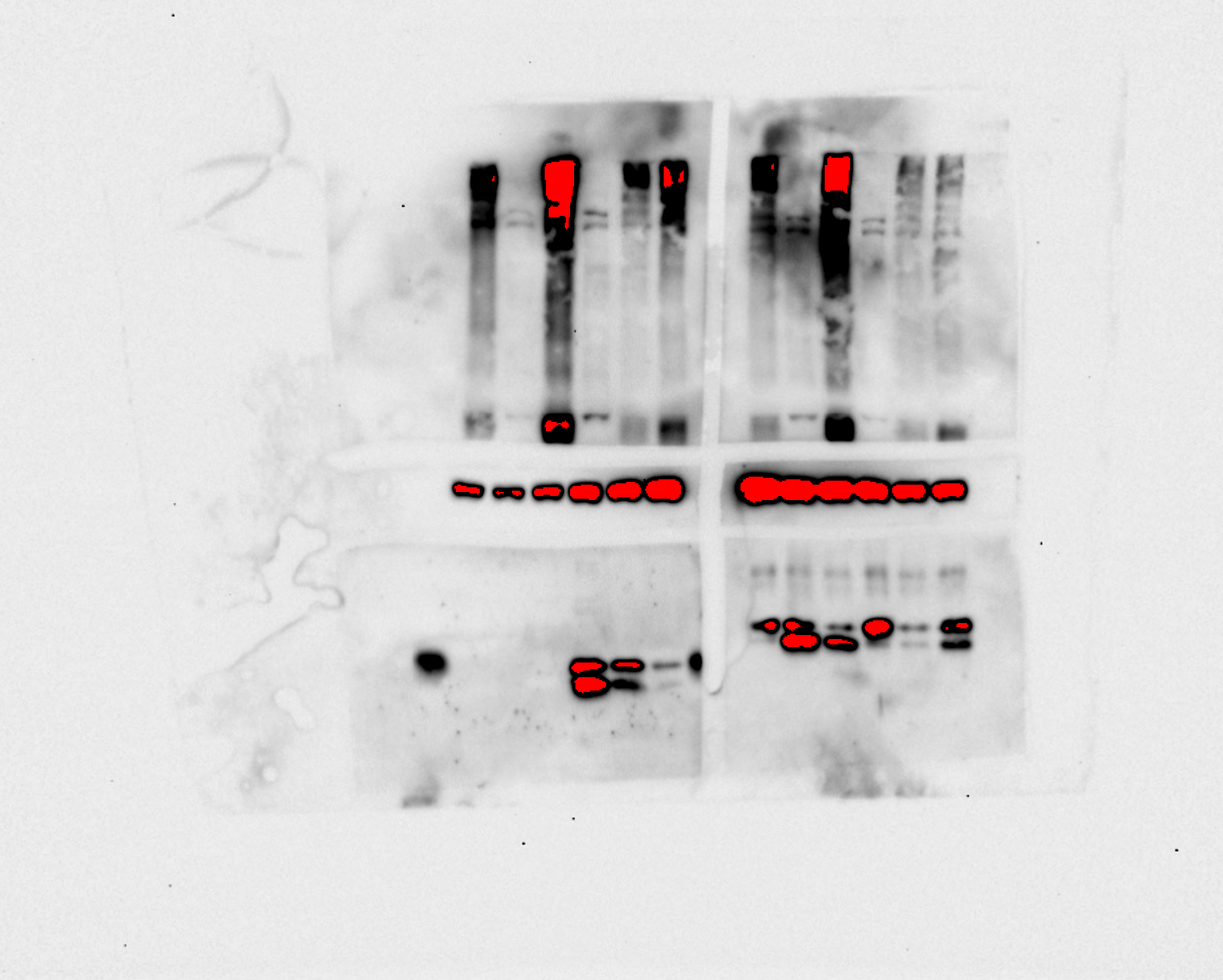

Supplement: Figure 1—source data 1. [file elife-76387-fig1-data1.zip › Figure 1- source data 1/2020-02-28 12h42m40s Chemiluminescence 207.142s 3T3 gp91 phox.tif]

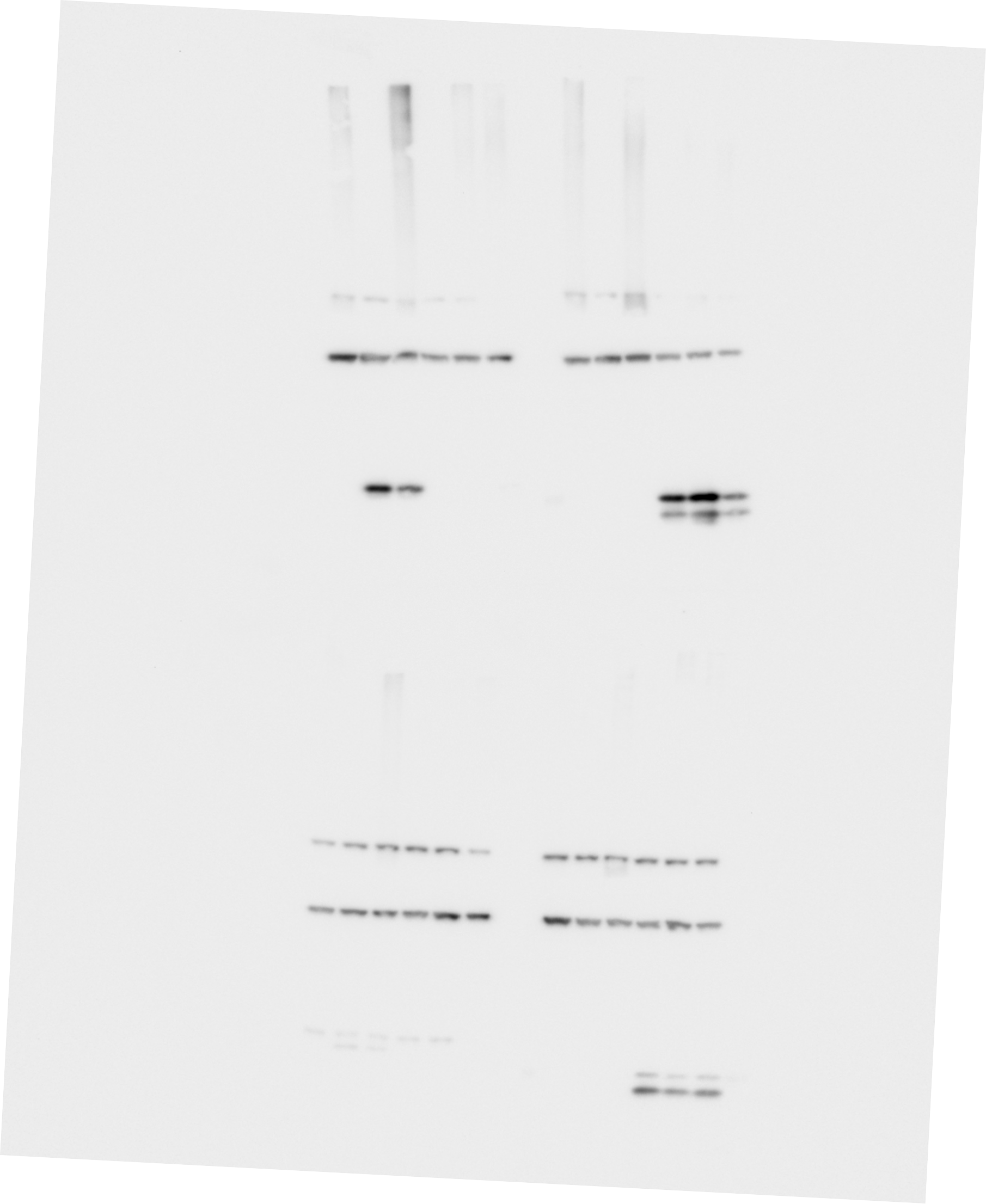

Supplement: Figure 1—source data 1. [file elife-76387-fig1-data1.zip › Figure 1- source data 1/2020-03-06 12h30m41s Chemiluminescence 10.000s COS7 actin.tif]

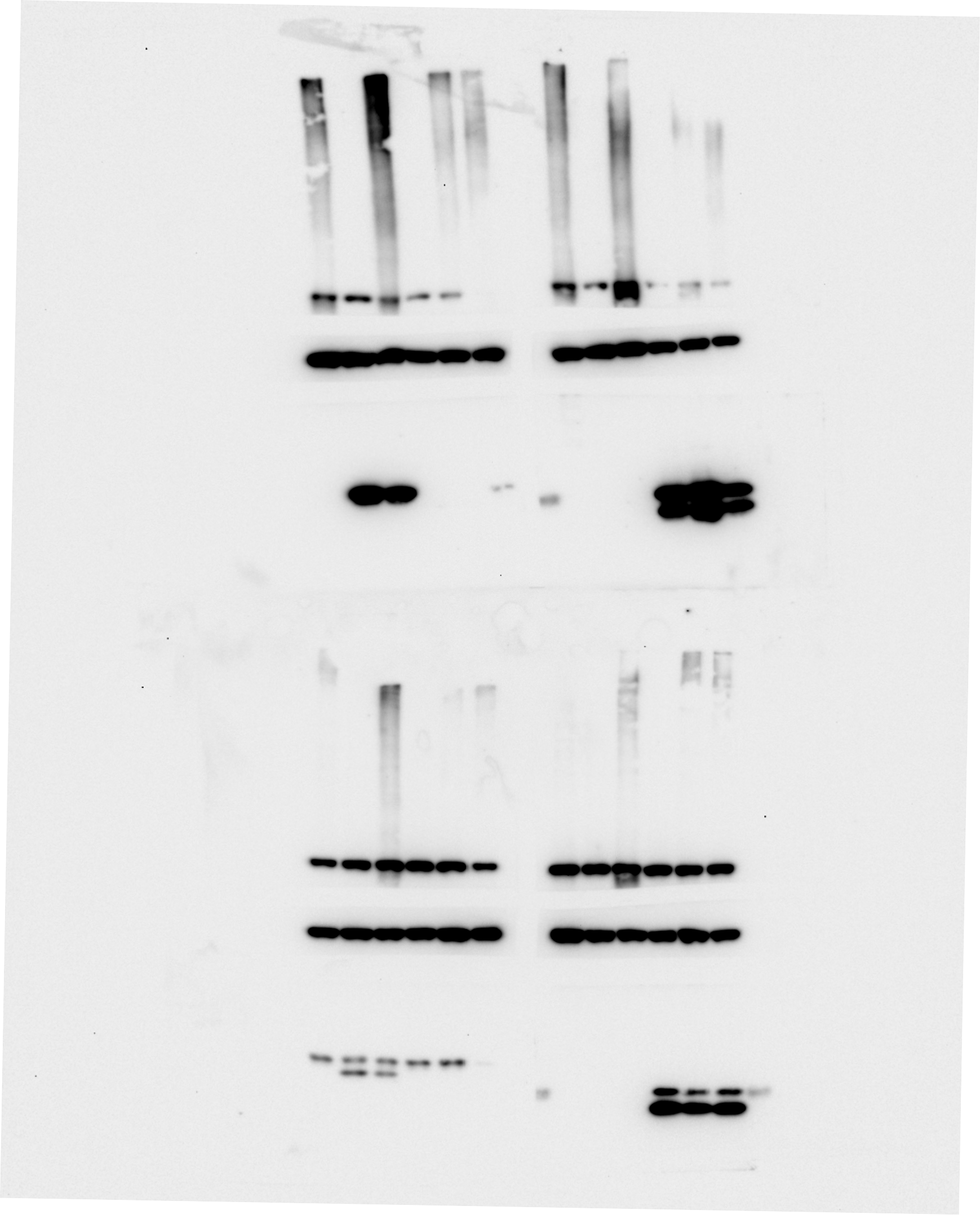

Supplement: Figure 1—source data 1. [file elife-76387-fig1-data1.zip › Figure 1- source data 1/2020-03-06 12h34m06s Chemiluminescence 200.713 COS7 gp91phox.tif]

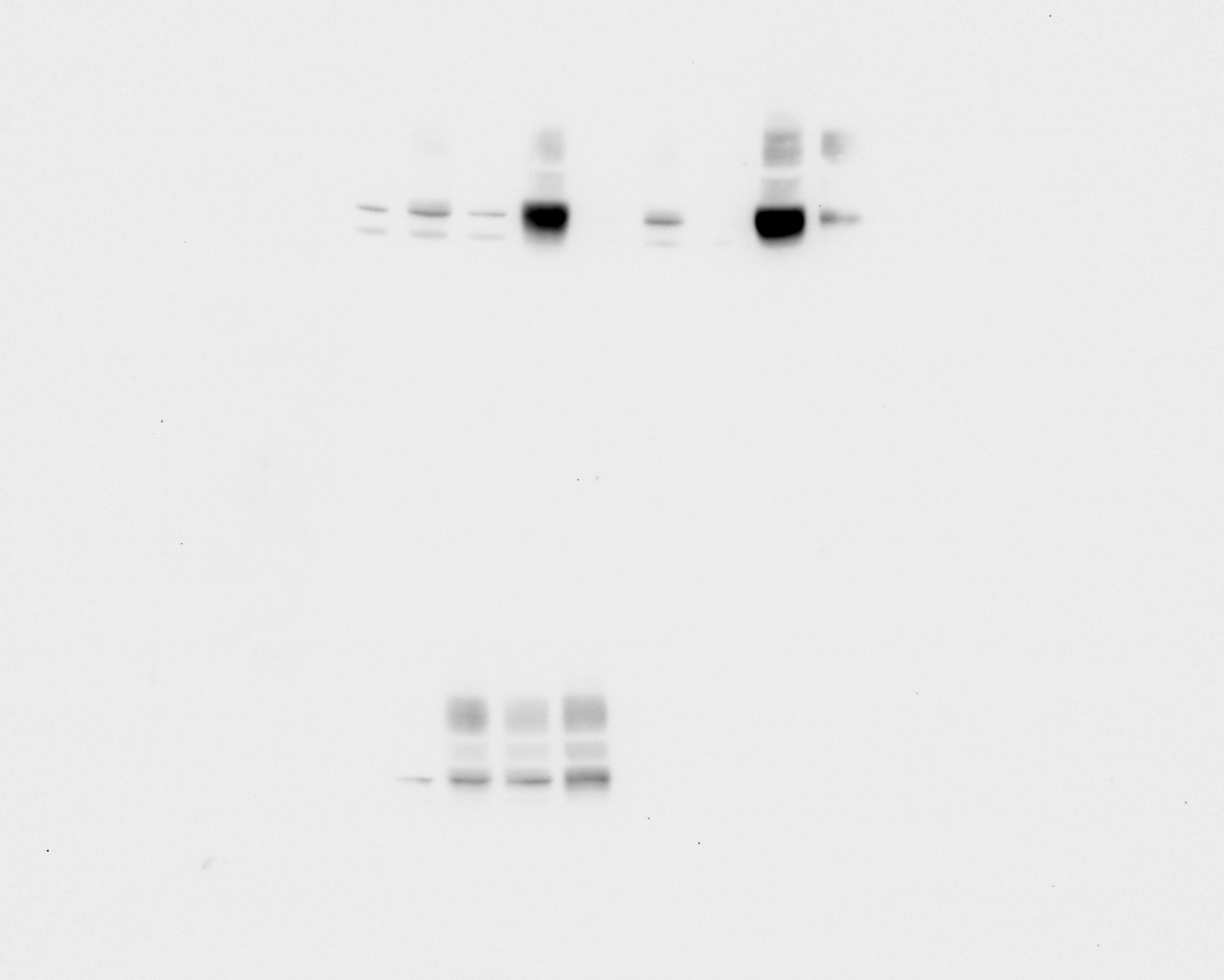

Supplement: Figure 1—source data 2. [file elife-76387-fig1-data2.zip › Figure 1- source data 2/2019-11-14 10h51m20s Chemiluminescence 103.684s transfection 2.tif]

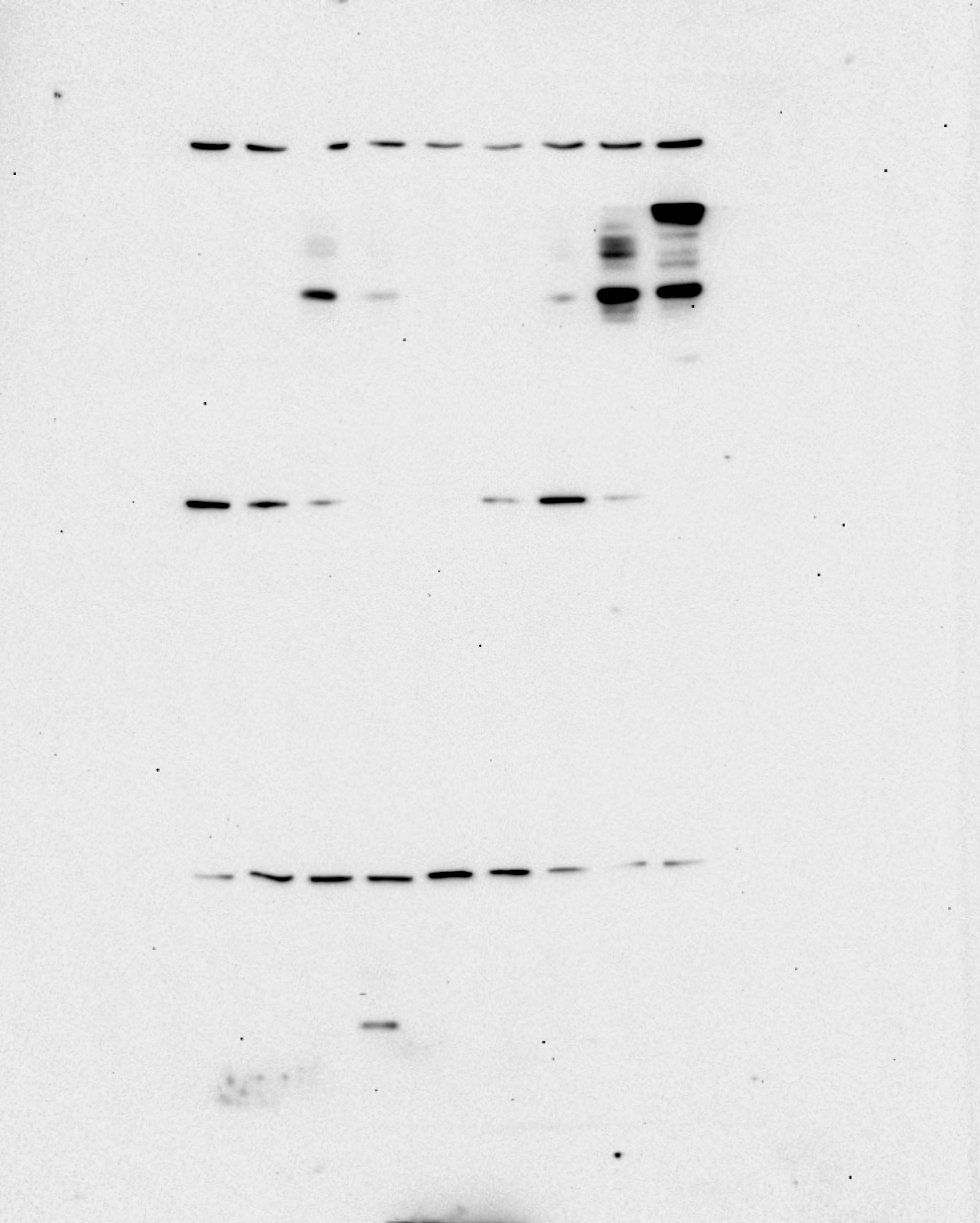

Supplement: Figure 1—source data 2. [file elife-76387-fig1-data2.zip › Figure 1- source data 2/2019-11-14 11h29m03s Chemiluminescence 852.911s vinculin.tif]

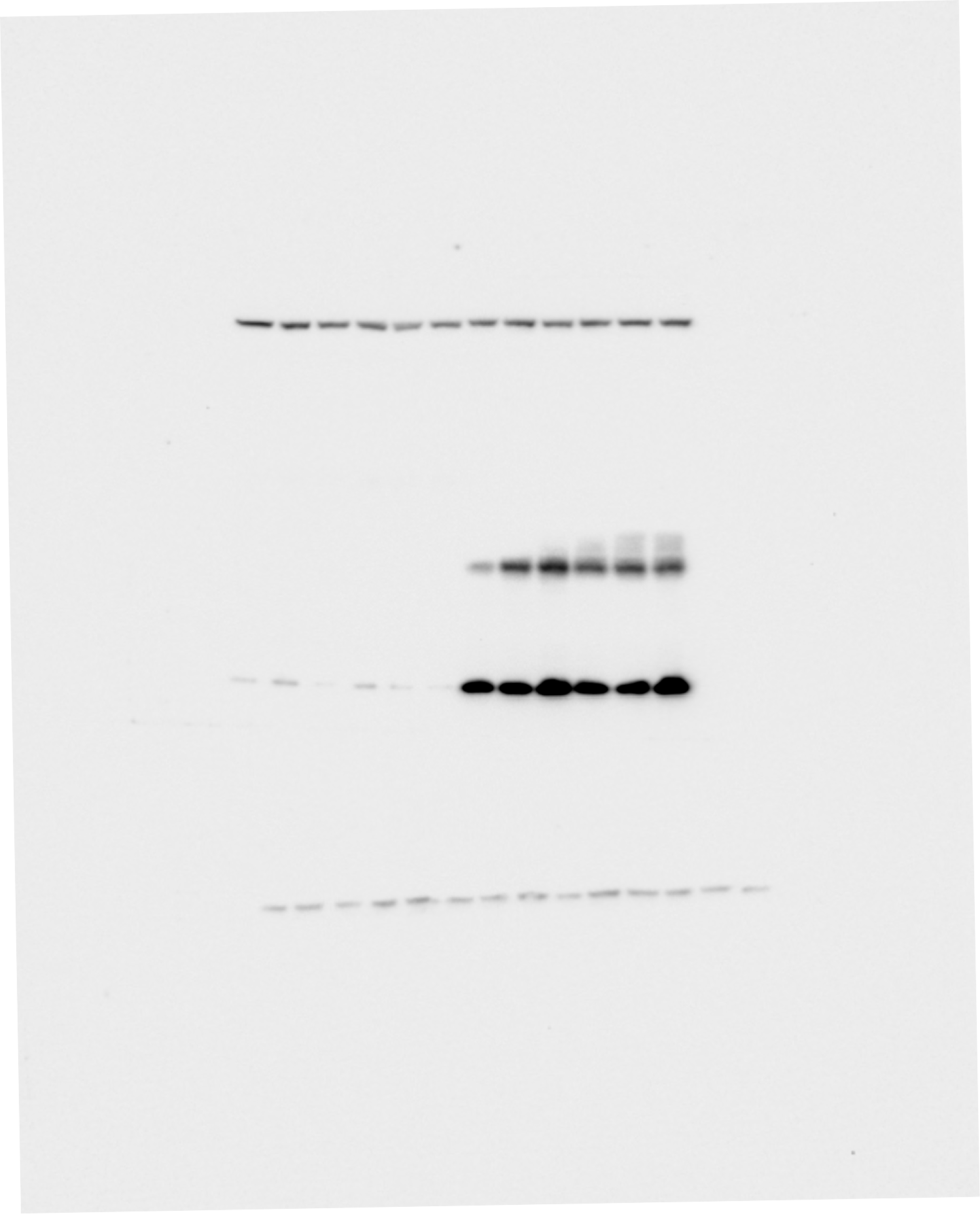

Supplement: Figure 1—source data 2. [file elife-76387-fig1-data2.zip › Figure 1- source data 2/2020-01-21 11h23m48s Chemiluminescence 10.000s p22 (1).tif]

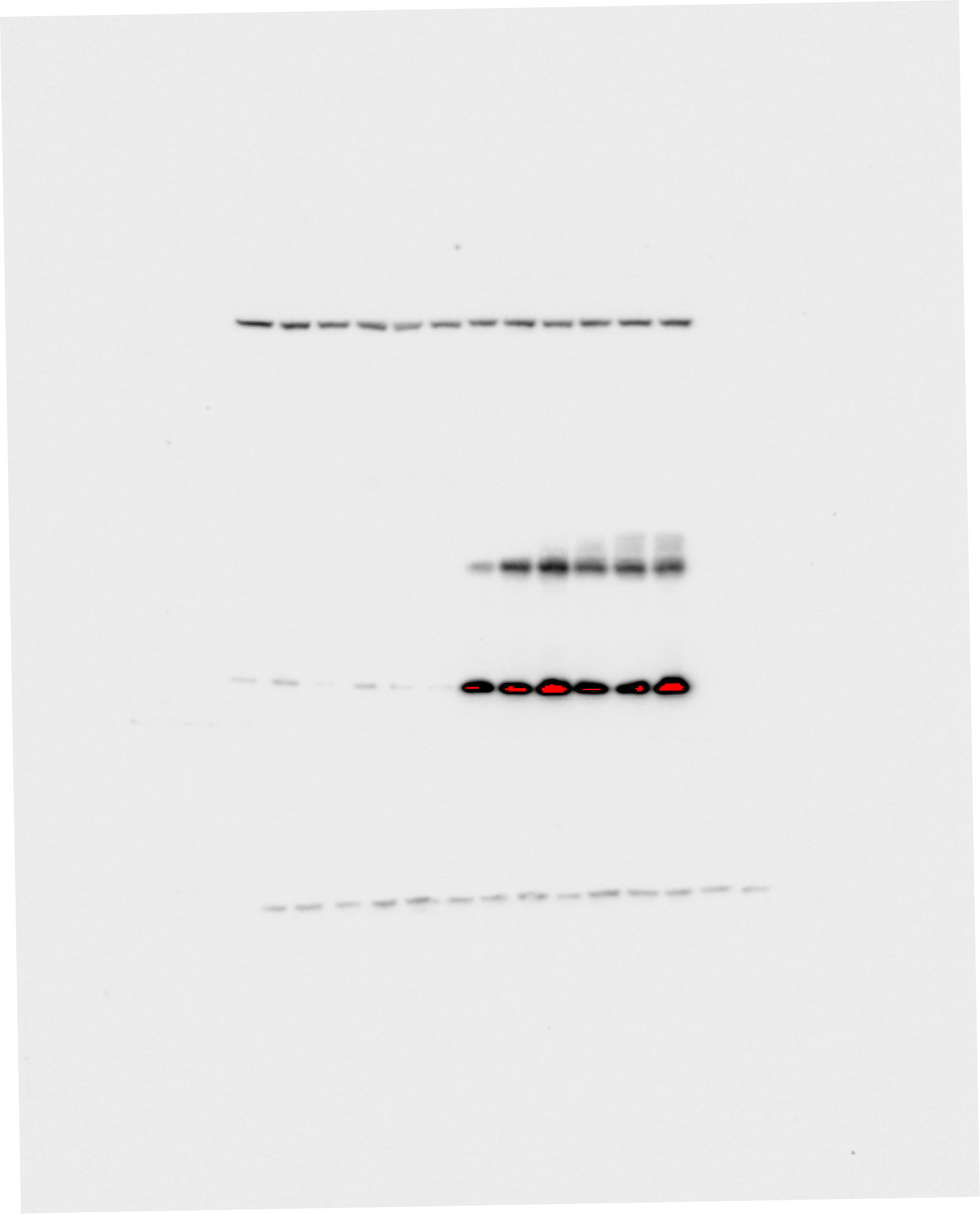

Supplement: Figure 1—source data 2. [file elife-76387-fig1-data2.zip › Figure 1- source data 2/2020-01-21 11h23m48s Chemiluminescence 10.000s vinculin.tif]

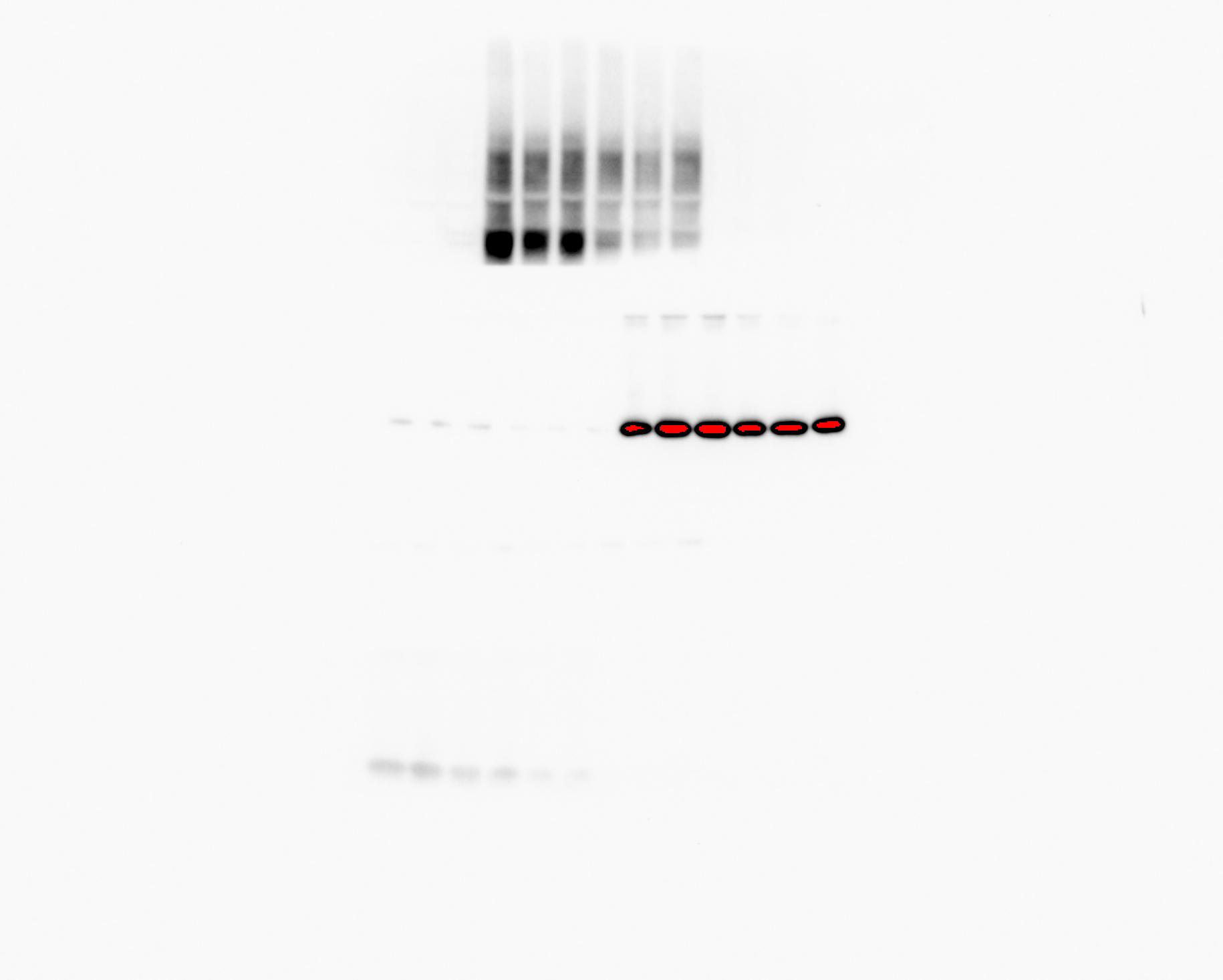

Supplement: Figure 1—source data 2. [file elife-76387-fig1-data2.zip › Figure 1- source data 2/2020-06-23 16h07m52s Chemiluminescence 10.000s gp91 x3.tif]

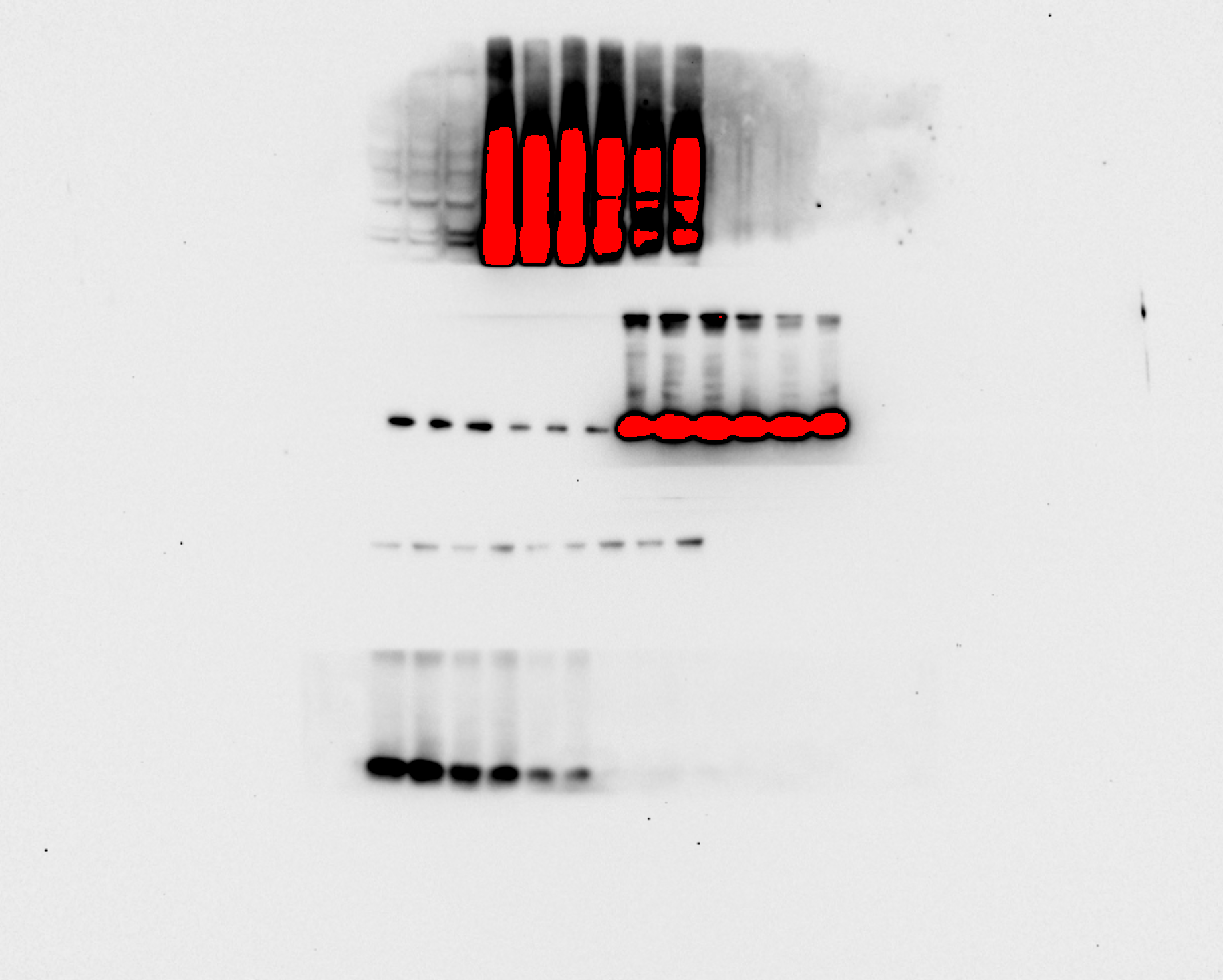

Supplement: Figure 1—source data 2. [file elife-76387-fig1-data2.zip › Figure 1- source data 2/2020-06-23 16h12m05s Chemiluminescence 183.912s eros x3.tif]

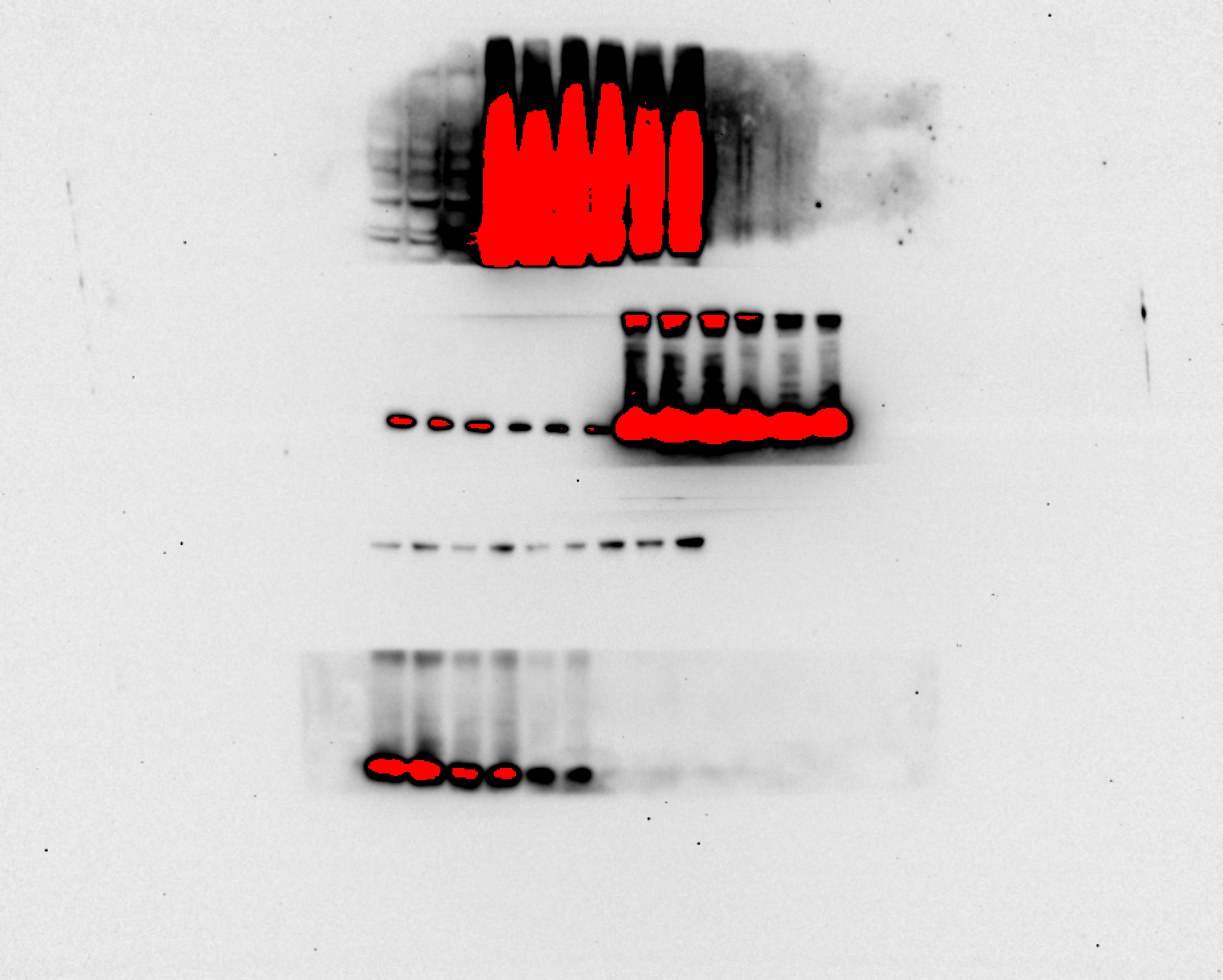

Supplement: Figure 1—source data 2. [file elife-76387-fig1-data2.zip › Figure 1- source data 2/2020-06-23 16h21m20s Chemiluminescence 722.604s actin x3.tif]

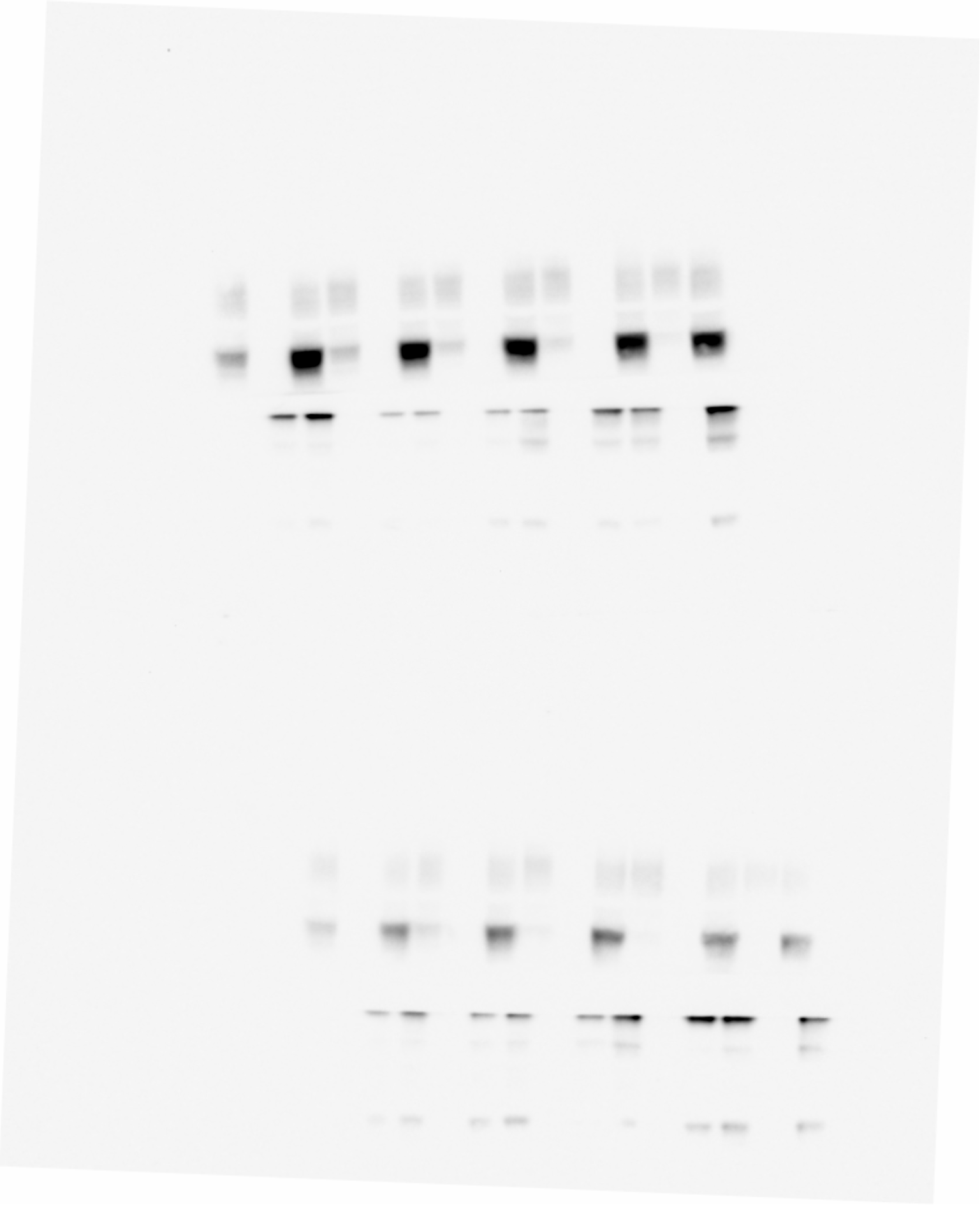

Supplement: Figure 1—source data 3. [file elife-76387-fig1-data3.zip › Figure 1- source data 3/2019-12-18 11h44m08s Chemiluminescence 10.000s gp91 exp 1 cyclo.tif]

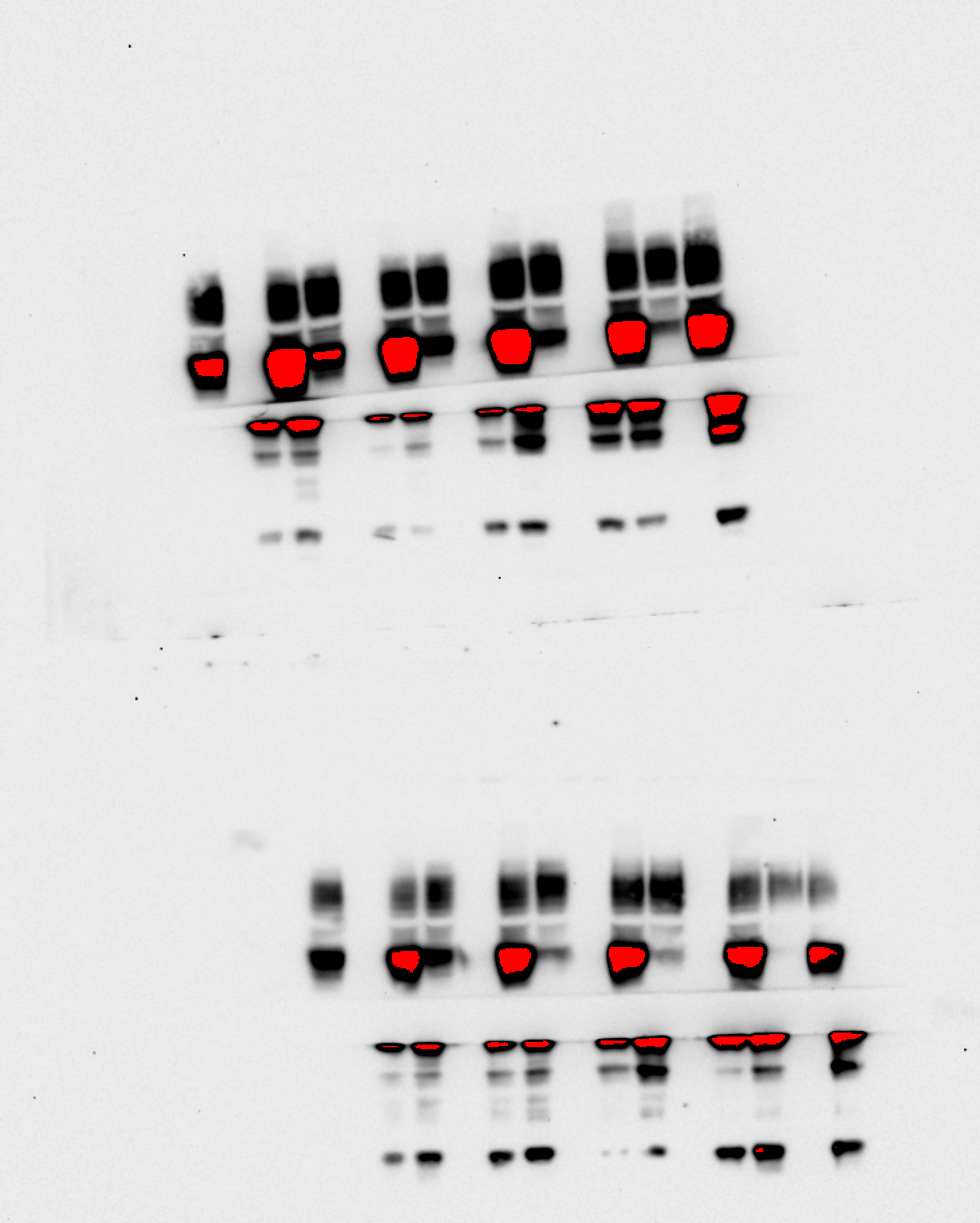

Supplement: Figure 1—source data 3. [file elife-76387-fig1-data3.zip › Figure 1- source data 3/2019-12-18 11h47m44s Chemiluminescence 216.956s eros exp 1.tif]

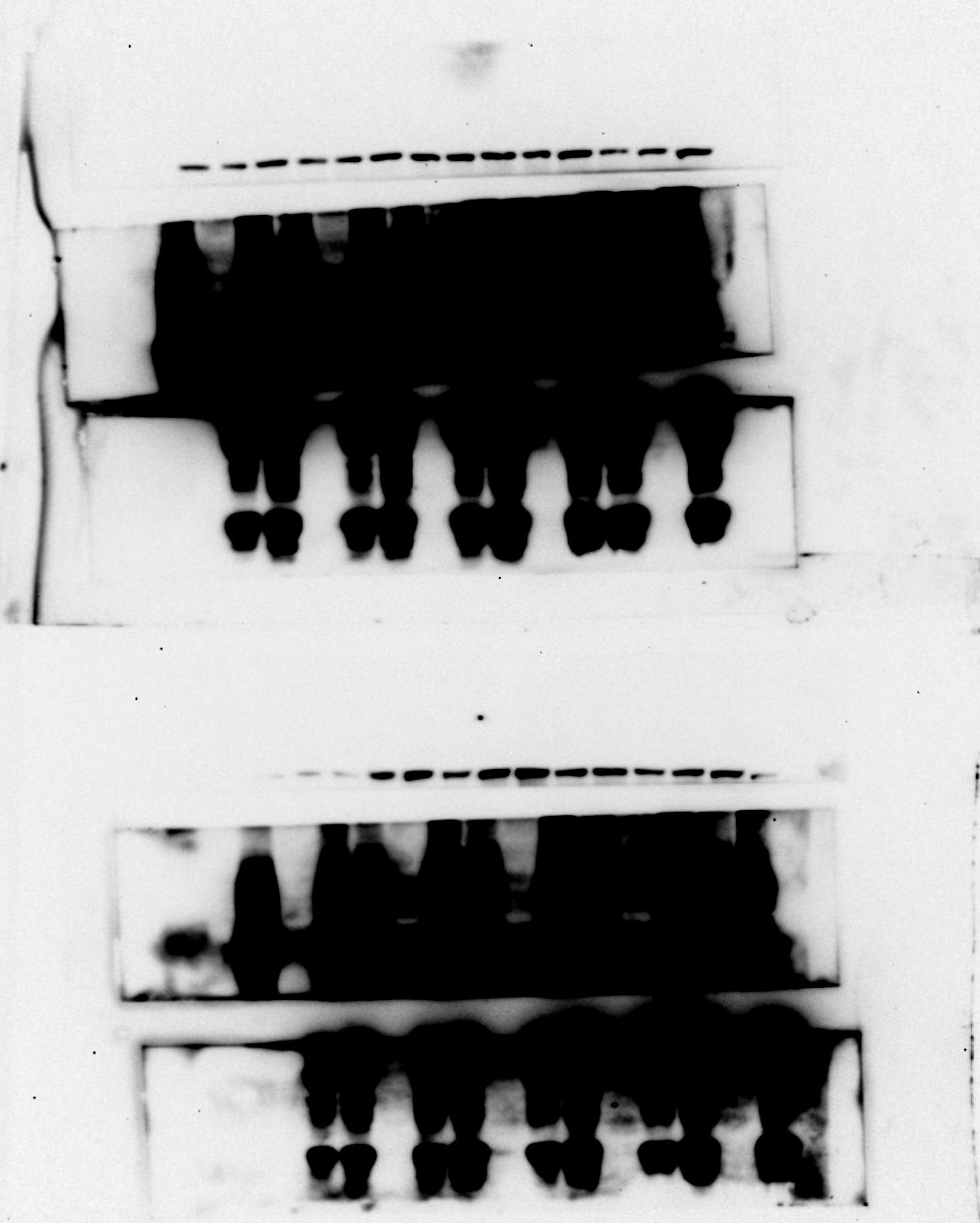

Supplement: Figure 1—source data 3. [file elife-76387-fig1-data3.zip › Figure 1- source data 3/2019-12-18 13h11m46s Chemiluminescence 745.205s vinculin exp 1 cyclo.tif]

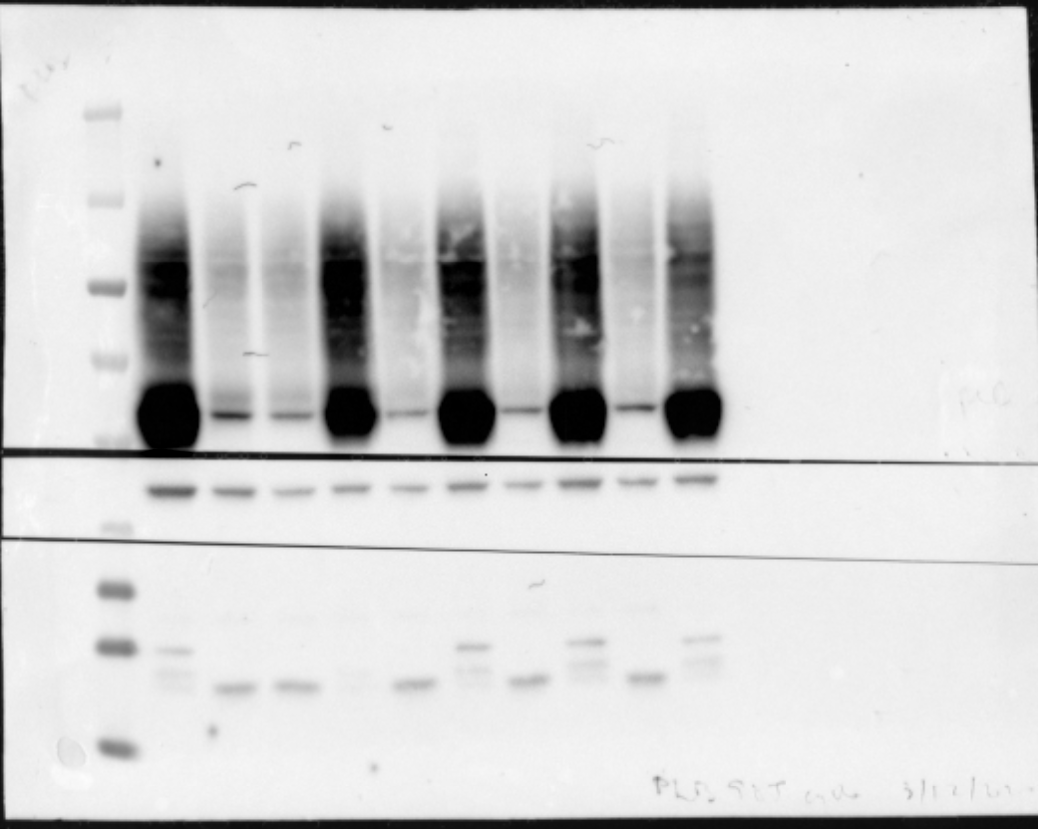

Supplement: Figure 1—source data 3. [file elife-76387-fig1-data3.zip › Figure 1- source data 3/2020-12-04 14h52m02s Colorimetric 0.046s+2020-12-04 14h53m34s Chemiluminescence 61.739s gp91.tif]

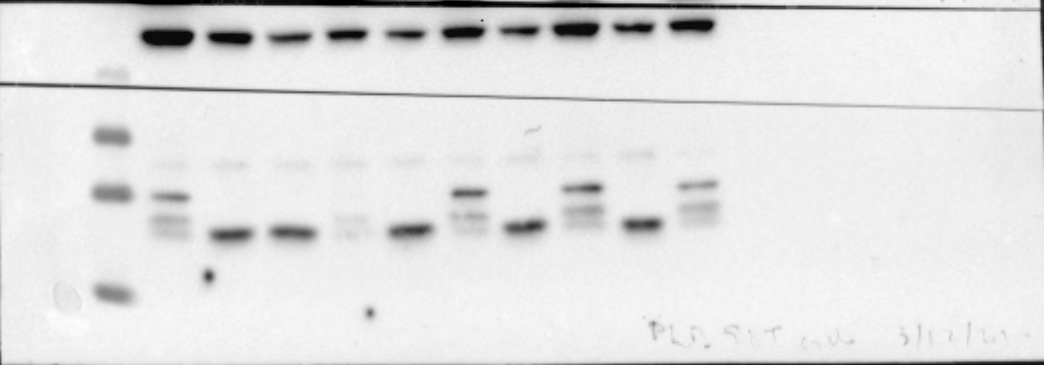

Supplement: Figure 1—source data 3. [file elife-76387-fig1-data3.zip › Figure 1- source data 3/2020-12-04 14h55m22s Chemiluminescence 165.217s+2020-12-04 14h52m02s Colorimetric 0.046s actin.tif]

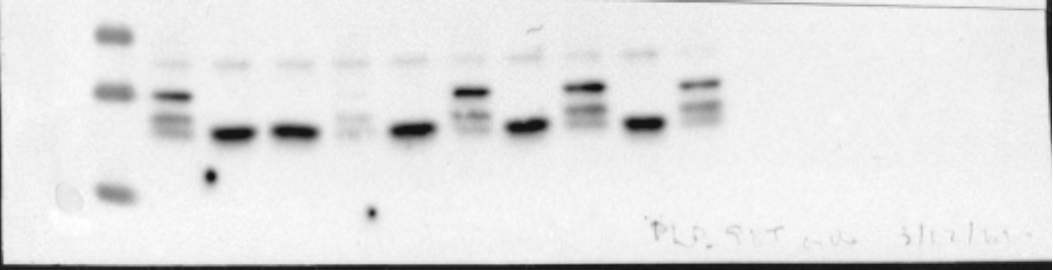

Supplement: Figure 1—source data 3. [file elife-76387-fig1-data3.zip › Figure 1- source data 3/2020-12-04 14h58m04s Chemiluminescence 320.434s+2020-12-04 14h52m02s Colorimetric 0.046s Eros.tif]

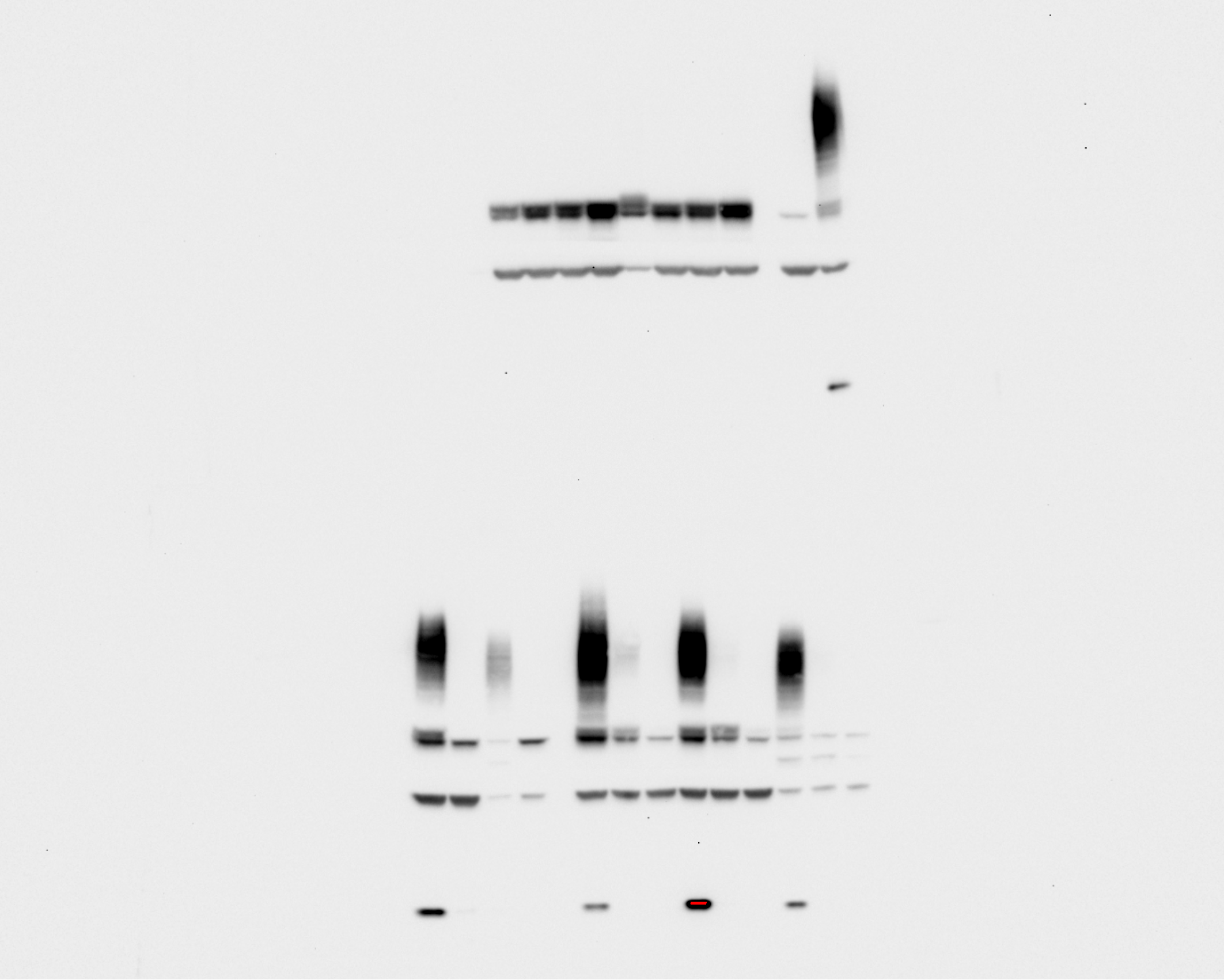

Supplement: Figure 1—source data 3. [file elife-76387-fig1-data3.zip › Figure 1- source data 3/2020-12-18 13h26m39s Chemiluminescence 91.250s PLB985 EROS KO gp91.tif]

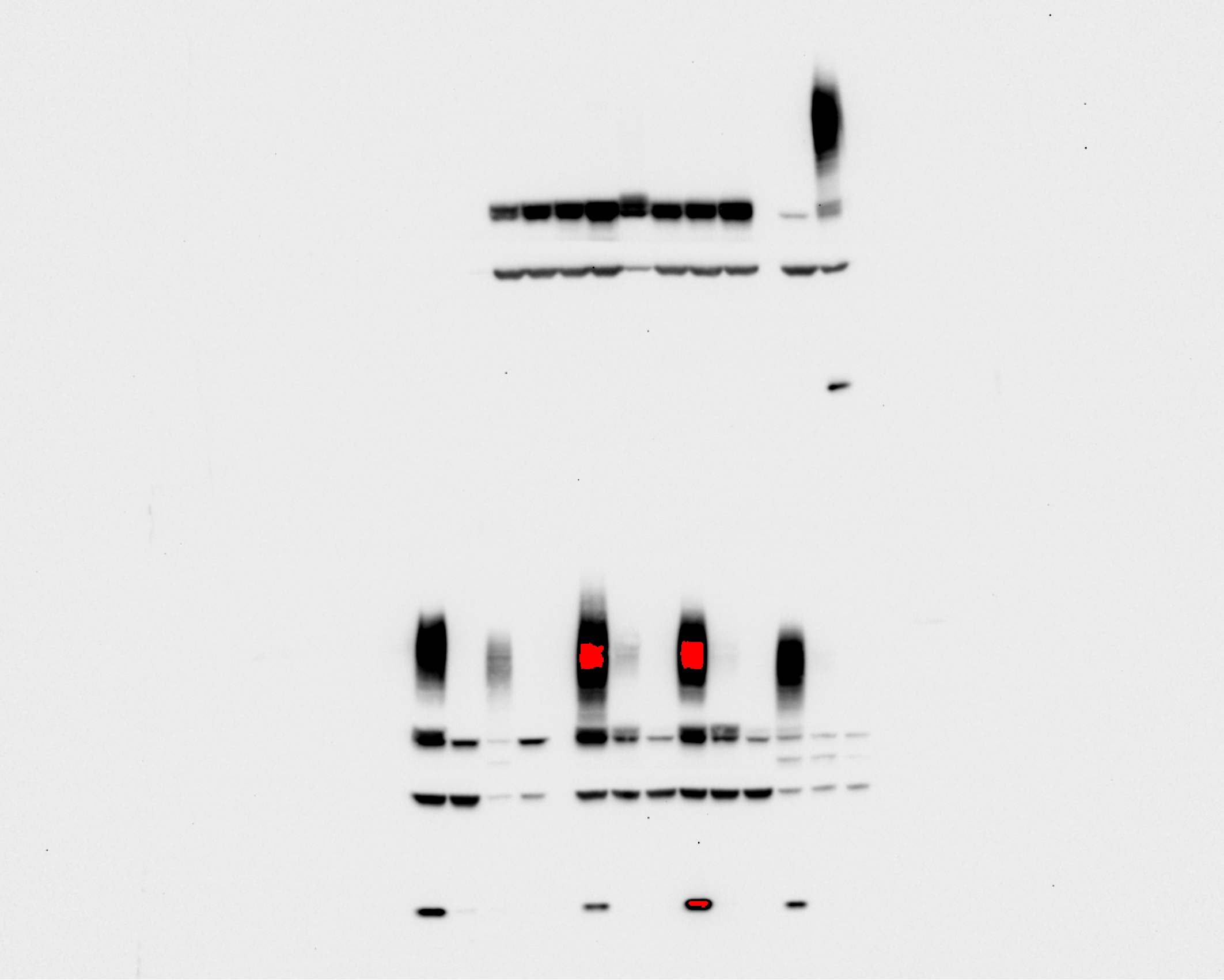

Supplement: Figure 1—source data 3. [file elife-76387-fig1-data3.zip › Figure 1- source data 3/2020-12-18 13h27m45s Chemiluminescence 152.500s PLB p22KO gp91.tif]

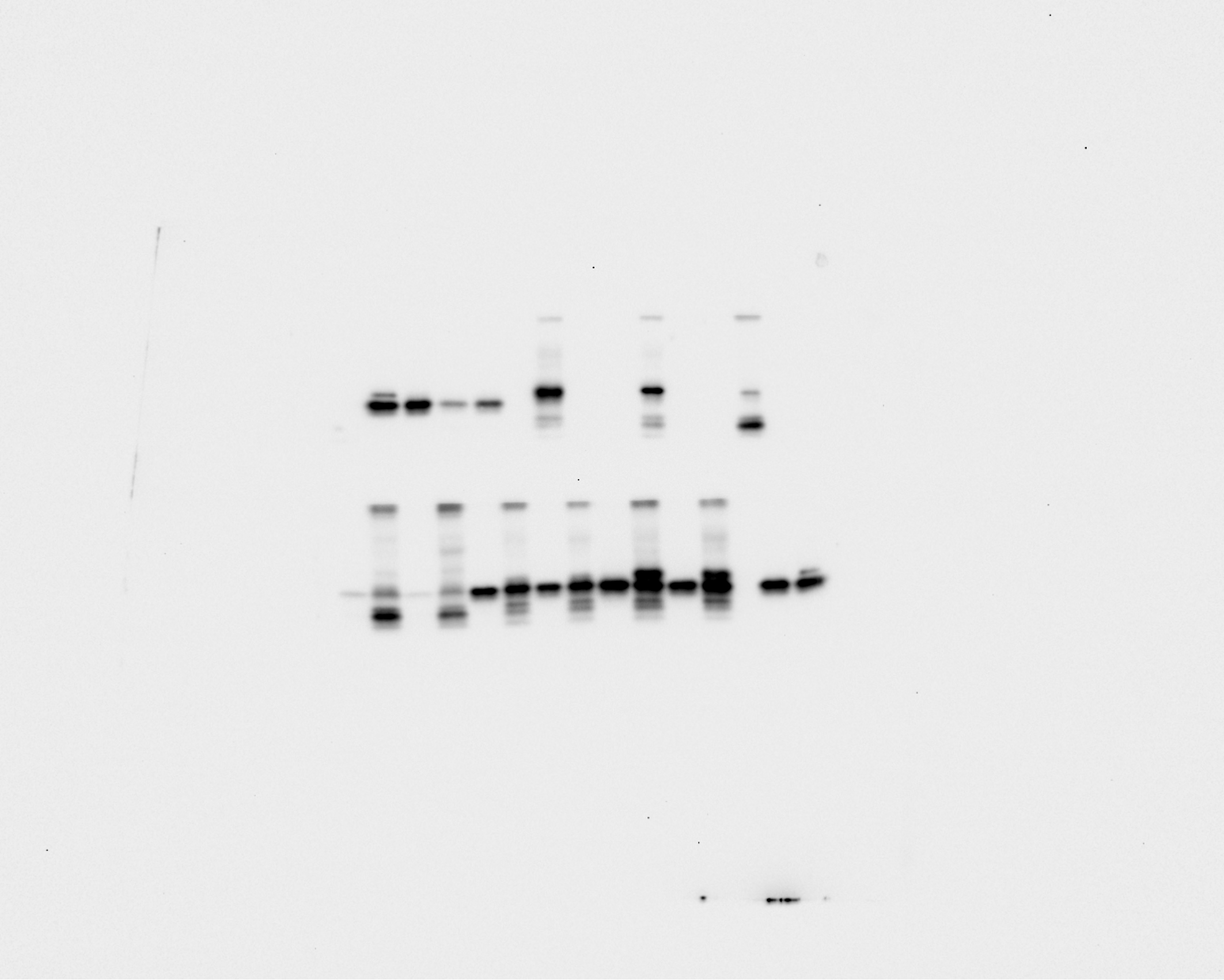

Supplement: Figure 1—source data 3. [file elife-76387-fig1-data3.zip › Figure 1- source data 3/2021-01-08 15h04m20s Chemiluminescence 157.826s PLB p22 KO EROS KO Eros.tif]

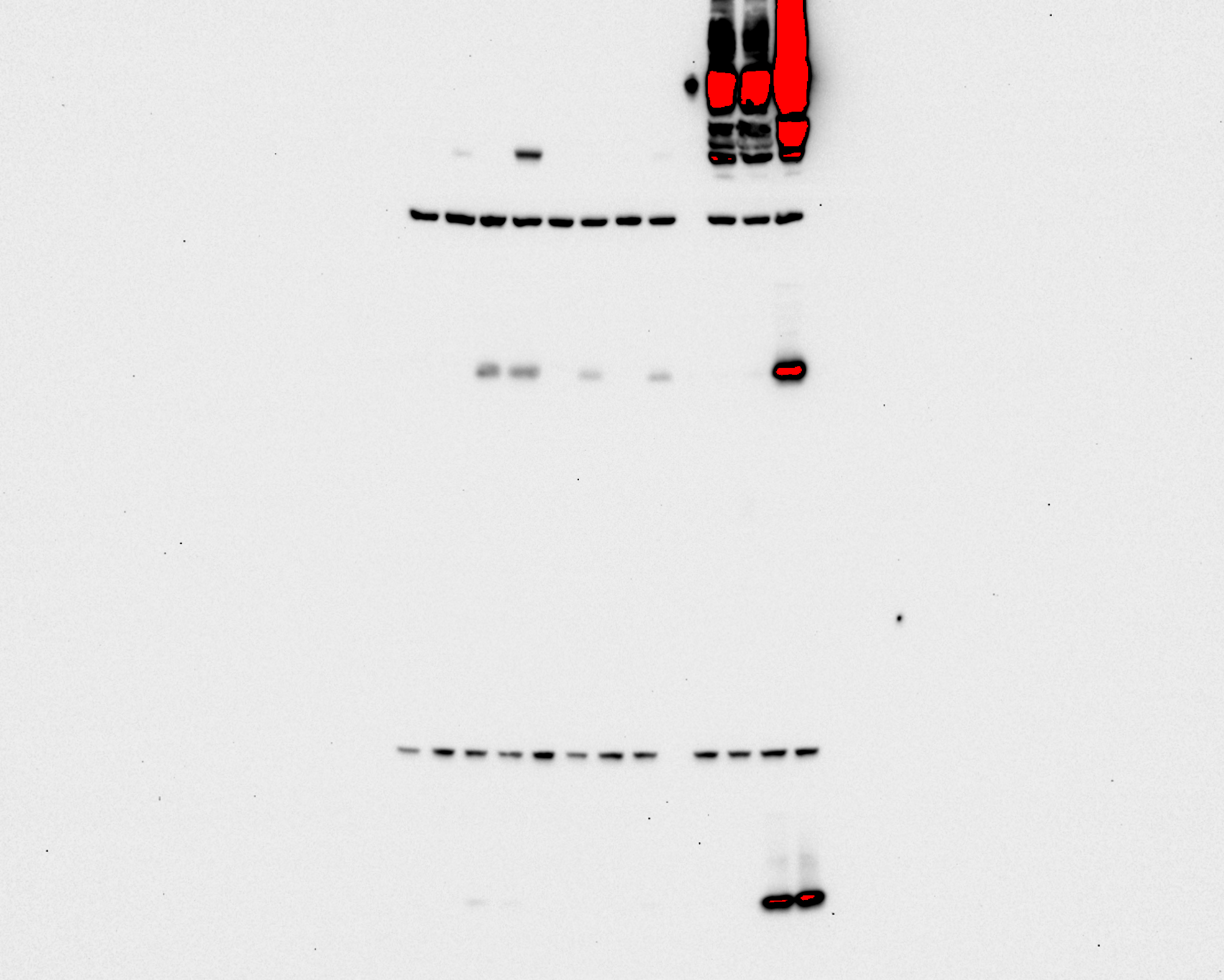

Supplement: Figure 1—figure supplement 1—source data 1. [file elife-76387-fig1-figsupp1-data1.zip › Figure 1 figure supplement 1- source data 1/2020-06-24 15h57m00s Chemiluminescence 953.472s NIH3T3 actin.tif]

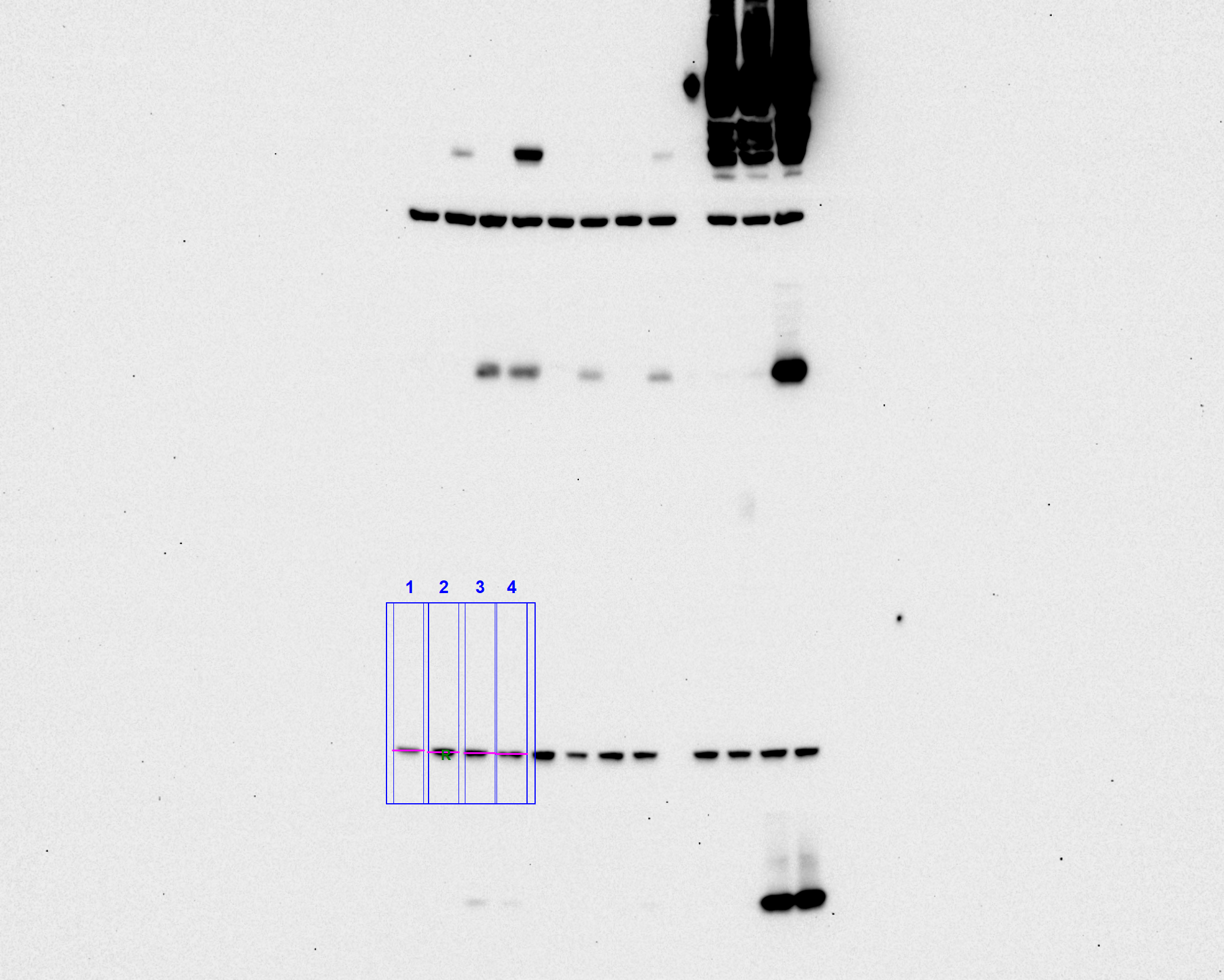

Supplement: Figure 1—figure supplement 1—source data 1. [file elife-76387-fig1-figsupp1-data1.zip › Figure 1 figure supplement 1- source data 1/2020-06-24 16h11m59s Chemiluminescence 1799.988s NIH3T3 gp91.tif]

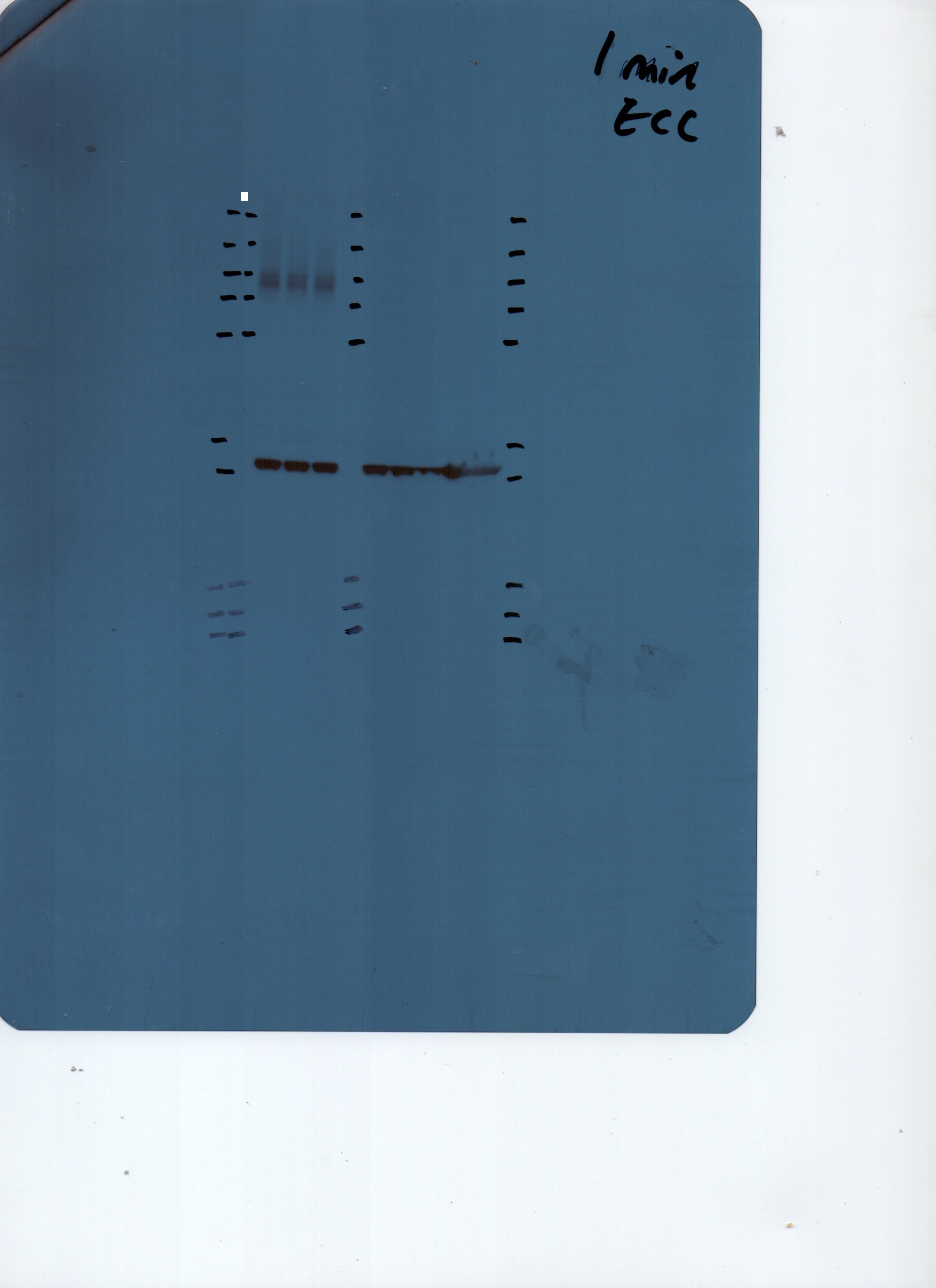

Supplement: Figure 1—figure supplement 1—source data 1. [file elife-76387-fig1-figsupp1-data1.zip › Figure 1 figure supplement 1- source data 1/EROS magic 20.12.18 3.tif]

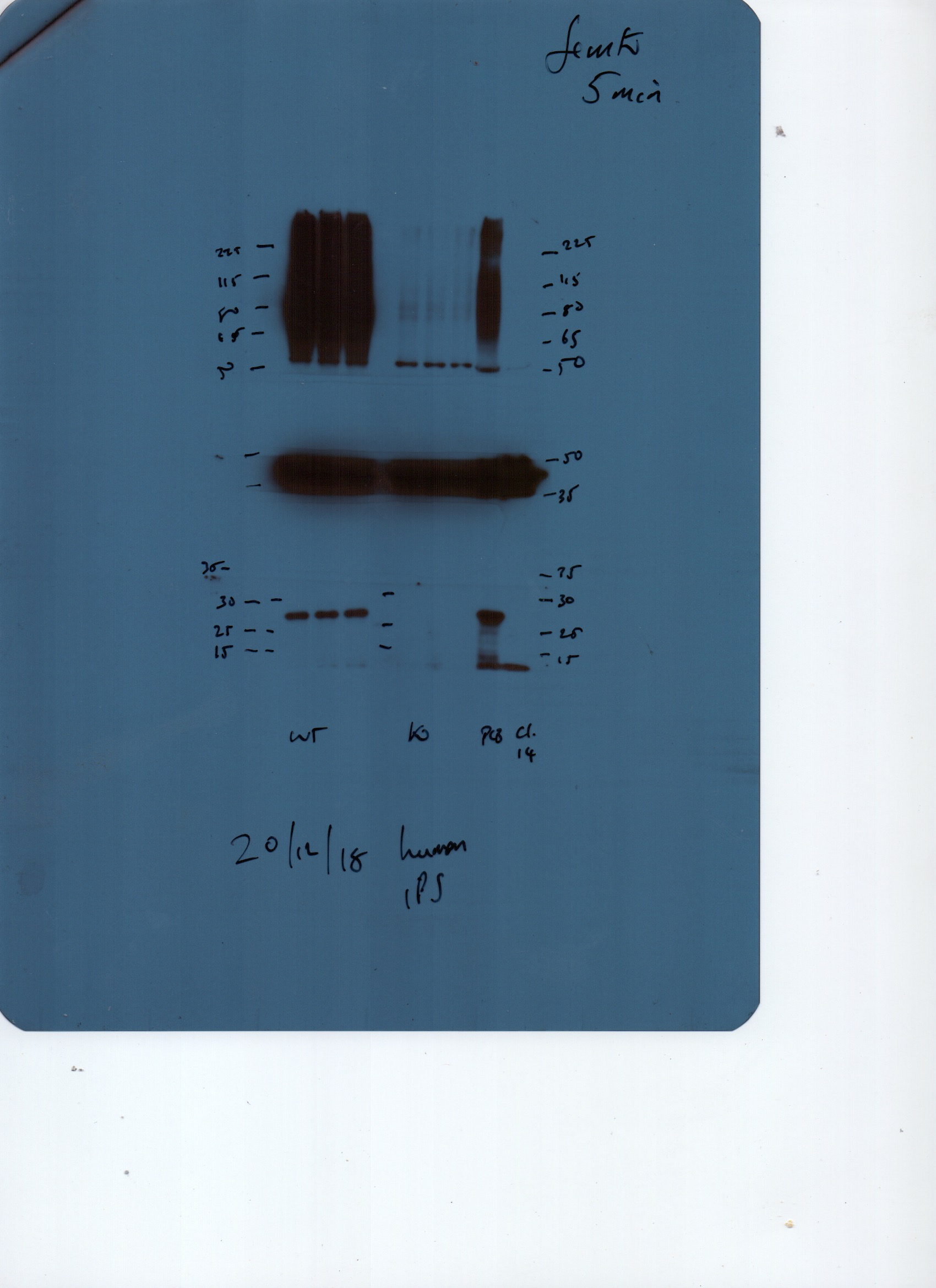

Supplement: Figure 1—figure supplement 1—source data 1. [file elife-76387-fig1-figsupp1-data1.zip › Figure 1 figure supplement 1- source data 1/EROS magic 20.12.18 4.tif]

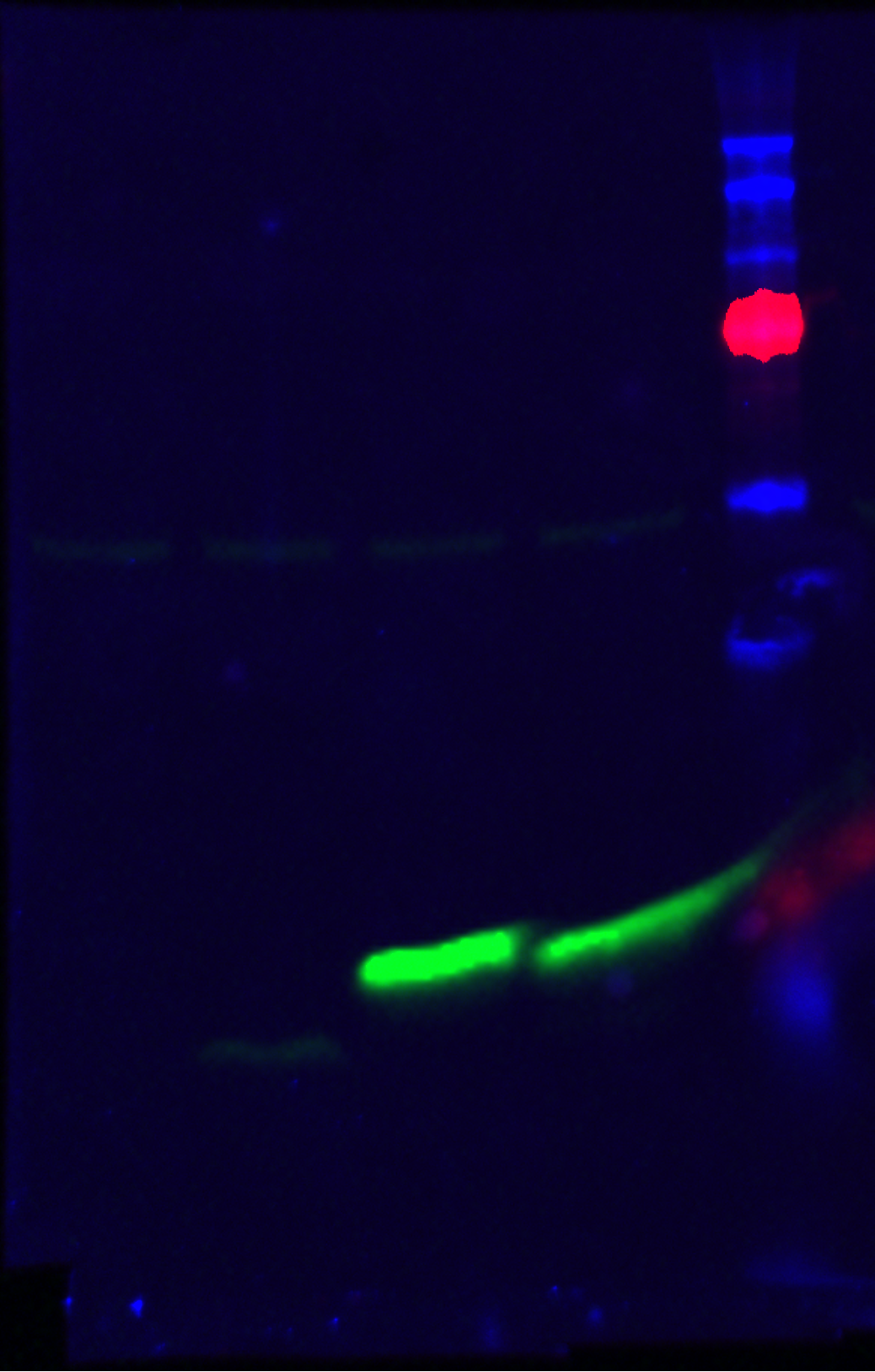

Supplement: Figure 1—figure supplement 1—source data 1. [file elife-76387-fig1-figsupp1-data1.zip › Figure 1 figure supplement 1- source data 1/SI-Fig1C EROS.tif]

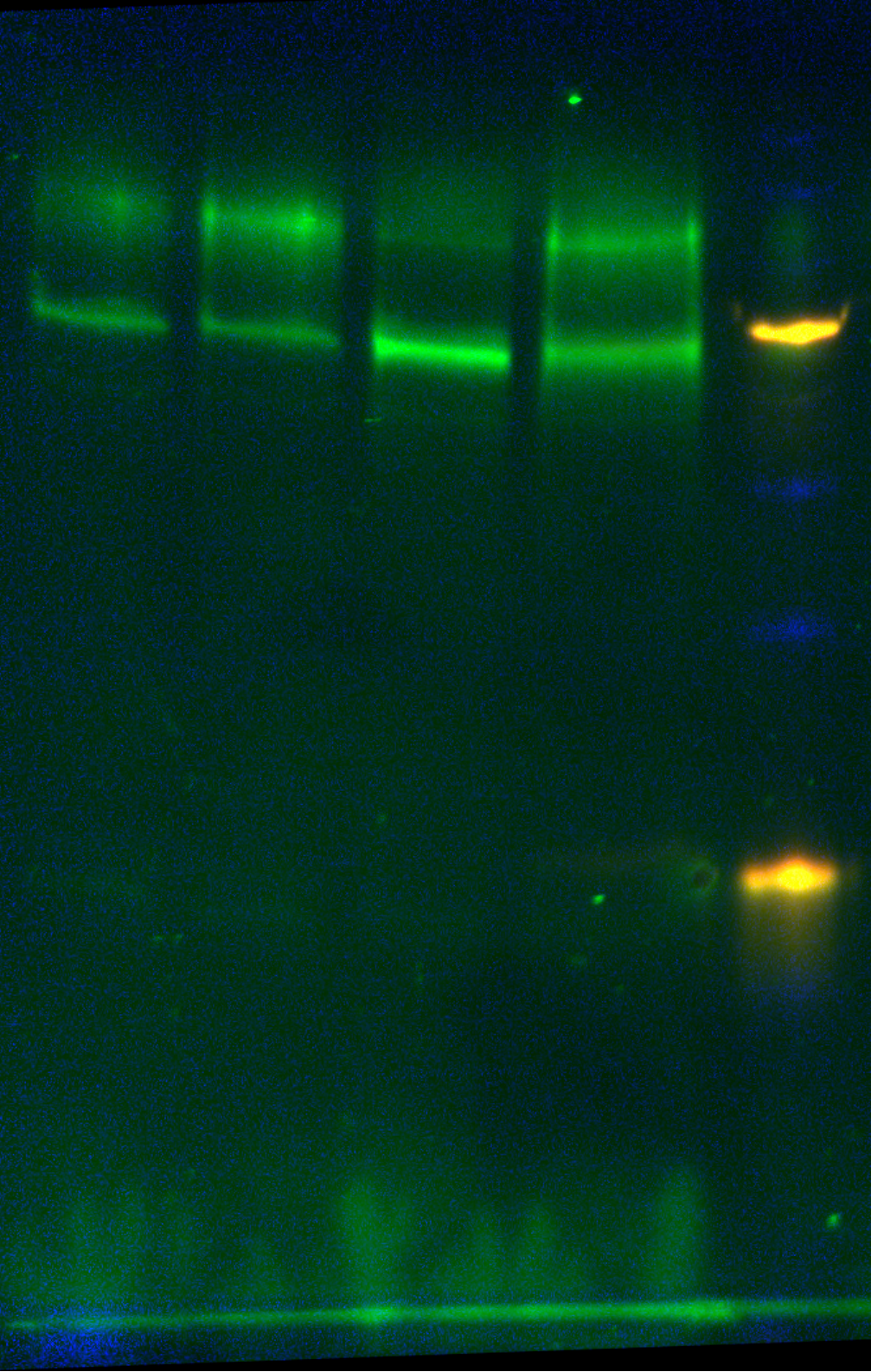

Supplement: Figure 1—figure supplement 1—source data 1. [file elife-76387-fig1-figsupp1-data1.zip › Figure 1 figure supplement 1- source data 1/SI-Fig1C NOX2 (GFP).tif]

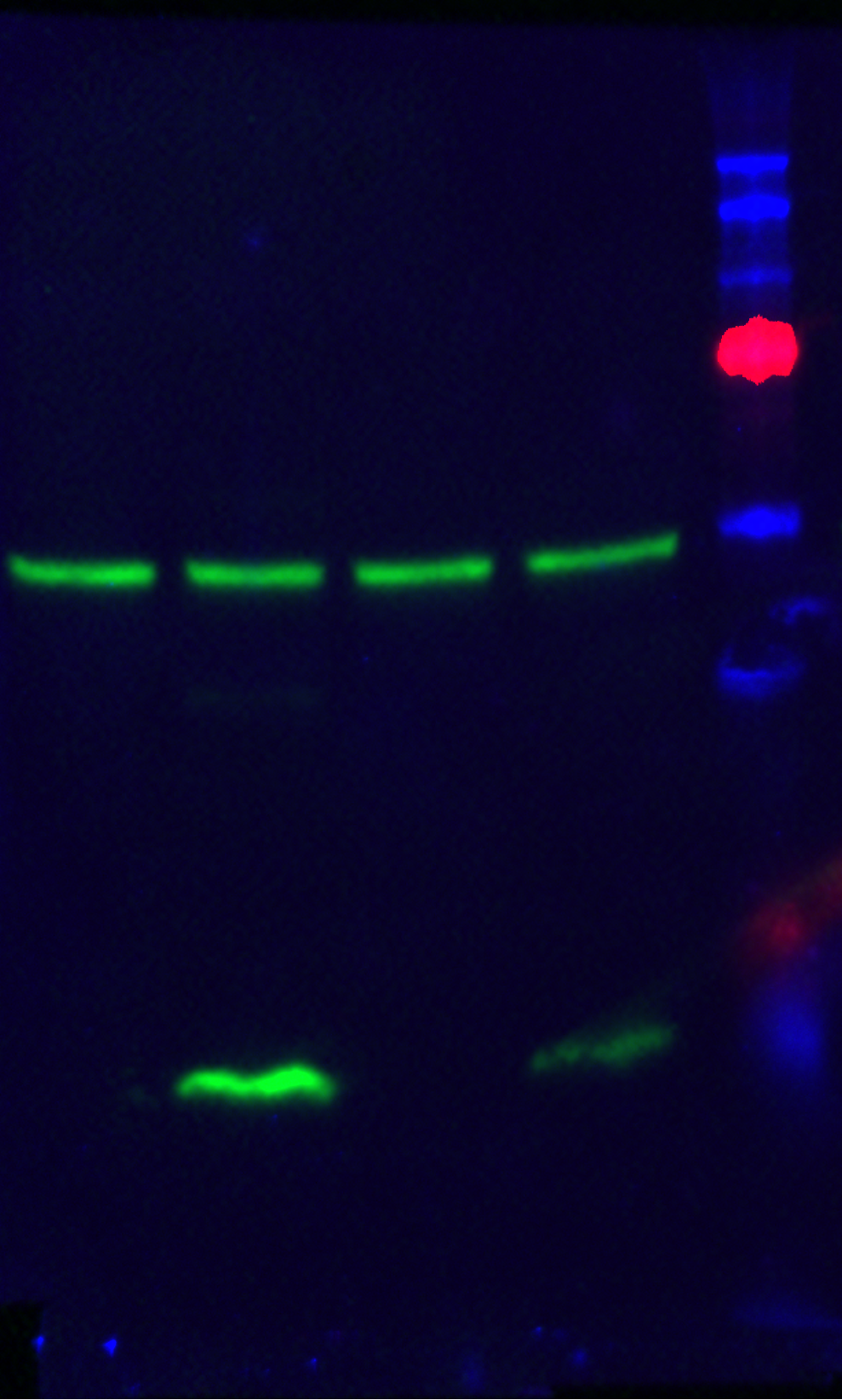

Supplement: Figure 1—figure supplement 1—source data 1. [file elife-76387-fig1-figsupp1-data1.zip › Figure 1 figure supplement 1- source data 1/SI-Fig1C tubulin and anti-p22.tif]

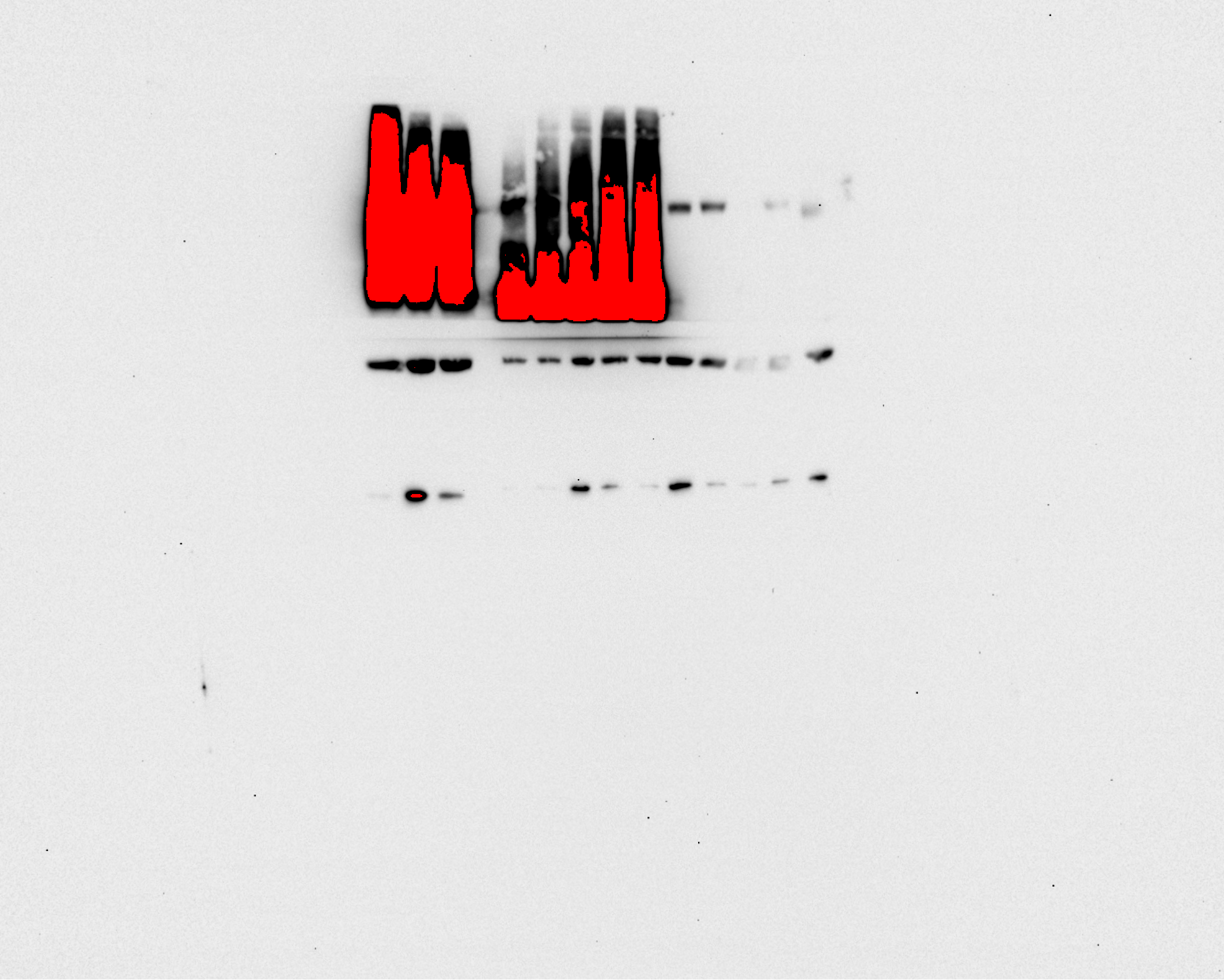

Supplement: Figure 1—figure supplement 1—source data 2. [file elife-76387-fig1-figsupp1-data2.zip › Figure 1 figure supplement 1- source data 2/2020-07-22 12h04m53s Chemiluminescence 786.085s BMDM gp91 KO actin.tif]

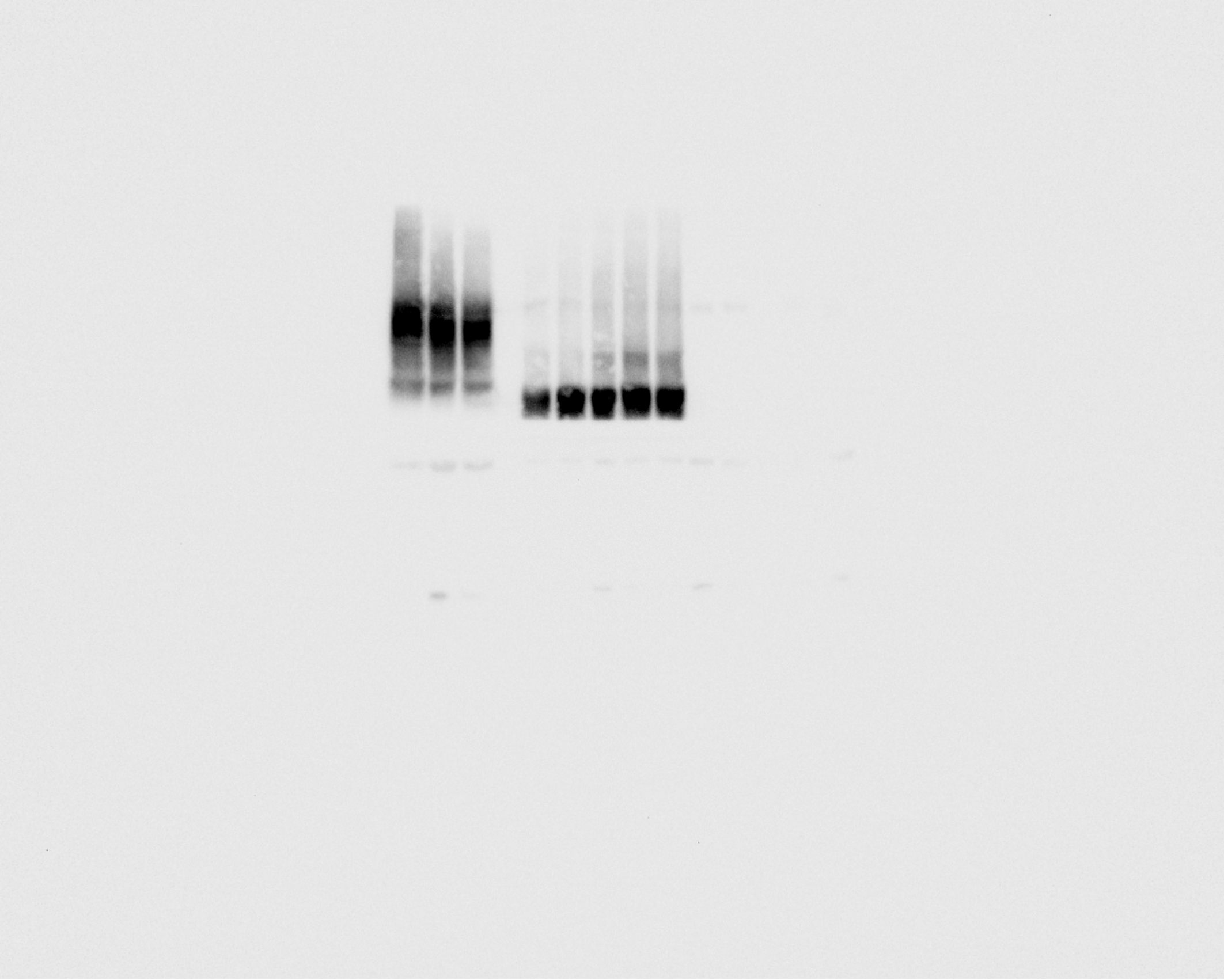

Supplement: Figure 1—figure supplement 1—source data 2. [file elife-76387-fig1-figsupp1-data2.zip › Figure 1 figure supplement 1- source data 2/2020-07-22 12h09m39s Chemiluminescence 8.000s BMDM gp91 KO gp91.tif]

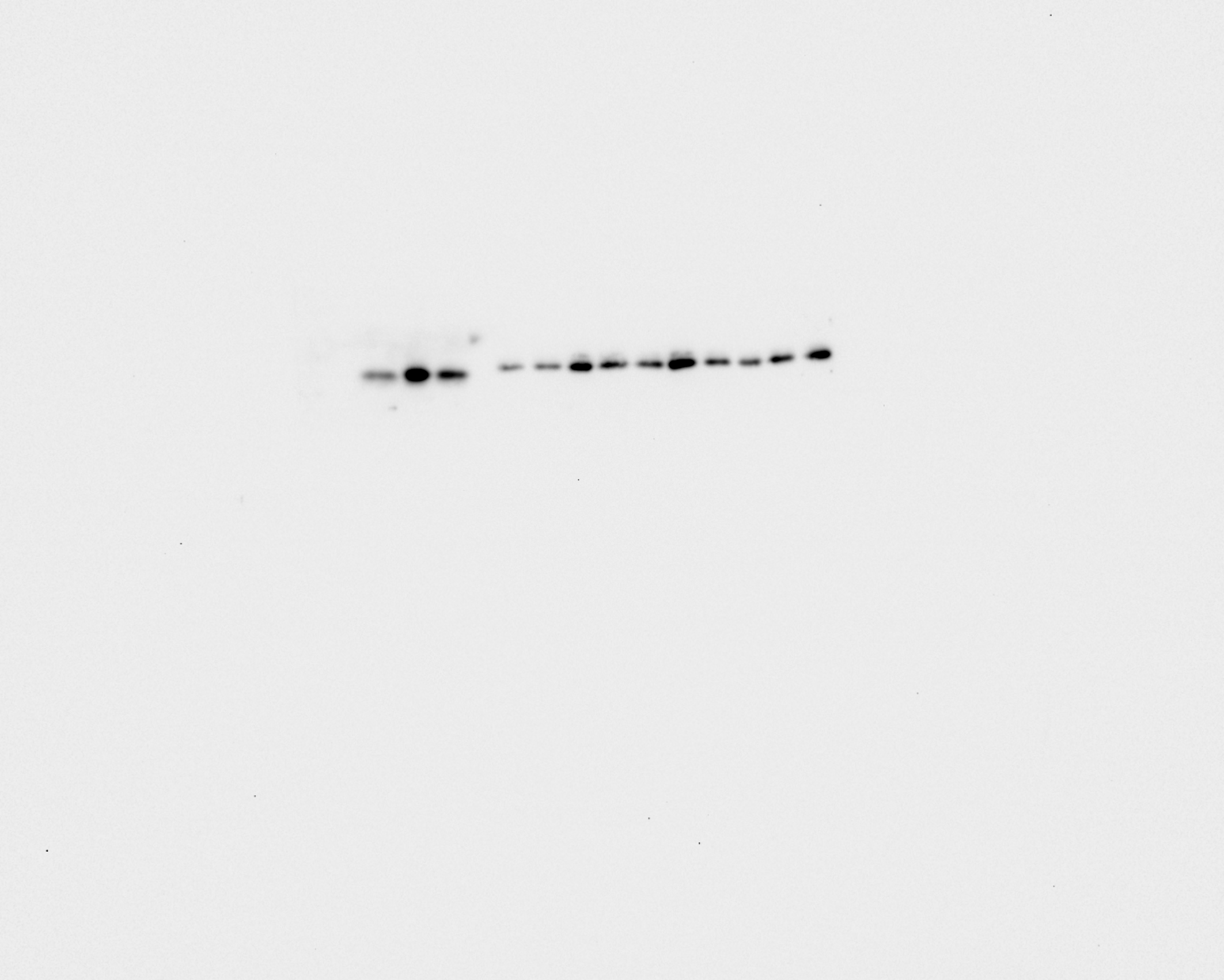

Supplement: Figure 1—figure supplement 1—source data 2. [file elife-76387-fig1-figsupp1-data2.zip › Figure 1 figure supplement 1- source data 2/2020-07-22 12h18m37s Chemiluminescence 102.067s BMDM gp91 KO eros.tif]

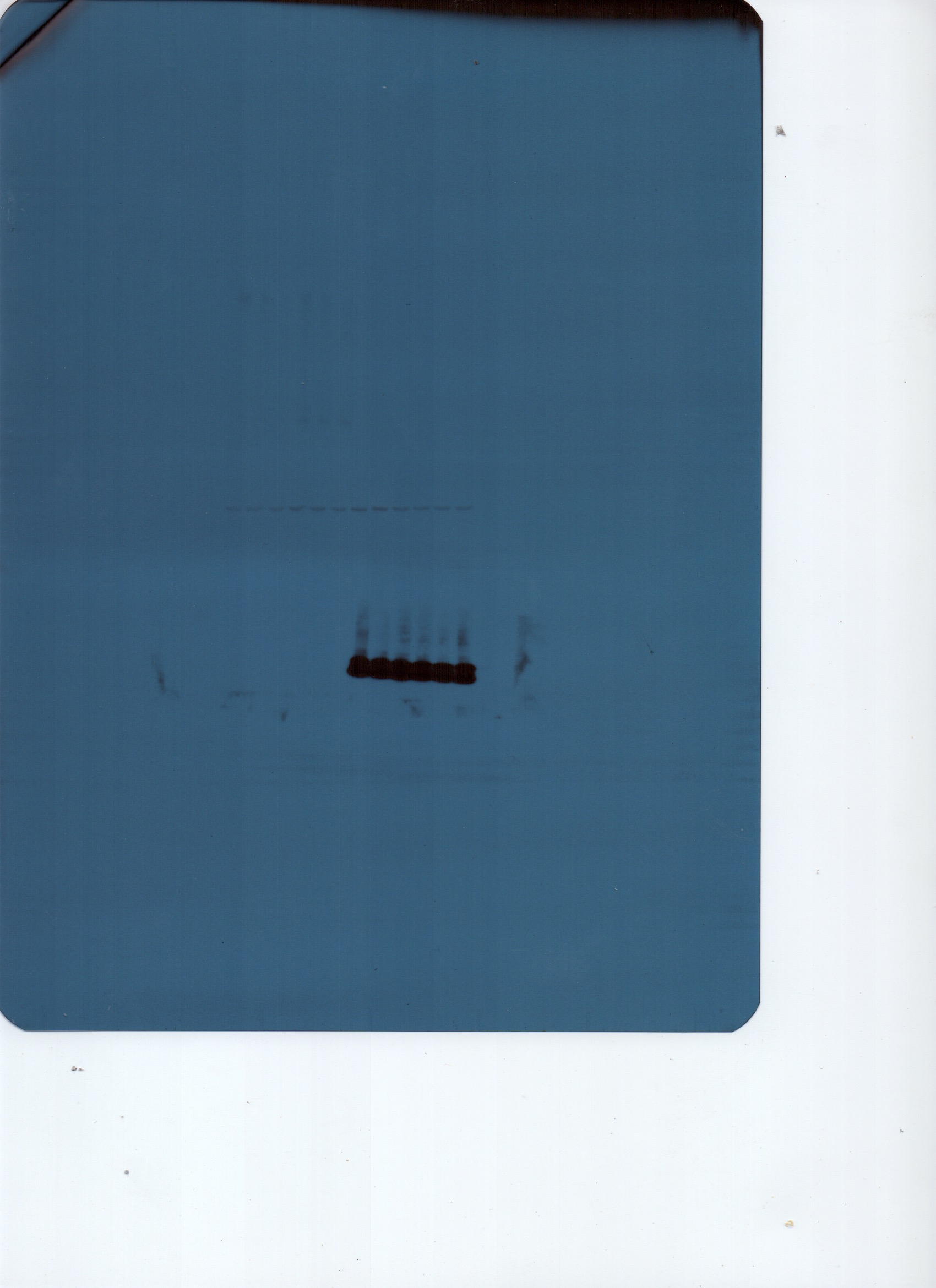

Supplement: Figure 1—figure supplement 1—source data 2. [file elife-76387-fig1-figsupp1-data2.zip › Figure 1 figure supplement 1- source data 2/gp91 EROS co-exp HEKs 1.2.19 2.tif]

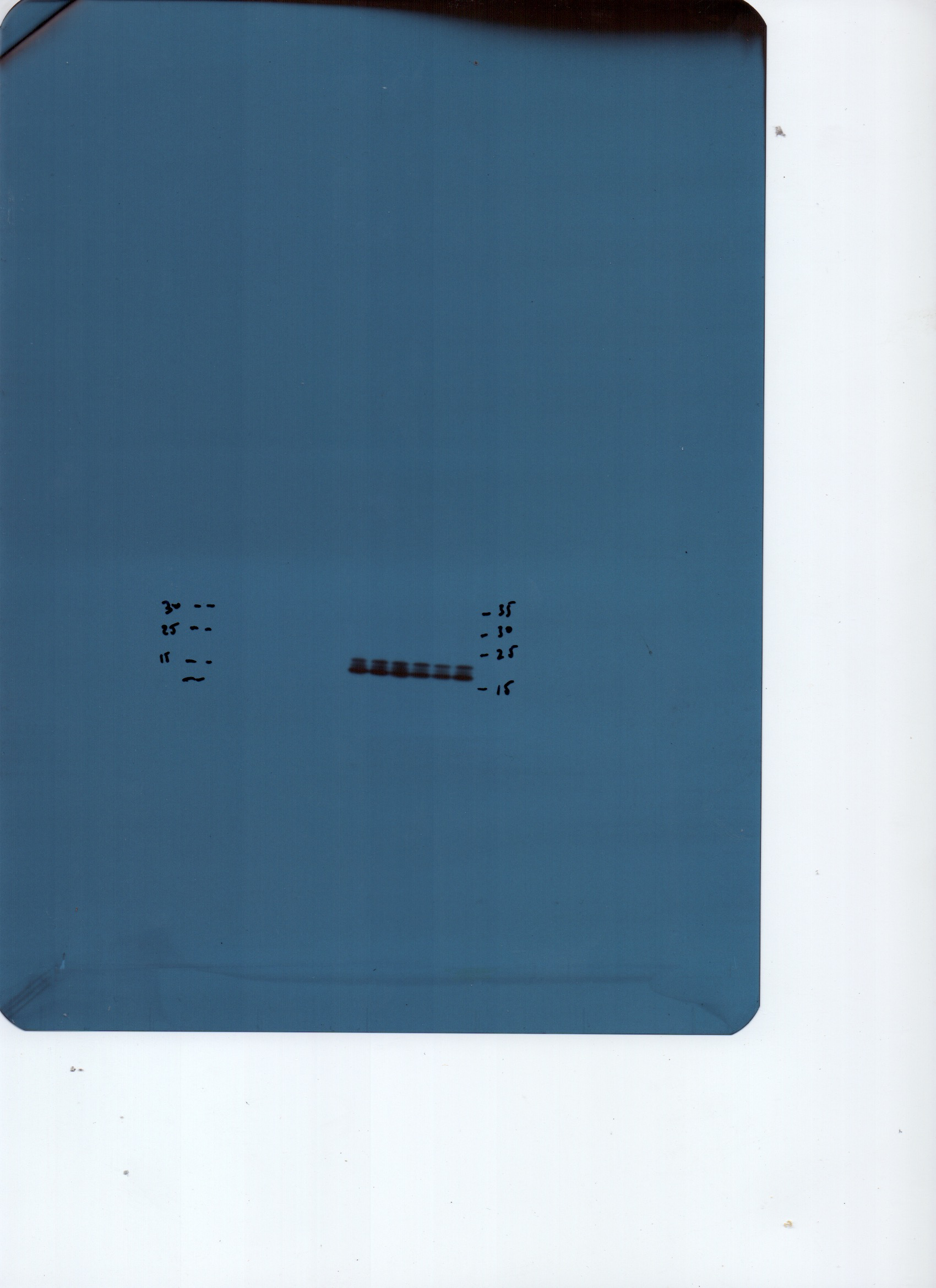

Supplement: Figure 1—figure supplement 1—source data 2. [file elife-76387-fig1-figsupp1-data2.zip › Figure 1 figure supplement 1- source data 2/gp91 EROS co-exp HEKs 1.2.19 4.tif]

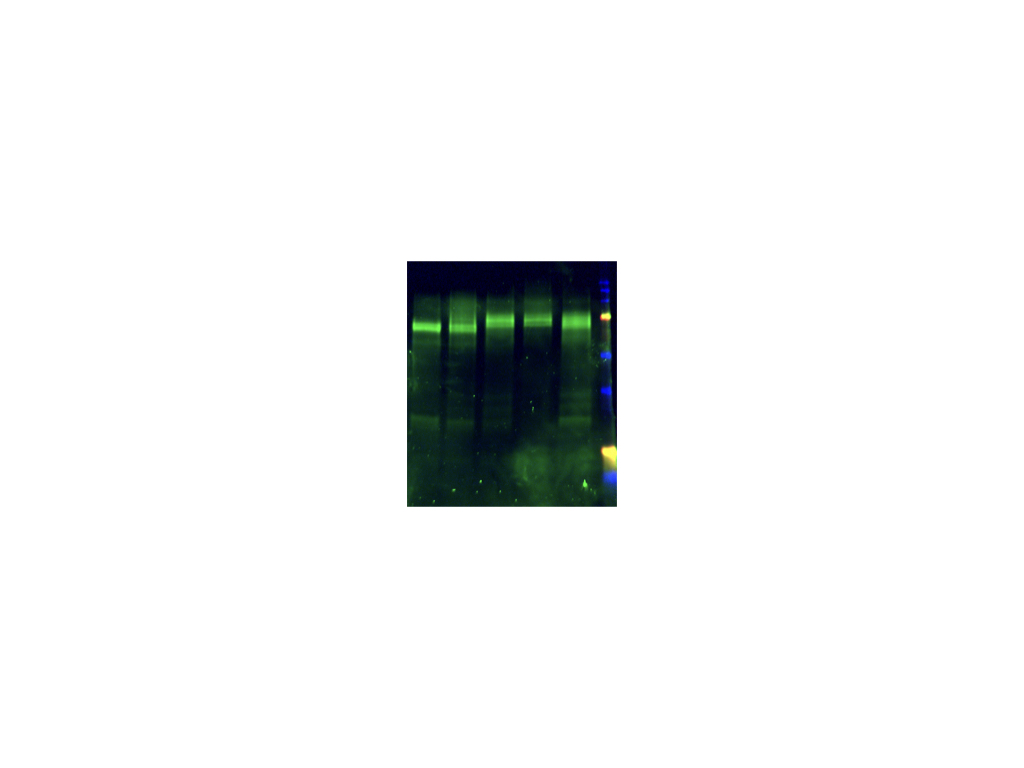

Supplement: Figure 2—source data 1. [file elife-76387-fig2-data1.zip › Figure 2- source data 1/Fig 2A.jpeg]

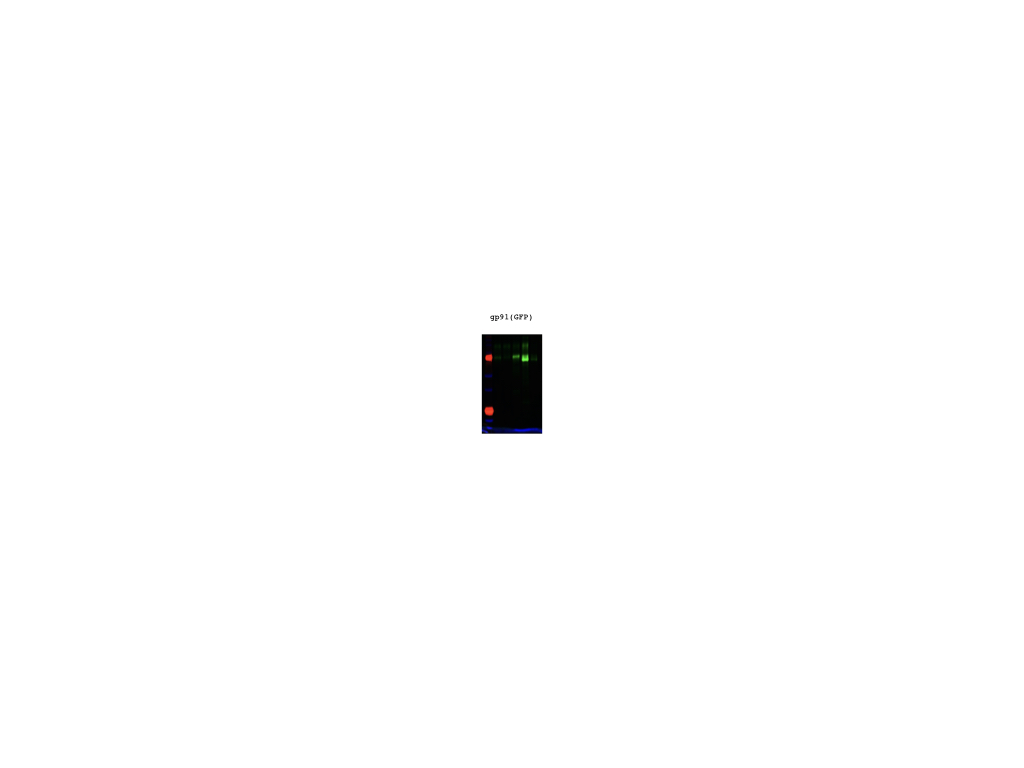

Supplement: Figure 2—source data 1. [file elife-76387-fig2-data1.zip › Figure 2- source data 1/Fig 2C gp91(GFP).jpeg]

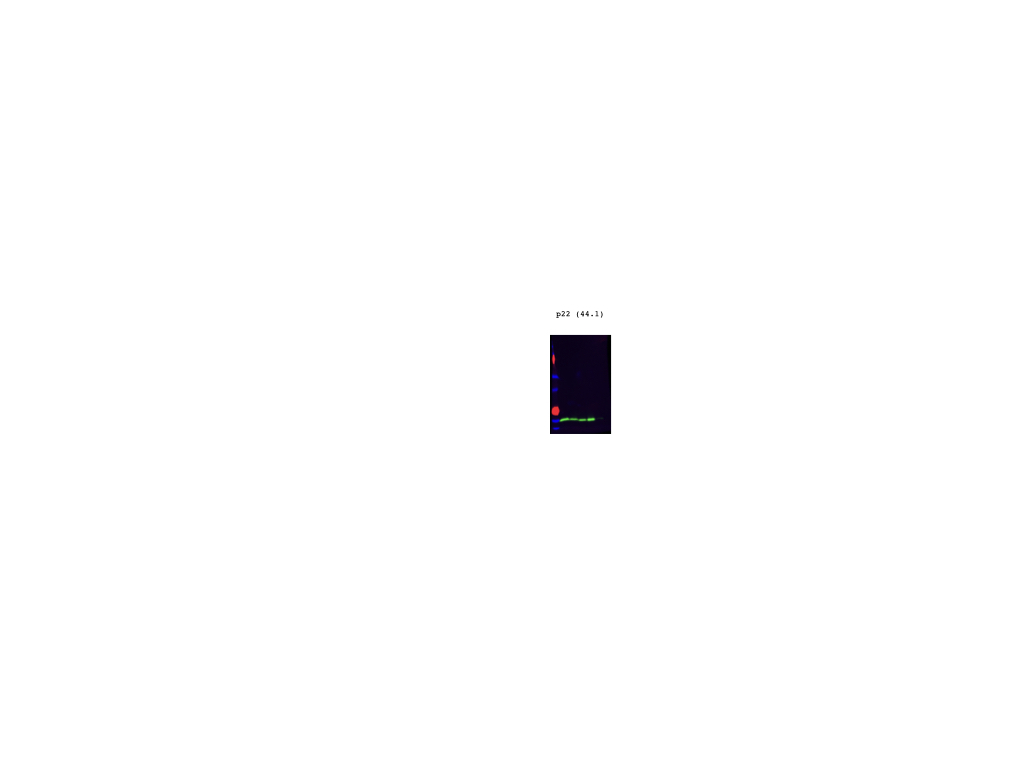

Supplement: Figure 2—source data 1. [file elife-76387-fig2-data1.zip › Figure 2- source data 1/Fig 2C p22.jpeg]

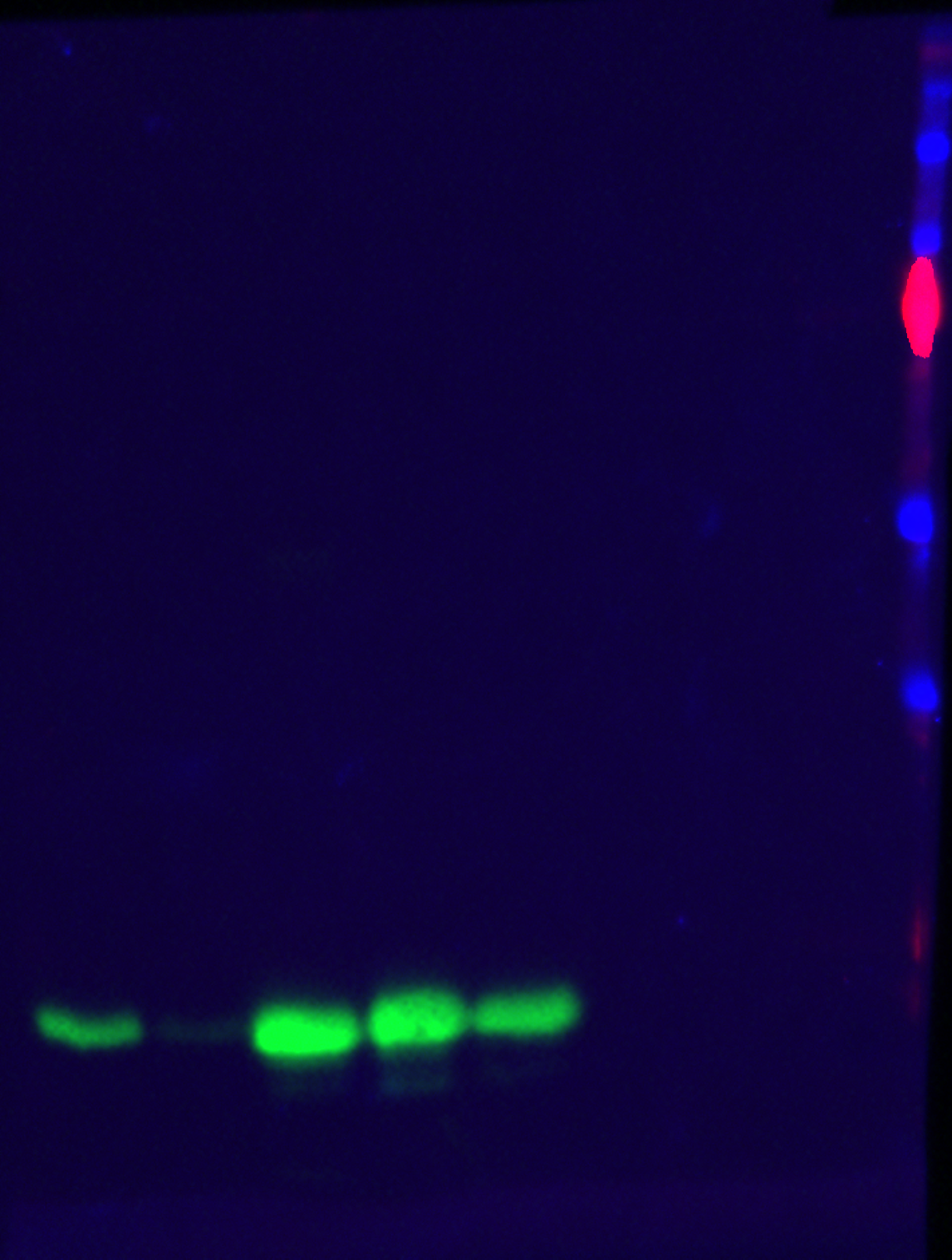

Supplement: Figure 2—source data 1. [file elife-76387-fig2-data1.zip › Figure 2- source data 1/Fig 2C_EROS.tiff]

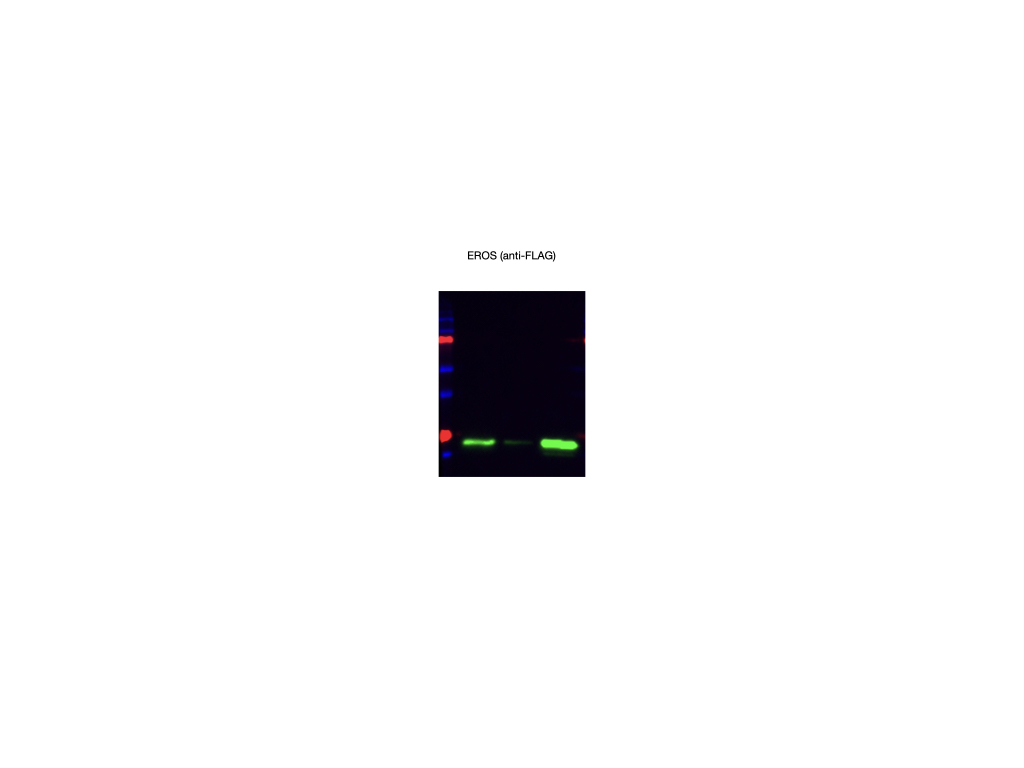

Supplement: Figure 2—source data 1. [file elife-76387-fig2-data1.zip › Figure 2- source data 1/Fig 2E EROS.jpeg]

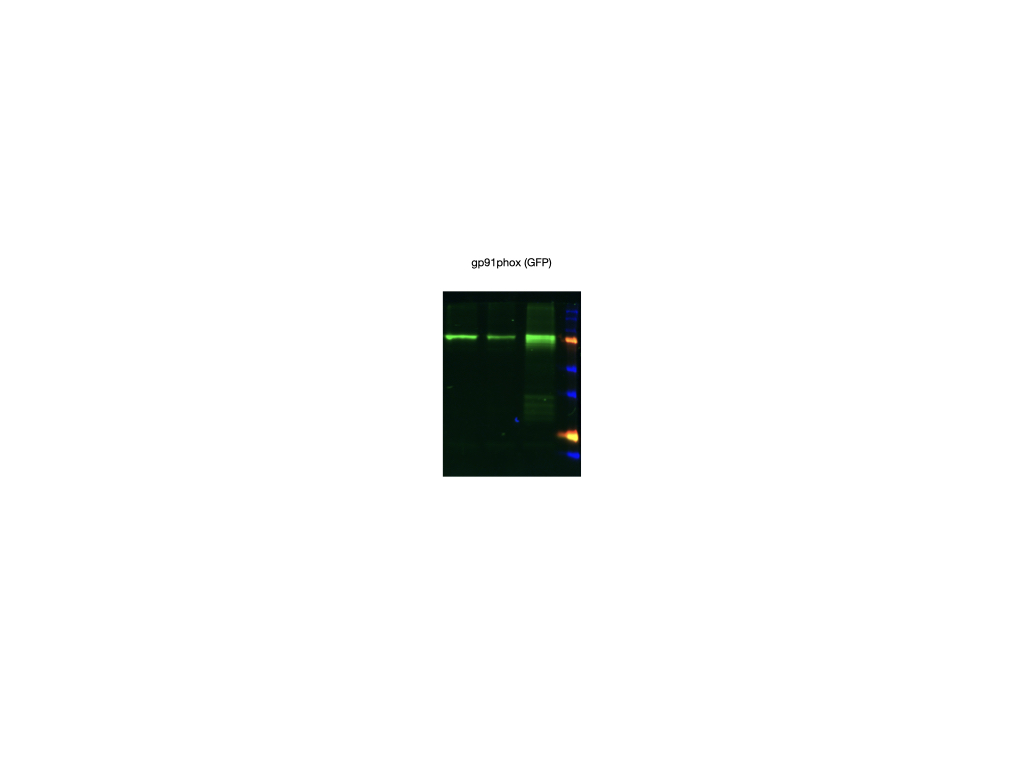

Supplement: Figure 2—source data 1. [file elife-76387-fig2-data1.zip › Figure 2- source data 1/Fig 2E gp91(GFP).jpeg]

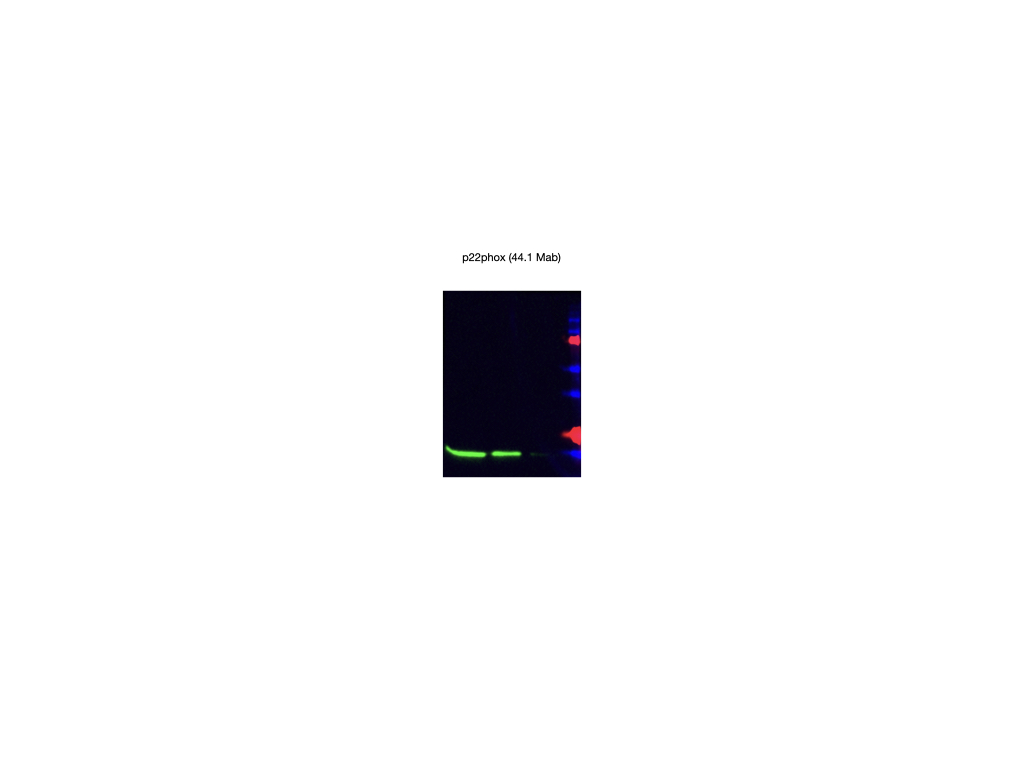

Supplement: Figure 2—source data 1. [file elife-76387-fig2-data1.zip › Figure 2- source data 1/Fig 2E p22.jpeg]

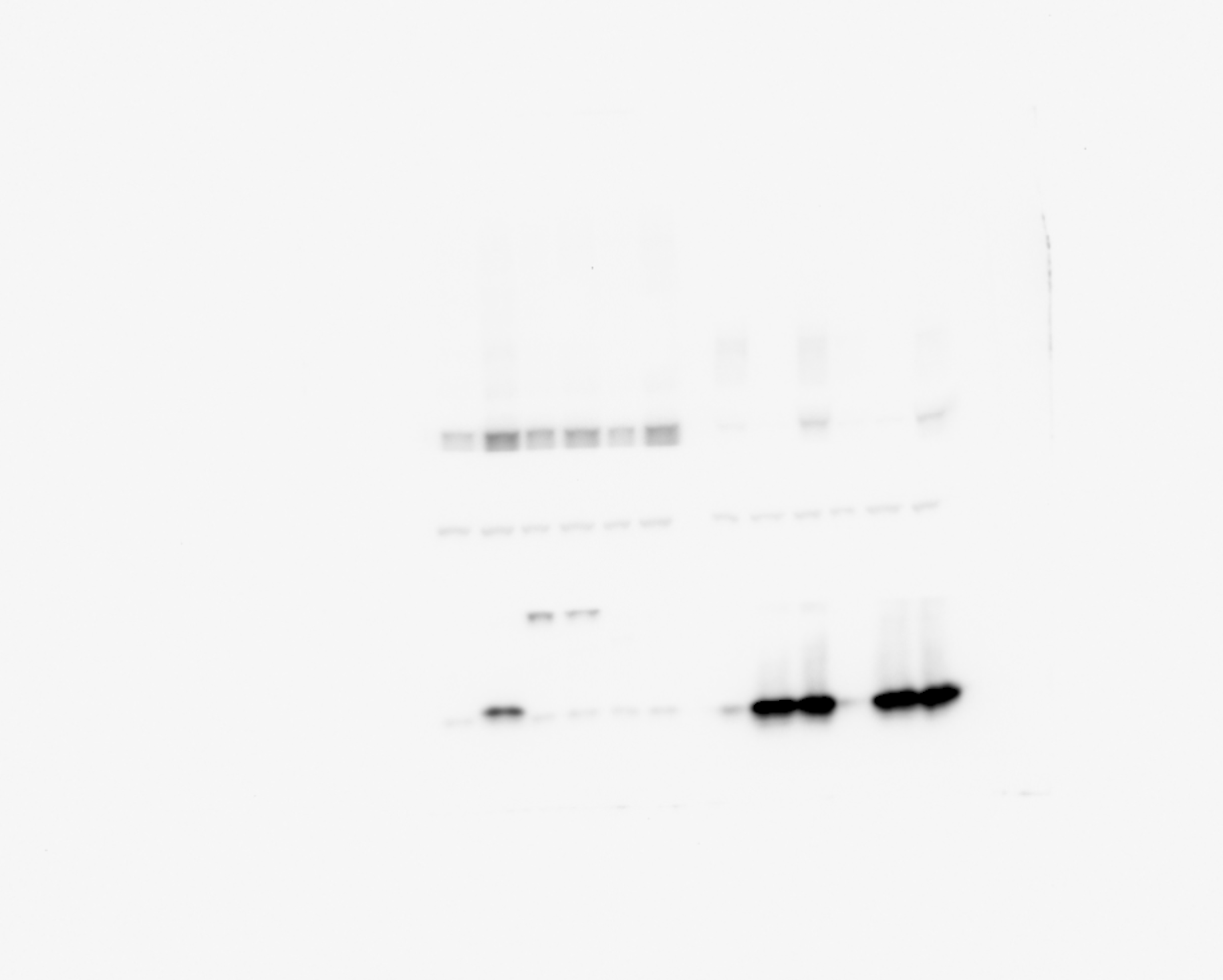

Supplement: Figure 2—figure supplement 1—source data 1. [file elife-76387-fig2-figsupp1-data1.zip › Figure 2 figure supplement 1- source data 1/2020-12-11 12h51m49s Chemiluminescence 10.000s HEK293 NOX2 SA Eros.tif]

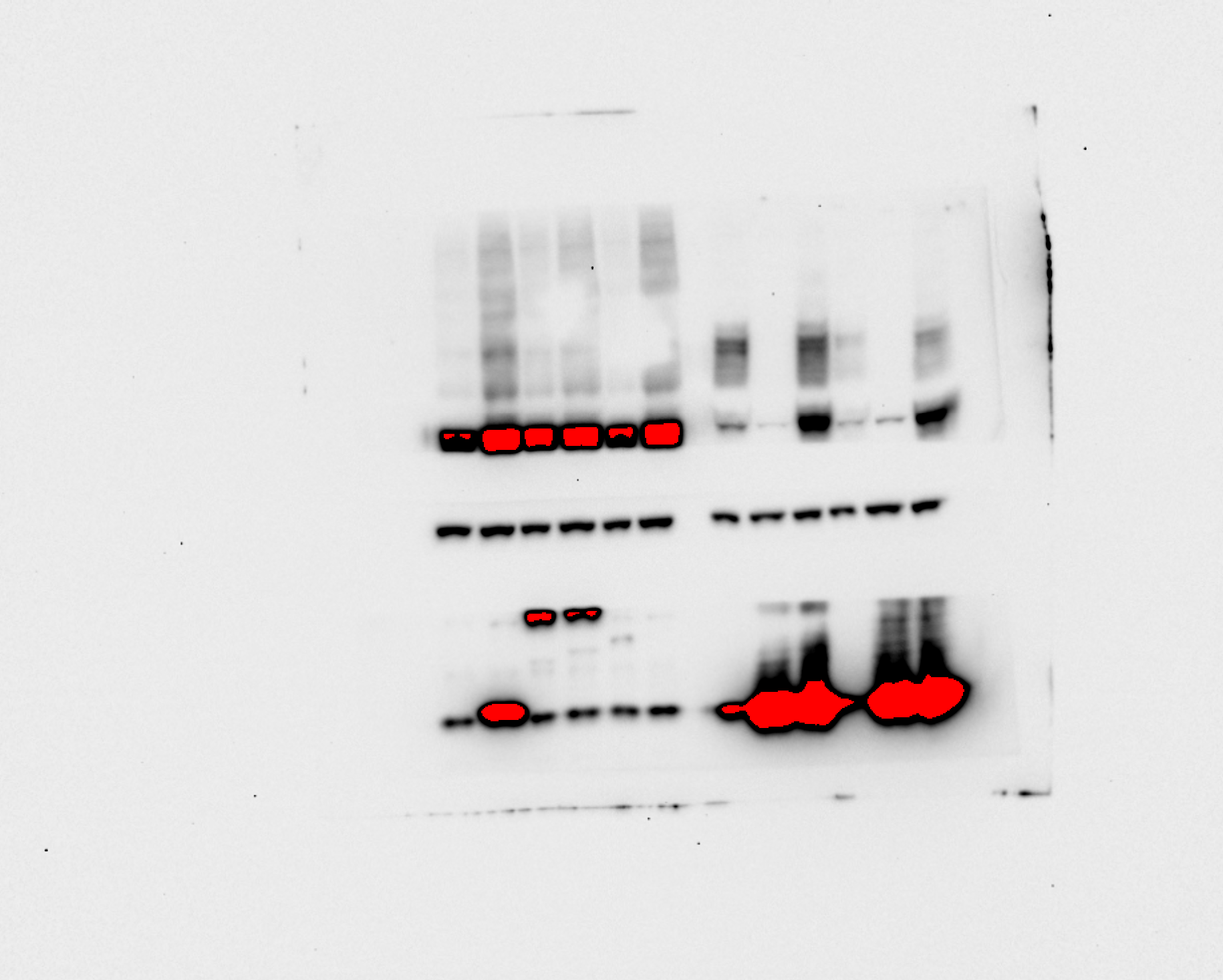

Supplement: Figure 2—figure supplement 1—source data 1. [file elife-76387-fig2-figsupp1-data1.zip › Figure 2 figure supplement 1- source data 1/2020-12-11 12h53m57s Chemiluminescence 133.448s HEK293 NOX2 SA gp91.tif]

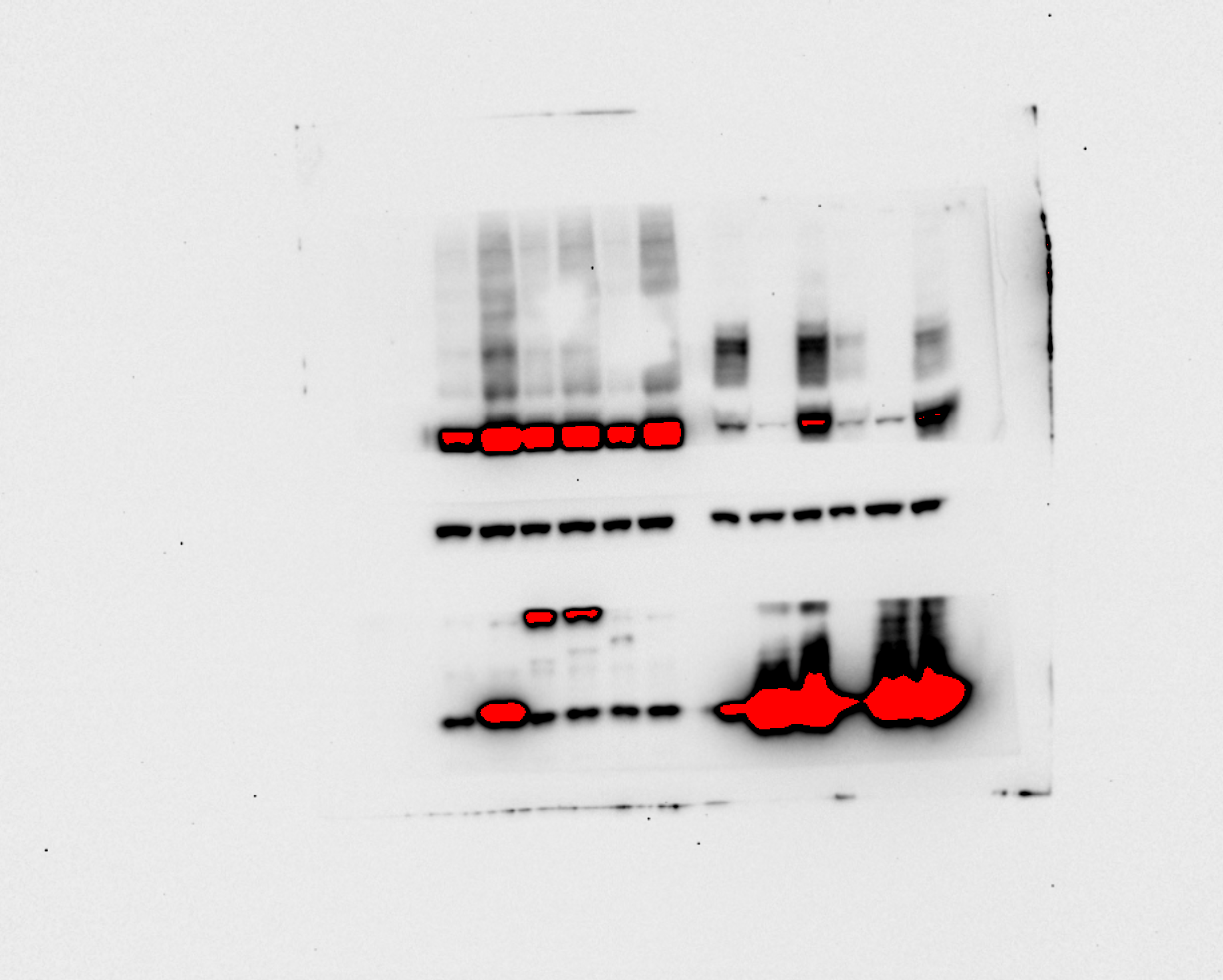

Supplement: Figure 2—figure supplement 1—source data 1. [file elife-76387-fig2-figsupp1-data1.zip › Figure 2 figure supplement 1- source data 1/2020-12-11 12h55m01s Chemiluminescence 195.172s HEK293 NOX2 SA actin.tif]

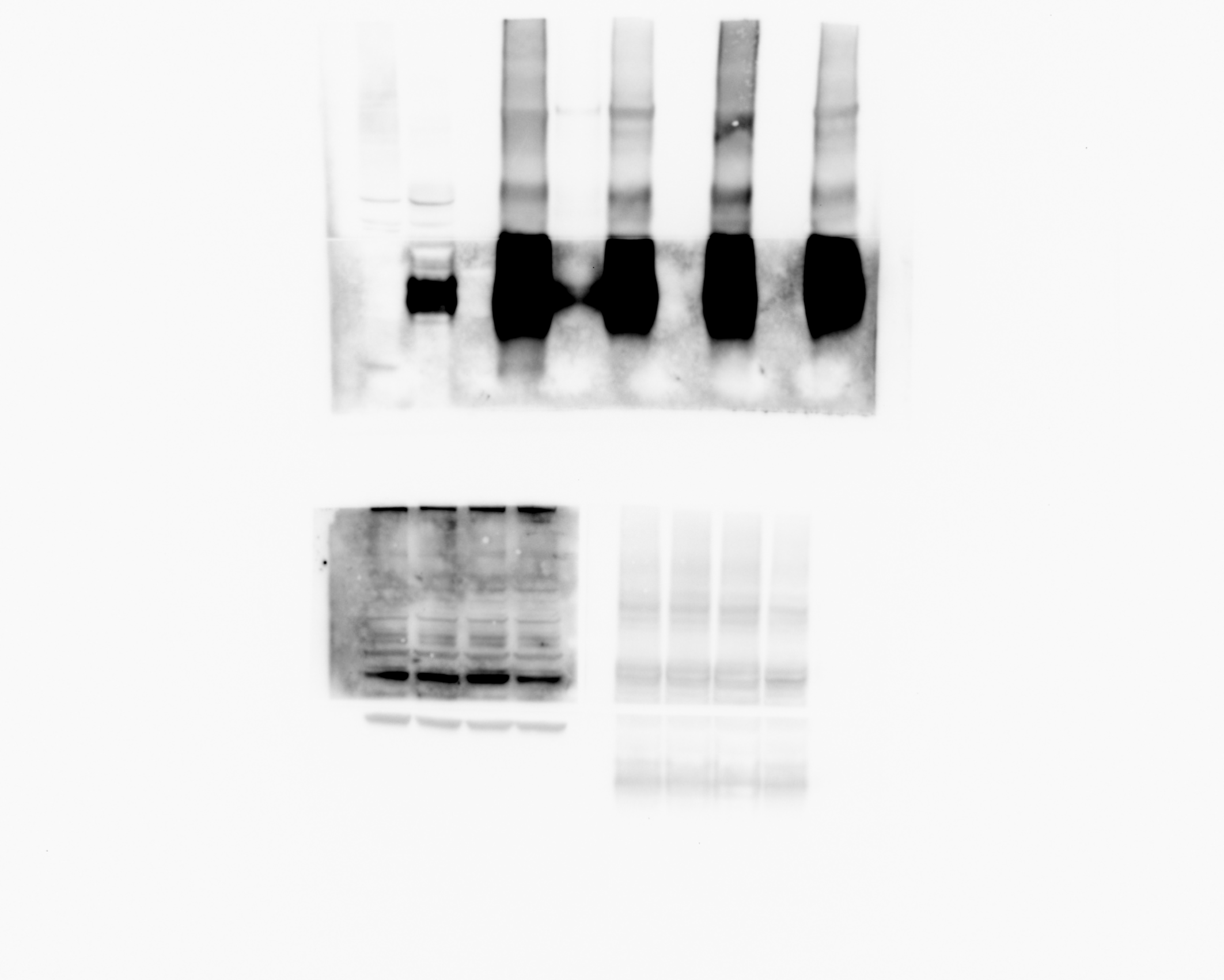

Supplement: Figure 2—figure supplement 1—source data 1. [file elife-76387-fig2-figsupp1-data1.zip › Figure 2 figure supplement 1- source data 1/2022-06-24 14h48m34s Chemiluminescence 30.000s IP PLB985 blot EROS.tif]

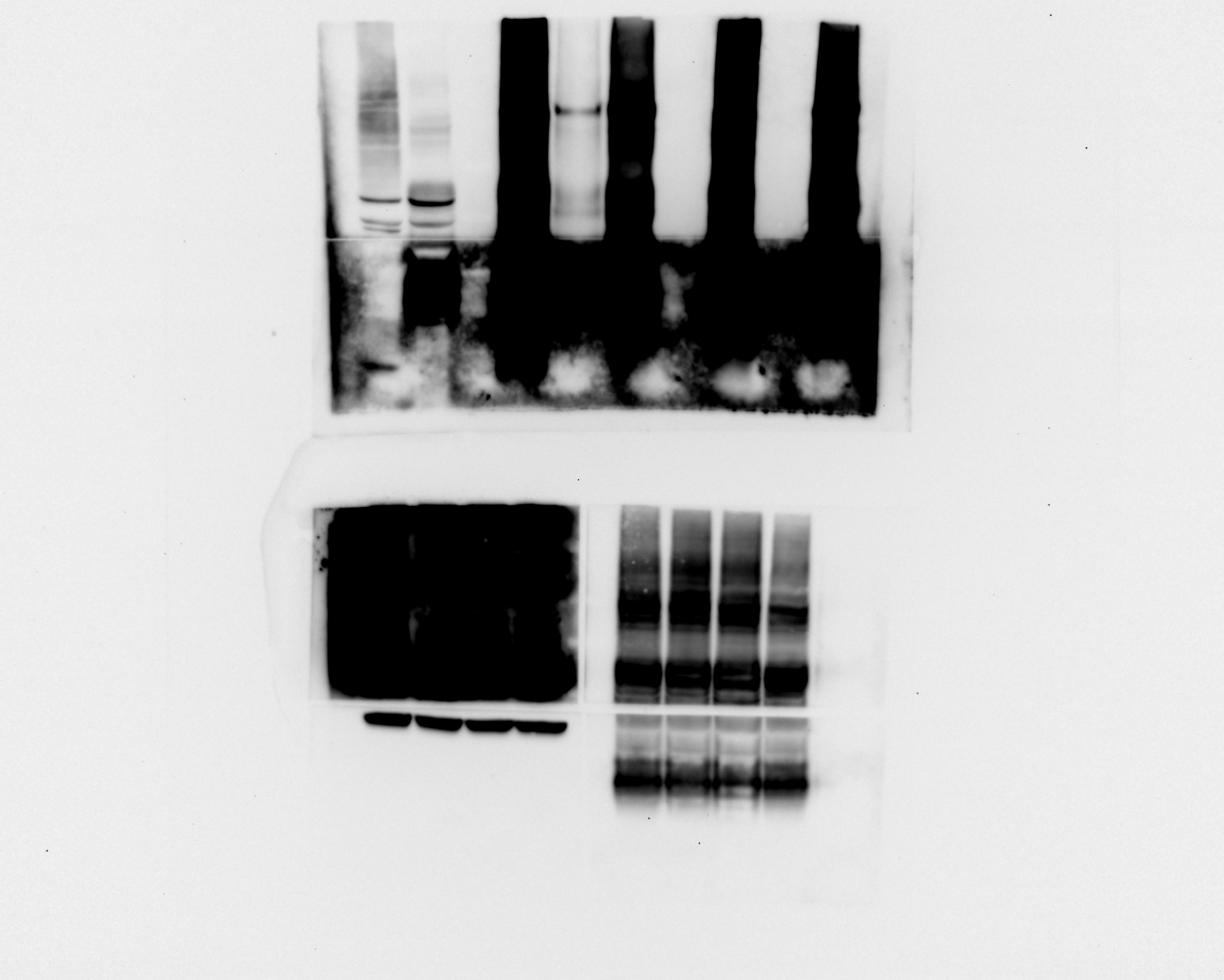

Supplement: Figure 2—figure supplement 1—source data 1. [file elife-76387-fig2-figsupp1-data1.zip › Figure 2 figure supplement 1- source data 1/2022-06-24 14h57m48s Chemiluminescence 360.000s IP PLB985 blot gp91phox.tif]

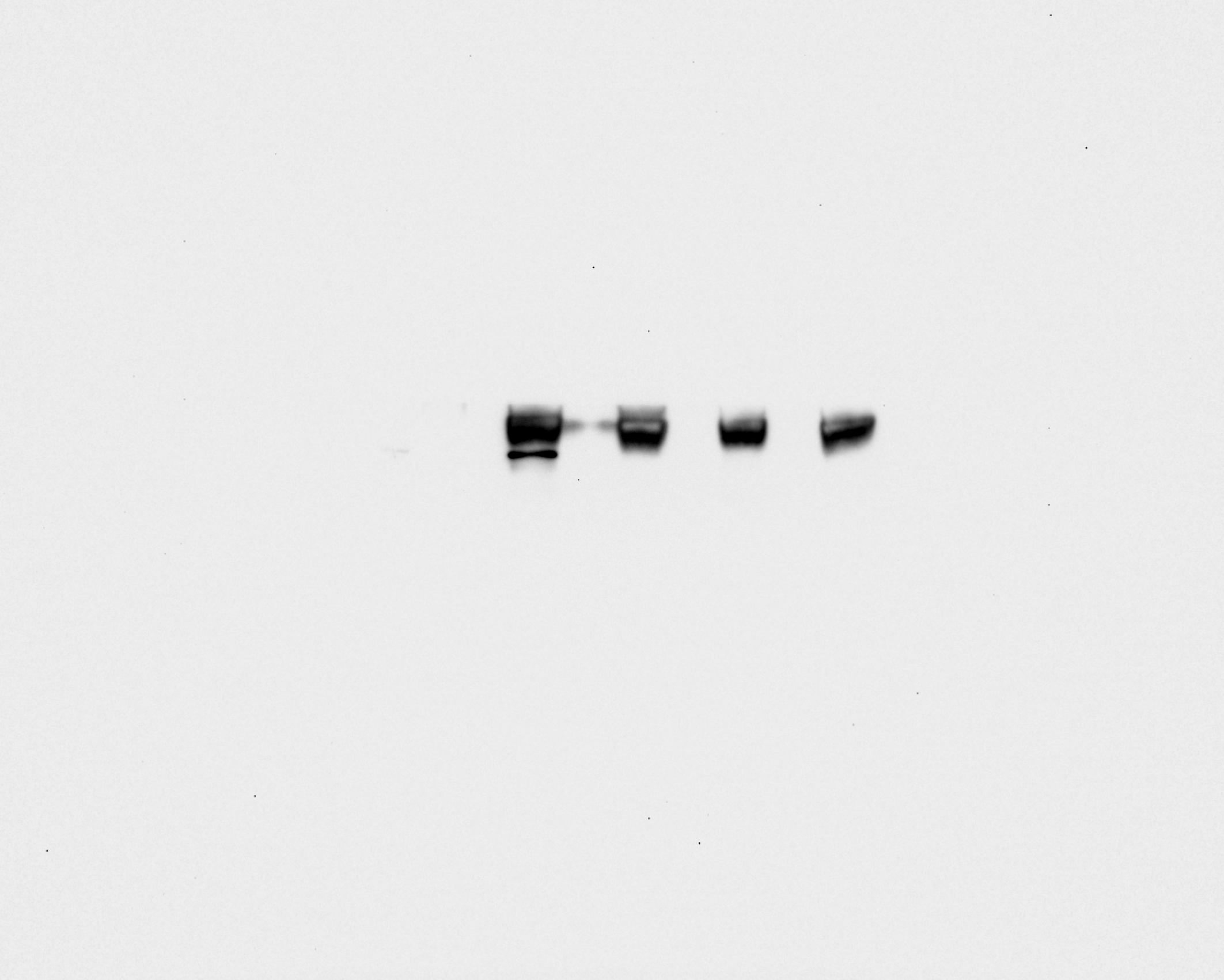

Supplement: Figure 2—figure supplement 1—source data 1. [file elife-76387-fig2-figsupp1-data1.zip › Figure 2 figure supplement 1- source data 1/2022-07-04 14h24m26s Chemiluminescence 204.346s IP PLB985 blot p22 IP.tif]

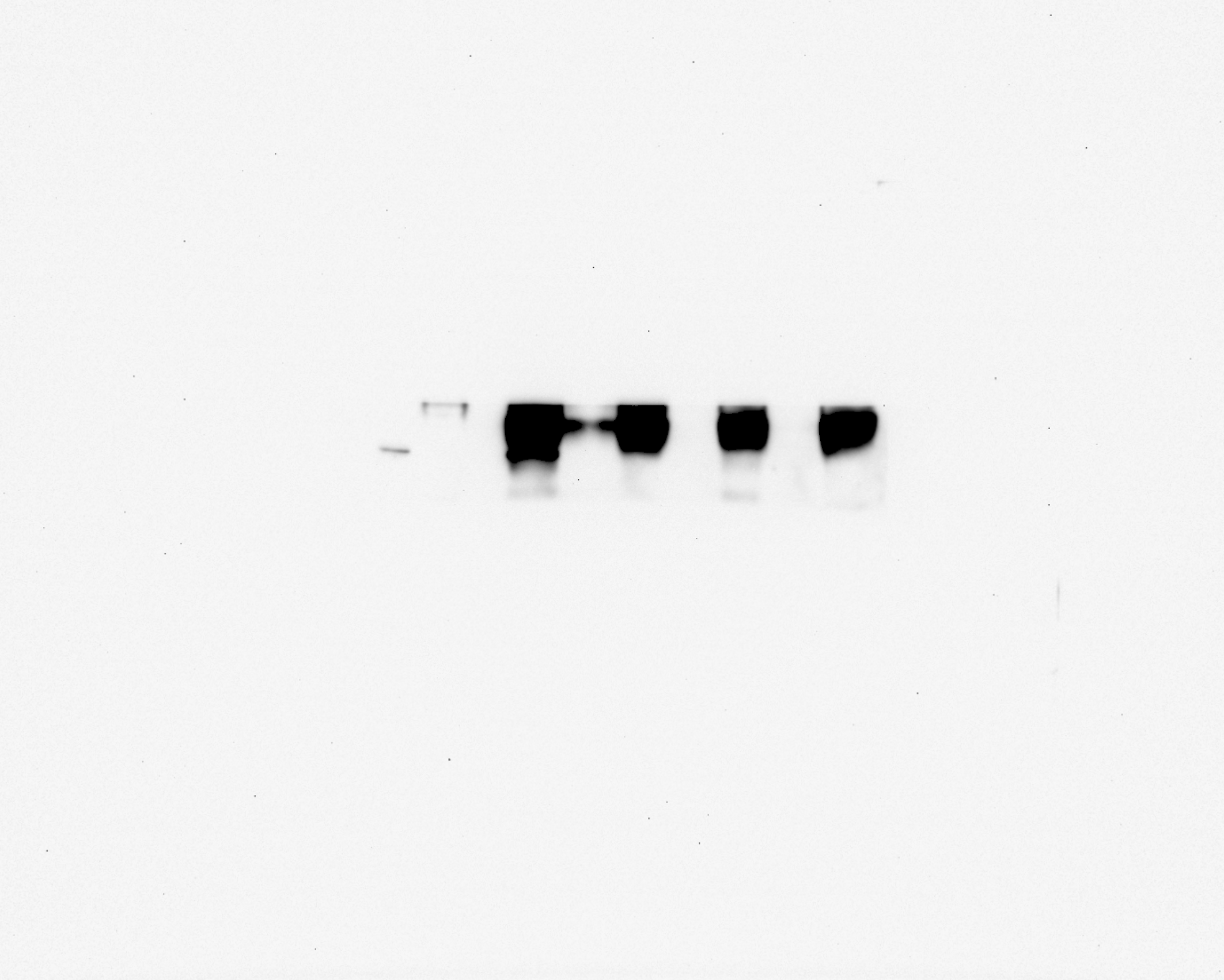

Supplement: Figure 2—figure supplement 1—source data 1. [file elife-76387-fig2-figsupp1-data1.zip › Figure 2 figure supplement 1- source data 1/2022-07-04 14h47m35s Chemiluminescence 1499.986s IP PLB985 blot p22 input.tif]

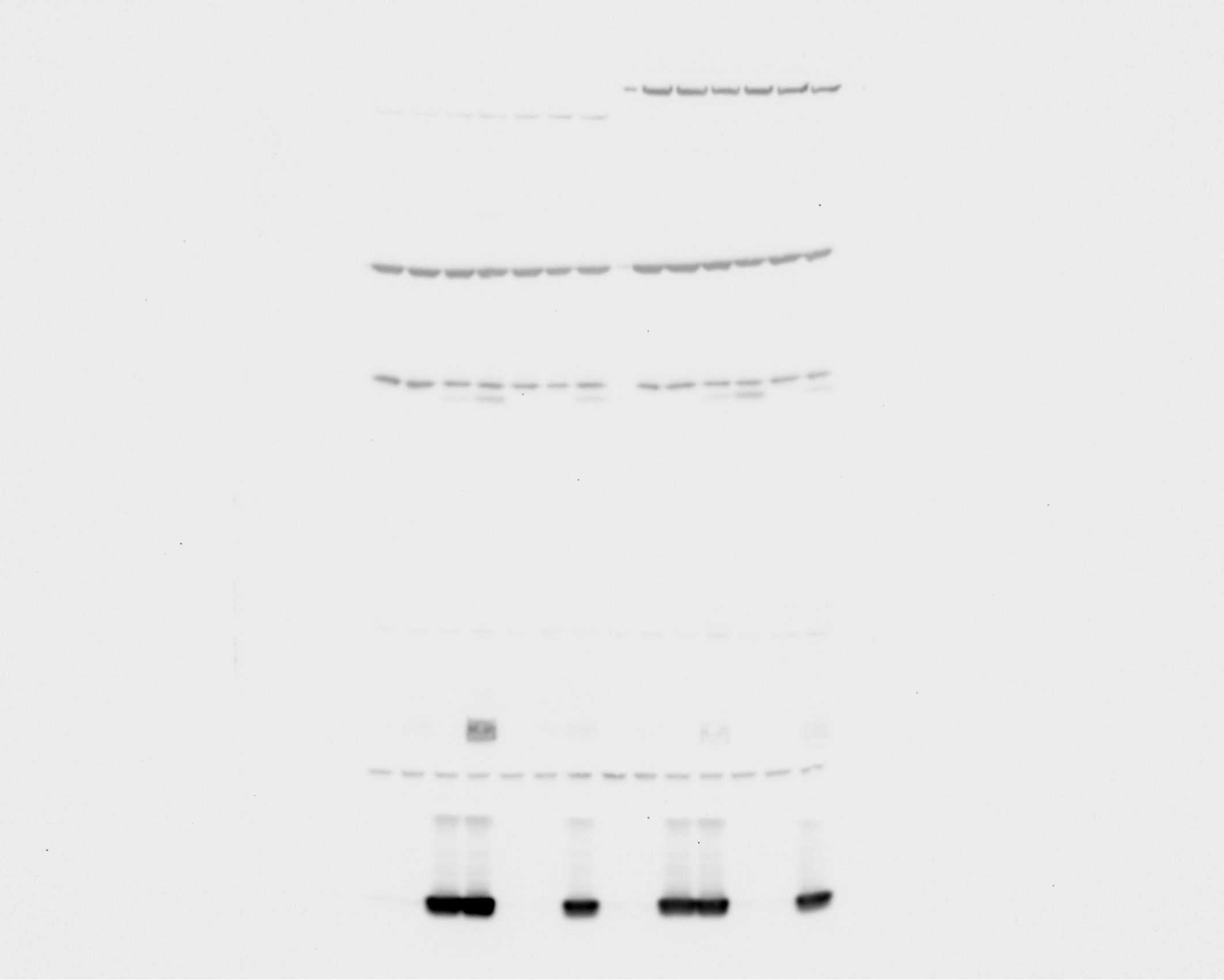

Supplement: Figure 2—figure supplement 1—source data 2. [file elife-76387-fig2-figsupp1-data2.zip › Figure 2 figure supplement 1- source data 2/2020-10-20 15h32m18s Chemiluminescence 80.689s HEK293 NOX4 SA eros.tif]

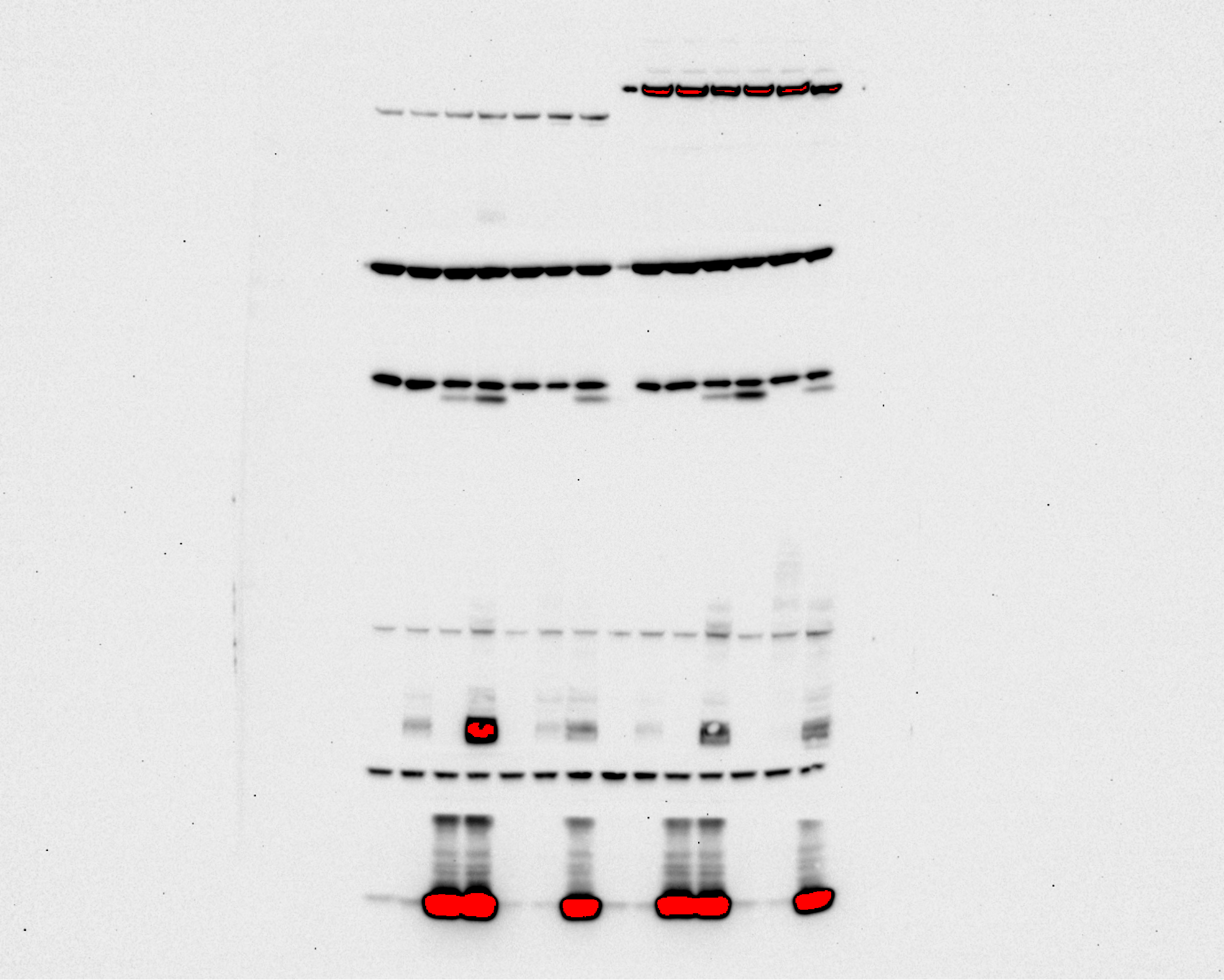

Supplement: Figure 2—figure supplement 1—source data 2. [file elife-76387-fig2-figsupp1-data2.zip › Figure 2 figure supplement 1- source data 2/2020-10-20 15h47m58s Chemiluminescence 942.402s HEK293 NOX4 SA actin.tif]

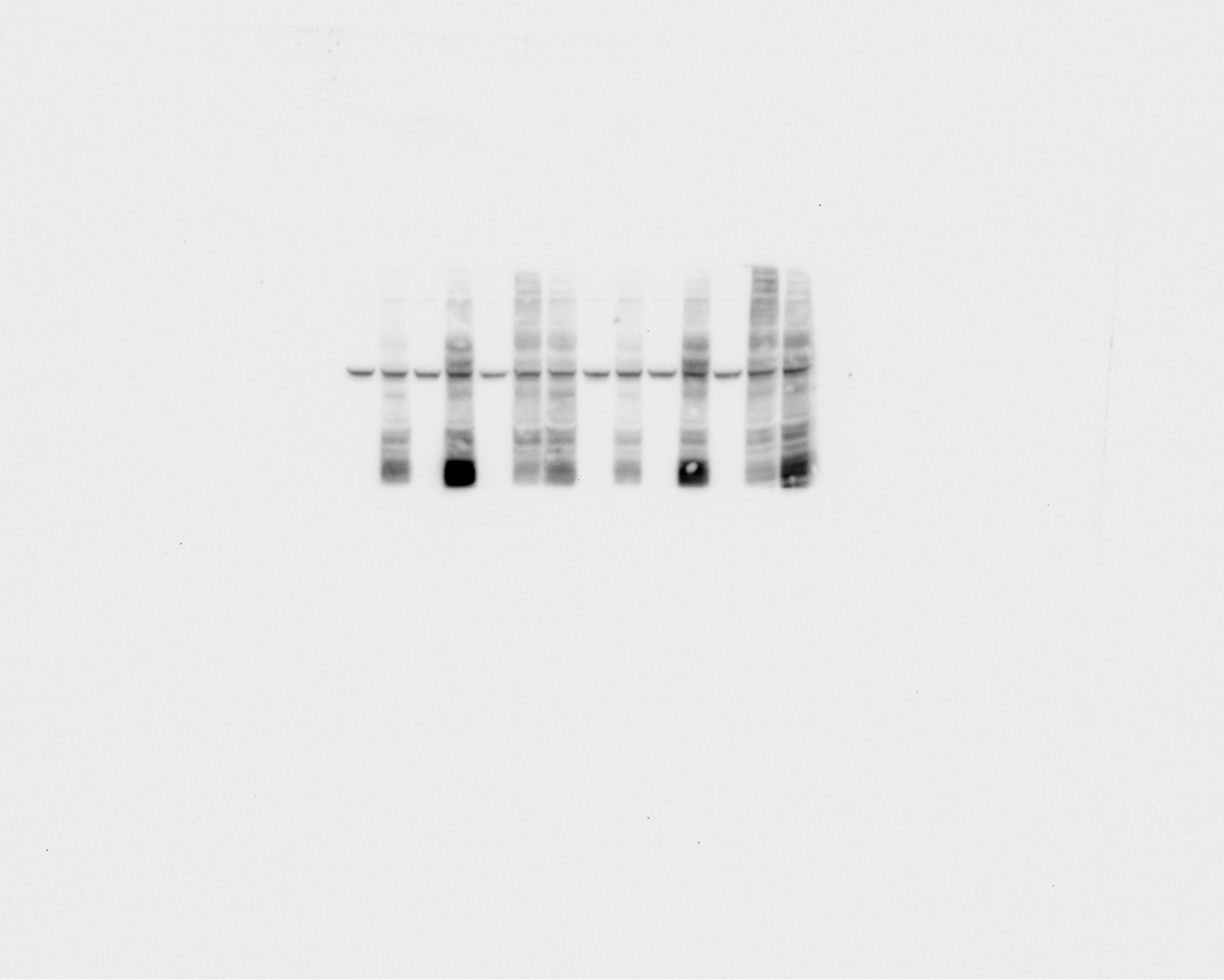

Supplement: Figure 2—figure supplement 1—source data 2. [file elife-76387-fig2-figsupp1-data2.zip › Figure 2 figure supplement 1- source data 2/2020-10-20 16h32m36s Chemiluminescence 59.583s HEK293 NOX4 SA gp91.tif]

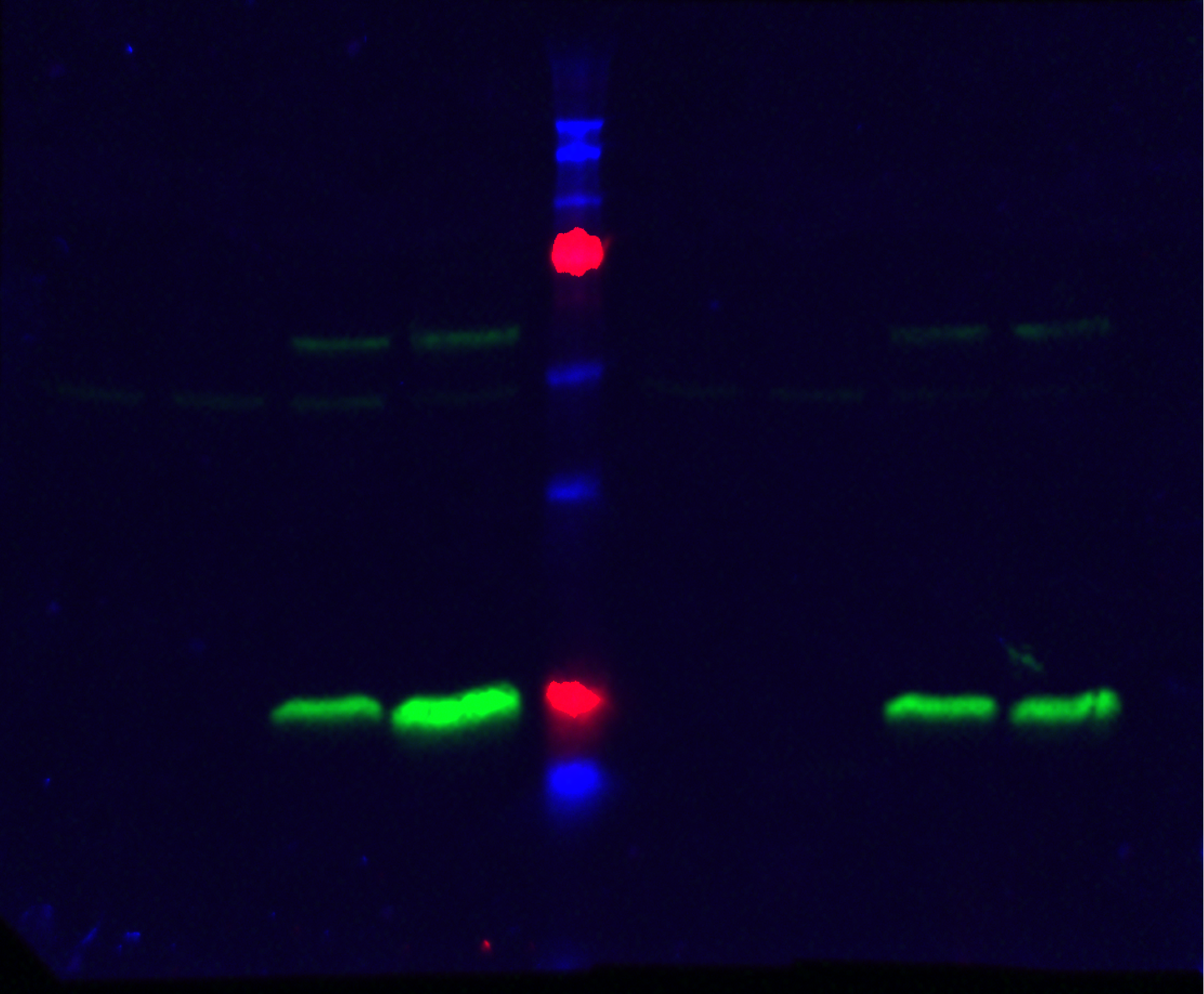

Supplement: Figure 2—figure supplement 1—source data 2. [file elife-76387-fig2-figsupp1-data2.zip › Figure 2 figure supplement 1- source data 2/SI-Fig2D EROS.tif]

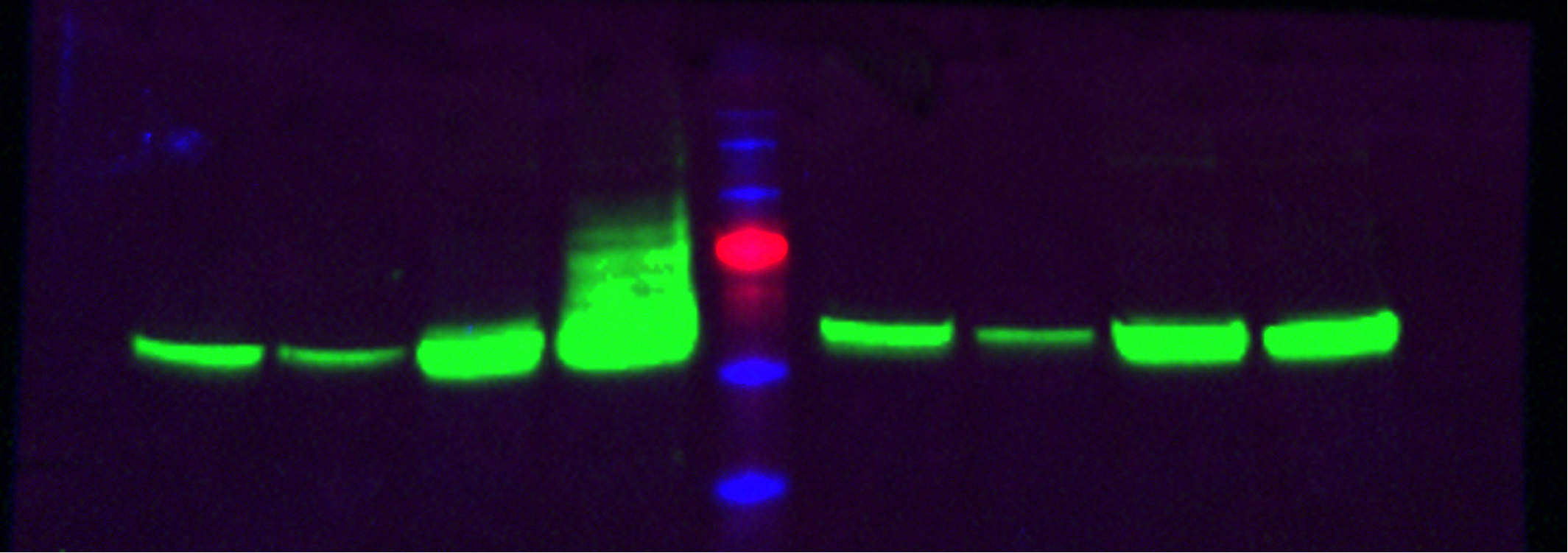

Supplement: Figure 2—figure supplement 1—source data 2. [file elife-76387-fig2-figsupp1-data2.zip › Figure 2 figure supplement 1- source data 2/SI-Fig2D NOX1 (anti-FLAG).tif]

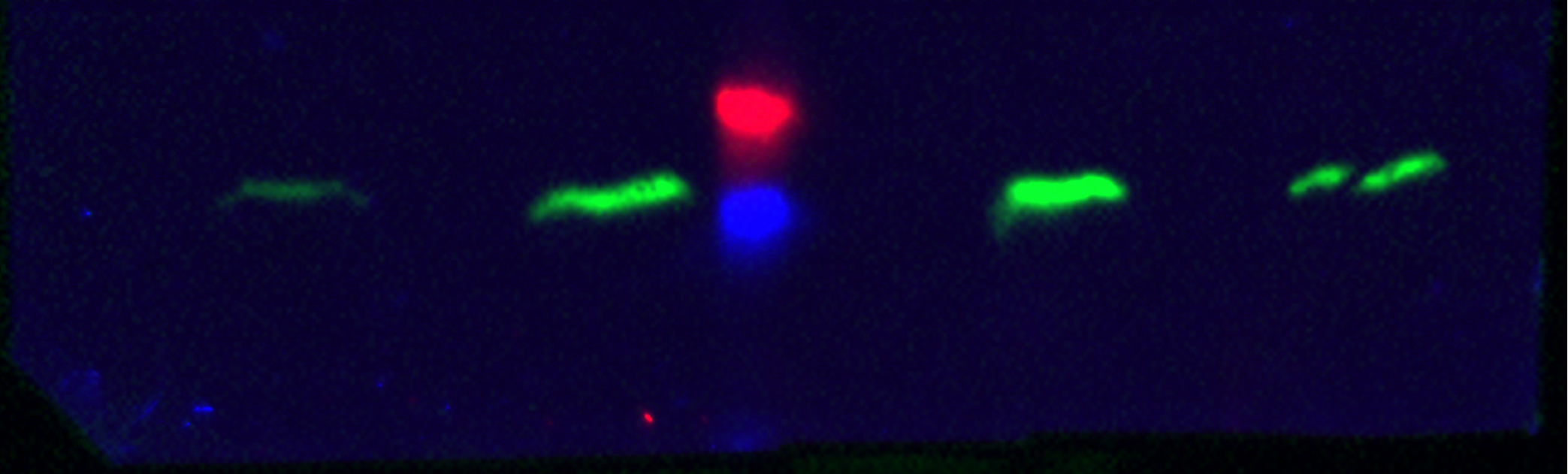

Supplement: Figure 2—figure supplement 1—source data 2. [file elife-76387-fig2-figsupp1-data2.zip › Figure 2 figure supplement 1- source data 2/SI-Fig2D p22.tif]

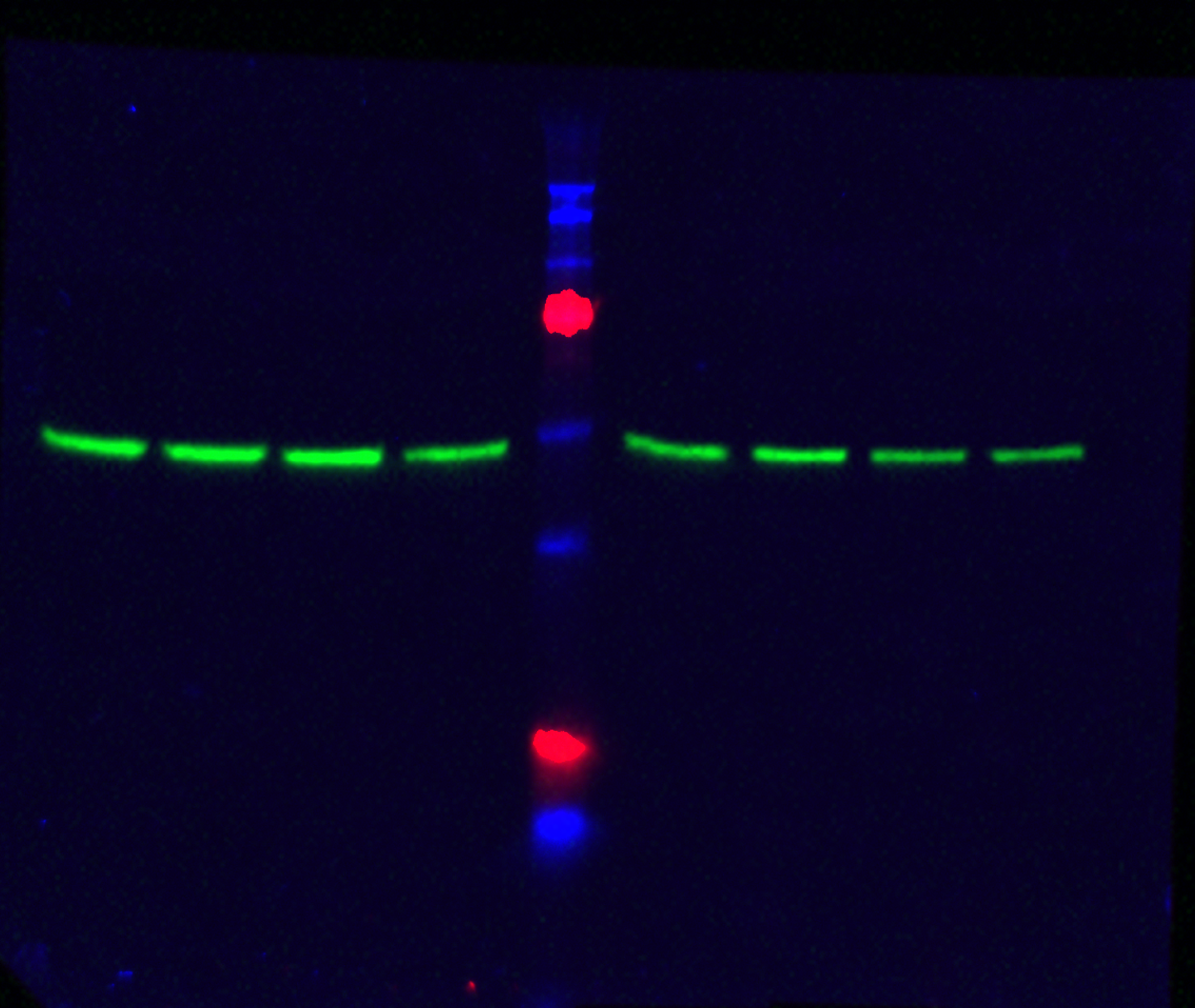

Supplement: Figure 2—figure supplement 1—source data 2. [file elife-76387-fig2-figsupp1-data2.zip › Figure 2 figure supplement 1- source data 2/SI-Fig2D tubulin.tif]

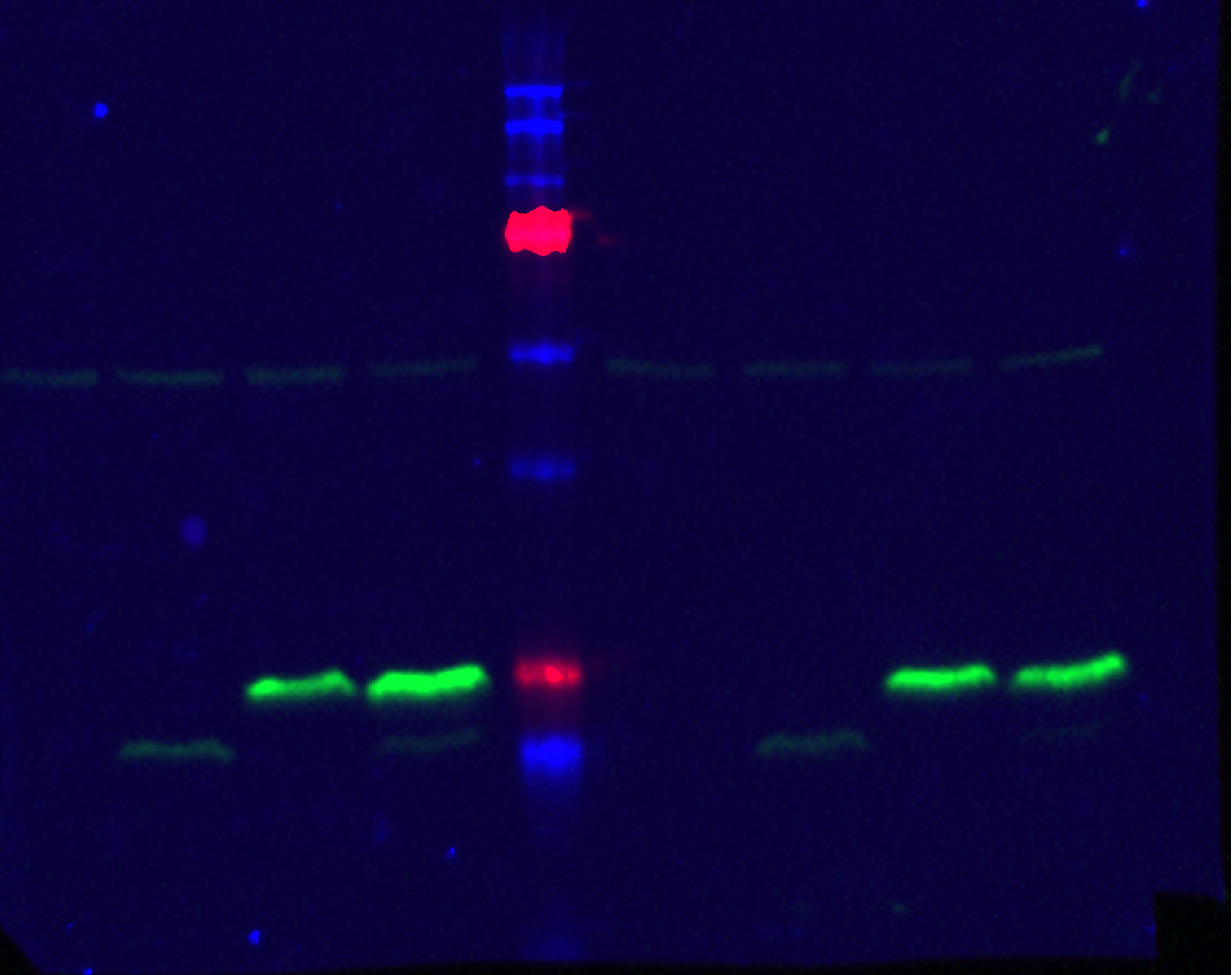

Supplement: Figure 2—figure supplement 1—source data 2. [file elife-76387-fig2-figsupp1-data2.zip › Figure 2 figure supplement 1- source data 2/SI-Fig2F EROS.tif]

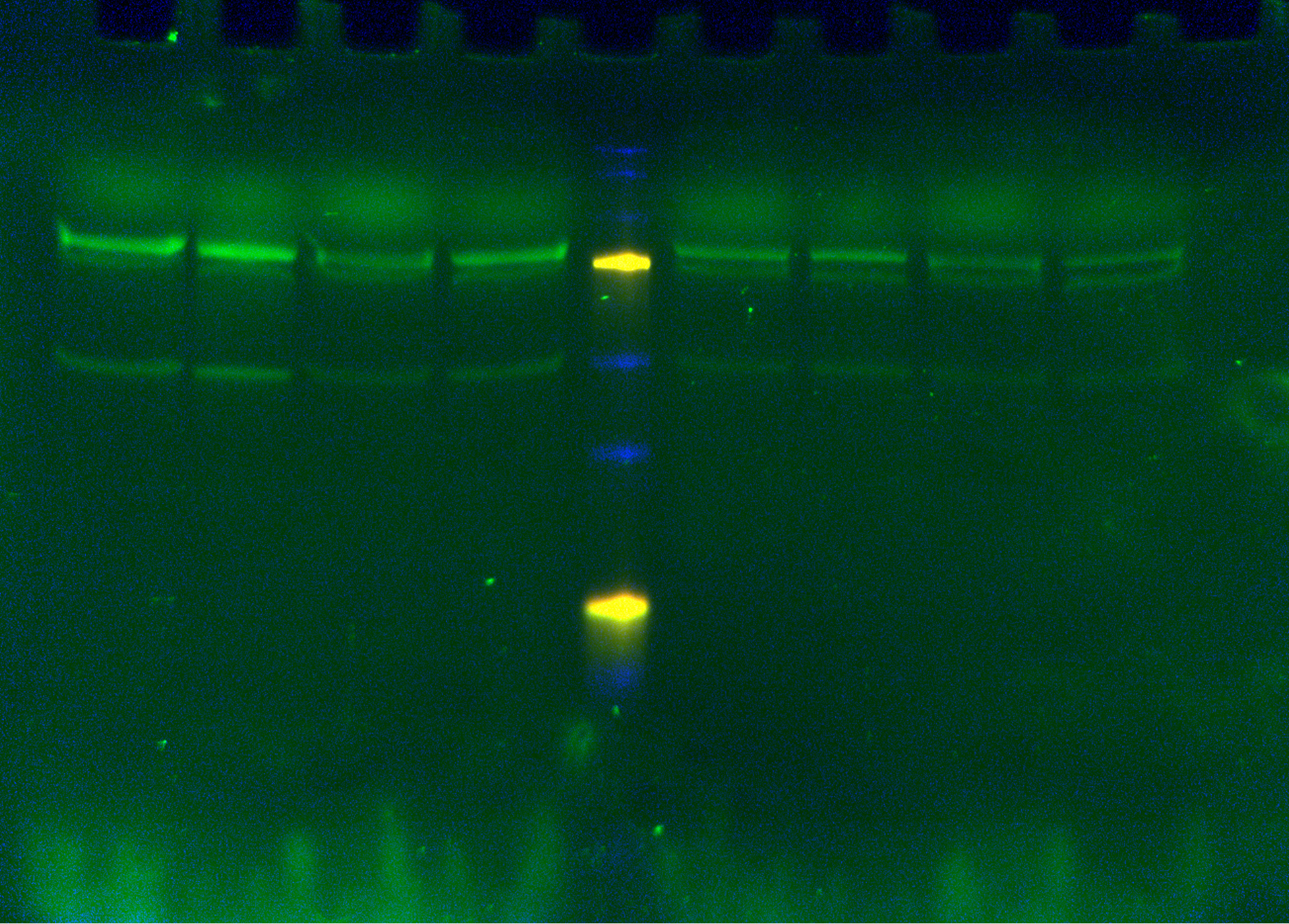

Supplement: Figure 2—figure supplement 1—source data 2. [file elife-76387-fig2-figsupp1-data2.zip › Figure 2 figure supplement 1- source data 2/SI-Fig2F NOX5(GFP).tif]

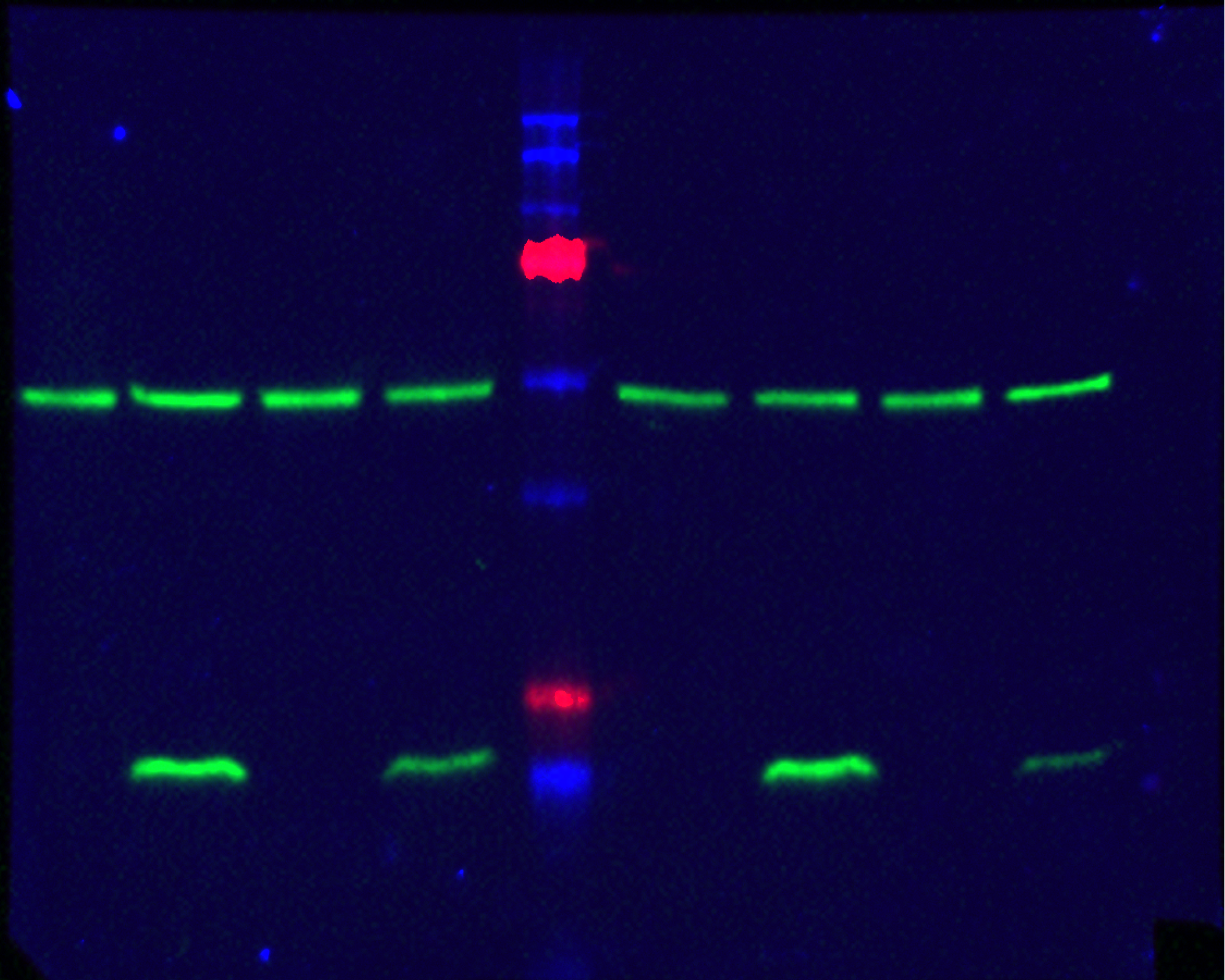

Supplement: Figure 2—figure supplement 1—source data 2. [file elife-76387-fig2-figsupp1-data2.zip › Figure 2 figure supplement 1- source data 2/SI-Fig2F p22.tif]

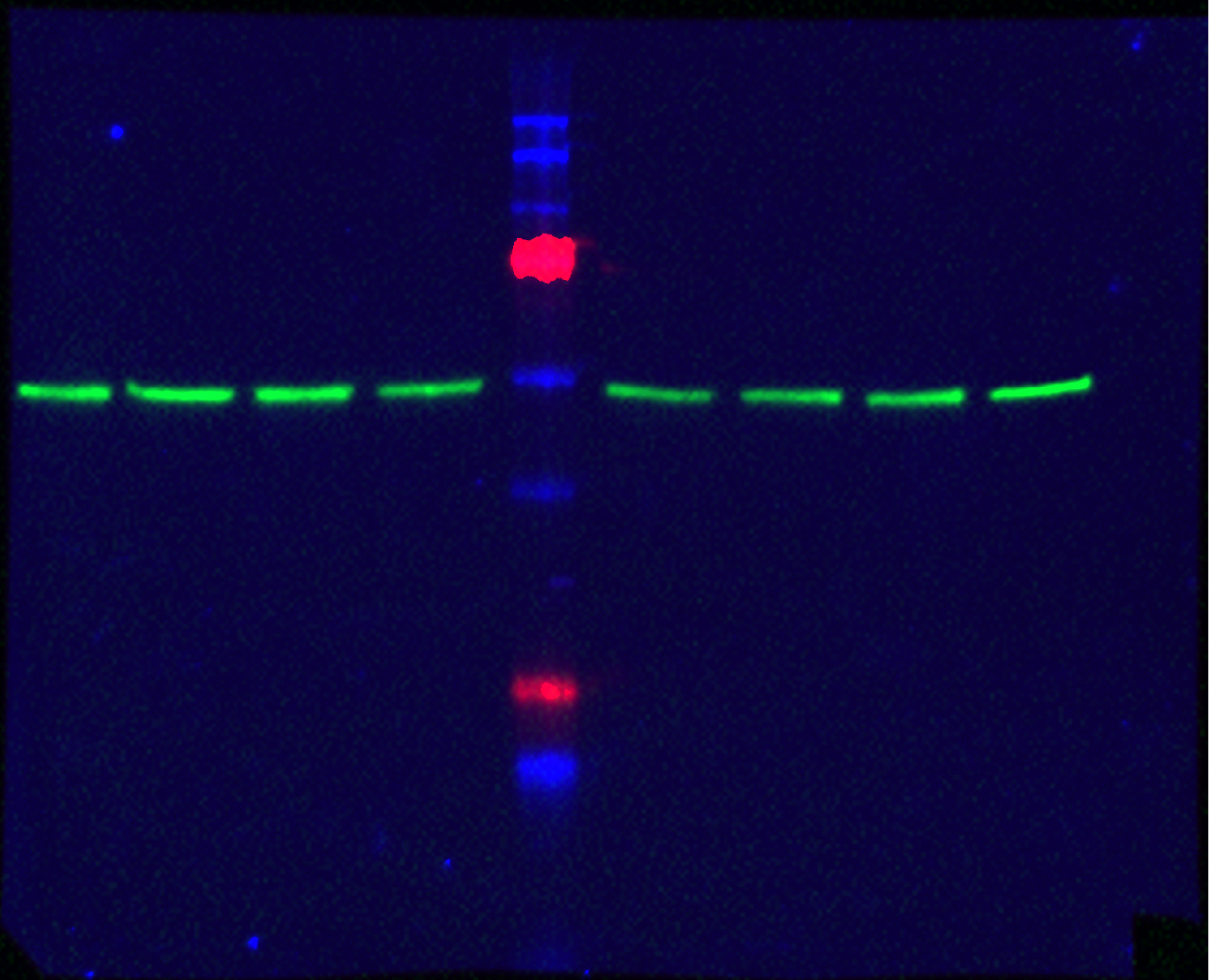

Supplement: Figure 2—figure supplement 1—source data 2. [file elife-76387-fig2-figsupp1-data2.zip › Figure 2 figure supplement 1- source data 2/SI-Fig2F tubulin.tif]

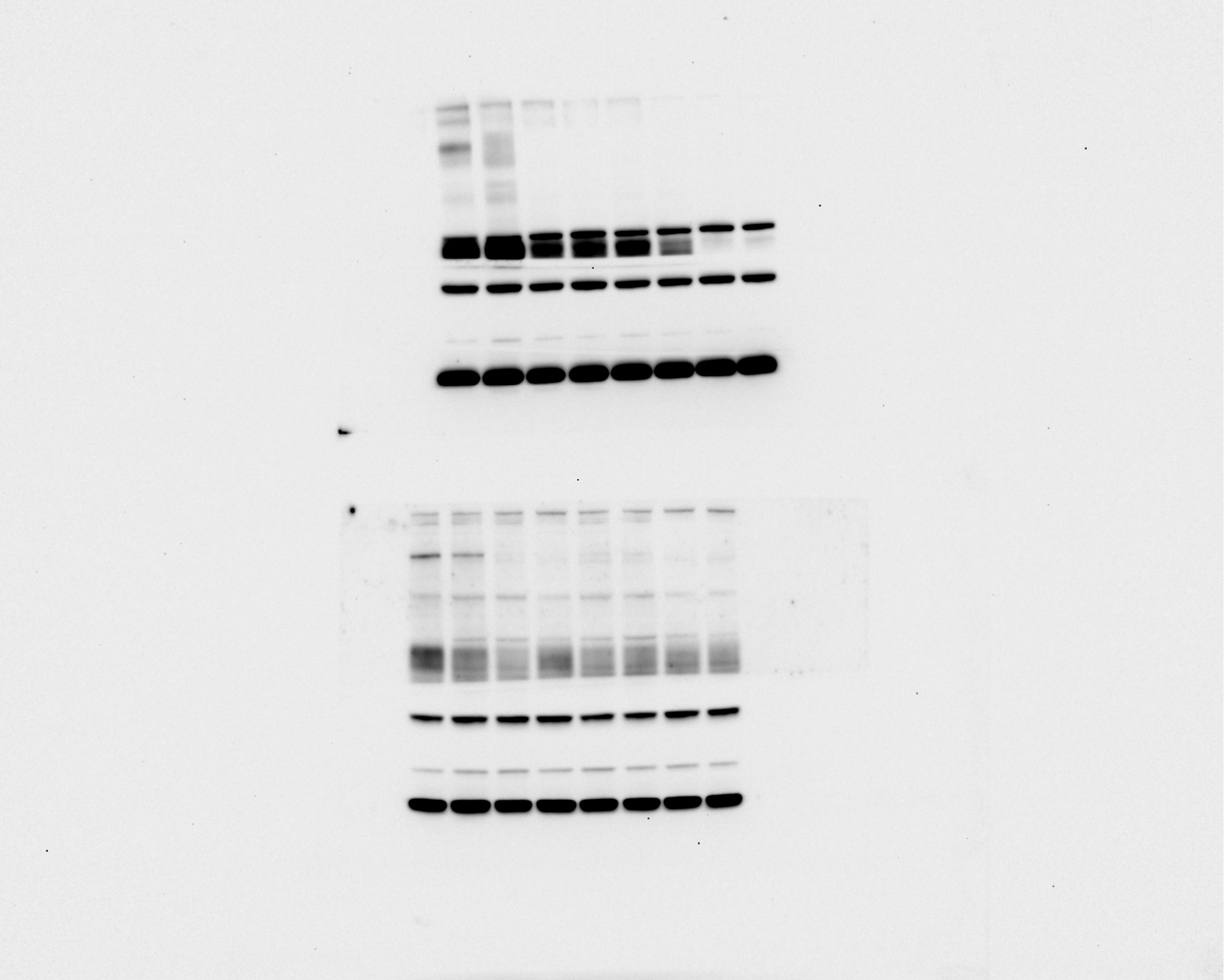

Supplement: Figure 2—figure supplement 1—source data 3. [file elife-76387-fig2-figsupp1-data3.zip › Figure 2 figure supplement 1- source data 3/2022-07-13 14h15m48s Chemiluminescence 1188.529s Kidney EROS KO, actin.tif]

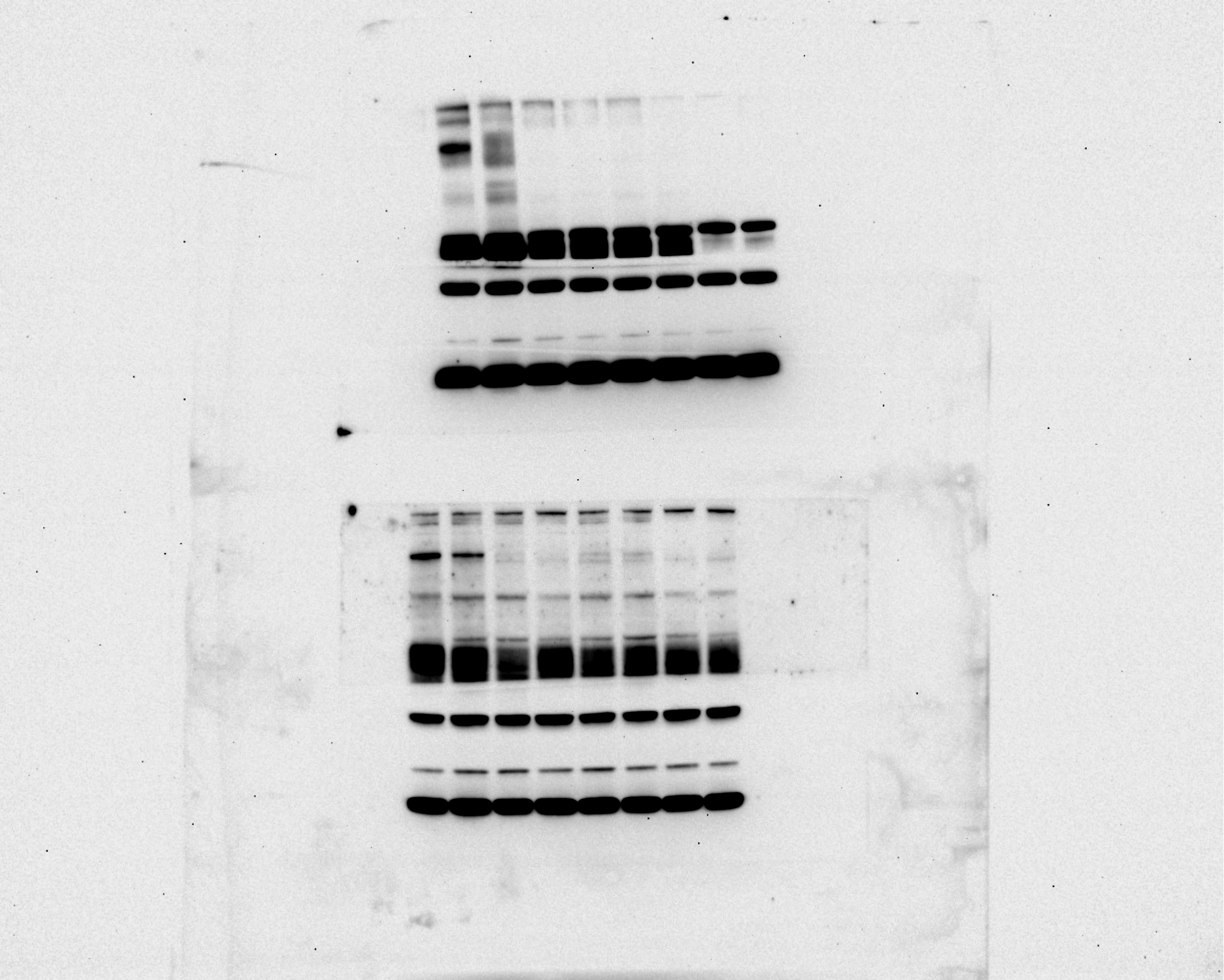

Supplement: Figure 2—figure supplement 1—source data 3. [file elife-76387-fig2-figsupp1-data3.zip › Figure 2 figure supplement 1- source data 3/2022-07-13 14h15m48s Chemiluminescence 1188.529s Kidney EROS KO, EROS.tif]

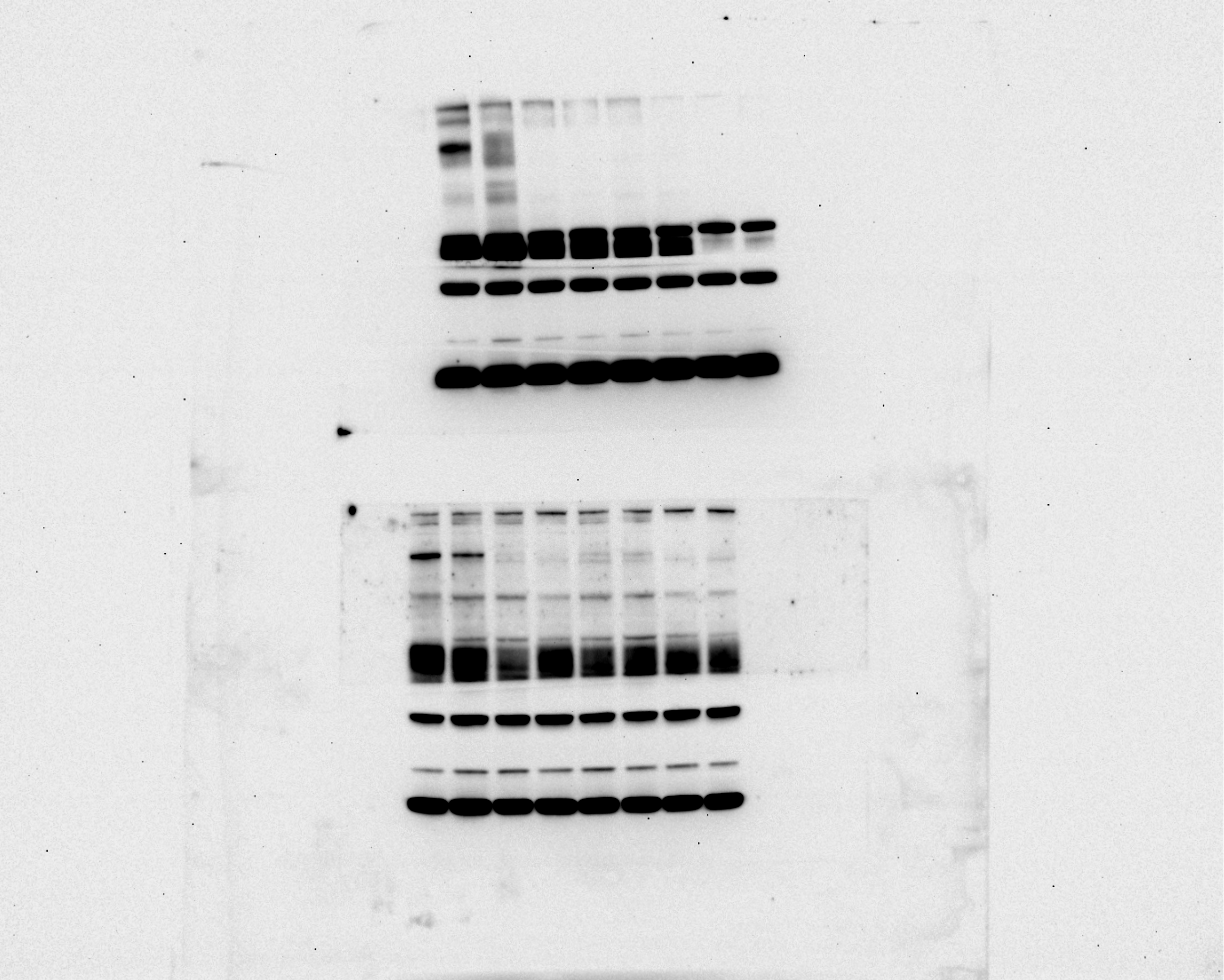

Supplement: Figure 2—figure supplement 1—source data 3. [file elife-76387-fig2-figsupp1-data3.zip › Figure 2 figure supplement 1- source data 3/2022-07-13 14h15m48s Chemiluminescence 1188.529s Kidney EROS KO, NOX4.tif]

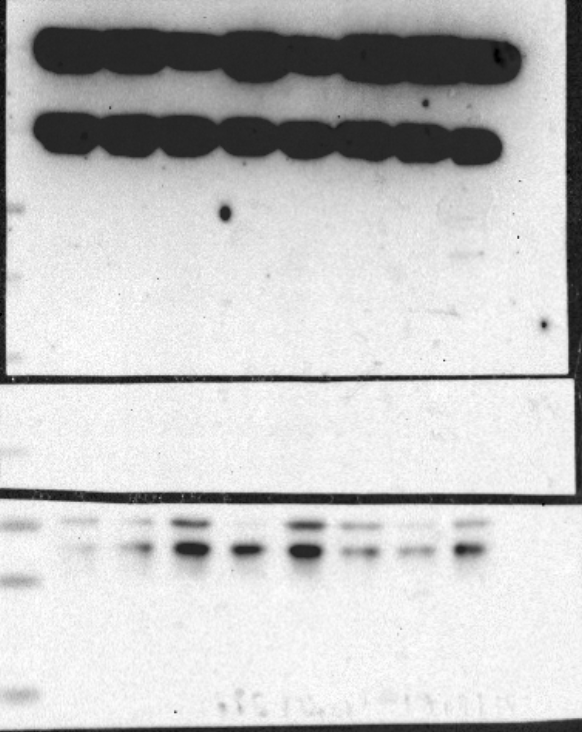

Supplement: Figure 2—figure supplement 1—source data 3. [file elife-76387-fig2-figsupp1-data3.zip › Figure 2 figure supplement 1- source data 3/2022-08-18 13h15m42s Colorimetric 0.030s+2022-08-18 13h58m39s Chemiluminescence 2249.976s Heart EROS.tif]

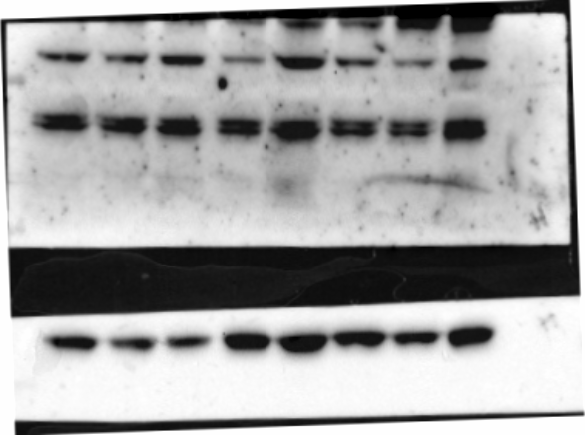

Supplement: Figure 2—figure supplement 1—source data 3. [file elife-76387-fig2-figsupp1-data3.zip › Figure 2 figure supplement 1- source data 3/2022-08-19 12h00m17s Chemiluminescence 216.426s+2022-08-19 11h56m00s Colorimetric 0.027s Heart NOX4 actin.tif]

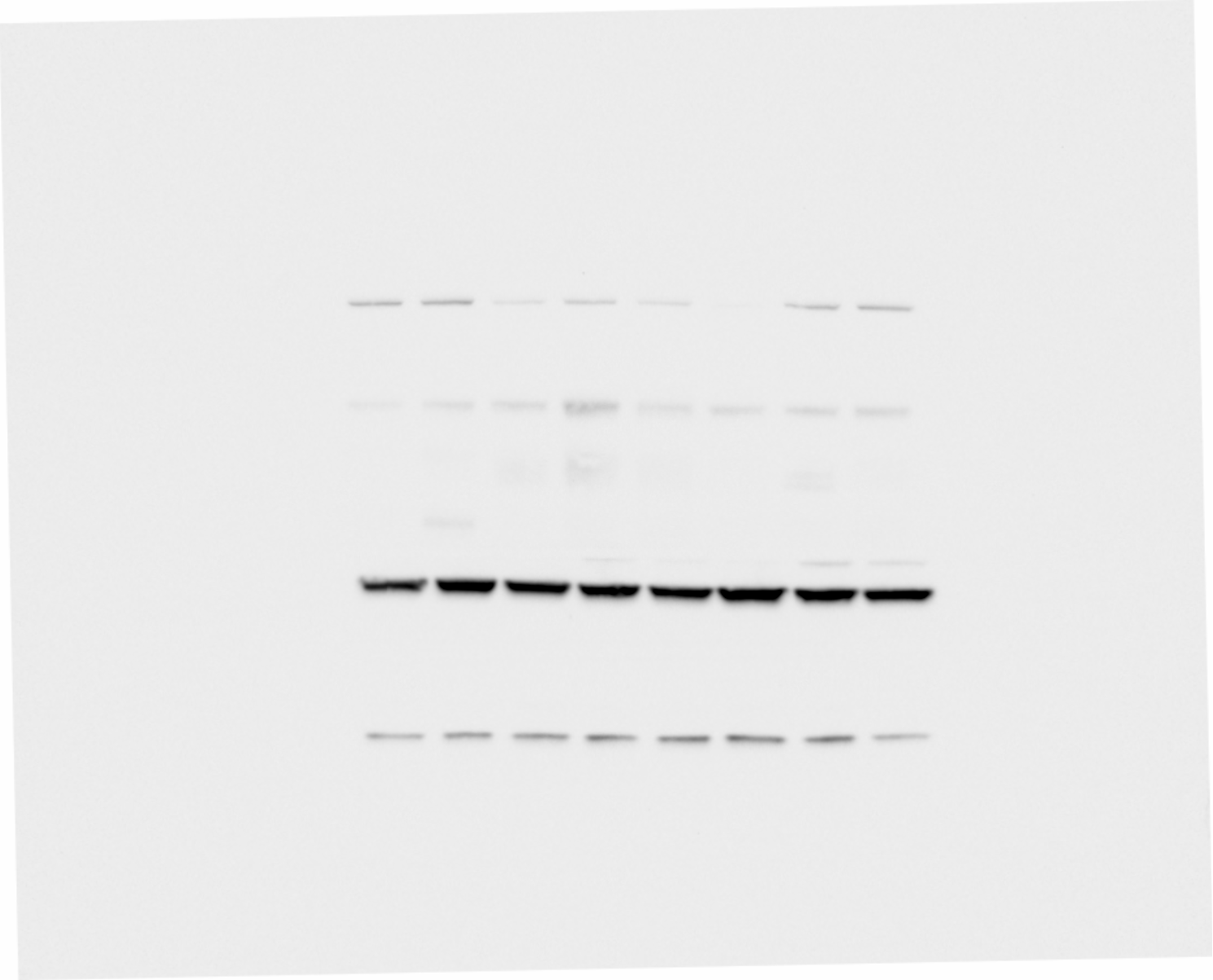

Supplement: Figure 2—figure supplement 1—source data 3. [file elife-76387-fig2-figsupp1-data3.zip › Figure 2 figure supplement 1- source data 3/2022-09-06 11h41m12s Chemiluminescence 5.000s Colon EROS KO actin.tif]

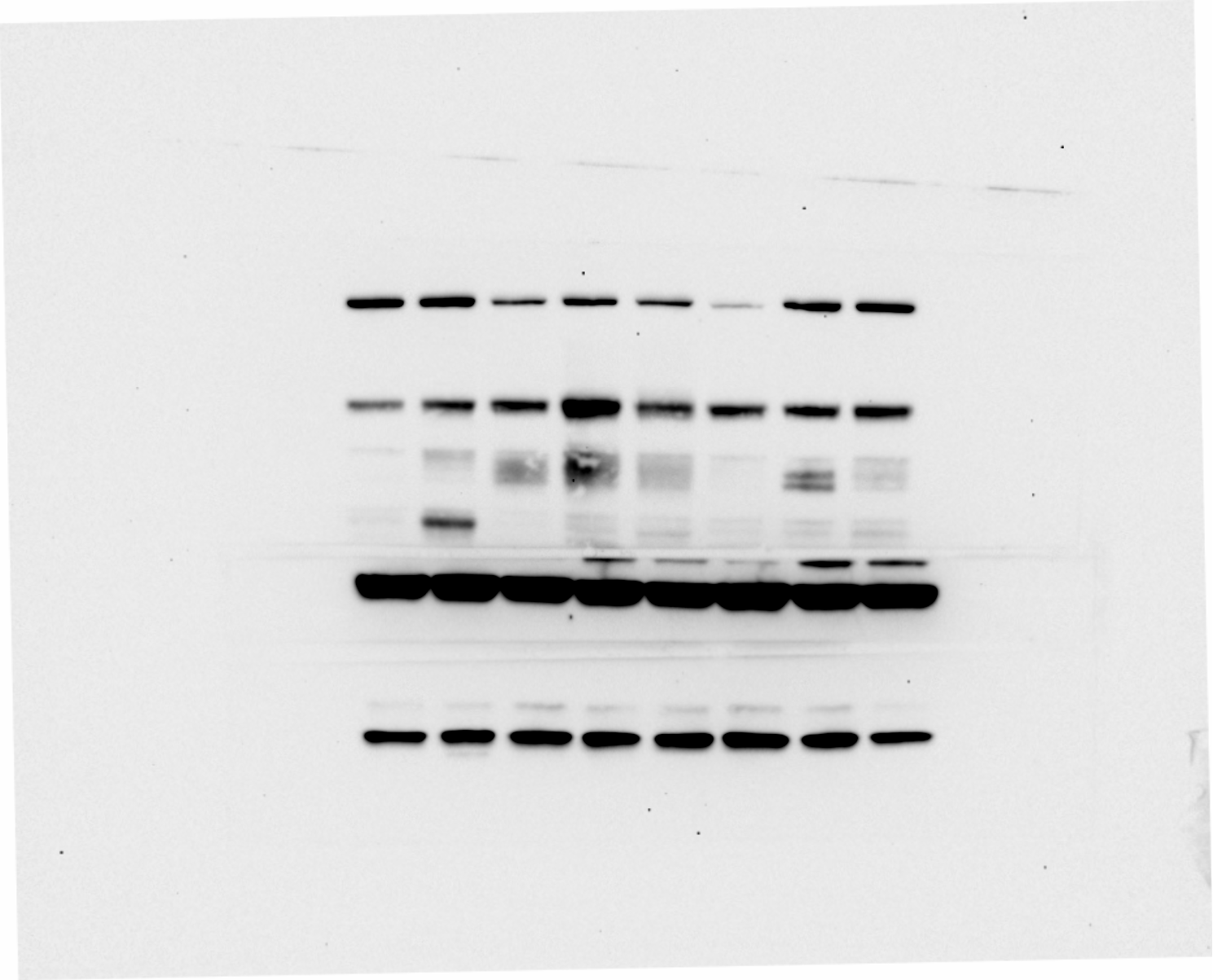

Supplement: Figure 2—figure supplement 1—source data 3. [file elife-76387-fig2-figsupp1-data3.zip › Figure 2 figure supplement 1- source data 3/2022-09-06 11h44m46s Chemiluminescence 212.306s Colon EROS KO EROS.tif]

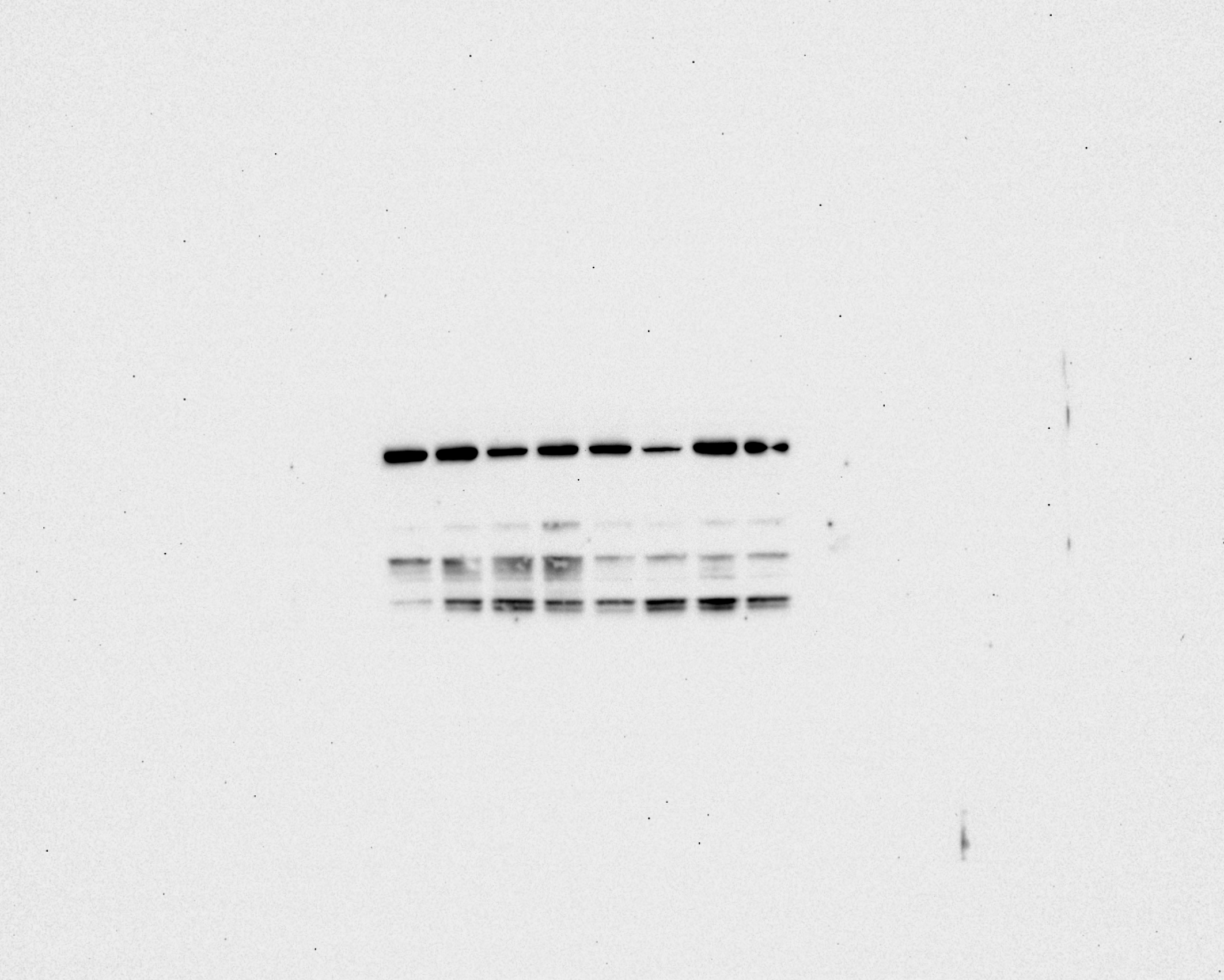

Supplement: Figure 2—figure supplement 1—source data 3. [file elife-76387-fig2-figsupp1-data3.zip › Figure 2 figure supplement 1- source data 3/2022-09-08 16h38m57s Chemiluminescence 1499.984s Colon EROS KO NOX1.tif]

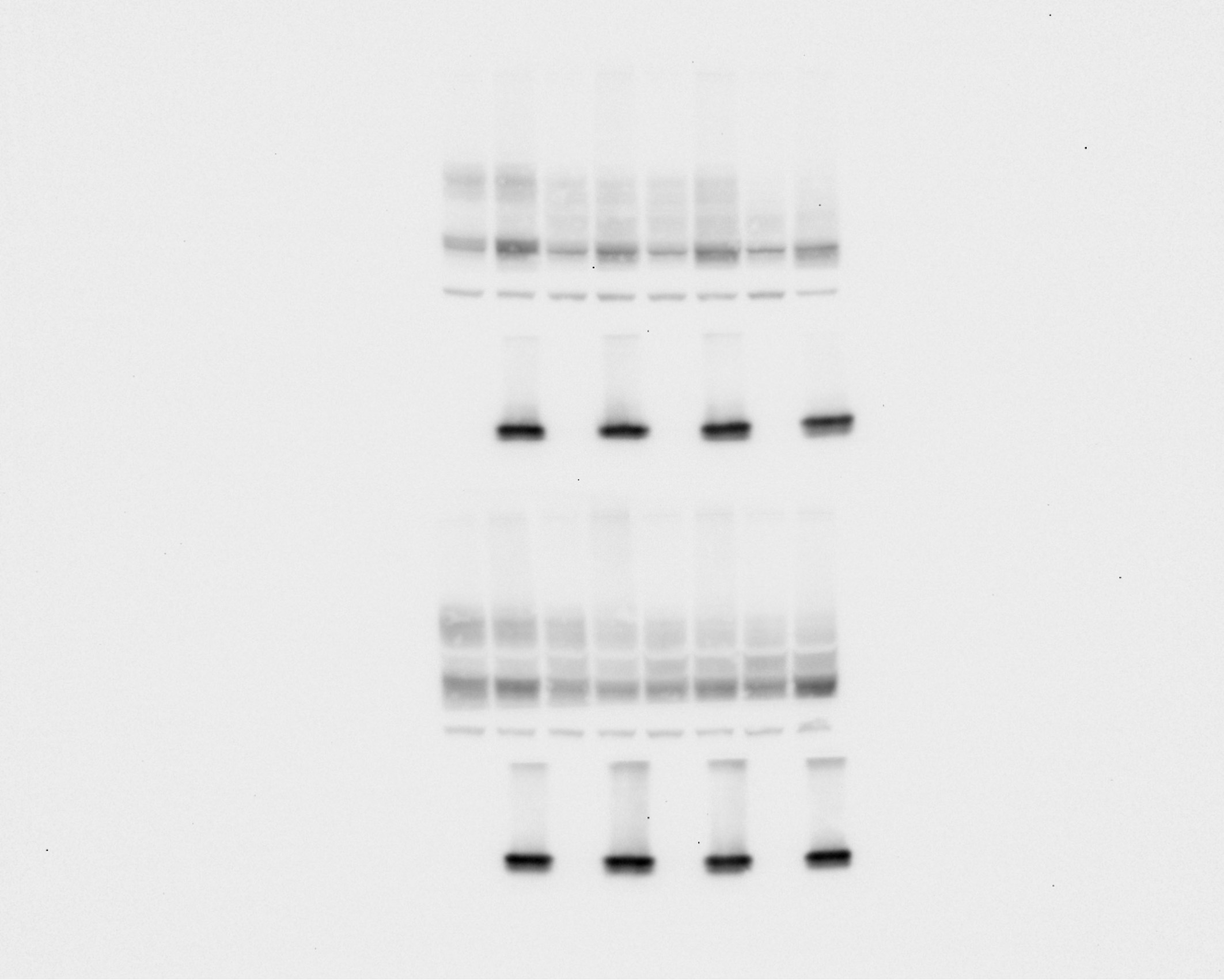

Supplement: Figure 3—source data 2. [file elife-76387-fig3-data2.zip › Figure 3- source data 2/2021-03-25 15h57m45s Chemiluminescence 164.137s HEK293 EROS NGI-1.tif]

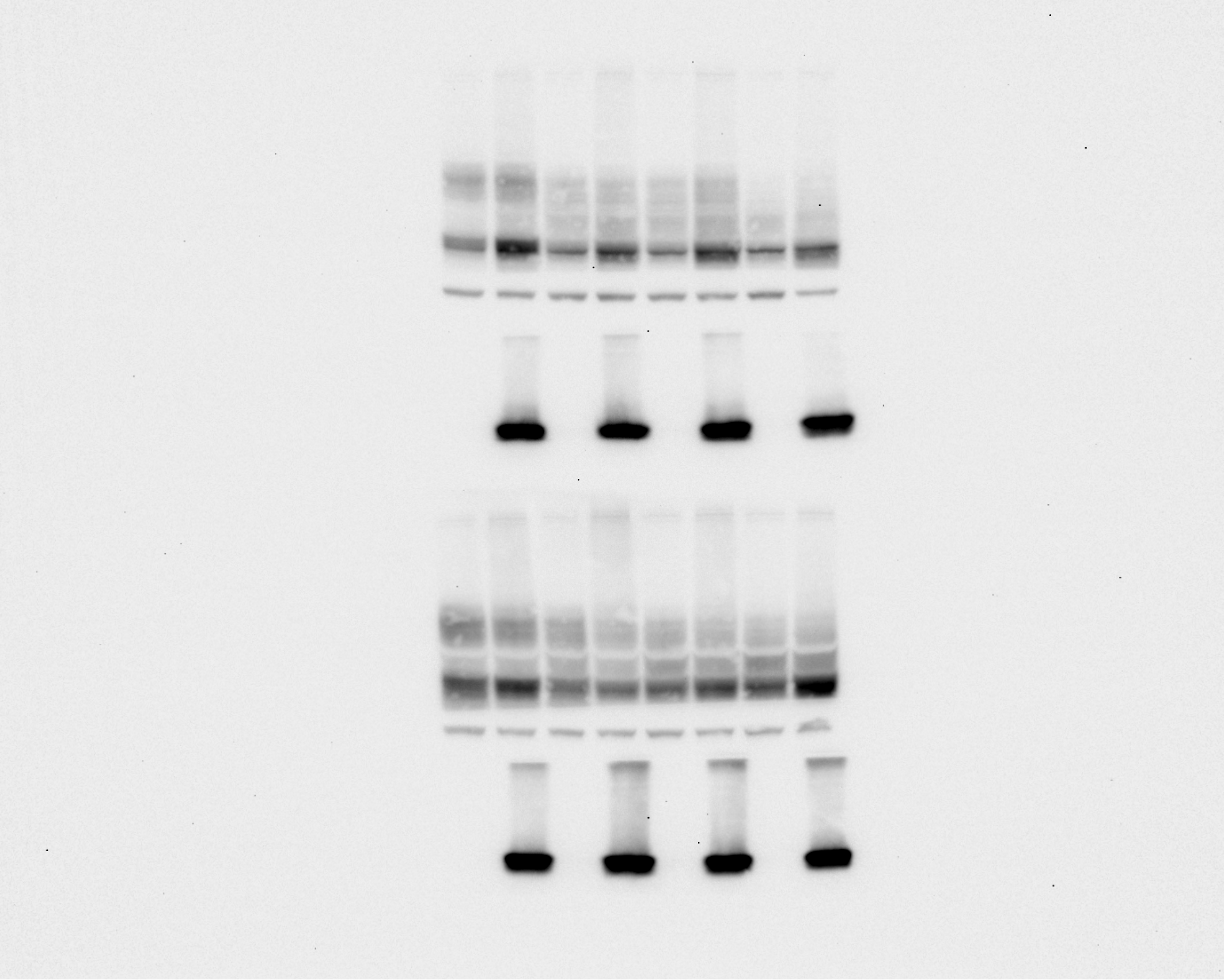

Supplement: Figure 3—source data 2. [file elife-76387-fig3-data2.zip › Figure 3- source data 2/2021-03-25 16h01m30s Chemiluminescence 369.653s HEK293 gp91 NGI-1.tif]

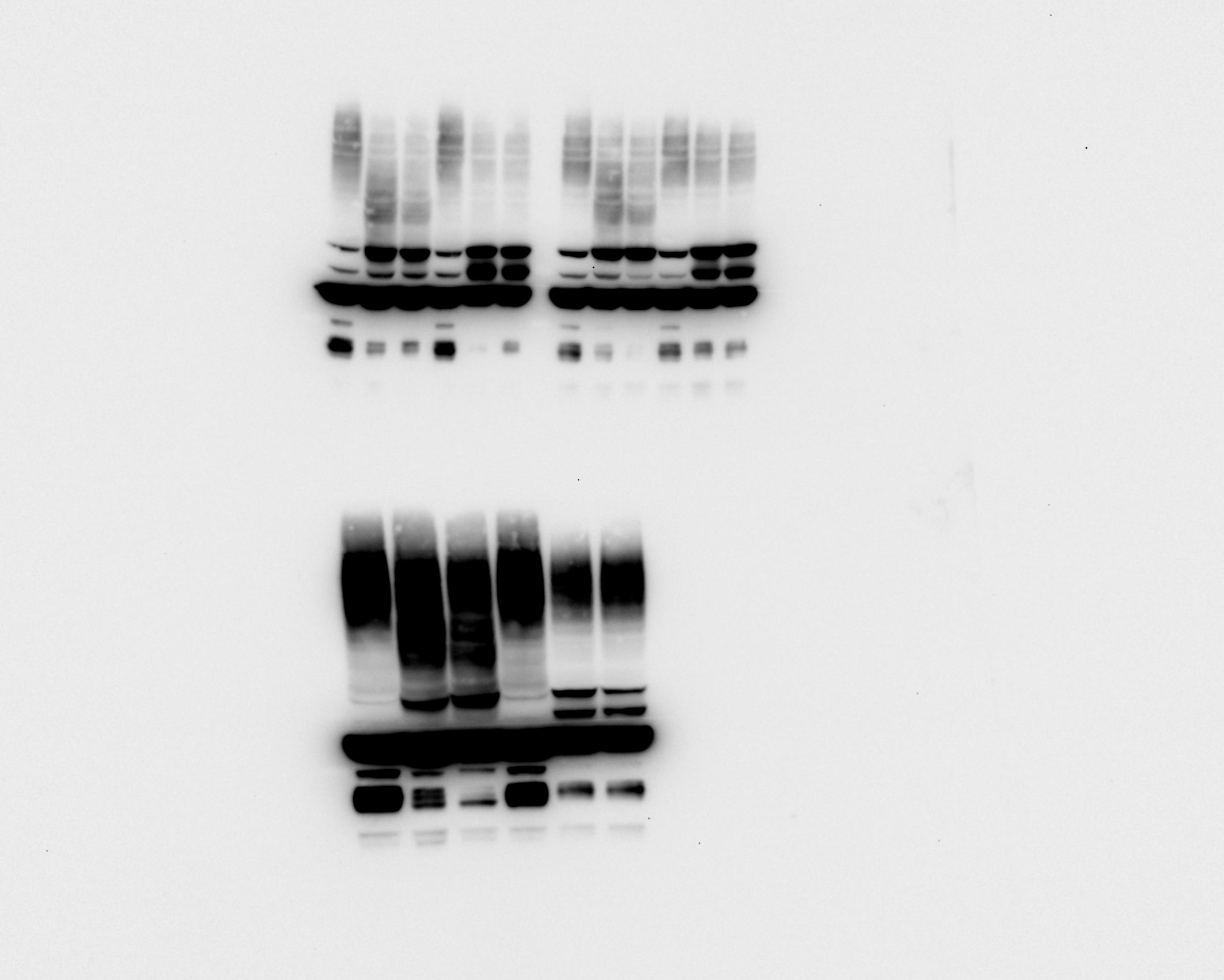

Supplement: Figure 3—source data 2. [file elife-76387-fig3-data2.zip › Figure 3- source data 2/2021-04-13 11h44m05s Chemiluminescence 112.758s PLB985 NGI-1 gp91.tif]

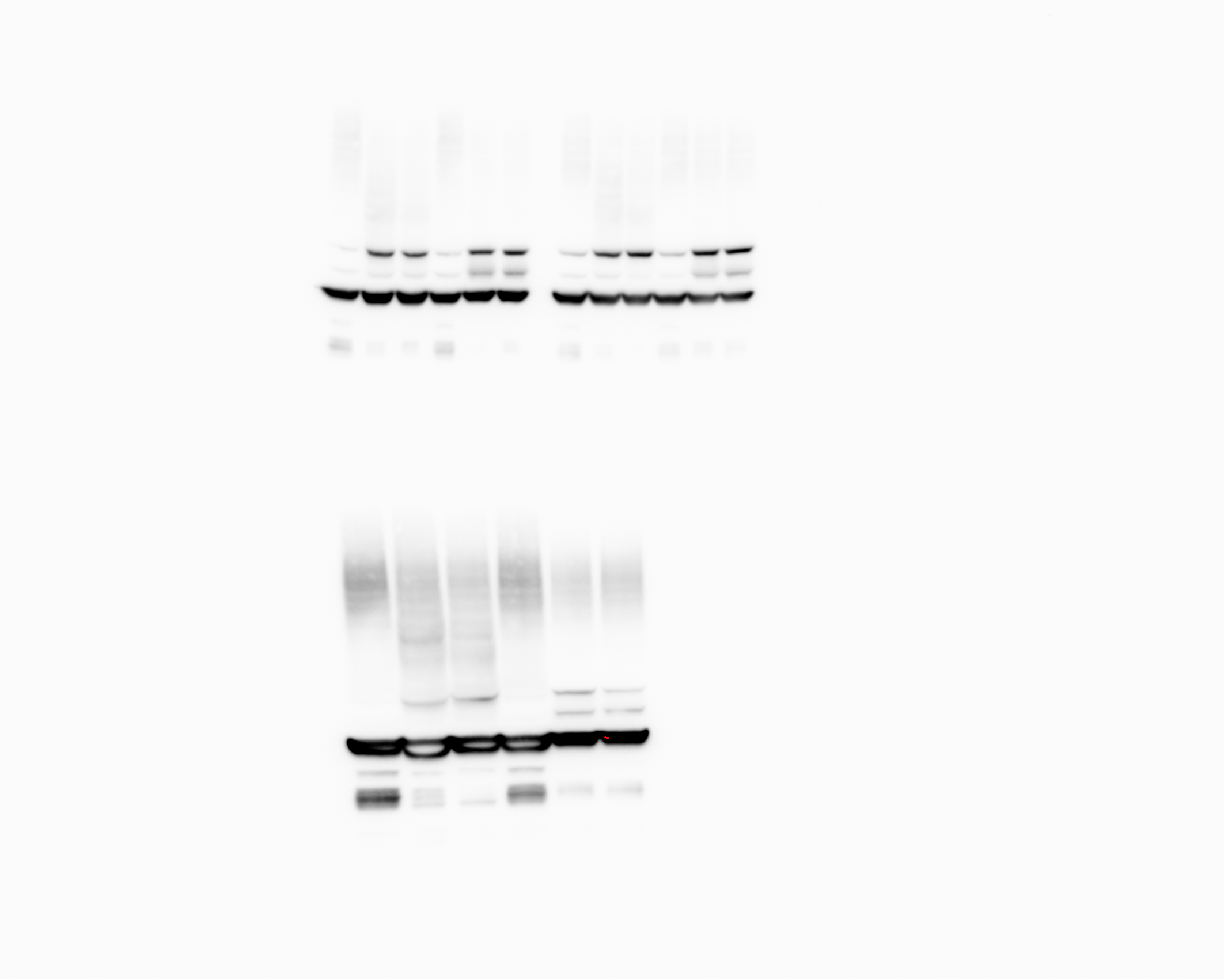

Supplement: Figure 3—source data 2. [file elife-76387-fig3-data2.zip › Figure 3- source data 2/2021-04-13 11h59m52s Chemiluminescence 20.000s PLB985 NGI-1 actin.tif]

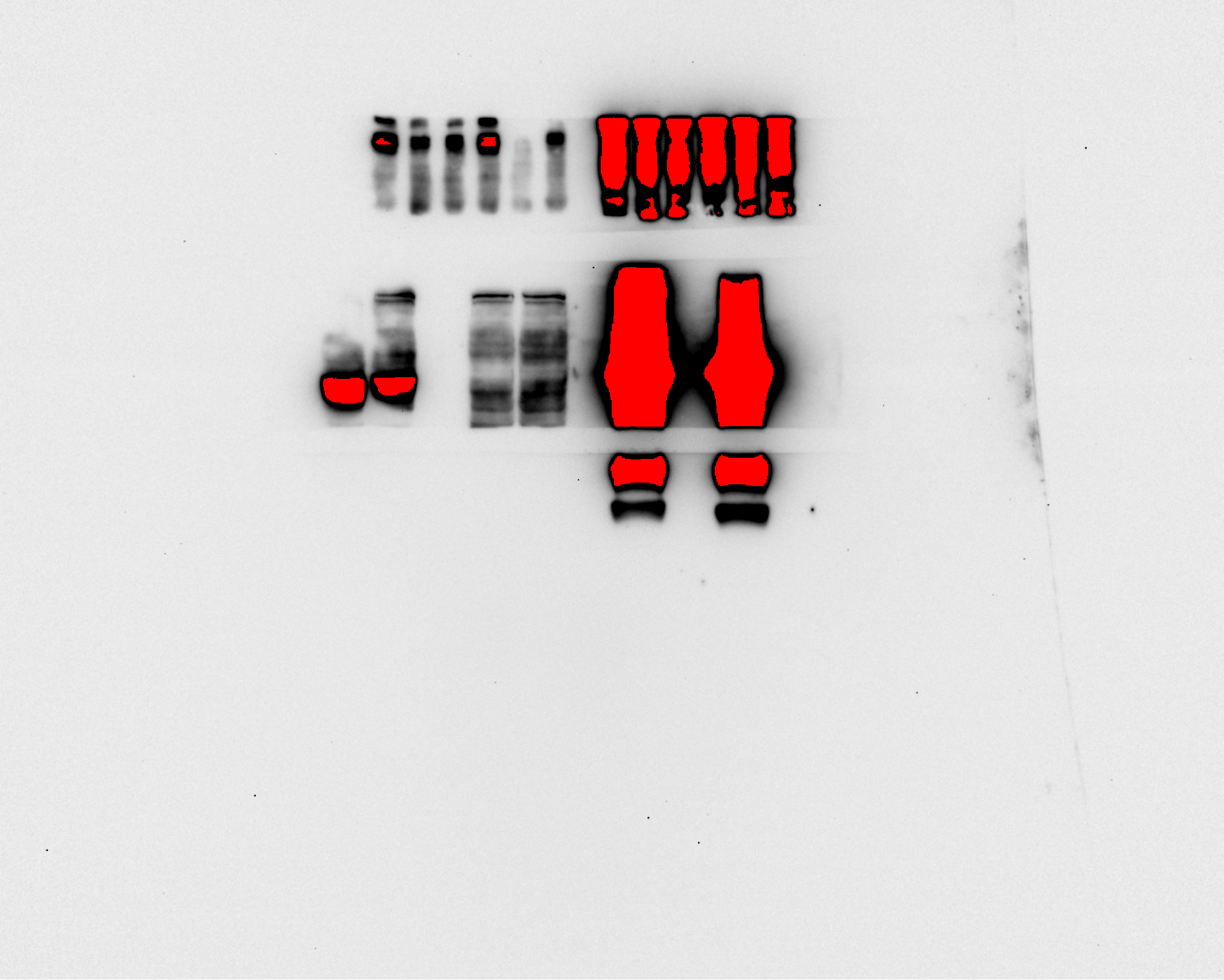

Supplement: Figure 3—source data 2. [file elife-76387-fig3-data2.zip › Figure 3- source data 2/2021-04-14 14h44m38s Chemiluminescence 357.930s PLB985 NGI-1 EROS.tif]

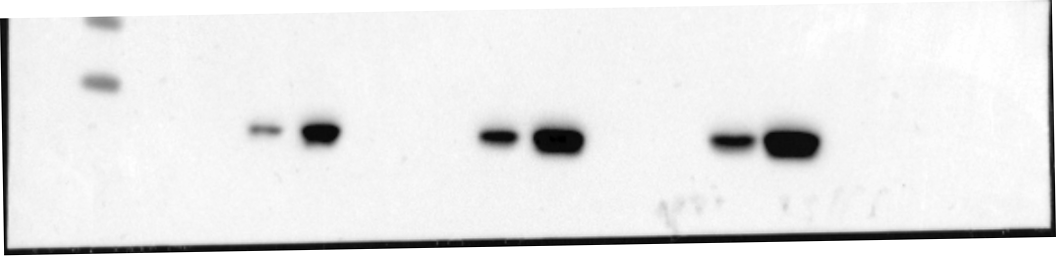

Supplement: Figure 3—source data 2. [file elife-76387-fig3-data2.zip › Figure 3- source data 2/2022-05-25 14h37m03s Chemiluminescence 112.758s+2022-05-25 14h34m31s Colorimetric 0.048s HEK293 WT STT3A KO STT3A KO 3rd exp Eros.tif]

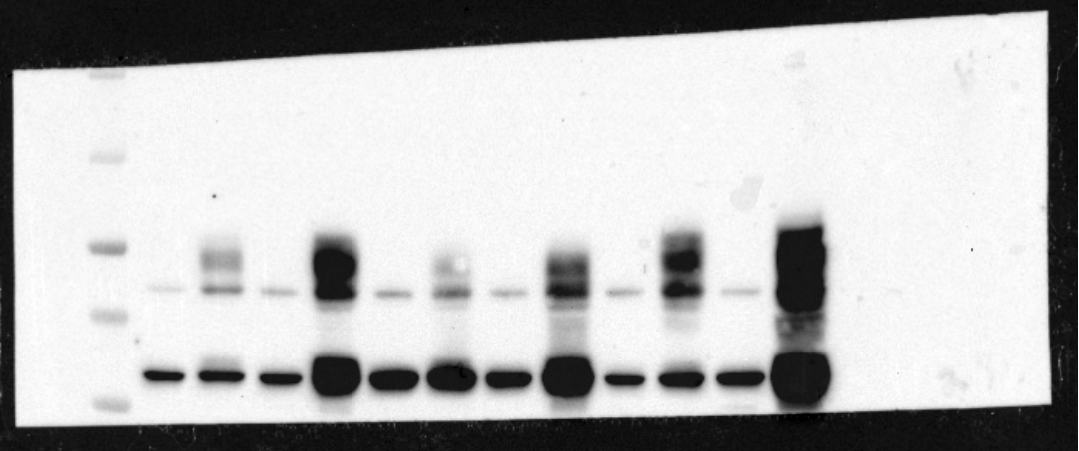

Supplement: Figure 3—source data 2. [file elife-76387-fig3-data2.zip › Figure 3- source data 2/2022-05-25 15h30m48s Chemiluminescence 579.129s+2022-05-25 15h19m42s Colorimetric 0.035s HEK293 WT STT3AKO STT3BKO 3rd exp gp91.tif]

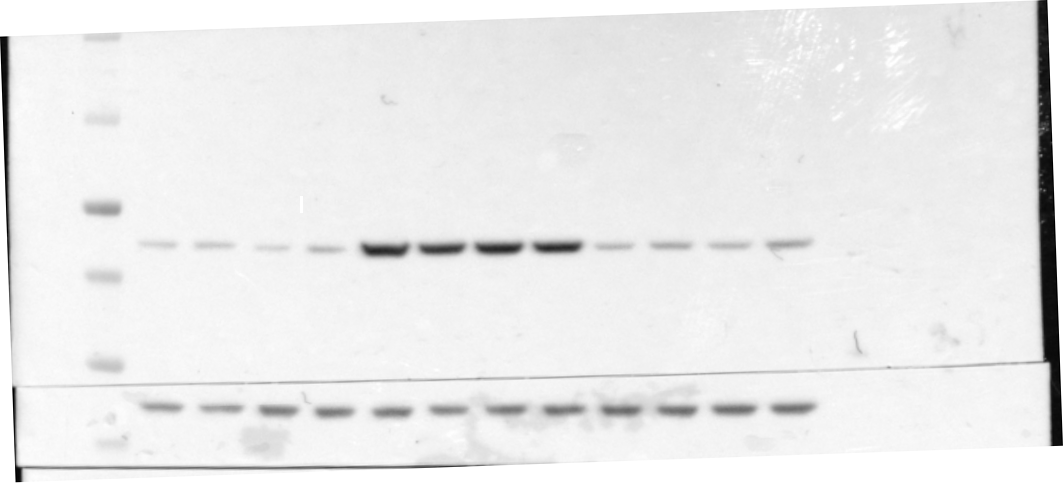

Supplement: Figure 3—source data 2. [file elife-76387-fig3-data2.zip › Figure 3- source data 2/2022-05-27 15h38m51s Colorimetric 0.146s+2022-05-27 15h39m47s Chemiluminescence 10.000s HEK293 WT STT3A KO STT3B KO 3rd exp BiP actin.tif]

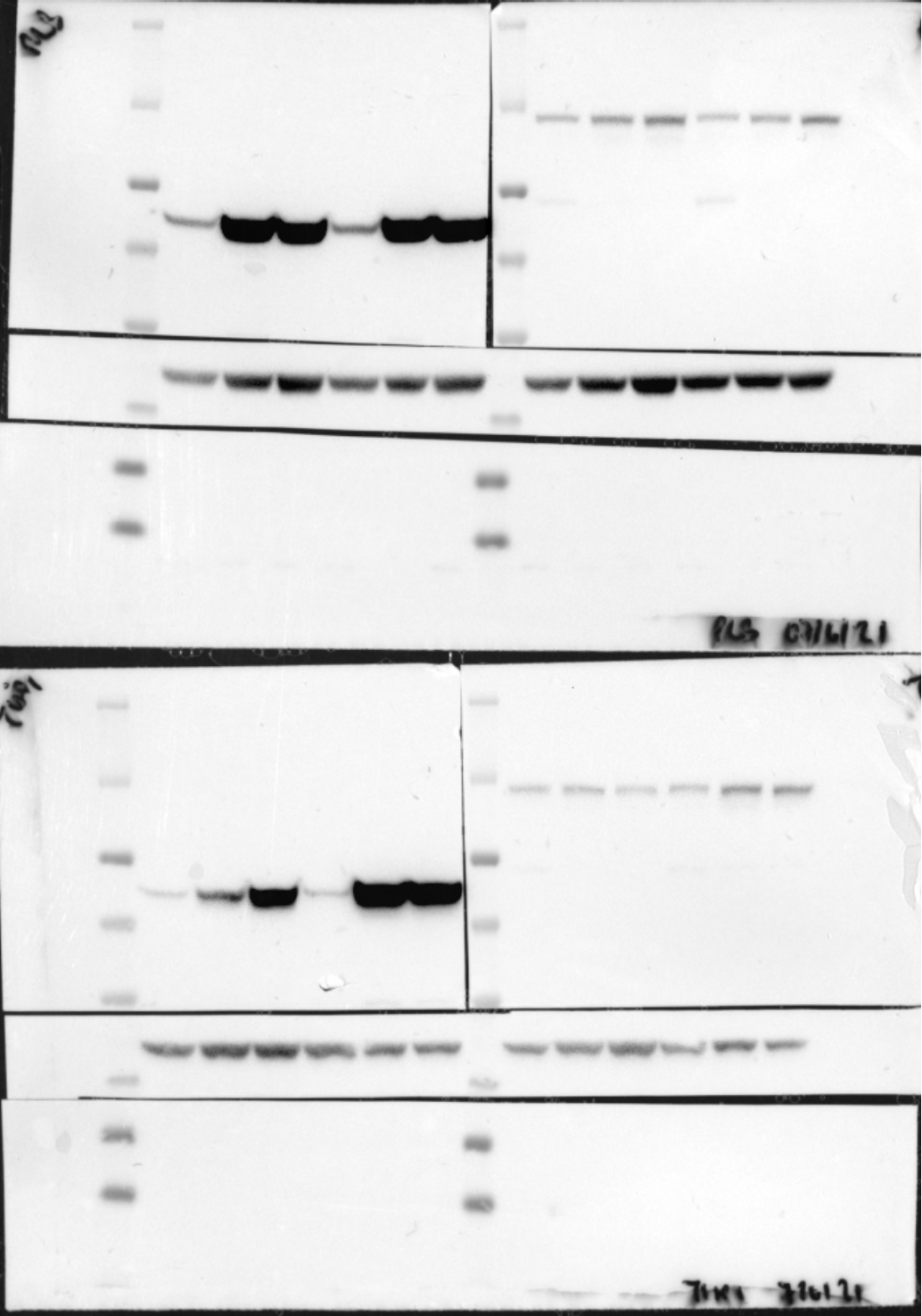

Supplement: Figure 3—figure supplement 1—source data 1. [file elife-76387-fig3-figsupp1-data1.zip › Figure 3 figure supplement 1- source data 1/2021-06-08 14h01m26s Colorimetric 0.202s+2021-06-08 14h26m06s Chemiluminescence 20.000s PBL985 NGI-1 Tunica BiP.tif]

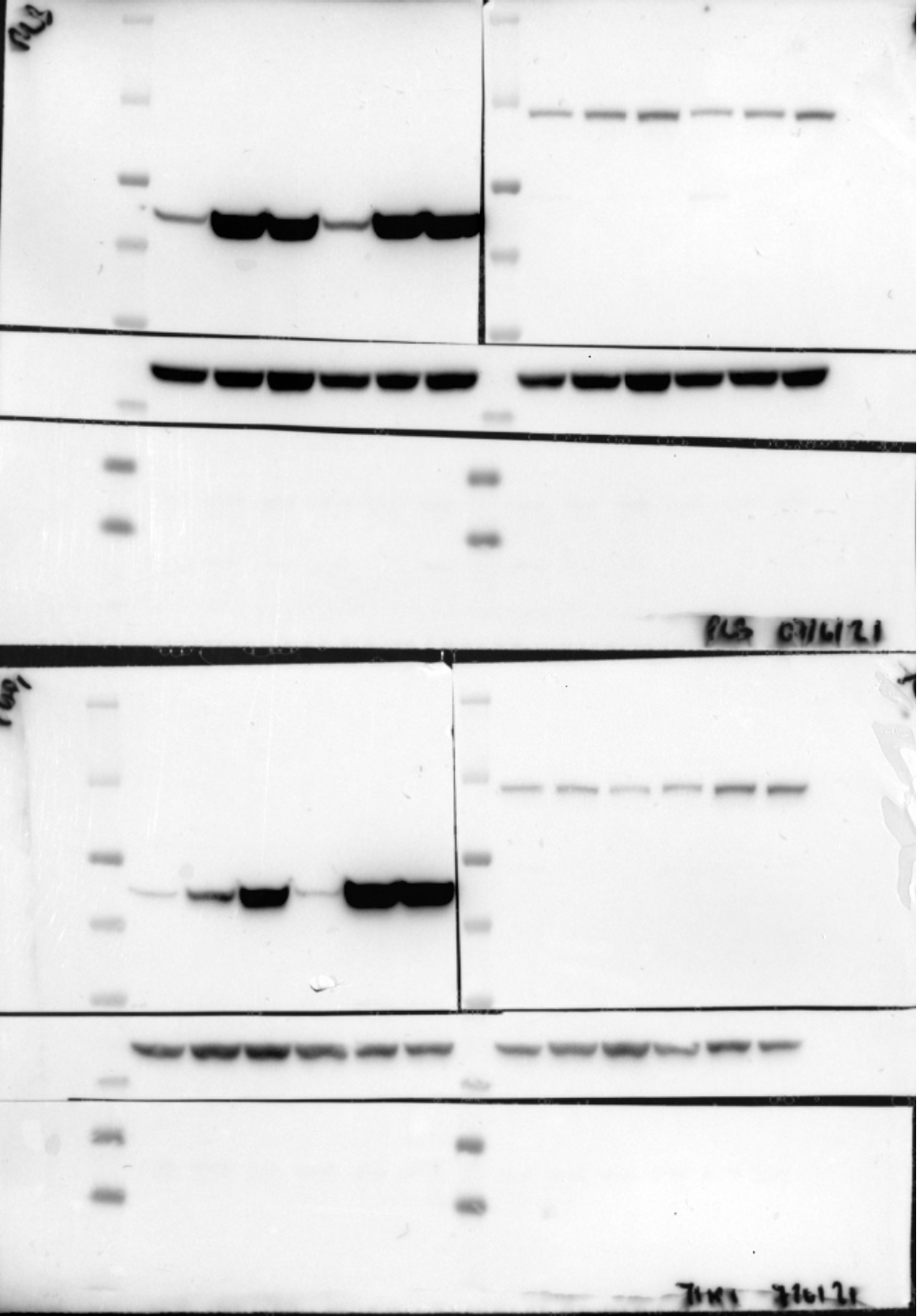

Supplement: Figure 3—figure supplement 1—source data 1. [file elife-76387-fig3-figsupp1-data1.zip › Figure 3 figure supplement 1- source data 1/2021-06-08 14h03m55s Chemiluminescence 72.631s+2021-06-08 14h01m26s Colorimetric 0.202s PLB985 NGI-1 Tunica actin.tif]

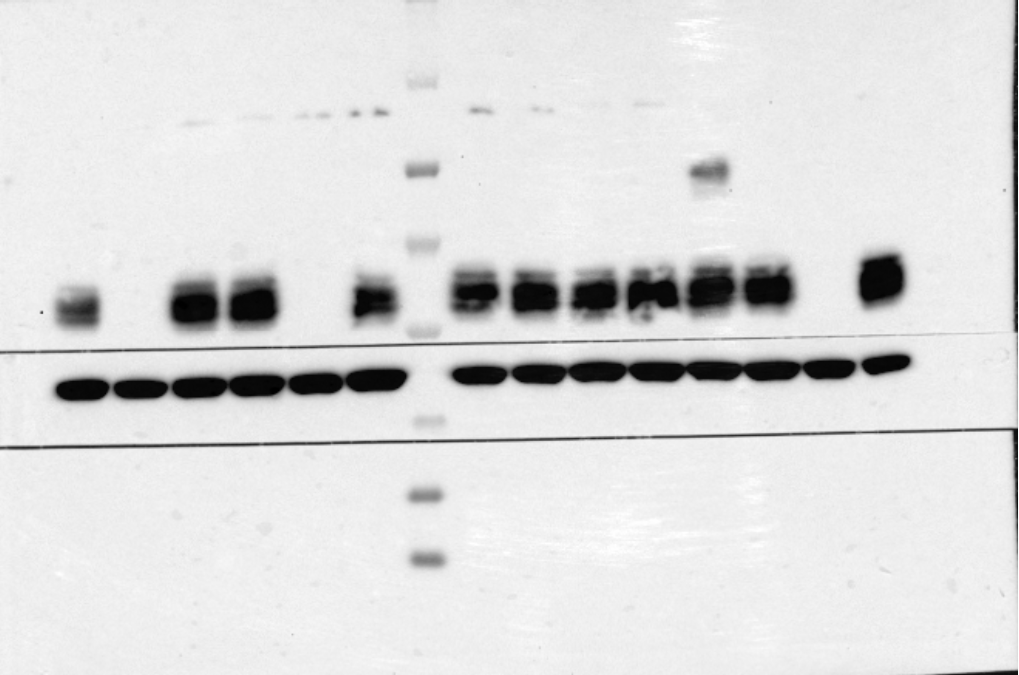

Supplement: Figure 3—figure supplement 1—source data 1. [file elife-76387-fig3-figsupp1-data1.zip › Figure 3 figure supplement 1- source data 1/2022-06-10 13h31m12s Chemiluminescence 256.896s+2022-06-10 13h26m04s Colorimetric 0.173s HEK293 WT STT3A KO STT3B KO STT3A expression.tif]

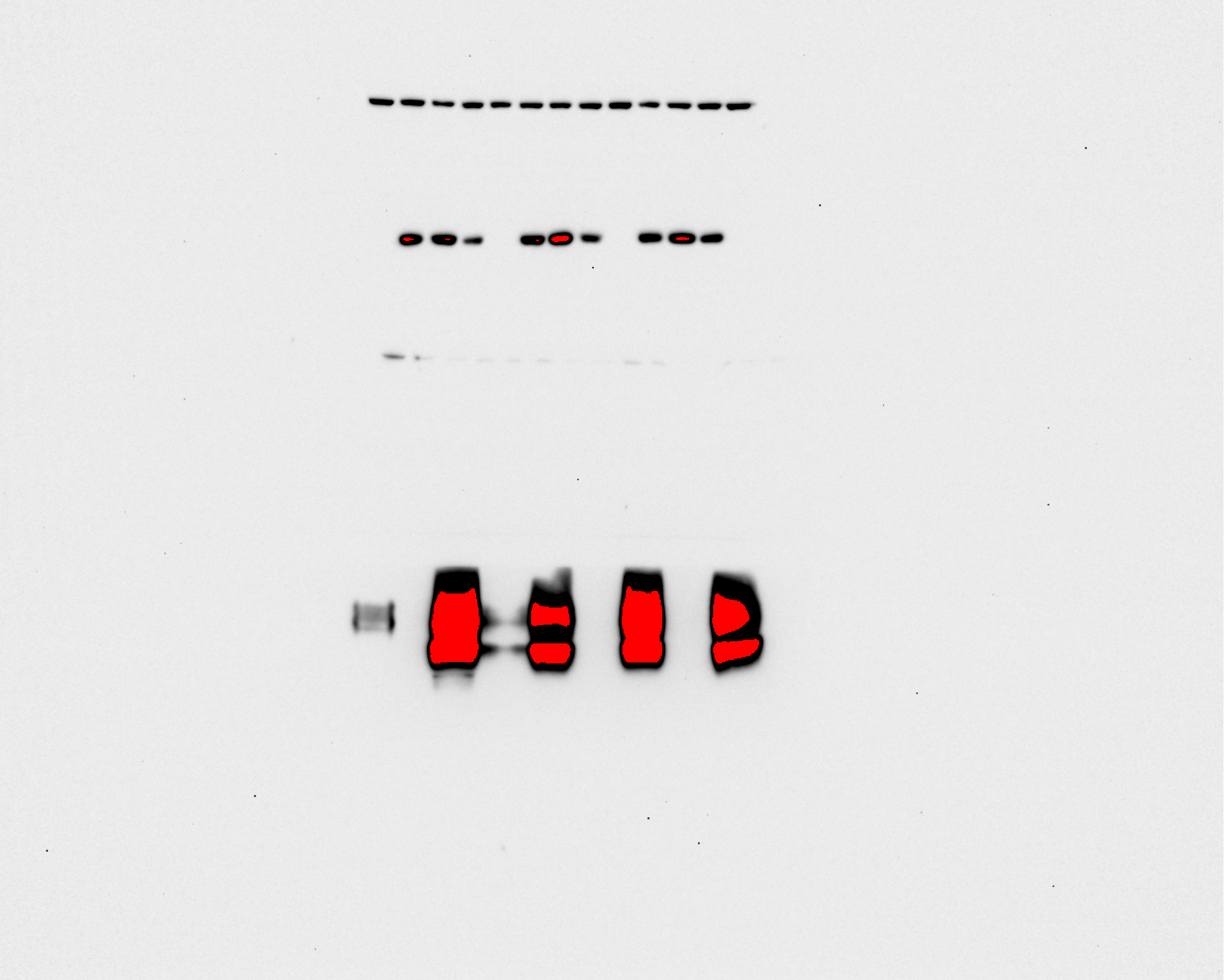

Supplement: Figure 3—figure supplement 1—source data 1. [file elife-76387-fig3-figsupp1-data1.zip › Figure 3 figure supplement 1- source data 1/2022-07-01 14h26m15s Chemiluminescence 269.128s HEK293 WT STT3A STT3B KO NGI Gp91 eros Actin.tif]

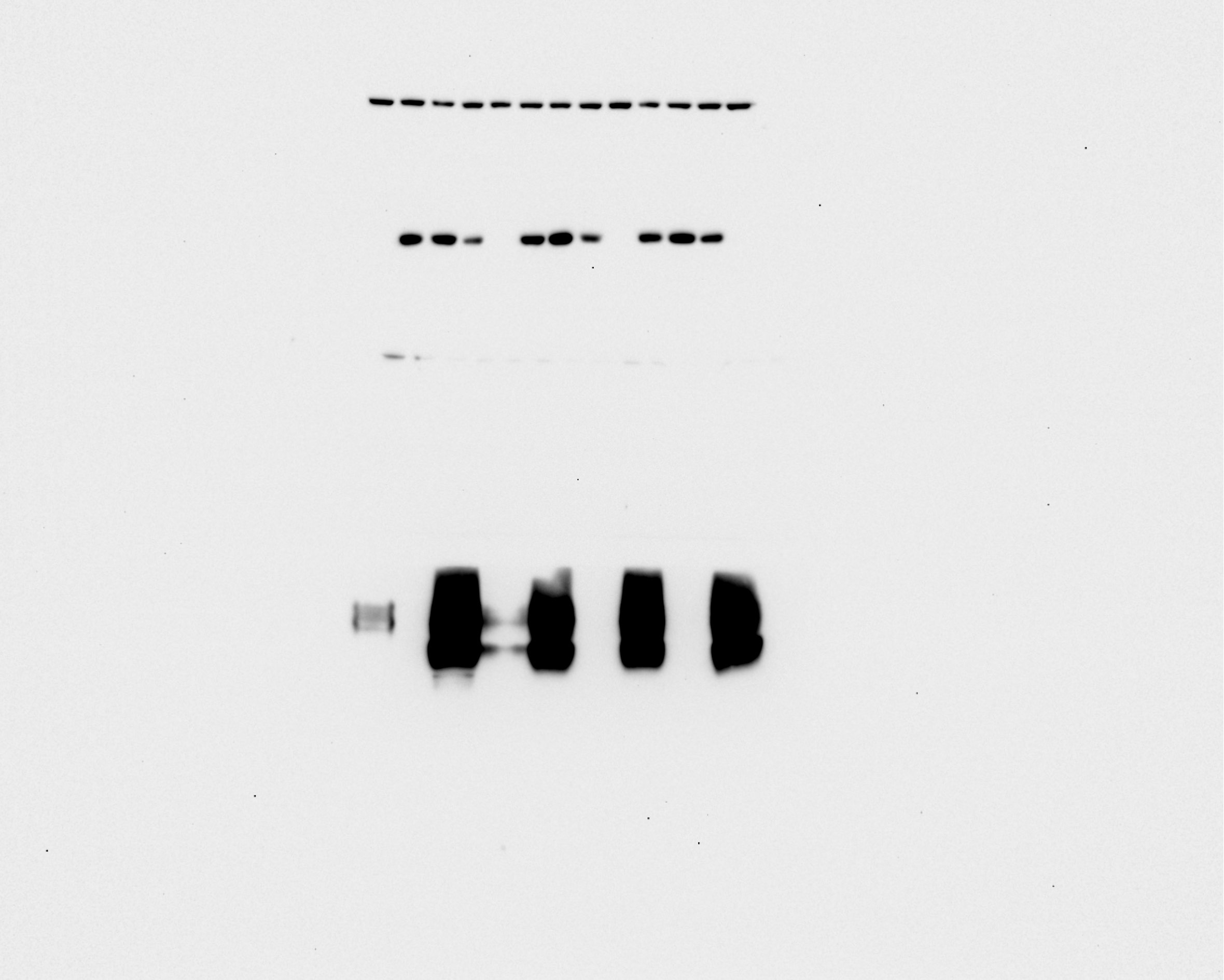

Supplement: Figure 3—figure supplement 1—source data 1. [file elife-76387-fig3-figsupp1-data1.zip › Figure 3 figure supplement 1- source data 1/2022-07-01 14h26m15s Chemiluminescence 269.128s HEK293 WT STT3A STT3B KO NGI Gp91 eros EROS.tif]

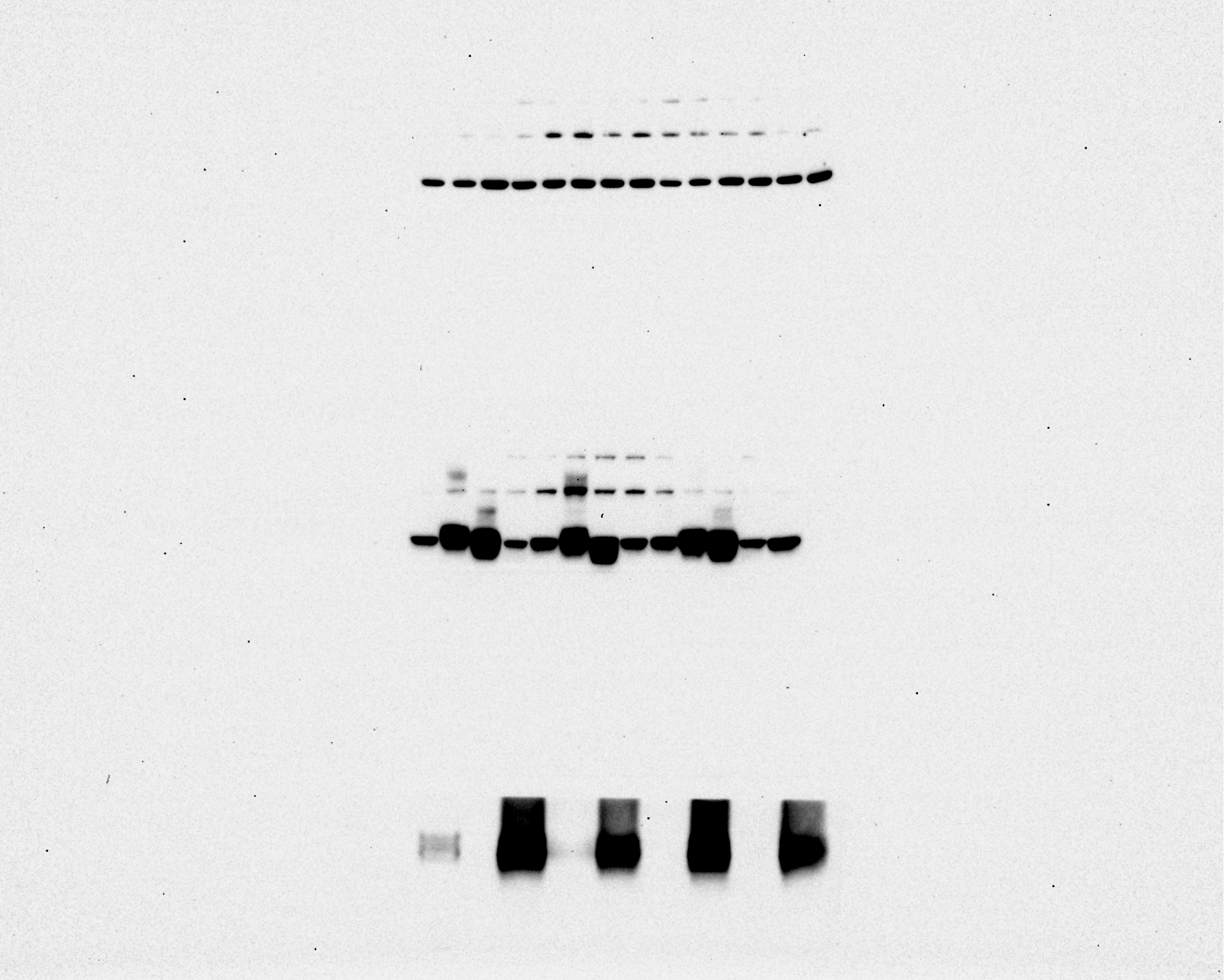

Supplement: Figure 3—figure supplement 1—source data 1. [file elife-76387-fig3-figsupp1-data1.zip › Figure 3 figure supplement 1- source data 1/2022-07-01 14h26m15s Chemiluminescence 269.128s HEK293 WT STT3A STT3B KO NGI Gp91 eros Gp91phox.tif]

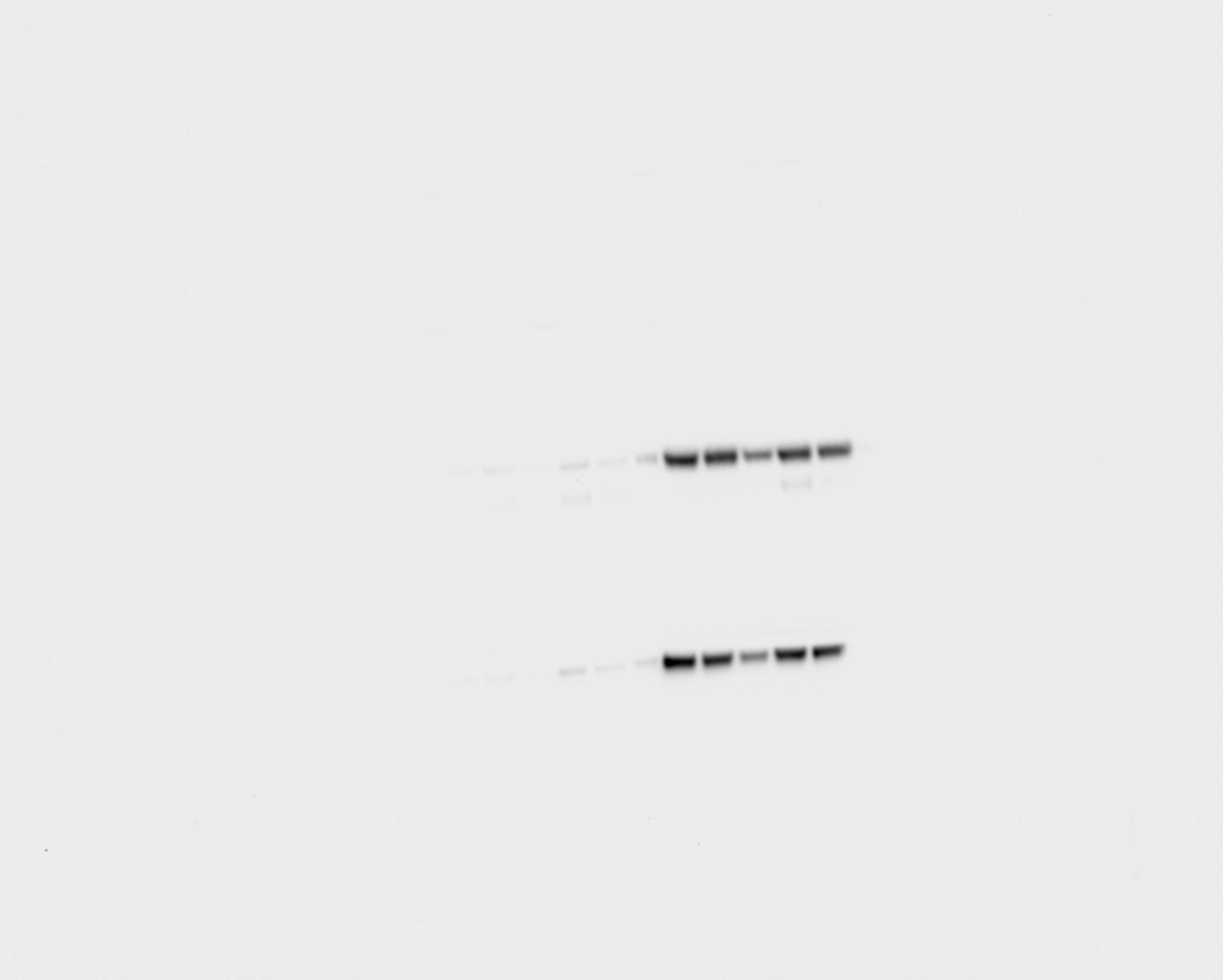

Supplement: Figure 5—source data 1. [file elife-76387-fig5-data1.zip › Figure 5- source data 1/2019-12-18 12h28m17s Chemiluminescence 10.000s BMDM EROS KO P2X7.tif]

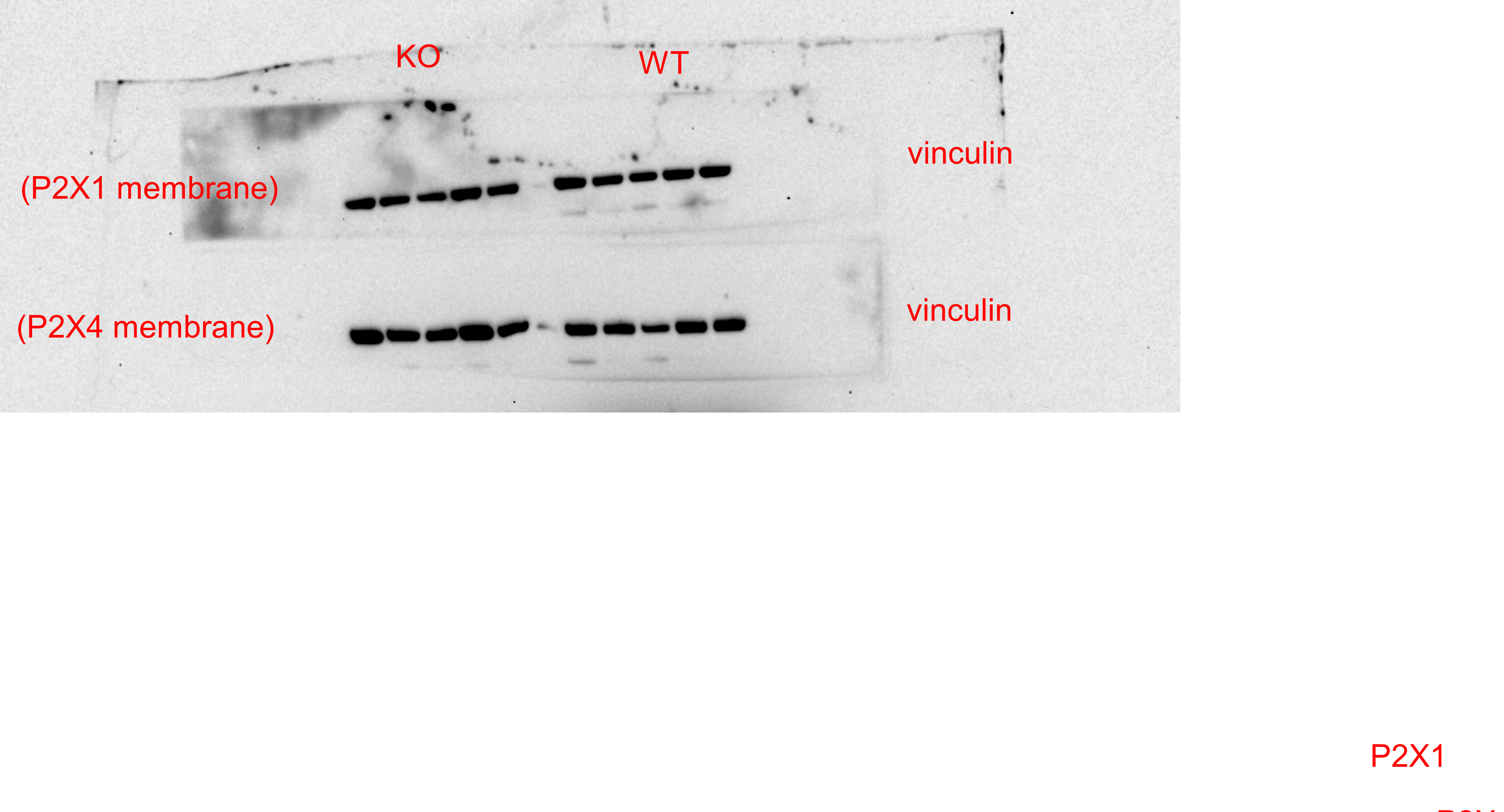

Supplement: Figure 5—source data 1. [file elife-76387-fig5-data1.zip › Figure 5- source data 1/20191218 vinculin femto 15.tif]

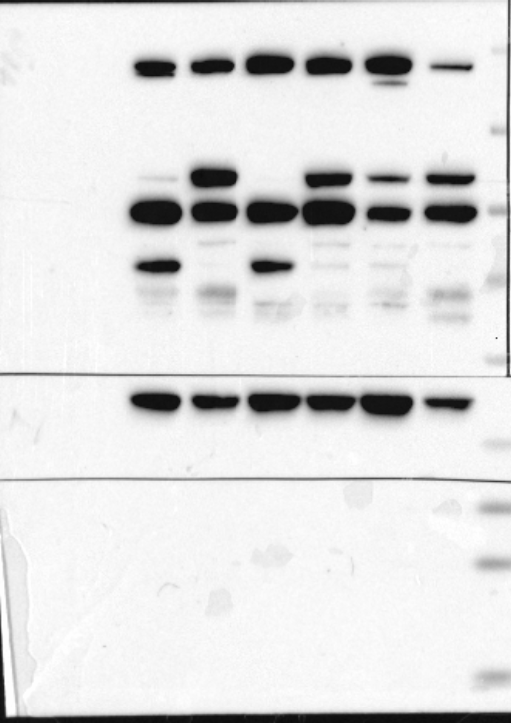

Supplement: Figure 5—source data 1. [file elife-76387-fig5-data1.zip › Figure 5- source data 1/2022-08-18 11h08m16s Chemiluminescence 215.516s+2022-08-18 11h03m51s Colorimetric 0.056s iPS P2X7.tif]

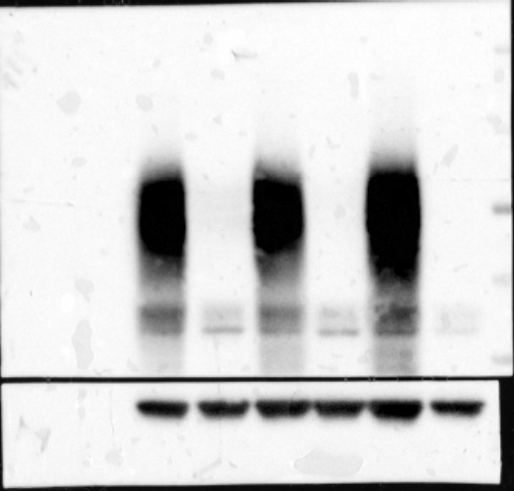

Supplement: Figure 5—source data 1. [file elife-76387-fig5-data1.zip › Figure 5- source data 1/2022-09-01 13h31m01s Colorimetric 0.026s+2022-09-01 14h00m42s Chemiluminescence 15.000s iPS actin.tif]

## Slide 1
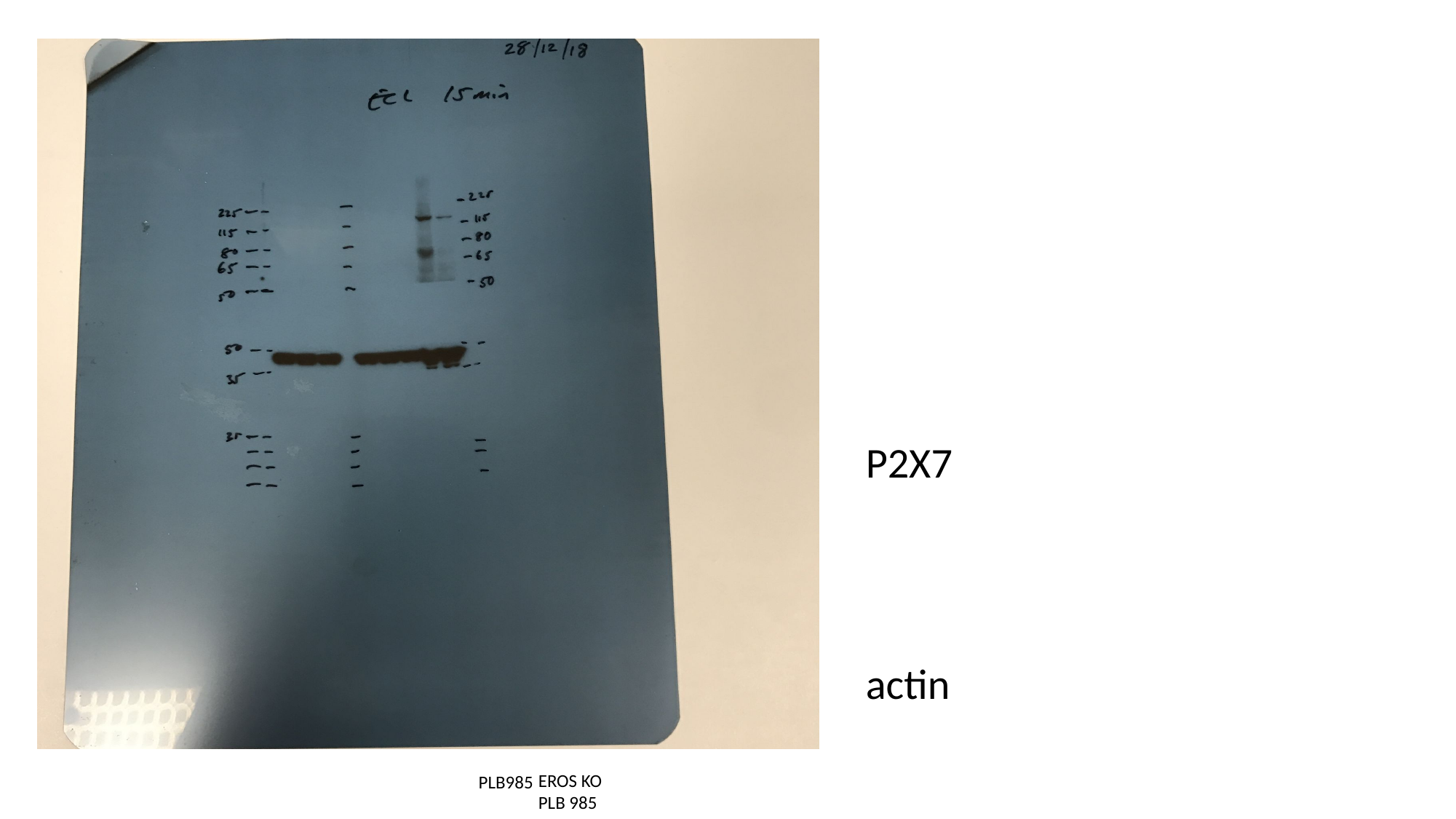

P2X7
actin
EROS KO
PLB 985
PLB985

Supplement: Figure 5—source data 1. [file elife-76387-fig5-data1.zip › Figure 5- source data 1/P2X7 on iPS derived macrophages and plb985 cells 28-12-2018 2 Lyra.pptx]

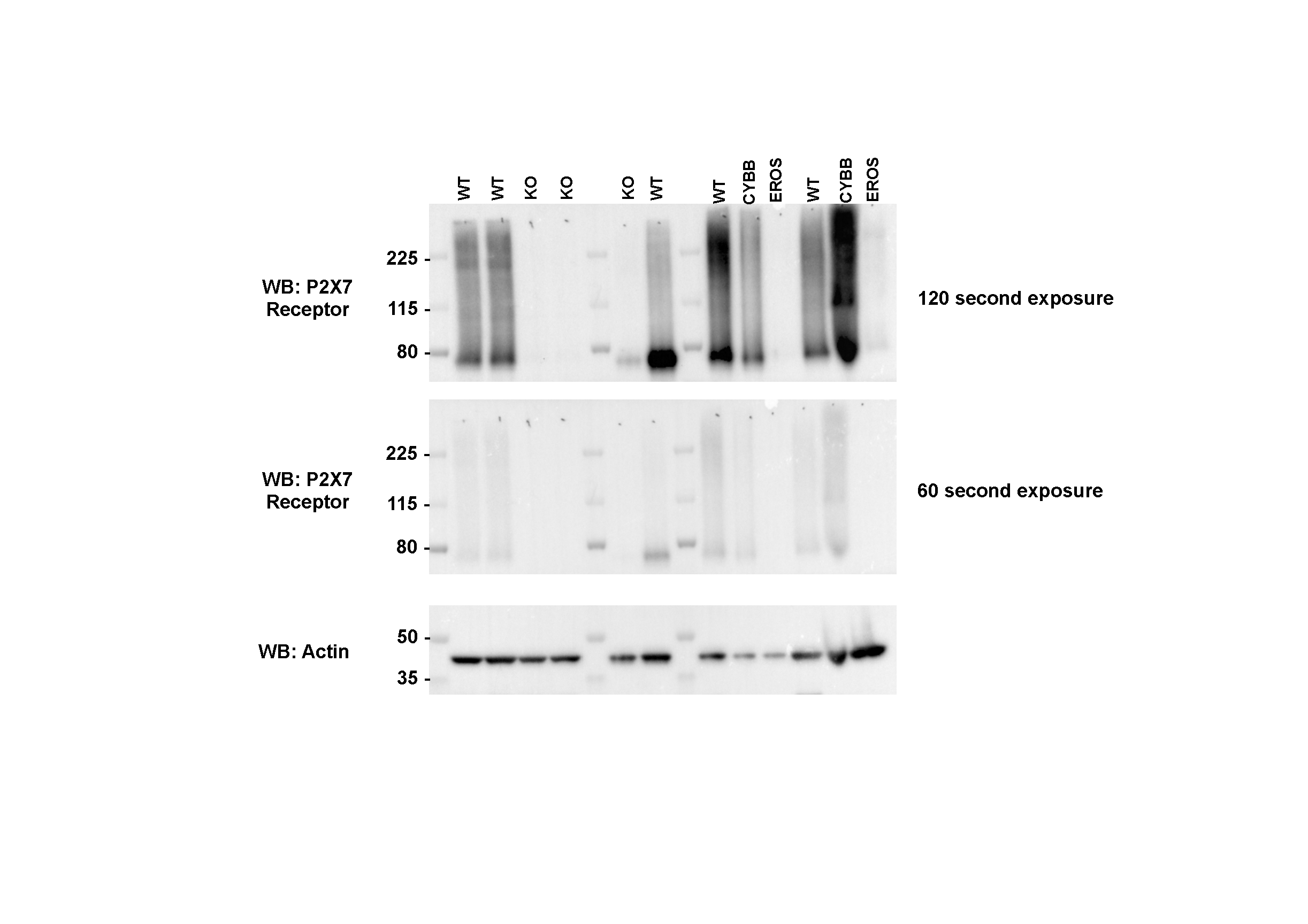

Supplement: Figure 5—source data 1. [file elife-76387-fig5-data1.zip › Figure 5- source data 1/P2X7R results John and Dave 2016.tiff]

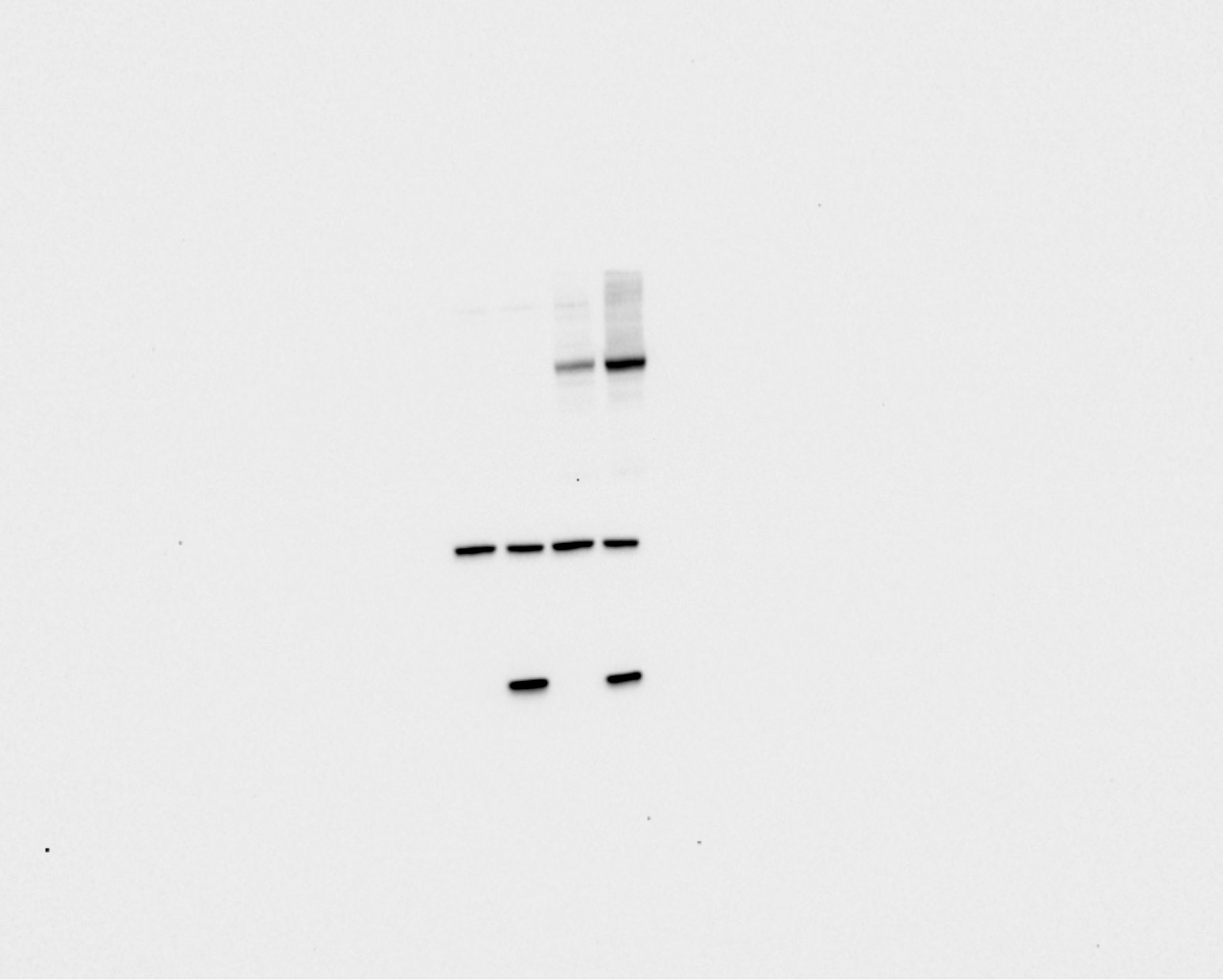

Supplement: Figure 5—source data 2. [file elife-76387-fig5-data2.zip › Figure 5- source data 2/2020-02-12 11h10m34s Chemiluminescence 59.583s HEK293 P2X7 co transfection actin.tif]

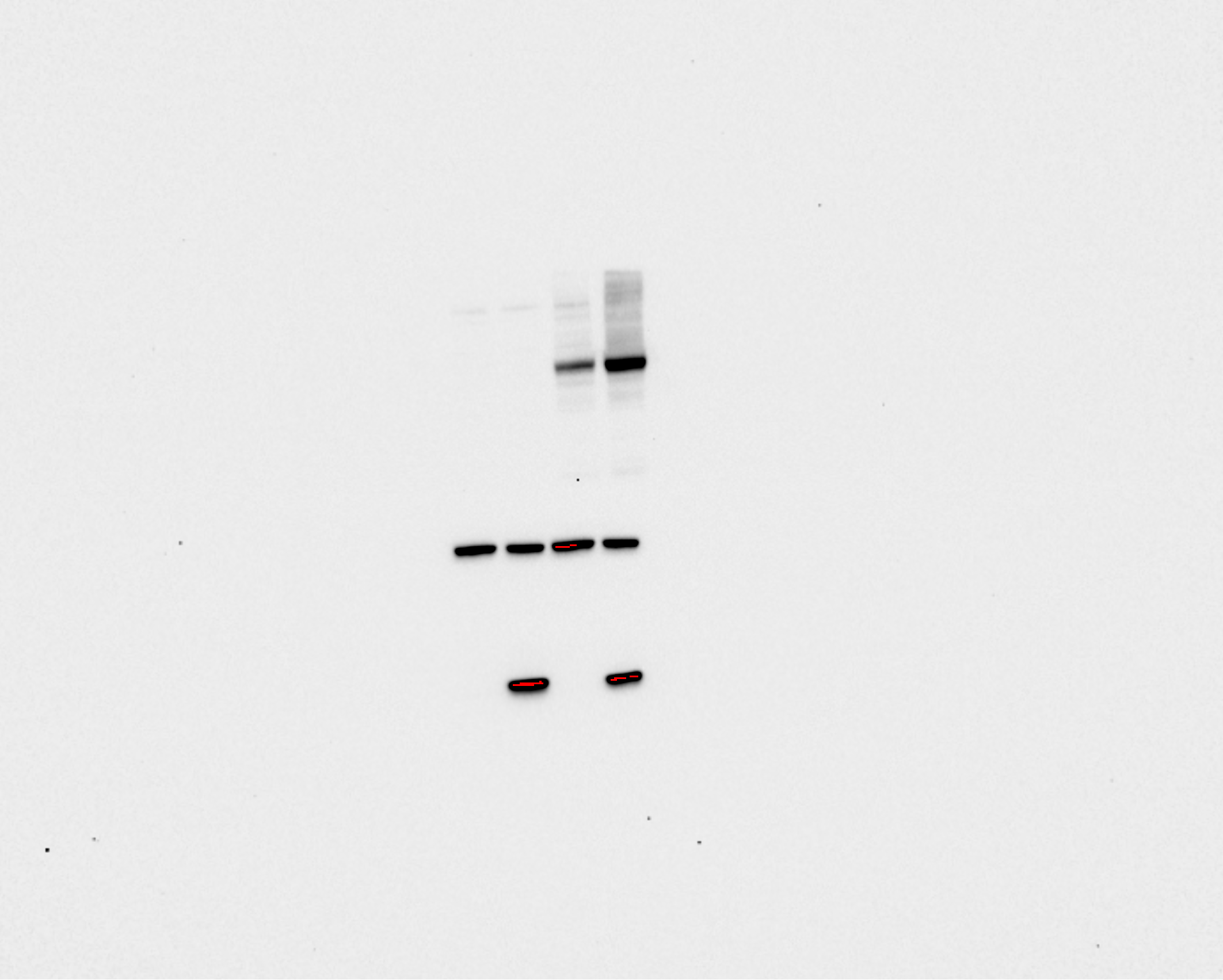

Supplement: Figure 5—source data 2. [file elife-76387-fig5-data2.zip › Figure 5- source data 2/2020-02-12 11h11m25s Chemiluminescence 109.166s HEK293 P2X7 co transfection.tif]

## Slide 1
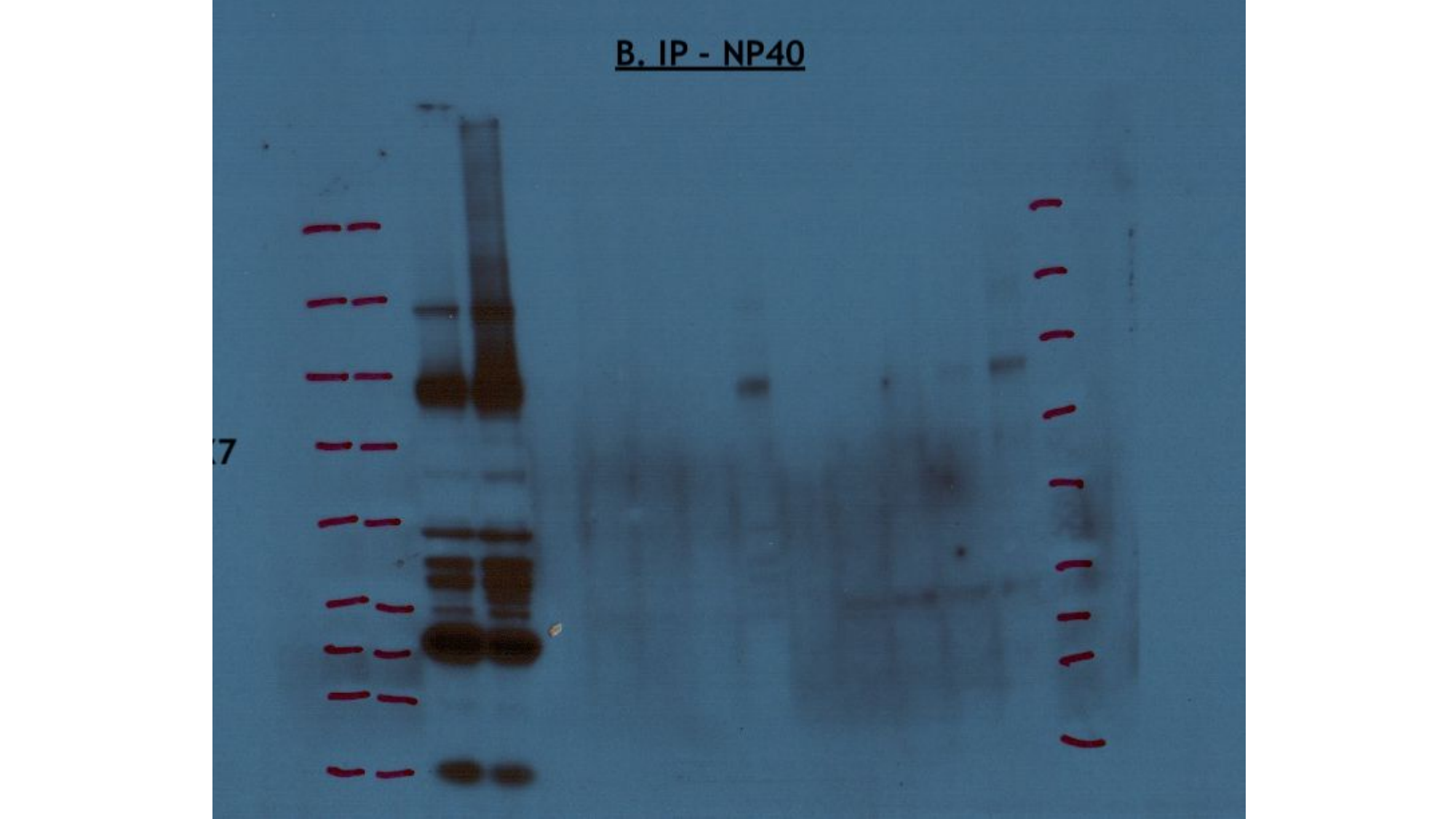

## Slide 2
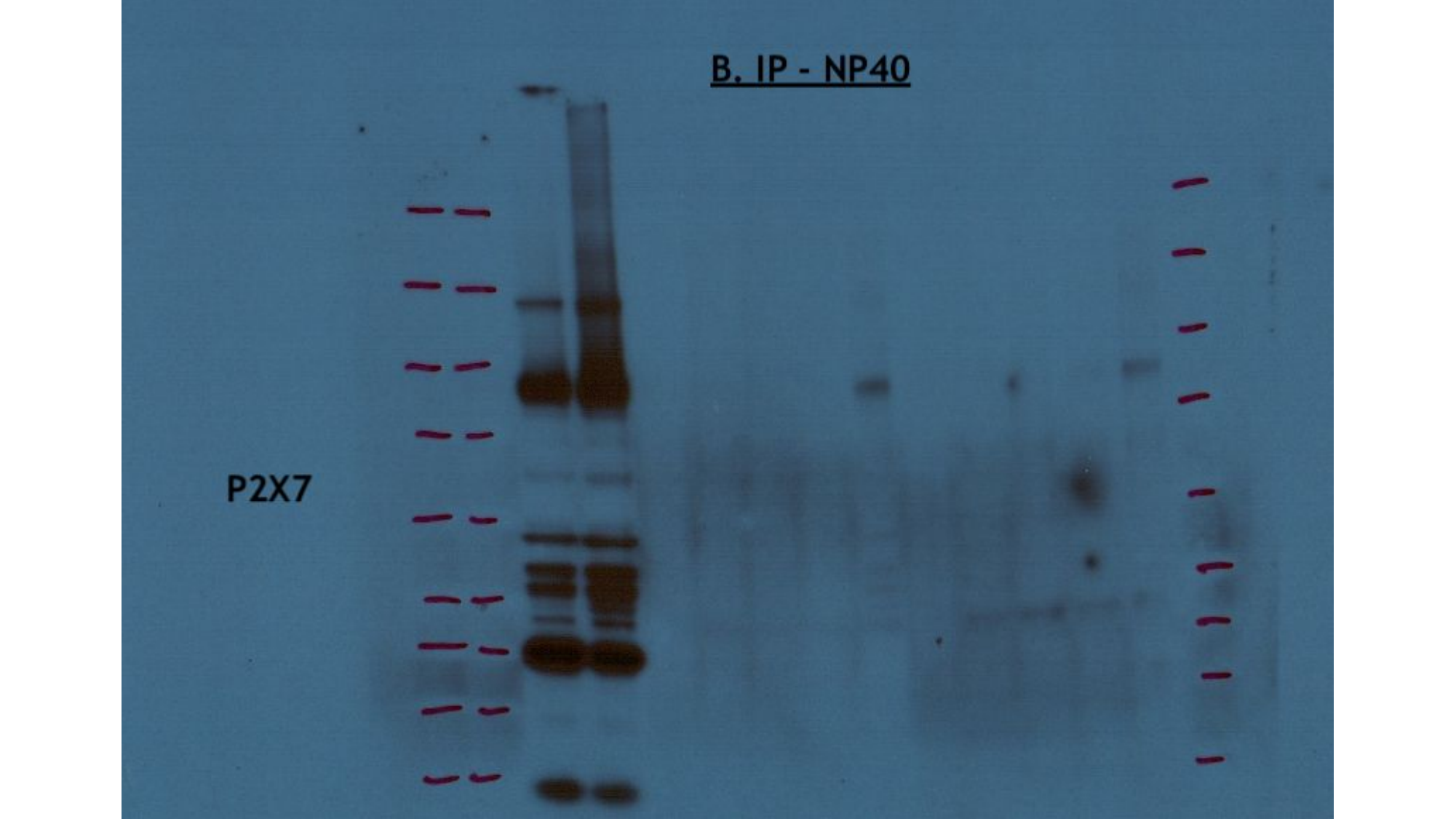

## Slide 3
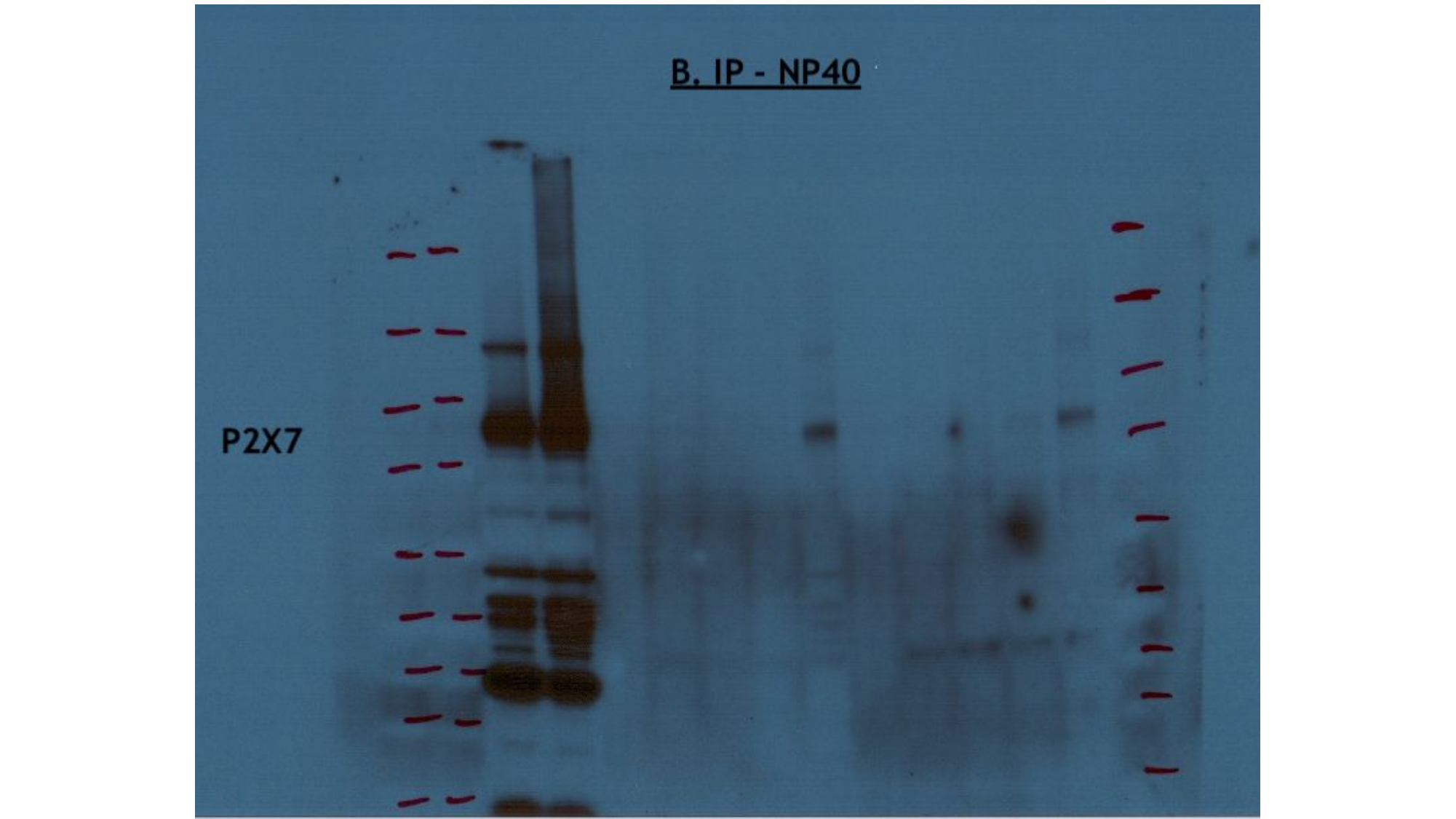

## Slide 4
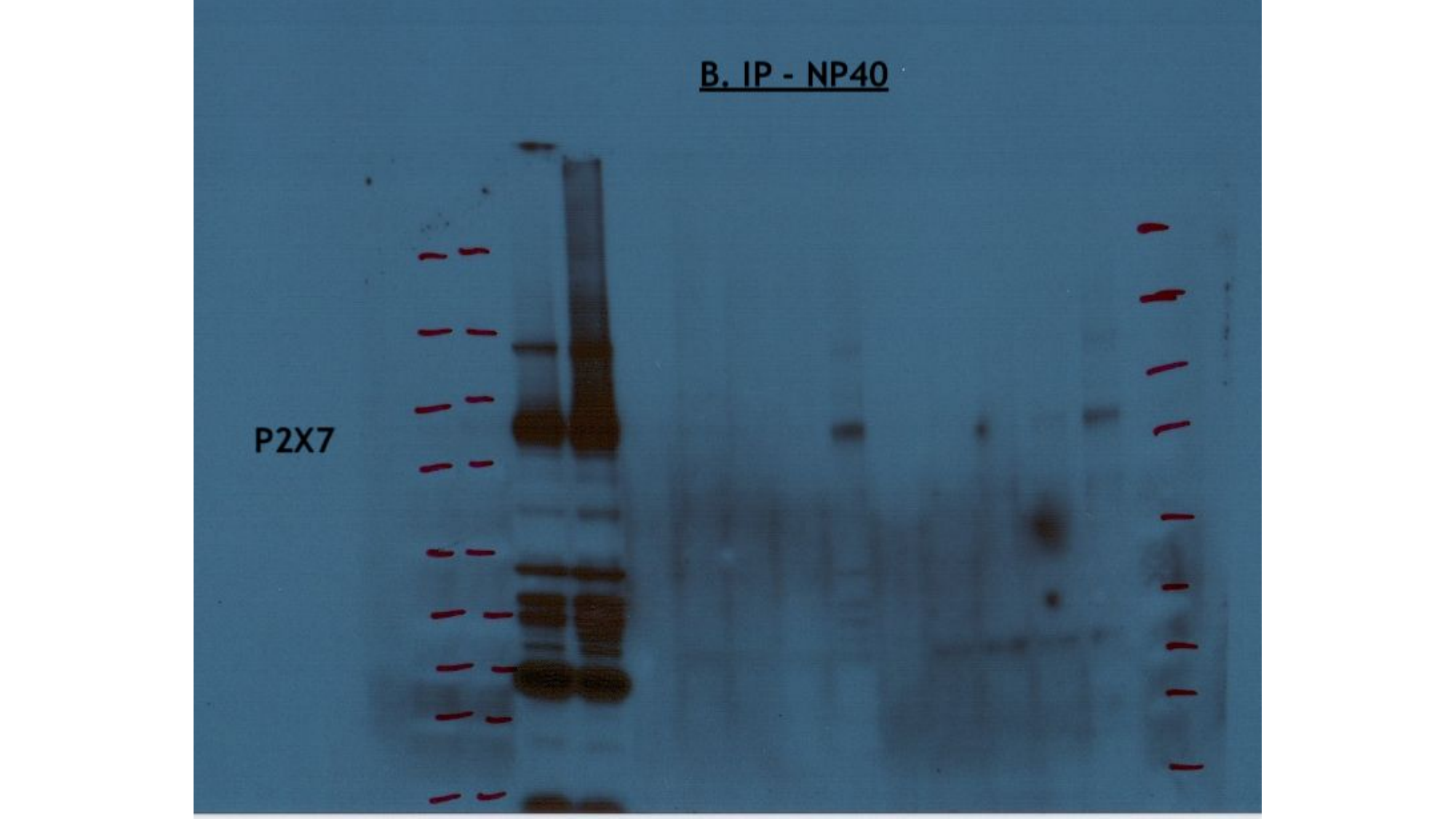

## Slide 5
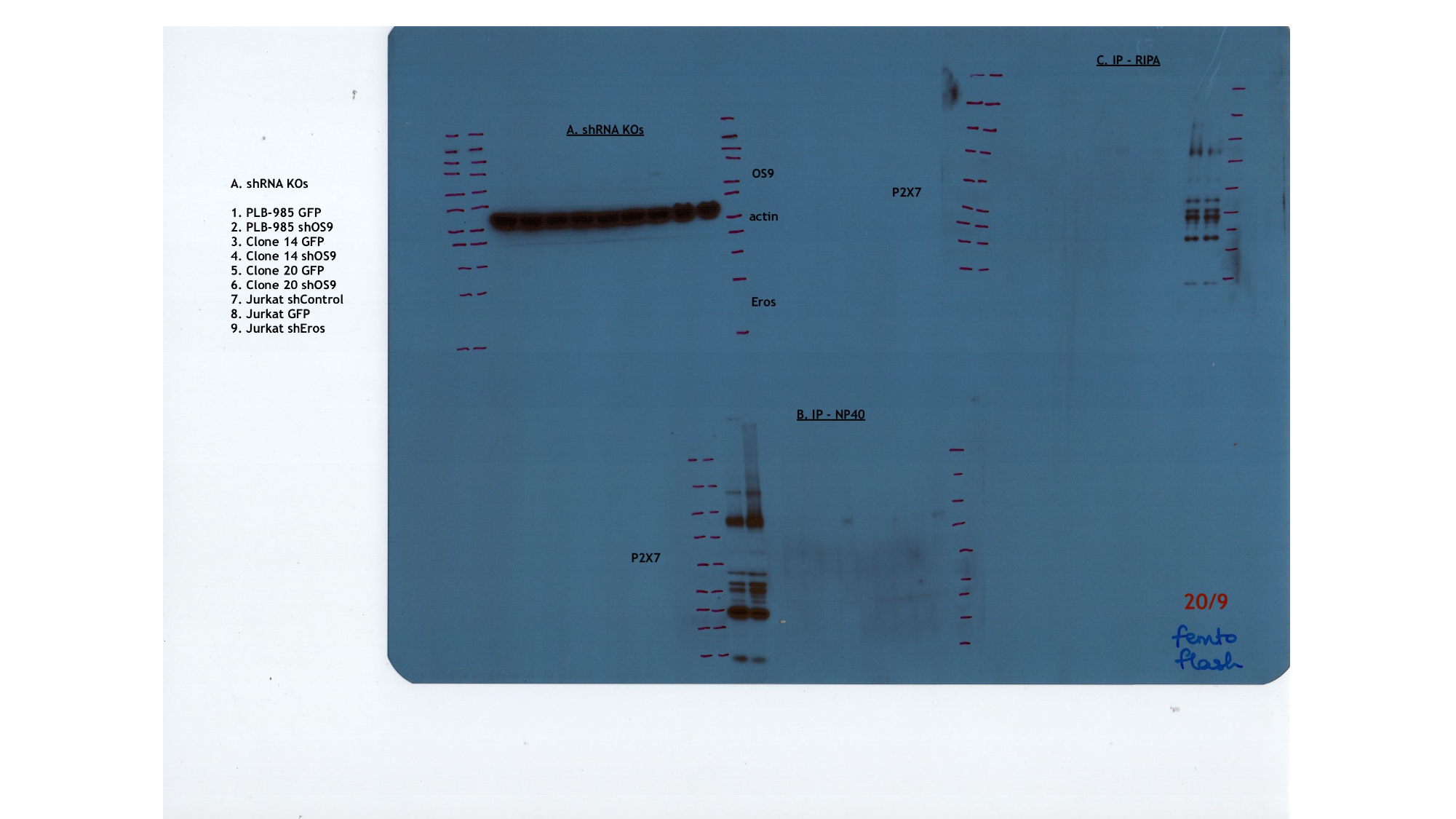

## Slide 6
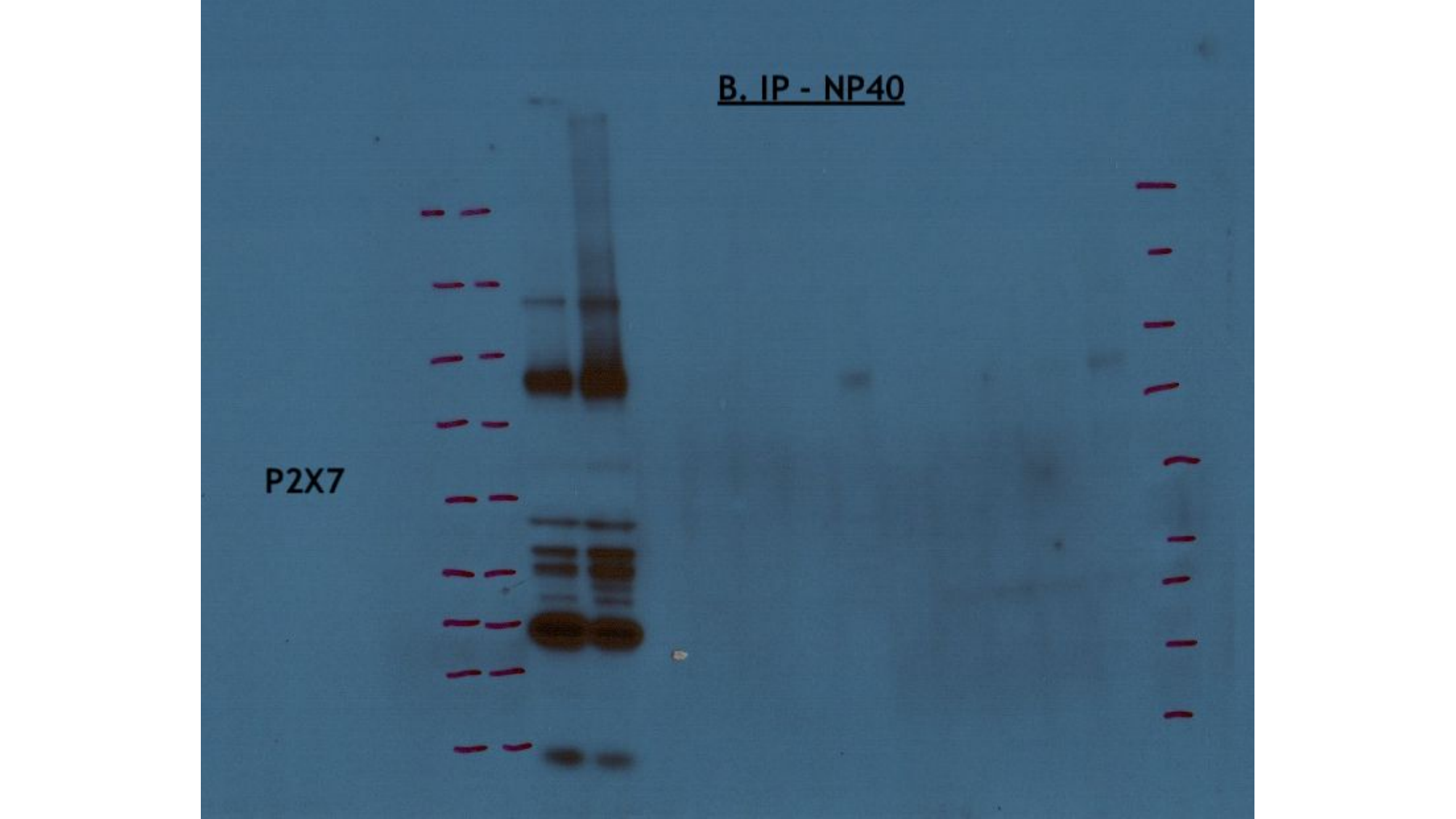

## Slide 7
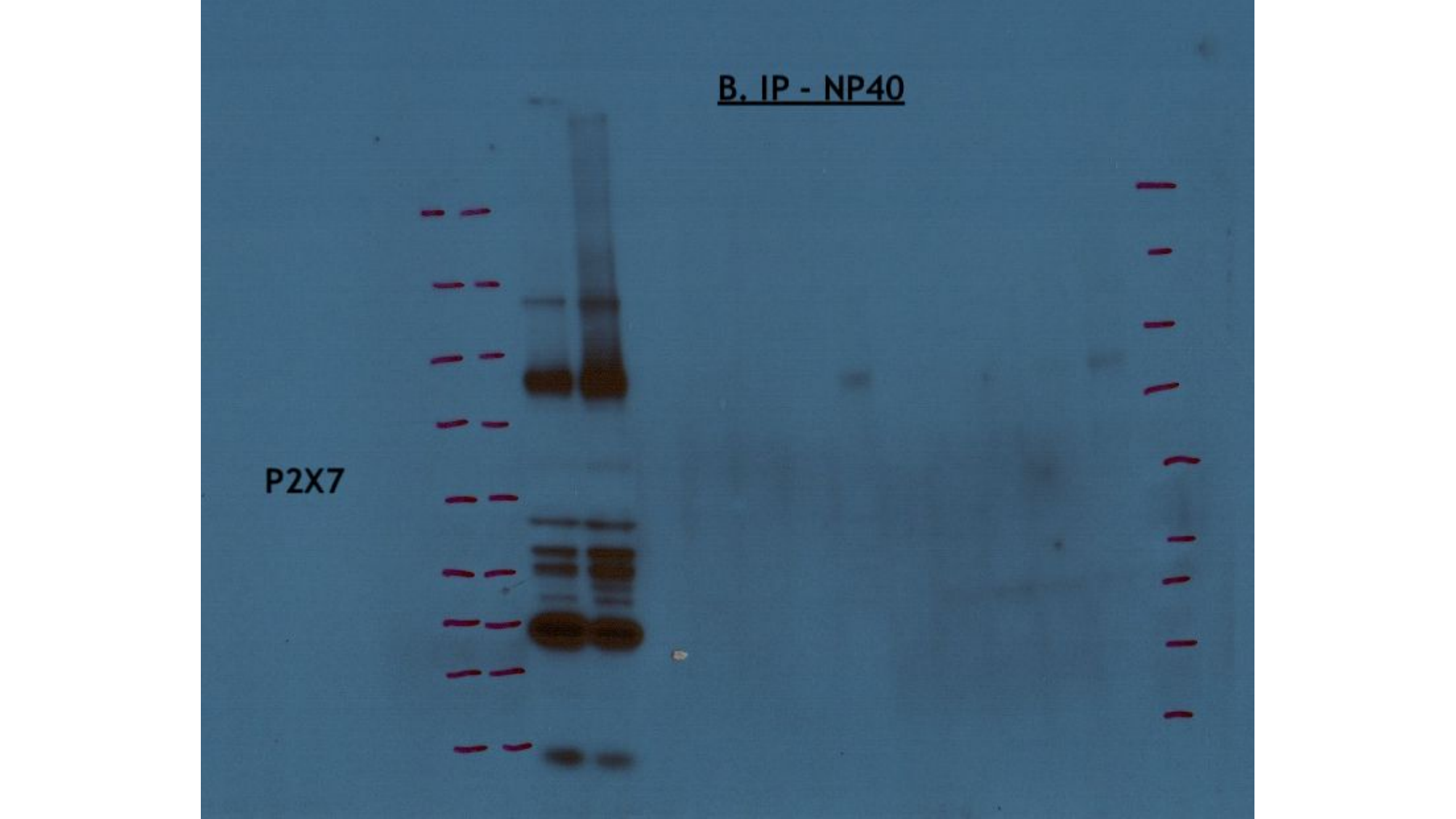

## Slide 8
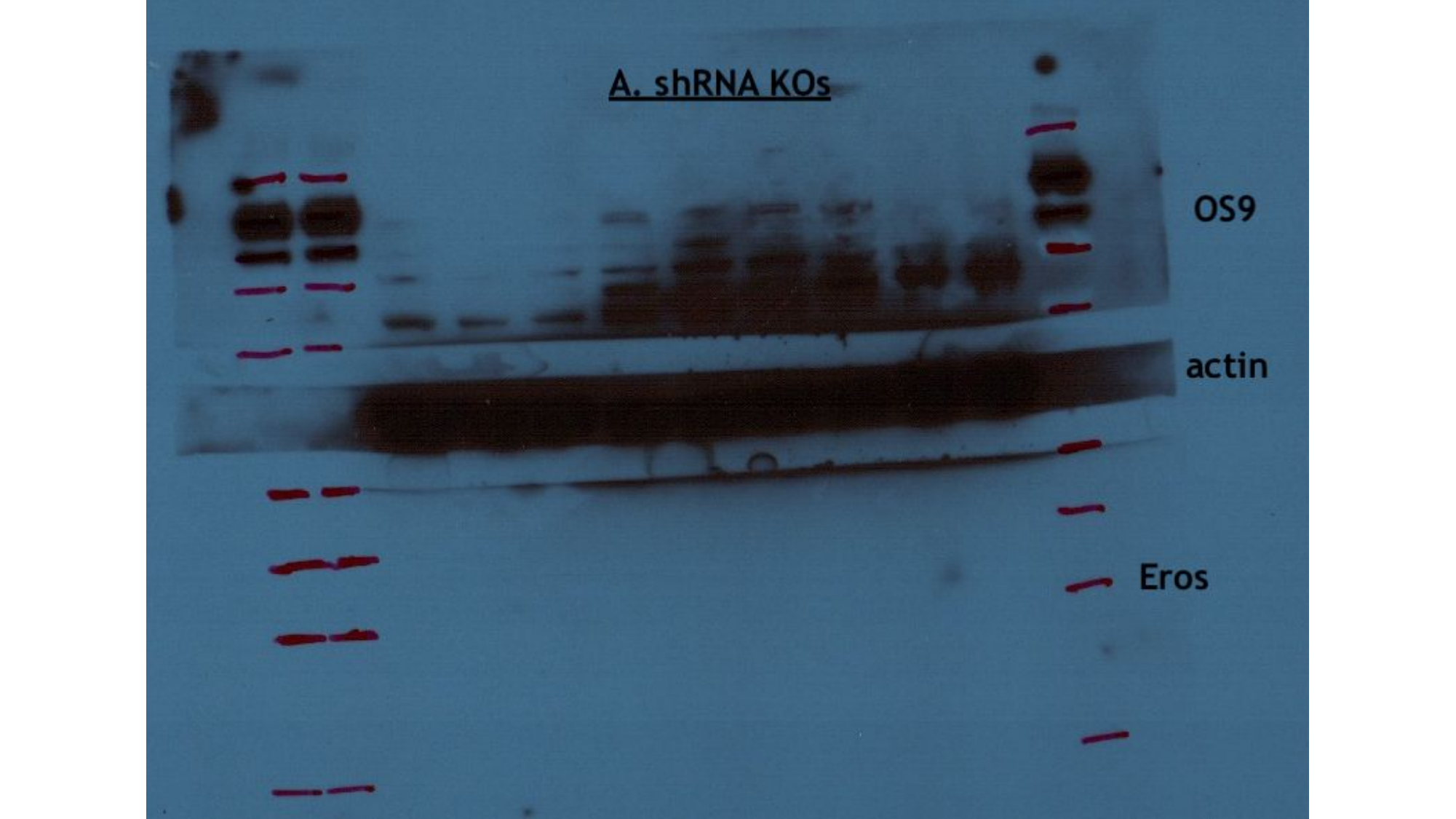

## Slide 9
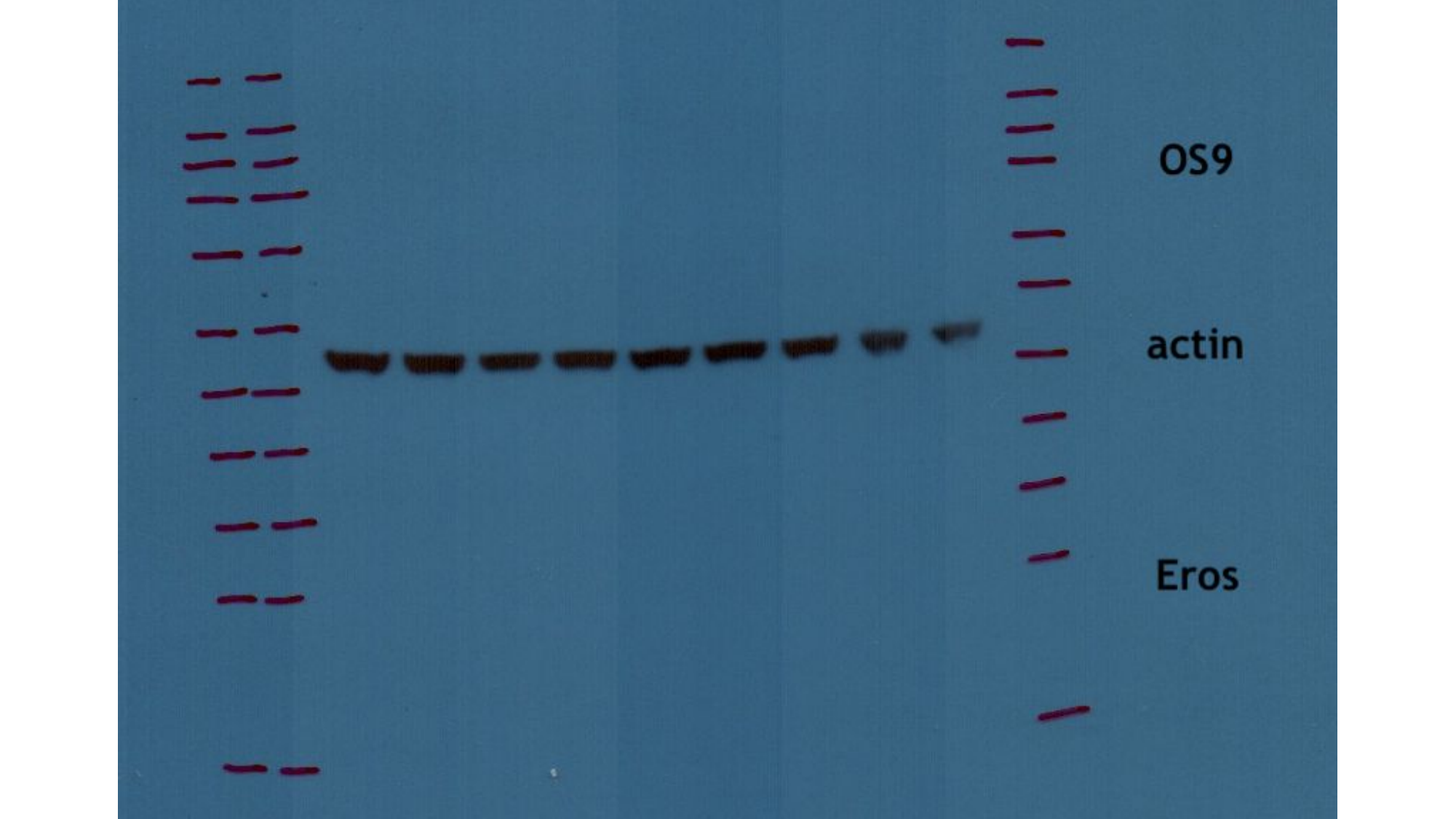

## Slide 10
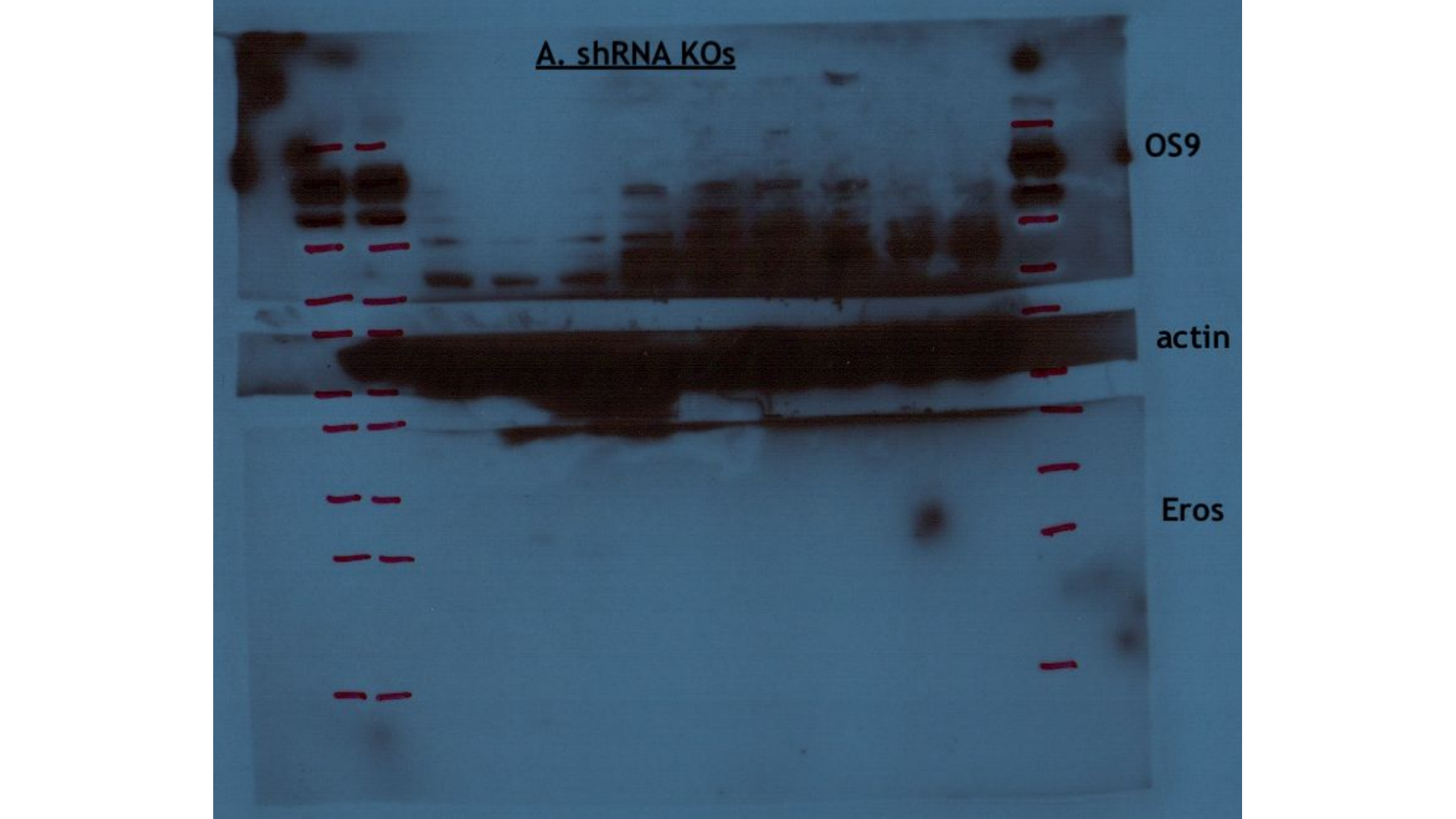

Supplement: Figure 5—source data 2. [file elife-76387-fig5-data2.zip › Figure 5- source data 2/P2X7 western pull down.pptx]

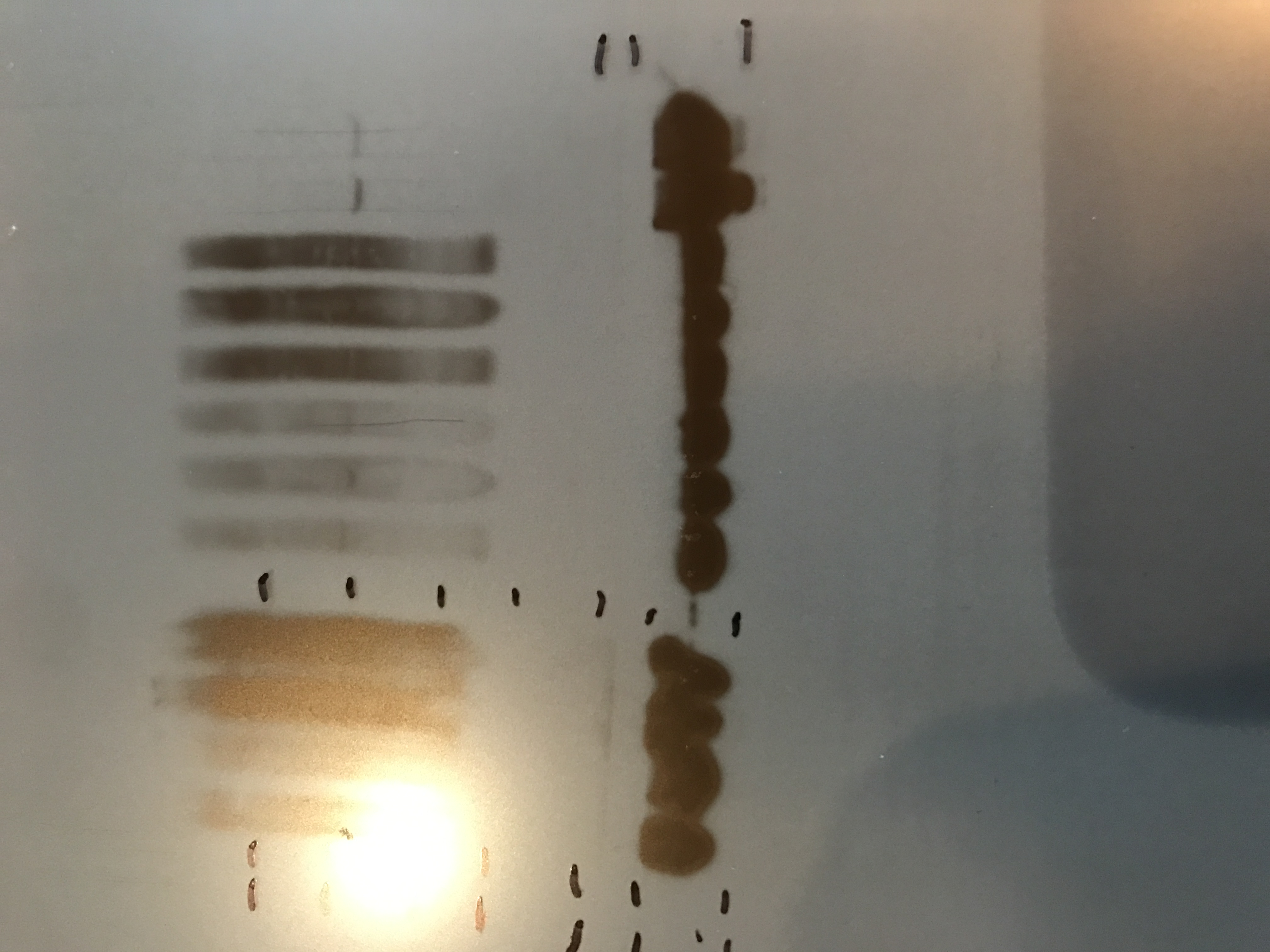

Supplement: Figure 5—source data 2. [file elife-76387-fig5-data2.zip › Figure 5- source data 2/Raw overexpression January 2019 P2X7.JPG]

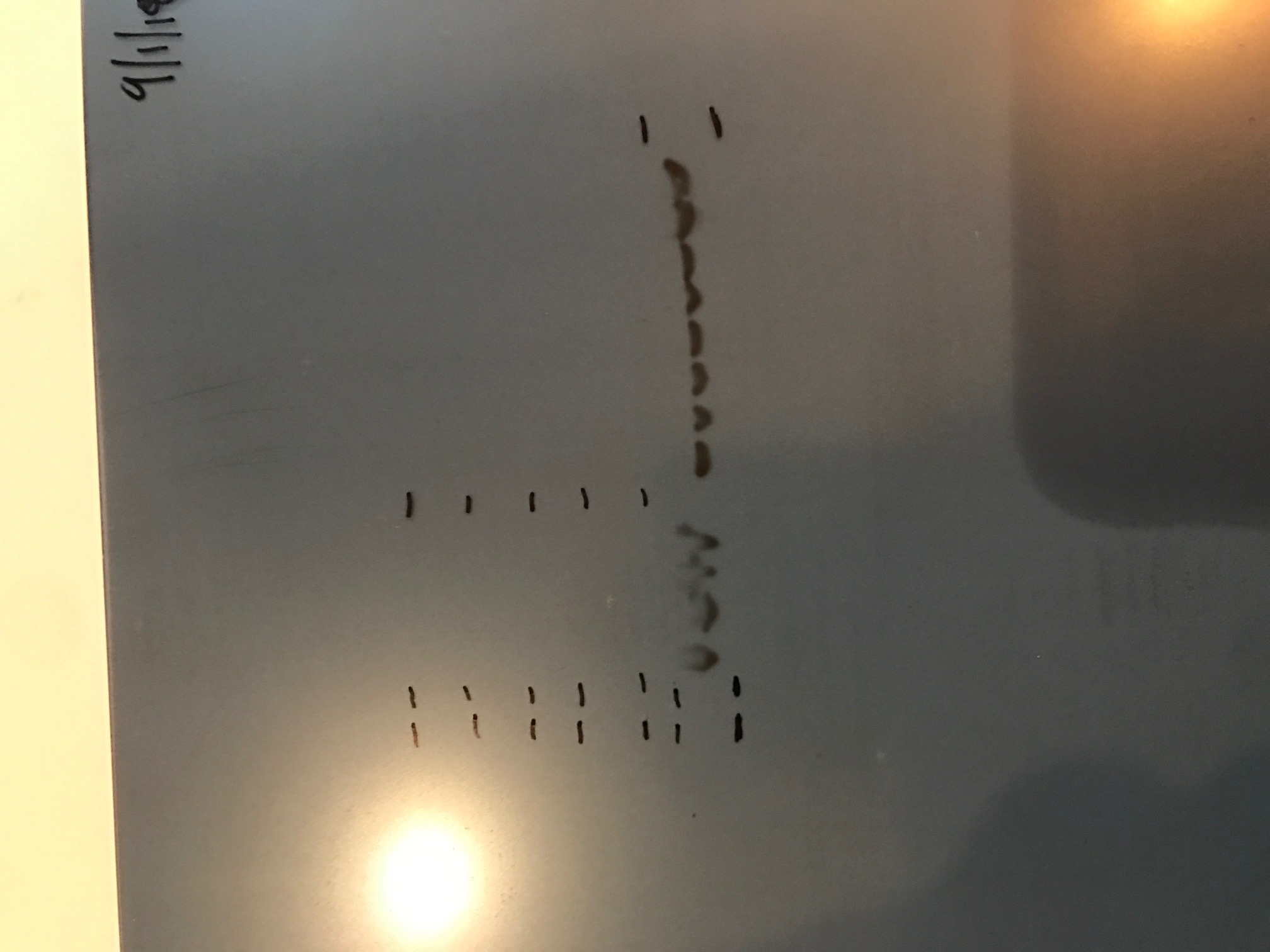

Supplement: Figure 5—source data 2. [file elife-76387-fig5-data2.zip › Figure 5- source data 2/Raw overexpression January 2019.JPG]

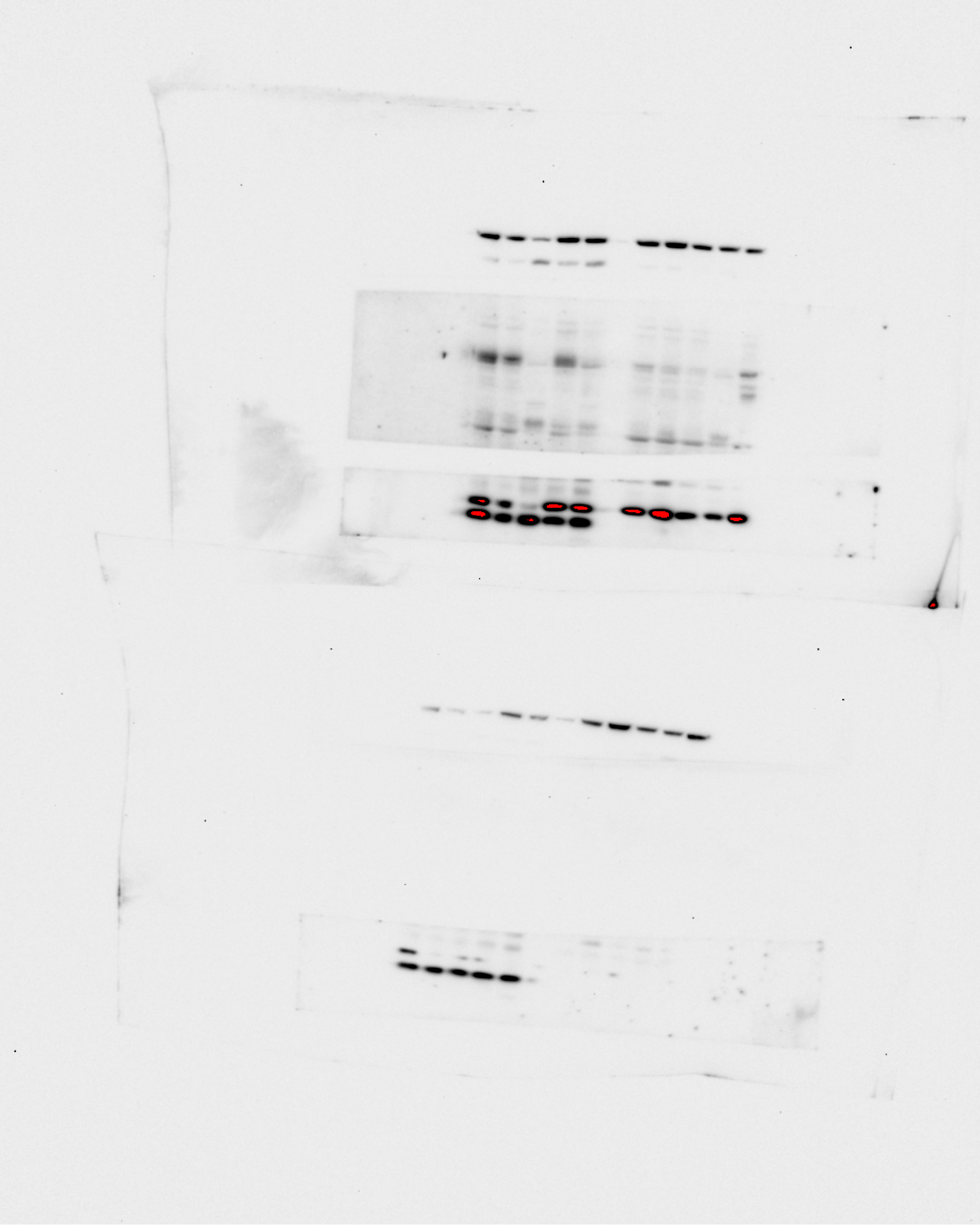

Supplement: Figure 5—source data 3. [file elife-76387-fig5-data3.zip › Figure 5- source data 3/2020-01-08 13h01m02s Chemiluminescence 313.332s BMDM P2X1 vinculin.tif]

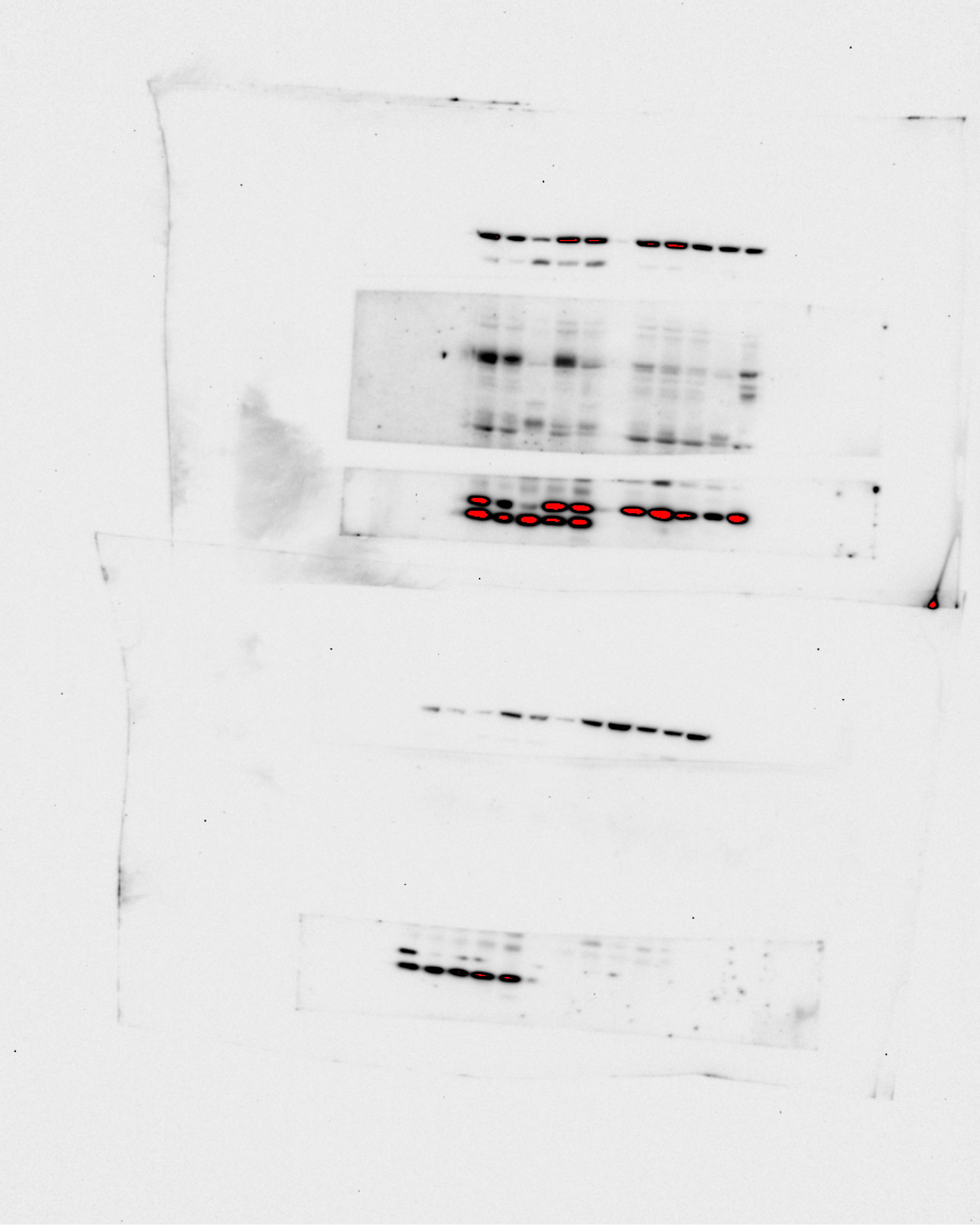

Supplement: Figure 5—source data 3. [file elife-76387-fig5-data3.zip › Figure 5- source data 3/2020-01-08 13h07m36s Chemiluminescence 693.330s BMDM P2X1 p2x1.tif]

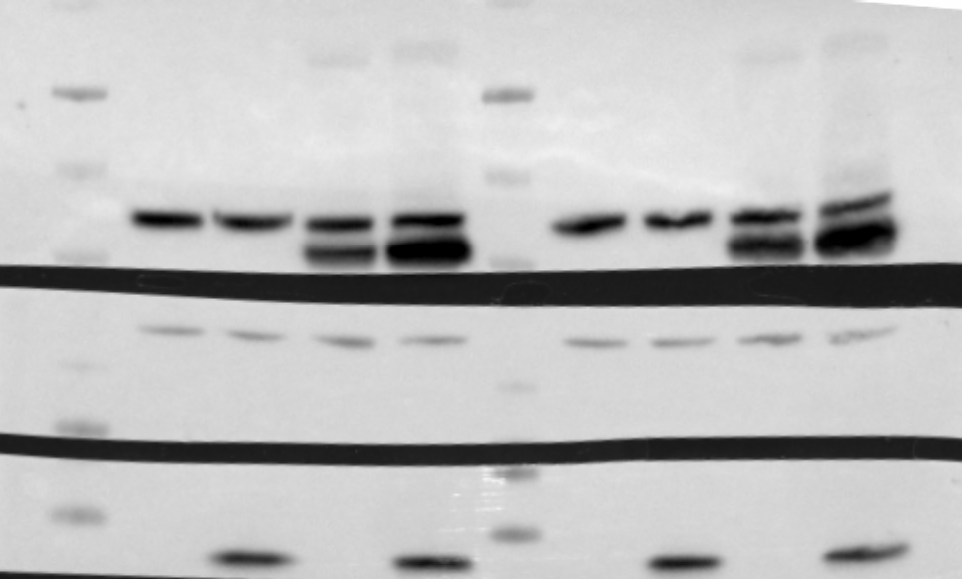

Supplement: Figure 5—source data 3. [file elife-76387-fig5-data3.zip › Figure 5- source data 3/20200623 p2x1 eros cotransfection pico 5 sec.tif]

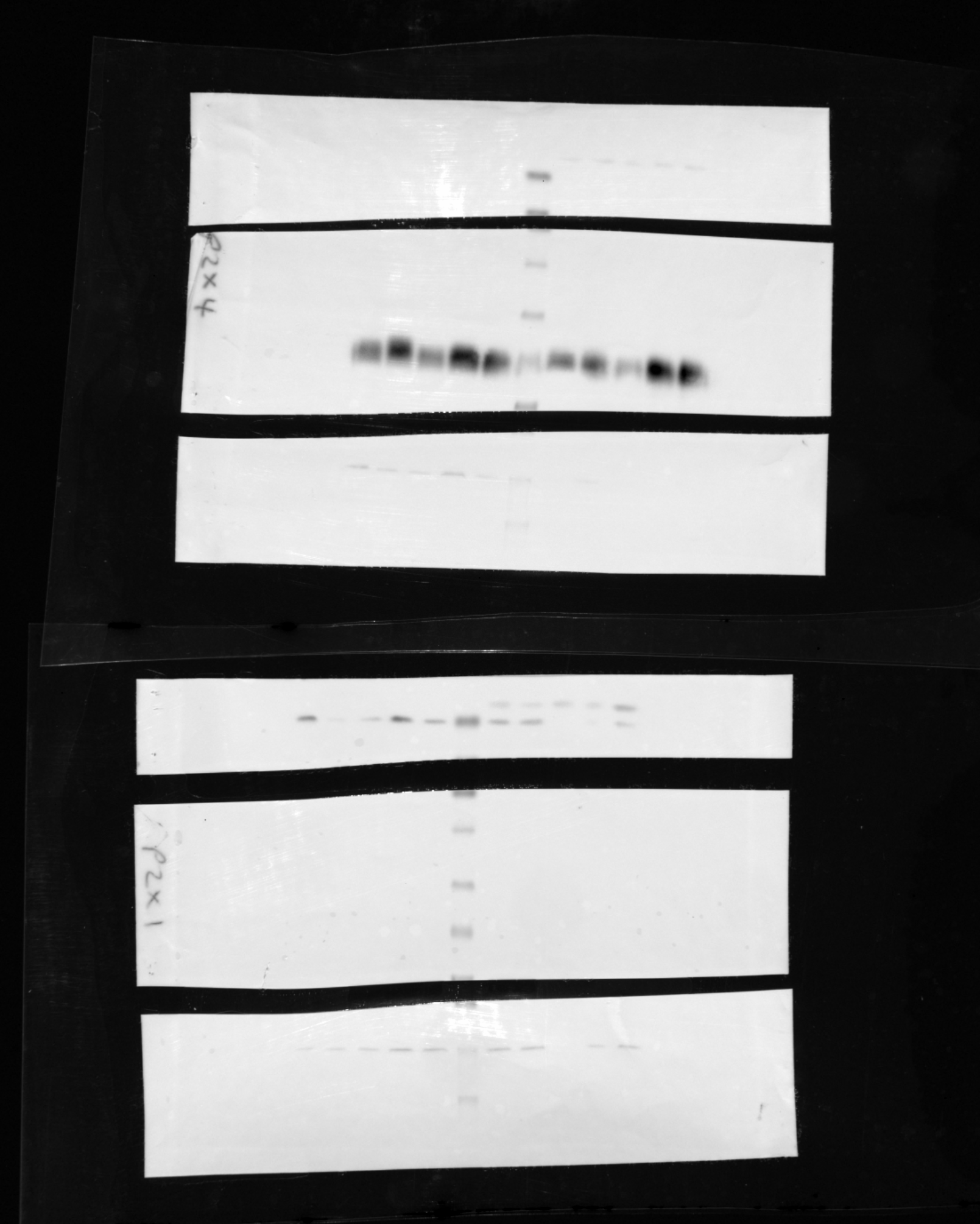

Supplement: Figure 5—figure supplement 1—source data 1. [file elife-76387-fig5-figsupp1-data1.zip › Figure 5 figure supplement 1- source data 1/2020-01-08 11h34m20s Chemiluminescence 60.000s+2020-01-08 11h32m37s Colorimetric 0.159s BMDM EROS KO P2X4 p2x4.tif]

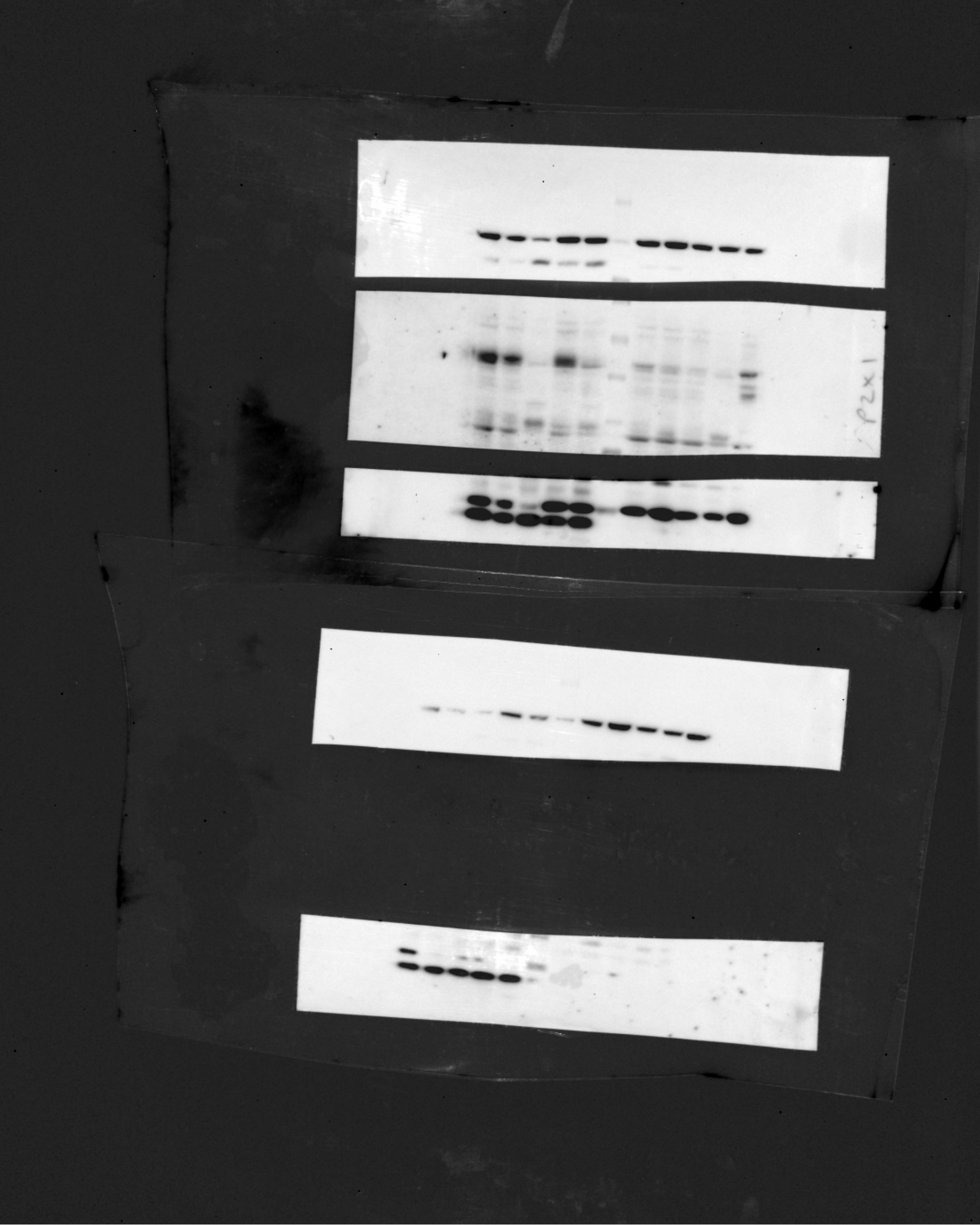

Supplement: Figure 5—figure supplement 1—source data 1. [file elife-76387-fig5-figsupp1-data1.zip › Figure 5 figure supplement 1- source data 1/2020-01-08 12h55m06s Colorimetric 0.140s+2020-01-08 13h07m36s Chemiluminescence 693.330s BMDM EROS KO P2X4 vinculin.tif]

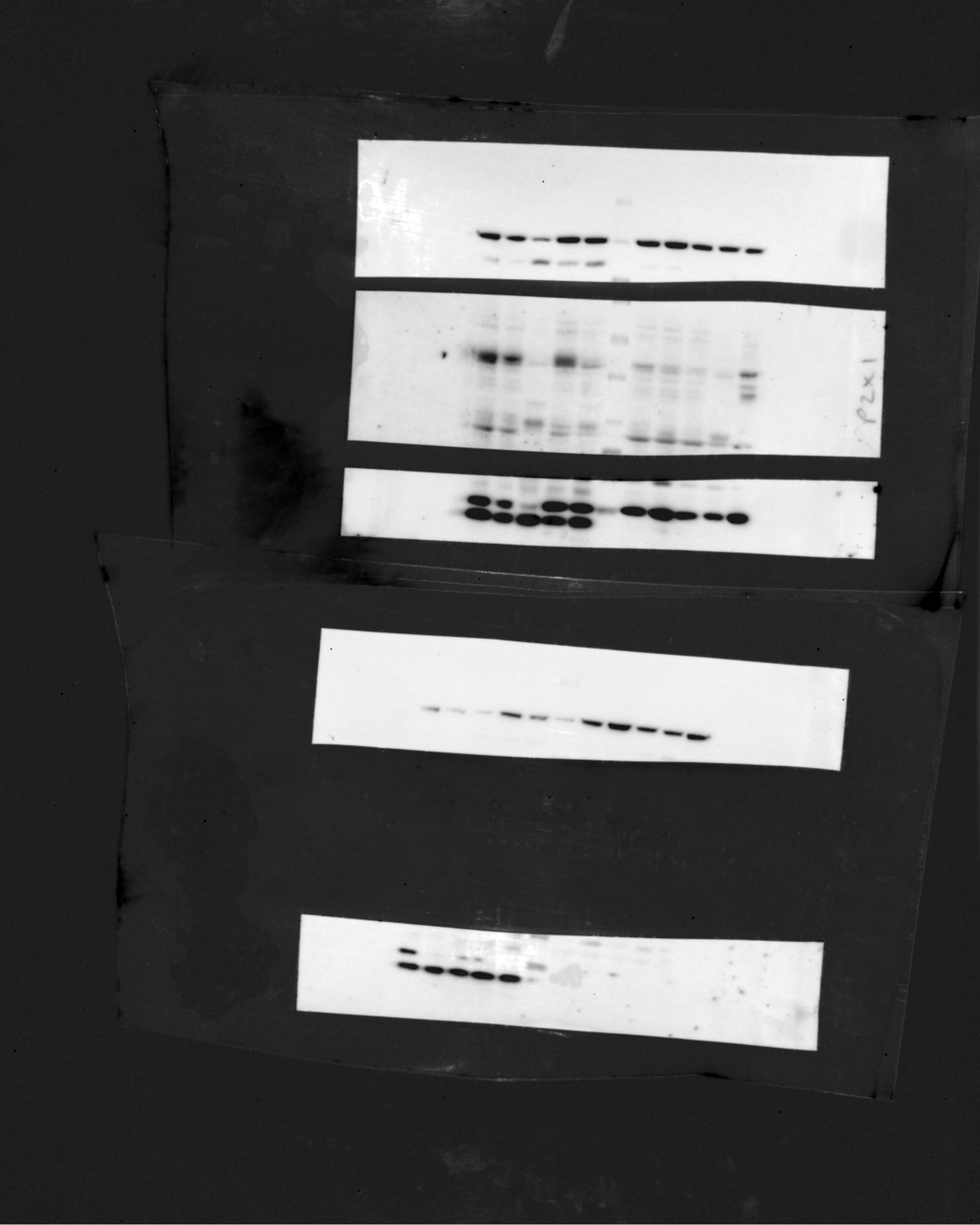

Supplement: Figure 5—figure supplement 1—source data 1. [file elife-76387-fig5-figsupp1-data1.zip › Figure 5 figure supplement 1- source data 1/2020-01-08 13h05m25s Chemiluminescence 566.664s+2020-01-08 12h55m06s Colorimetric 0.140s BMDM EROS KO P2X4 eros.tif]
