# Supplementary material for: EROS is a selective chaperone regulating the phagocyte NADPH oxidase and purinergic signalling
Source: eLife. 2022 Nov 24;11:e76387. doi: 10.7554/eLife.76387 (PMC9767466; doi:10.7554/eLife.76387)
Supplement: Figure 1—figure supplement 1—source data 3. [file elife-76387-fig1-figsupp1-data3.zip › Figure 1 figure supplement 1 - source data 3.pptx]

## Slide 1
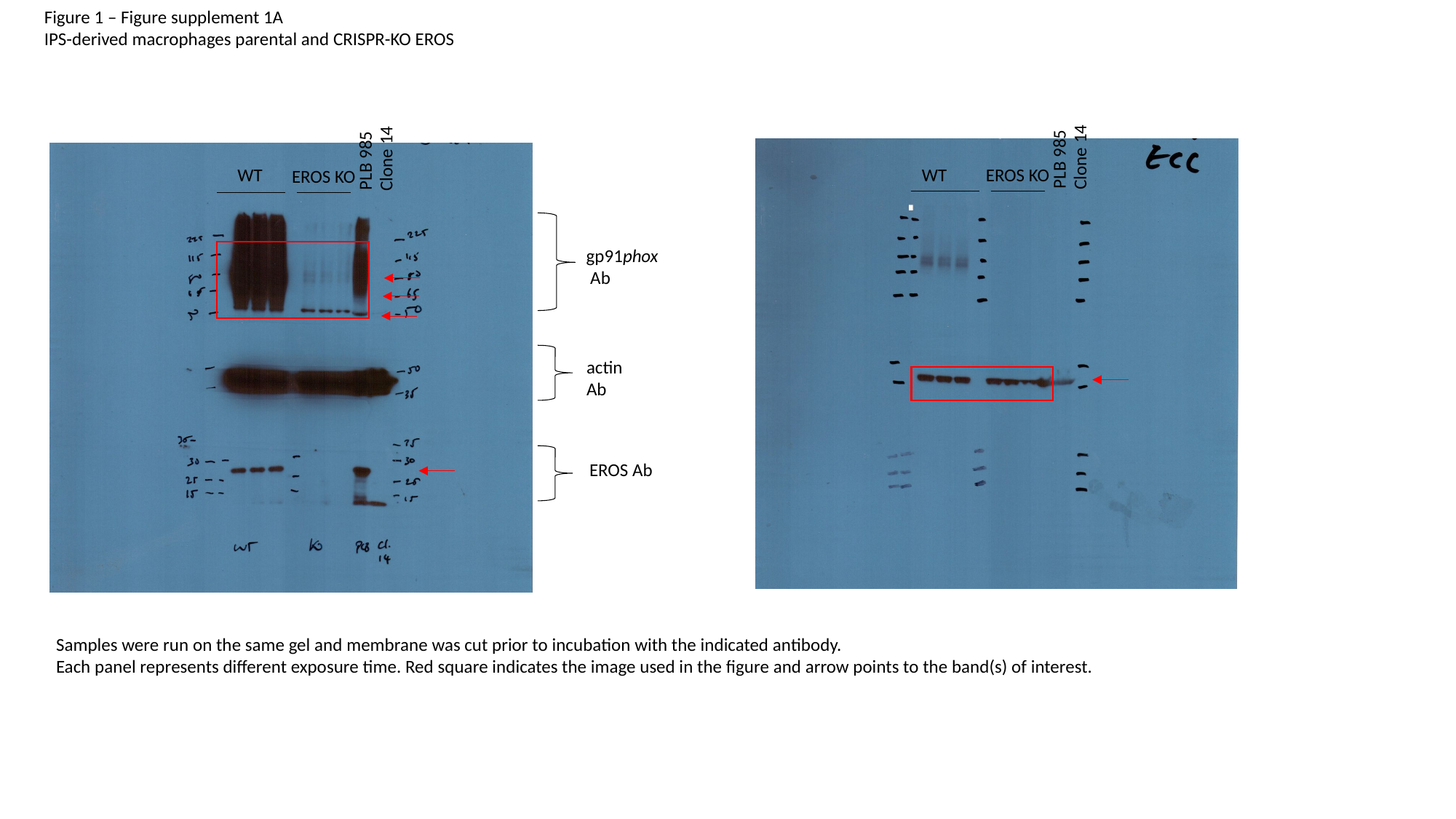

Figure 1 – Figure supplement 1A
IPS-derived macrophages parental and CRISPR-KO EROS
PLB 985
Clone 14
PLB 985
Clone 14
WT
WT
EROS KO
EROS KO
gp91phox
 Ab
actin
Ab
EROS Ab
Samples were run on the same gel and membrane was cut prior to incubation with the indicated antibody.
Each panel represents different exposure time. Red square indicates the image used in the figure and arrow points to the band(s) of interest.

## Slide 2
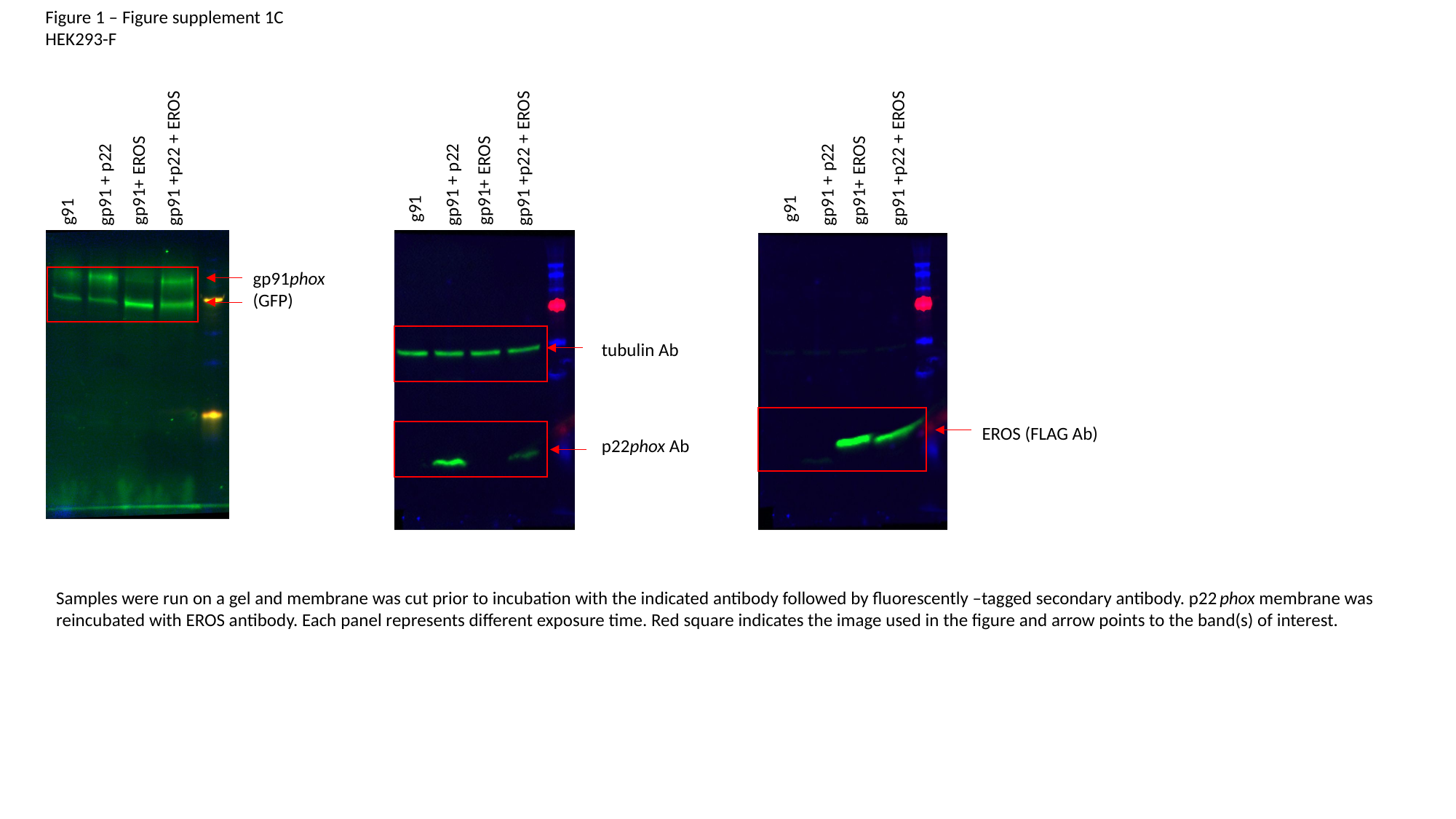

Figure 1 – Figure supplement 1C
HEK293-F
gp91 + p22
gp91 +p22 + EROS
gp91 +p22 + EROS
gp91 + p22
gp91 +p22 + EROS
gp91 + p22
gp91+ EROS
gp91+ EROS
gp91+ EROS
g91
g91
g91
gp91phox
(GFP)
tubulin Ab
EROS (FLAG Ab)
p22phox Ab
Samples were run on a gel and membrane was cut prior to incubation with the indicated antibody followed by fluorescently –tagged secondary antibody. p22phox membrane was reincubated with EROS antibody. Each panel represents different exposure time. Red square indicates the image used in the figure and arrow points to the band(s) of interest.

## Slide 3
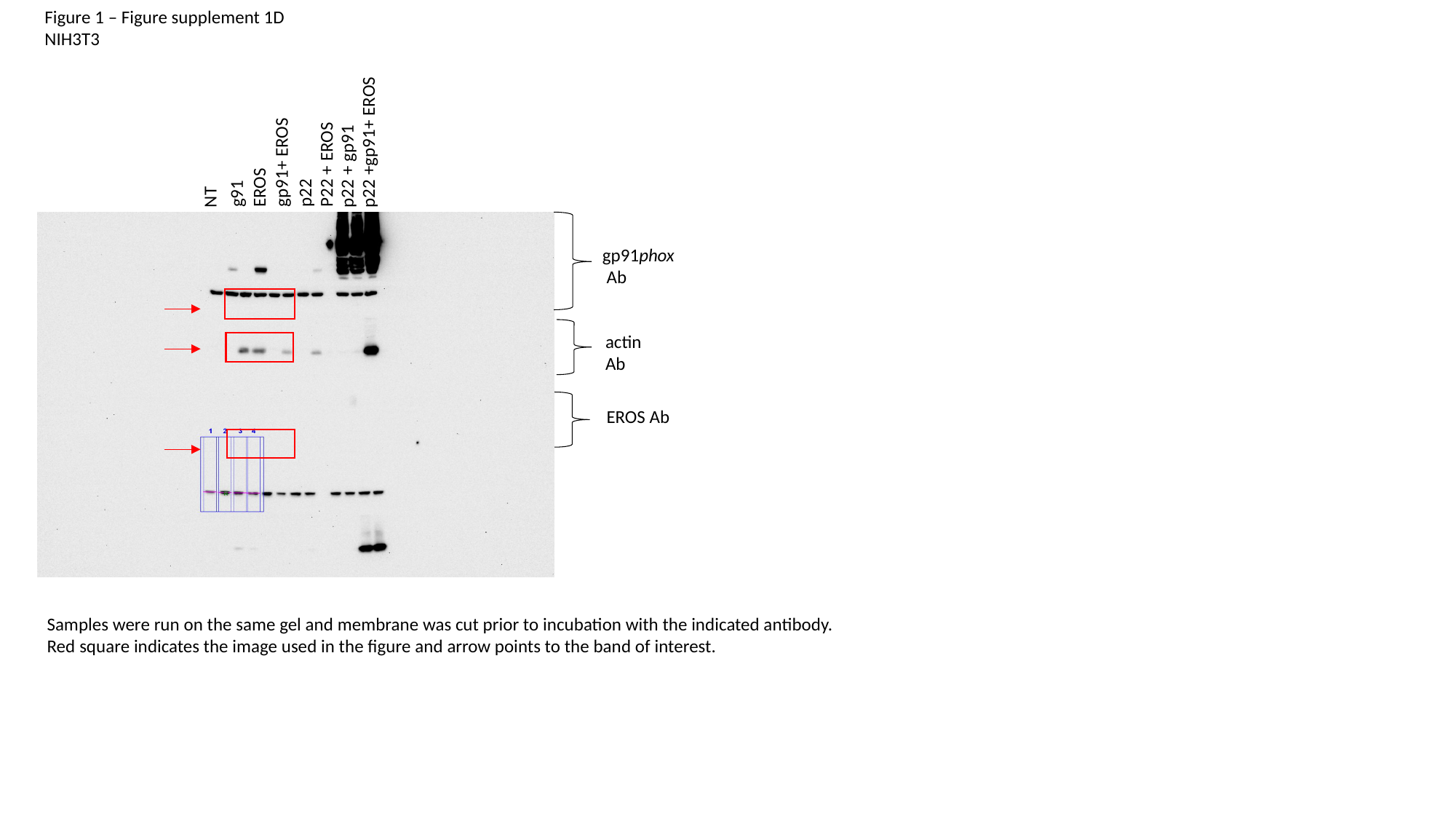

Figure 1 – Figure supplement 1D
NIH3T3
p22 + gp91
p22 +gp91+ EROS
gp91+ EROS
P22 + EROS
EROS
p22
g91
NT
gp91phox
 Ab
actin
Ab
EROS Ab
Samples were run on the same gel and membrane was cut prior to incubation with the indicated antibody.
Red square indicates the image used in the figure and arrow points to the band of interest.

## Slide 4
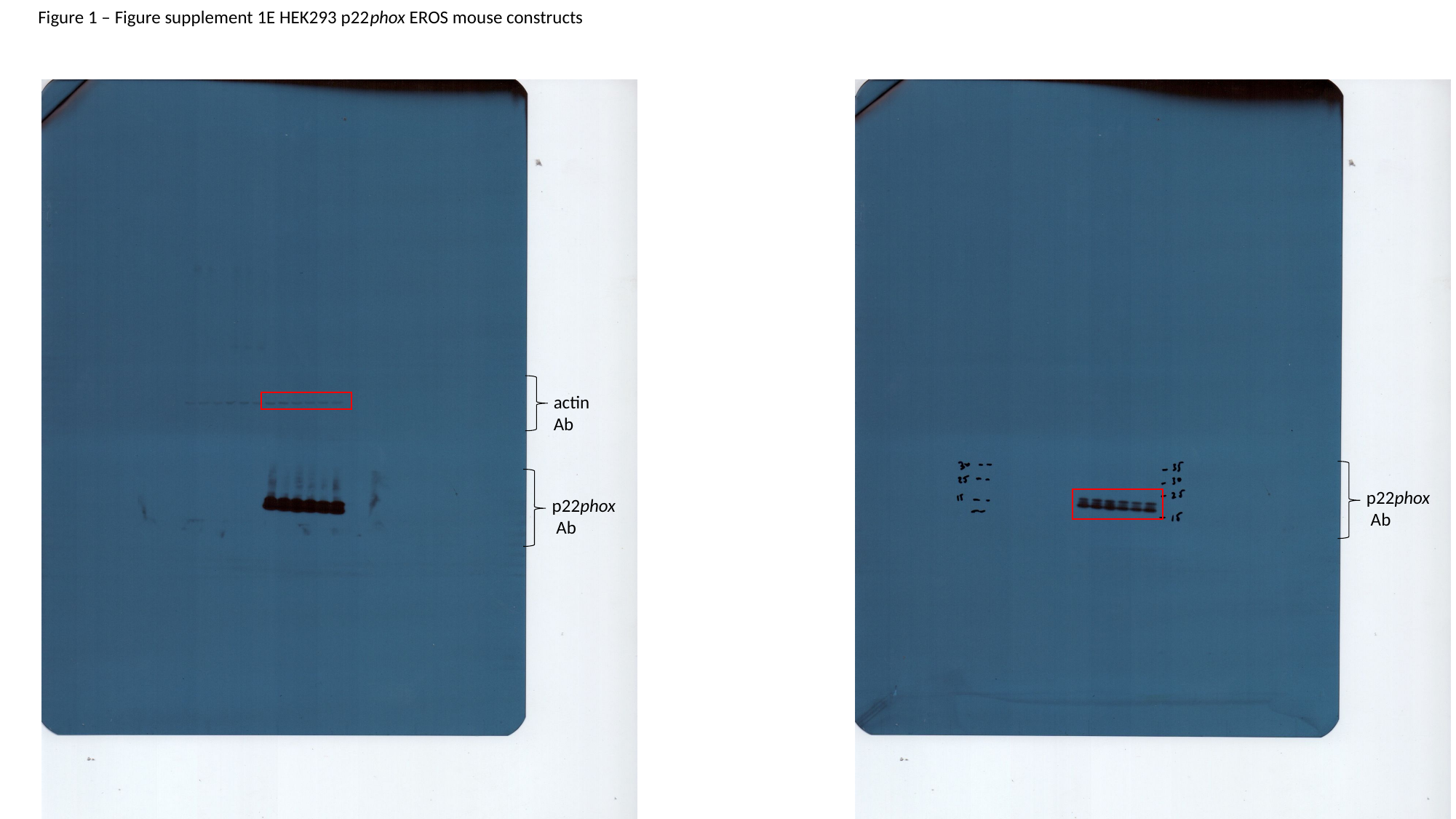

Figure 1 – Figure supplement 1E HEK293 p22phox EROS mouse constructs
actin
Ab
p22phox
 Ab
p22phox
 Ab

## Slide 5
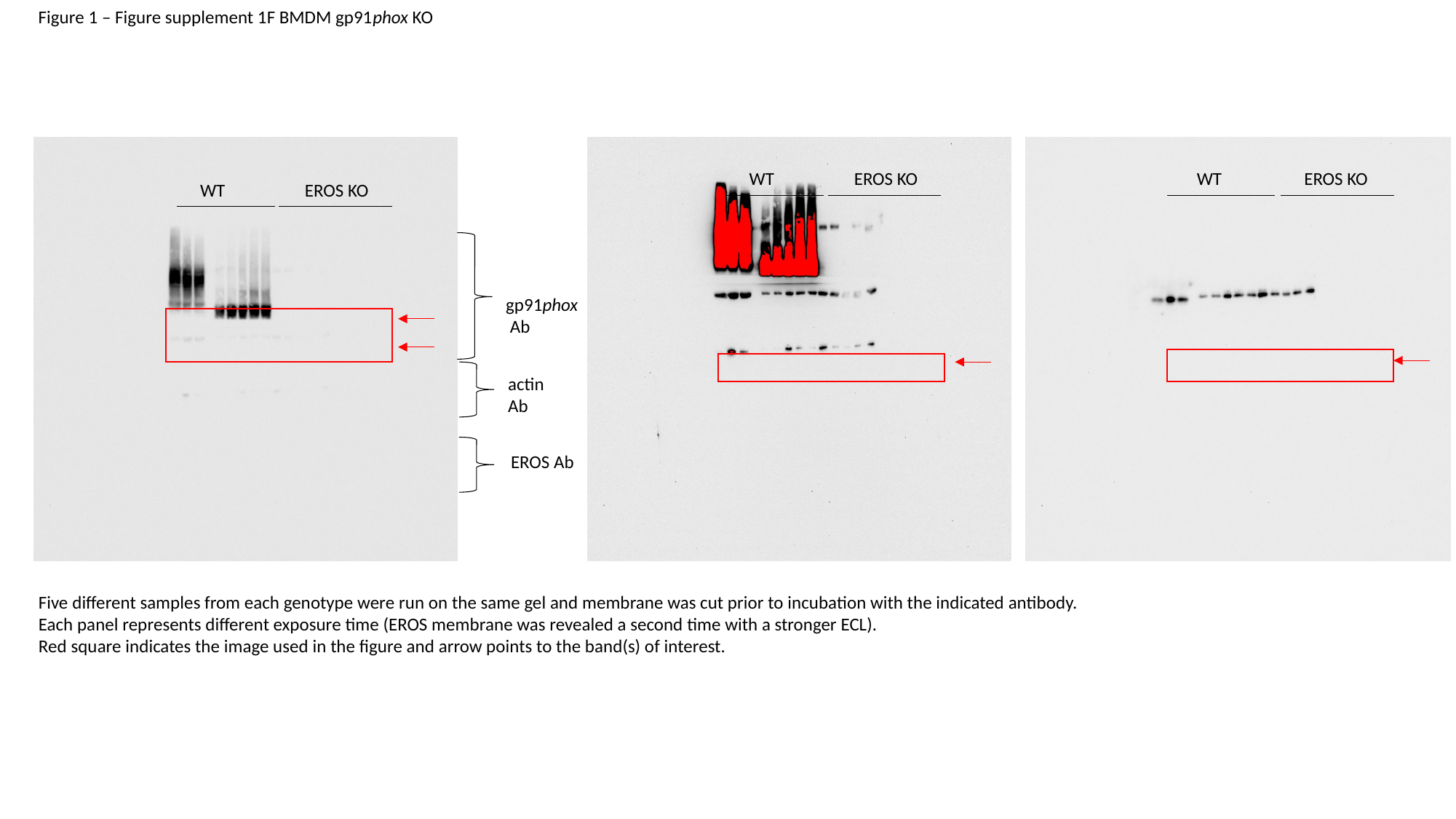

Figure 1 – Figure supplement 1F BMDM gp91phox KO
WT
EROS KO
WT
EROS KO
WT
EROS KO
gp91phox
 Ab
actin
Ab
EROS Ab
Five different samples from each genotype were run on the same gel and membrane was cut prior to incubation with the indicated antibody.
Each panel represents different exposure time (EROS membrane was revealed a second time with a stronger ECL).
Red square indicates the image used in the figure and arrow points to the band(s) of interest.
