# Supplementary material for: EROS is a selective chaperone regulating the phagocyte NADPH oxidase and purinergic signalling
Source: eLife. 2022 Nov 24;11:e76387. doi: 10.7554/eLife.76387 (PMC9767466; doi:10.7554/eLife.76387)
Supplement: Figure 2—source data 2. [file elife-76387-fig2-data2.zip › Figure 2- source data 2.pptx]

## Slide 1
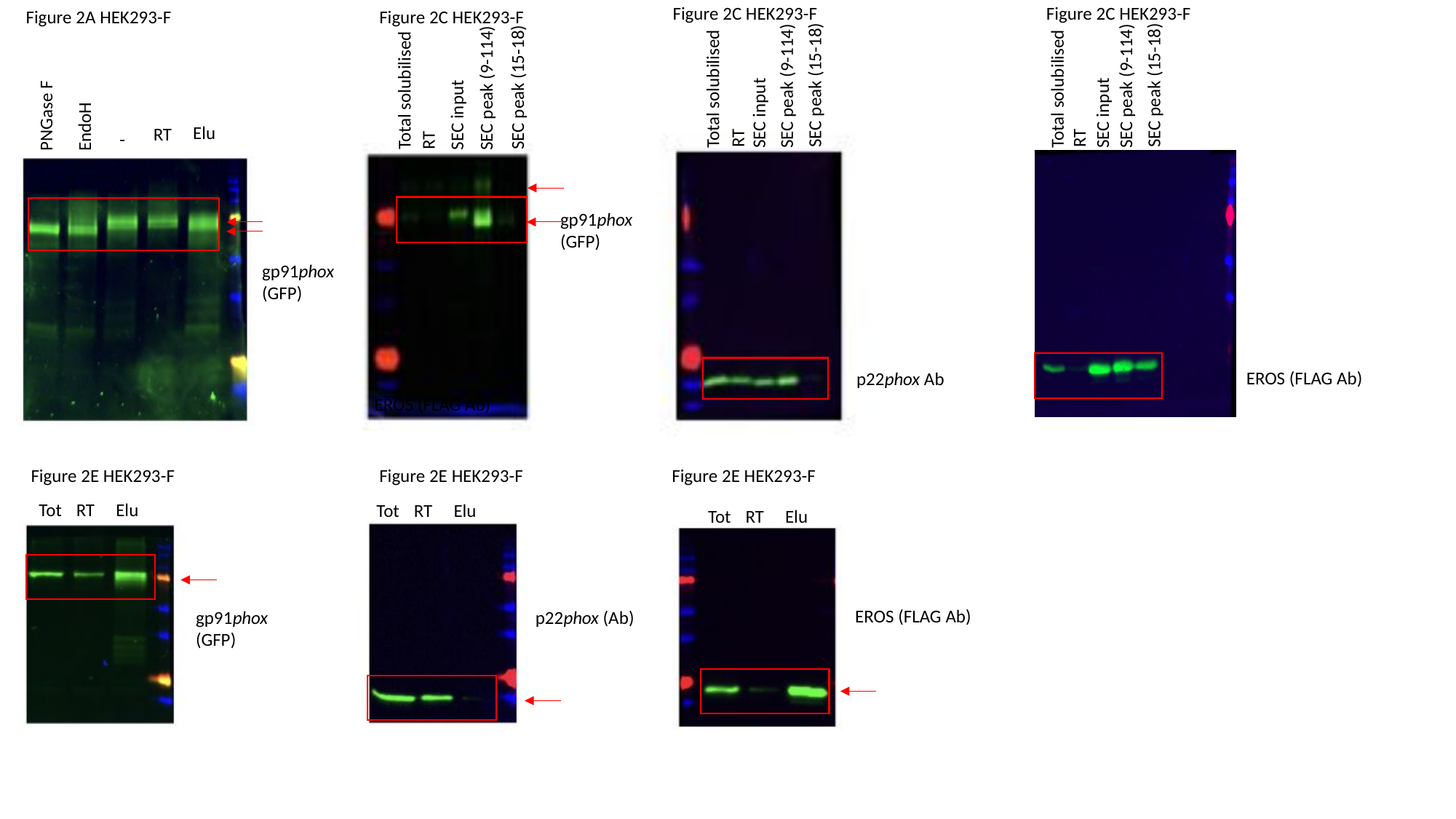

Figure 2C HEK293-F
Figure 2C HEK293-F
Figure 2C HEK293-F
Figure 2A HEK293-F
Total solubilised
Total solubilised
SEC peak (9-114)
SEC peak (9-114)
SEC peak (9-114)
SEC peak (15-18)
SEC peak (15-18)
SEC peak (15-18)
Total solubilised
SEC input
SEC input
SEC input
PNGase F
EndoH
Elu
RT
RT
RT
-
RT
gp91phox
(GFP)
gp91phox
(GFP)
EROS (FLAG Ab)
p22phox Ab
EROS (FLAG Ab)
Figure 2E HEK293-F
Figure 2E HEK293-F
Figure 2E HEK293-F
Tot
RT
Elu
Tot
RT
Elu
Tot
RT
Elu
EROS (FLAG Ab)
p22phox (Ab)
gp91phox
(GFP)
