# Supplementary material for: EROS is a selective chaperone regulating the phagocyte NADPH oxidase and purinergic signalling
Source: eLife. 2022 Nov 24;11:e76387. doi: 10.7554/eLife.76387 (PMC9767466; doi:10.7554/eLife.76387)
Supplement: Figure 2—figure supplement 1—source data 5. [file elife-76387-fig2-figsupp1-data5.zip › Figure 2 figure supplement 1- source data 5.pptx]

## Slide 1
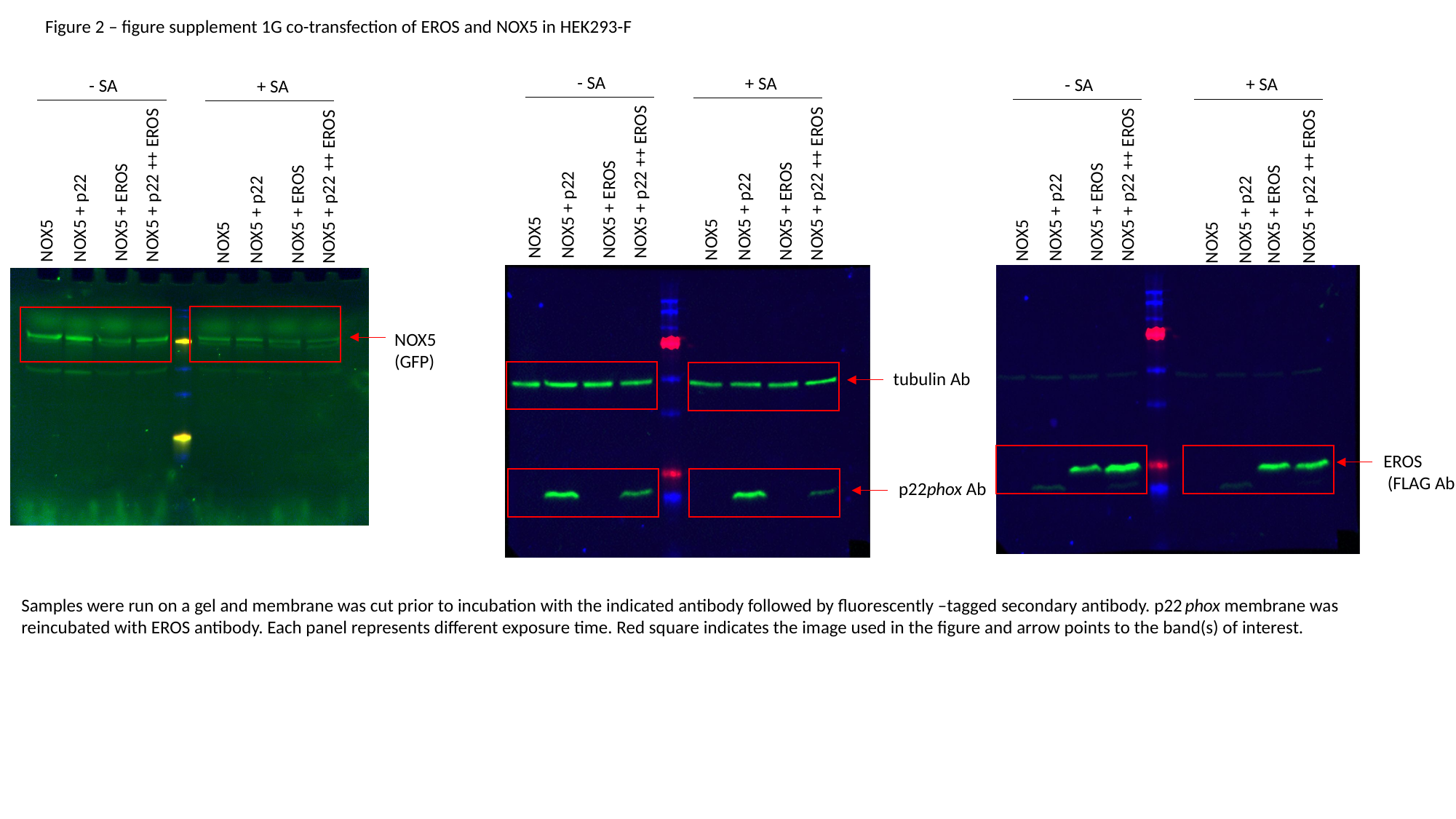

Figure 2 – figure supplement 1G co-transfection of EROS and NOX5 in HEK293-F
- SA
+ SA
+ SA
- SA
- SA
+ SA
NOX5
NOX5 + p22
NOX5 + p22 ++ EROS
NOX5
NOX5 + p22
NOX5 + p22 ++ EROS
NOX5
NOX5 + p22
NOX5 + p22 ++ EROS
NOX5
NOX5 + p22
NOX5 + p22 ++ EROS
NOX5
NOX5 + p22
NOX5 + p22 ++ EROS
NOX5
NOX5 + p22
NOX5 + p22 ++ EROS
NOX5 + EROS
NOX5 + EROS
NOX5 + EROS
NOX5 + EROS
NOX5 + EROS
NOX5 + EROS
NOX5
(GFP)
tubulin Ab
EROS
 (FLAG Ab)
p22phox Ab
Samples were run on a gel and membrane was cut prior to incubation with the indicated antibody followed by fluorescently –tagged secondary antibody. p22phox membrane was reincubated with EROS antibody. Each panel represents different exposure time. Red square indicates the image used in the figure and arrow points to the band(s) of interest.

## Slide 2
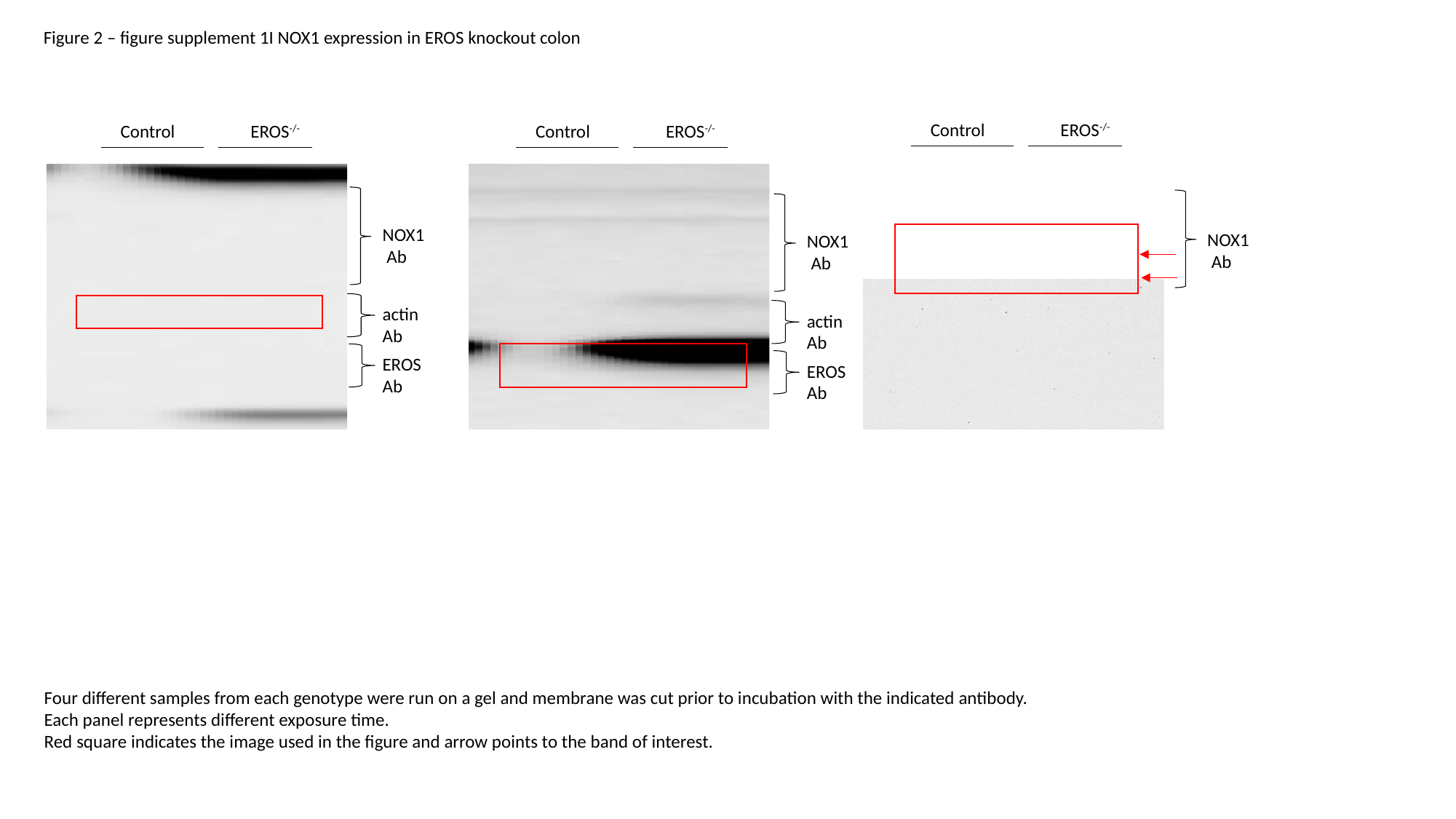

Figure 2 – figure supplement 1I NOX1 expression in EROS knockout colon
EROS-/-
Control
EROS-/-
EROS-/-
Control
Control
NOX1
 Ab
NOX1
 Ab
NOX1
 Ab
actin
Ab
actin
Ab
EROS
Ab
EROS
Ab
Four different samples from each genotype were run on a gel and membrane was cut prior to incubation with the indicated antibody.
Each panel represents different exposure time.
Red square indicates the image used in the figure and arrow points to the band of interest.

## Slide 3
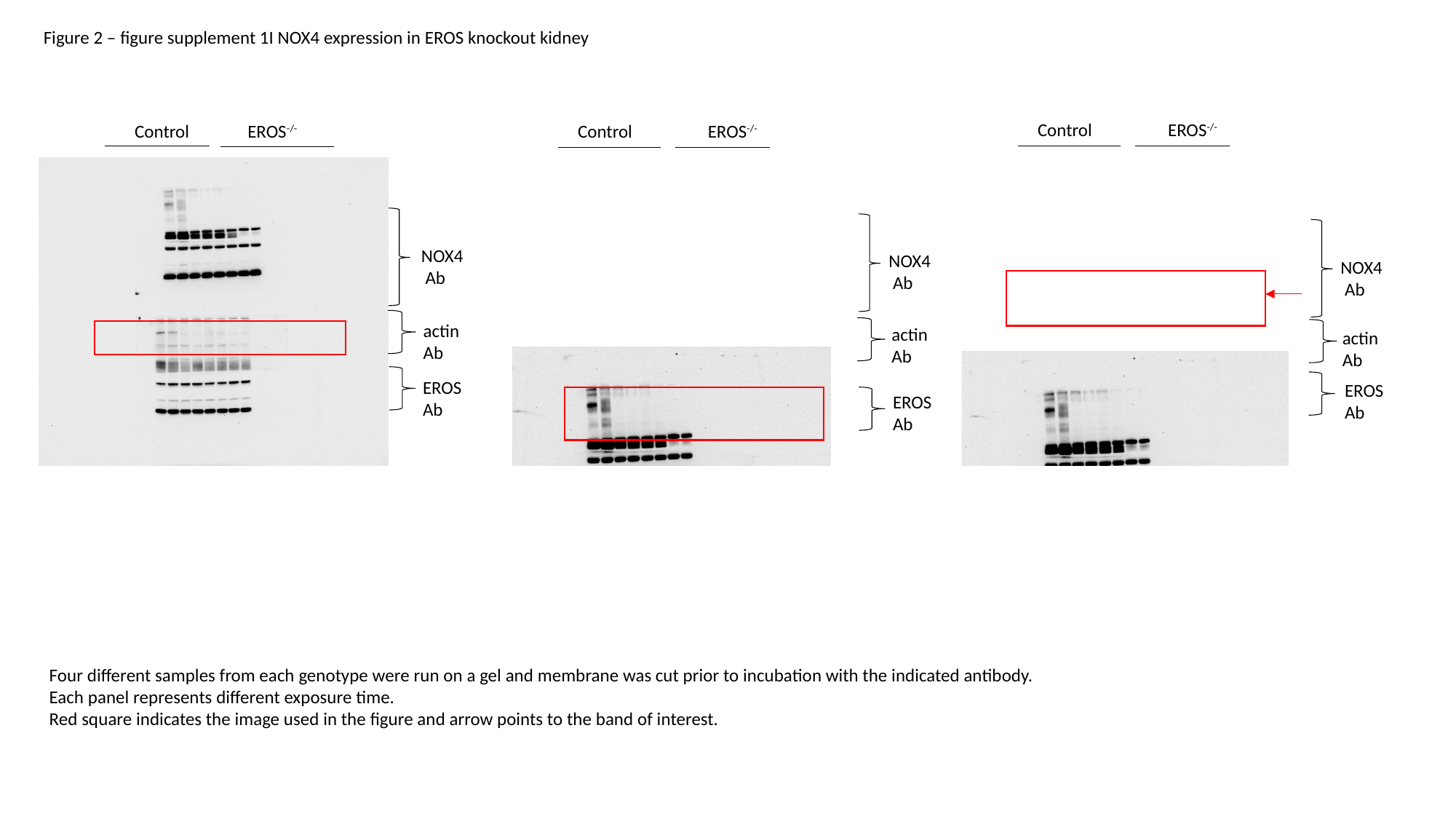

Figure 2 – figure supplement 1I NOX4 expression in EROS knockout kidney
EROS-/-
Control
EROS-/-
EROS-/-
Control
Control
NOX4
 Ab
NOX4
 Ab
NOX4
 Ab
actin
Ab
actin
Ab
actin
Ab
EROS
Ab
EROS
Ab
EROS
Ab
Four different samples from each genotype were run on a gel and membrane was cut prior to incubation with the indicated antibody.
Each panel represents different exposure time.
Red square indicates the image used in the figure and arrow points to the band of interest.

## Slide 4
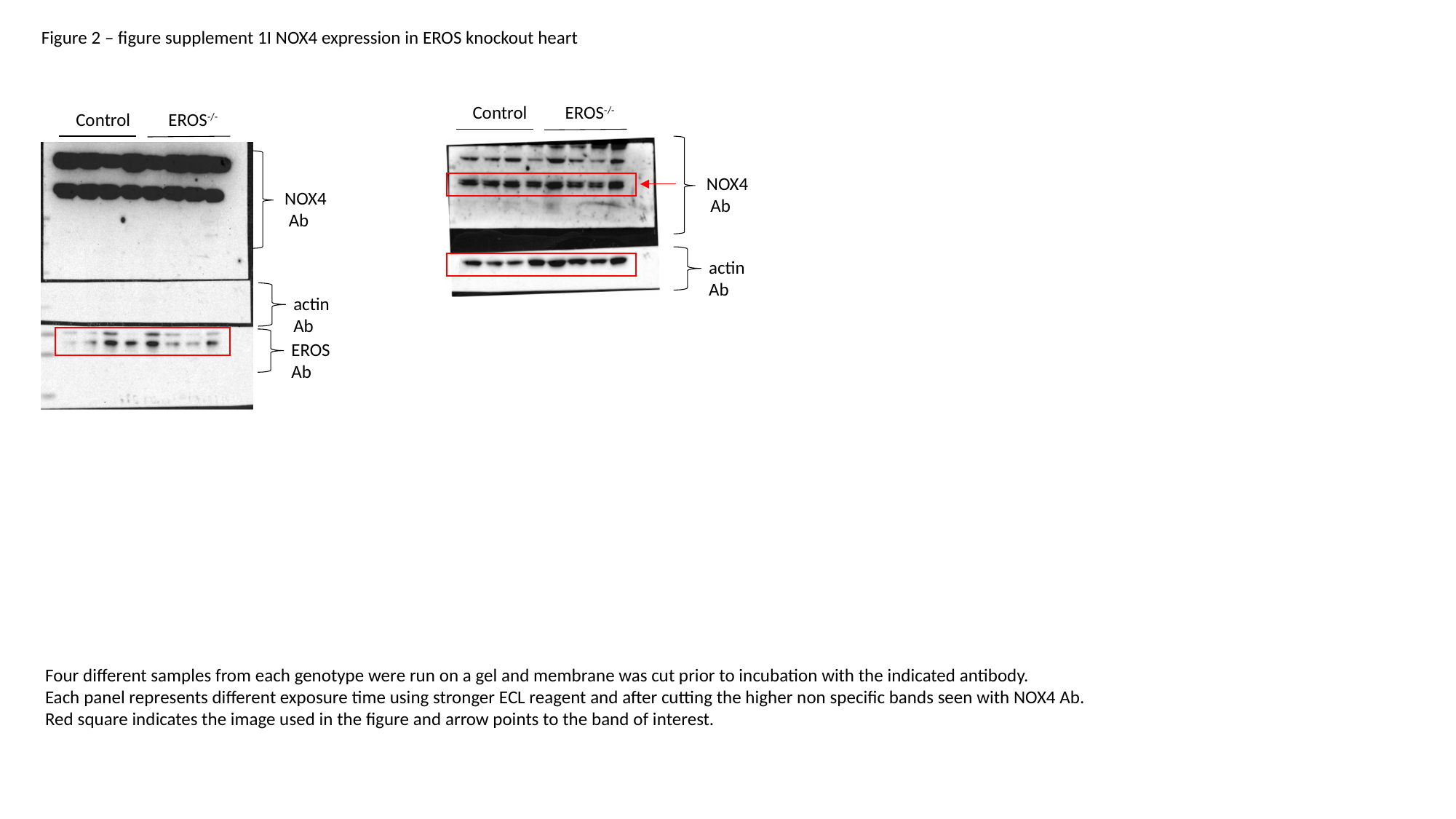

Figure 2 – figure supplement 1I NOX4 expression in EROS knockout heart
Control
EROS-/-
NOX4
 Ab
actin
Ab
Control
EROS-/-
NOX4
 Ab
actin
Ab
EROS
Ab
Four different samples from each genotype were run on a gel and membrane was cut prior to incubation with the indicated antibody.
Each panel represents different exposure time using stronger ECL reagent and after cutting the higher non specific bands seen with NOX4 Ab.
Red square indicates the image used in the figure and arrow points to the band of interest.
