# Supplementary material for: EROS is a selective chaperone regulating the phagocyte NADPH oxidase and purinergic signalling
Source: eLife. 2022 Nov 24;11:e76387. doi: 10.7554/eLife.76387 (PMC9767466; doi:10.7554/eLife.76387)
Supplement: Figure 3—source data 3. [file elife-76387-fig3-data3.zip › Figure 3- source data 3.pptx]

## Slide 1
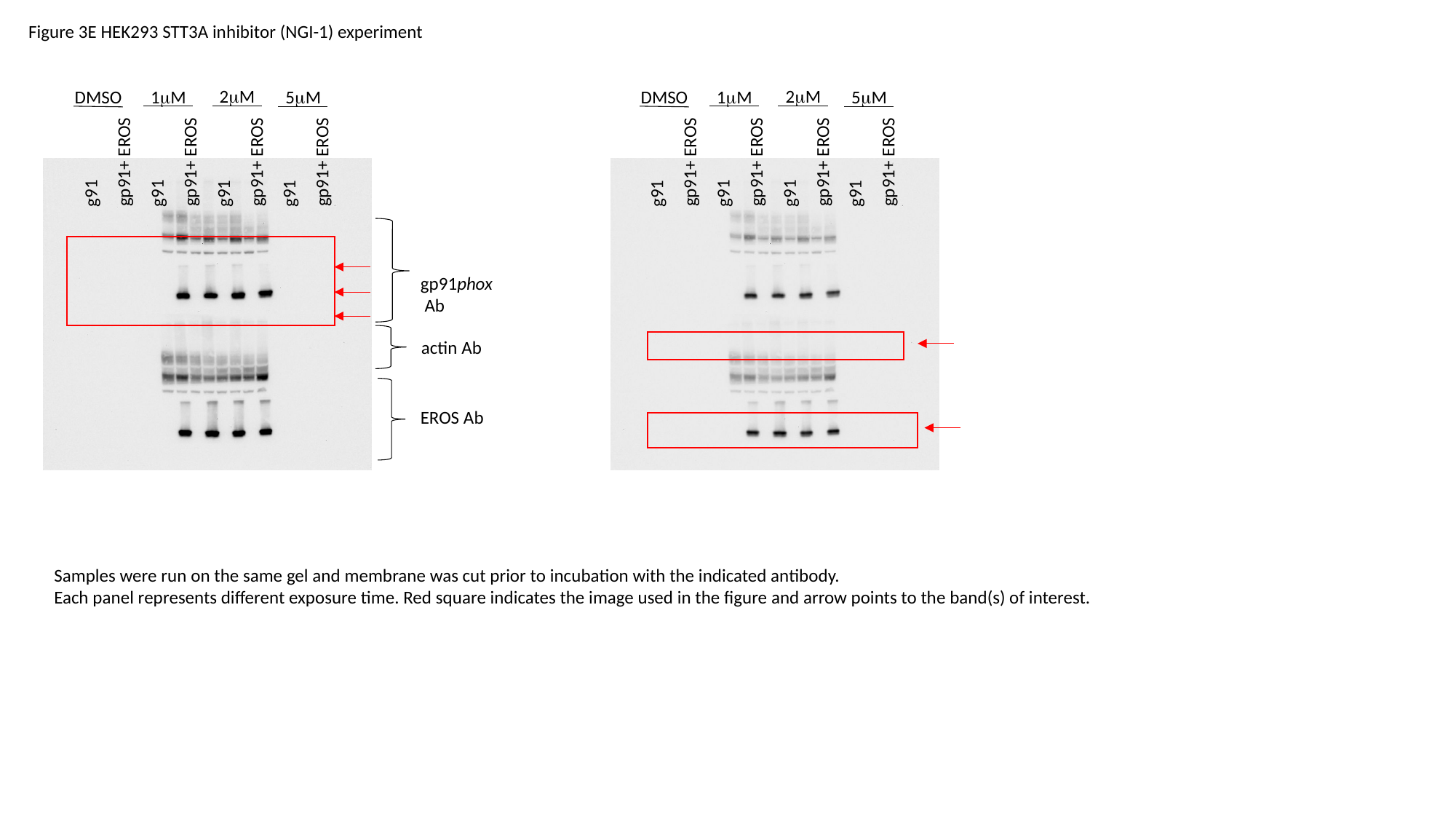

Figure 3E HEK293 STT3A inhibitor (NGI-1) experiment
2μM
2μM
1μM
1μM
DMSO
DMSO
5μM
5μM
gp91+ EROS
gp91+ EROS
gp91+ EROS
gp91+ EROS
gp91+ EROS
gp91+ EROS
gp91+ EROS
gp91+ EROS
g91
g91
g91
g91
g91
g91
g91
g91
gp91phox
 Ab
actin Ab
EROS Ab
Samples were run on the same gel and membrane was cut prior to incubation with the indicated antibody.
Each panel represents different exposure time. Red square indicates the image used in the figure and arrow points to the band(s) of interest.

## Slide 2
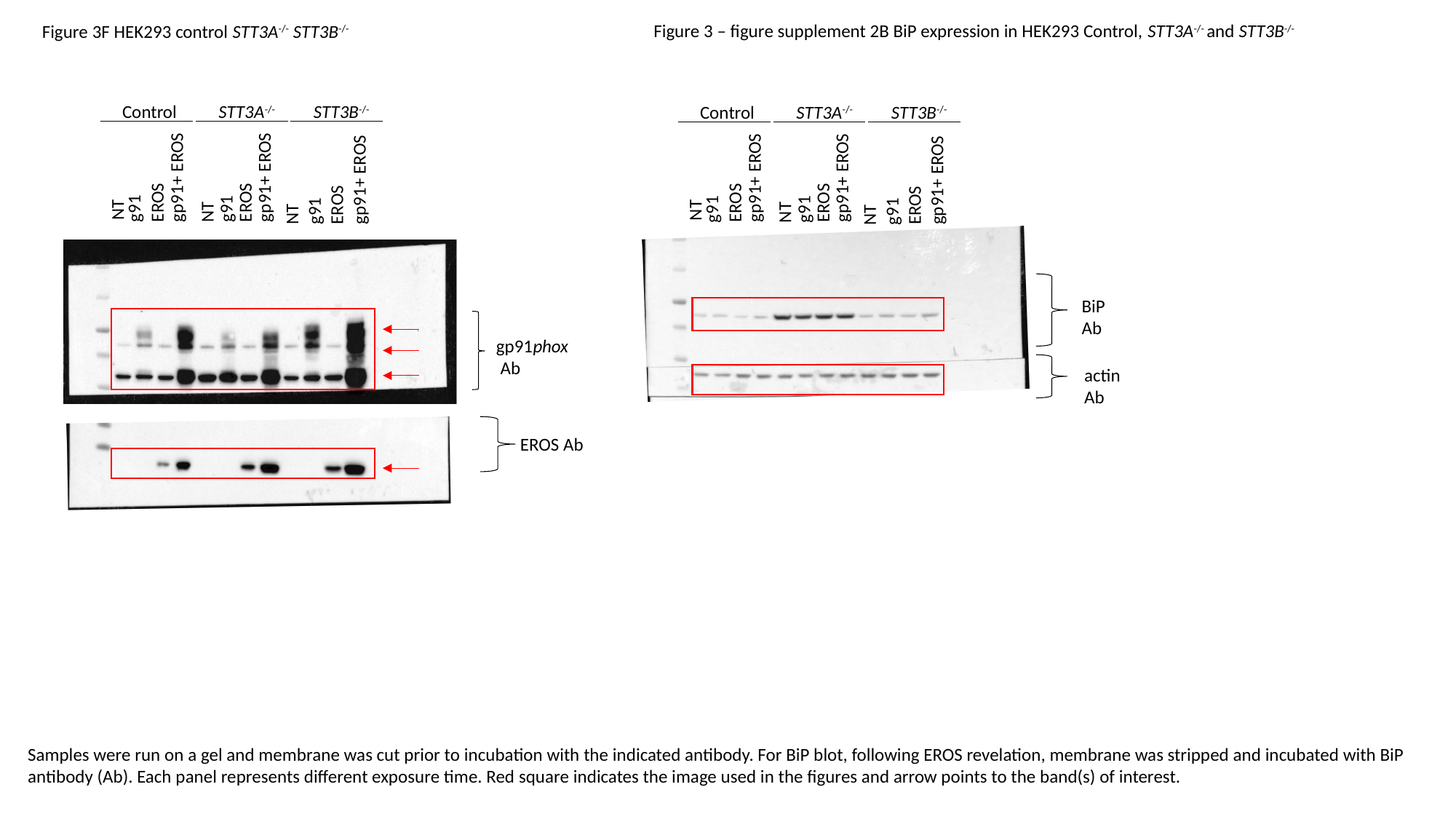

Figure 3 – figure supplement 2B BiP expression in HEK293 Control, STT3A-/- and STT3B-/-
Figure 3F HEK293 control STT3A-/- STT3B-/-
Control
STT3A-/-
STT3B-/-
Control
STT3A-/-
STT3B-/-
gp91+ EROS
gp91+ EROS
gp91+ EROS
gp91+ EROS
gp91+ EROS
gp91+ EROS
EROS
EROS
EROS
EROS
EROS
EROS
g91
g91
g91
g91
NT
NT
g91
g91
NT
NT
NT
NT
BiP
Ab
gp91phox
 Ab
actin
Ab
EROS Ab
Samples were run on a gel and membrane was cut prior to incubation with the indicated antibody. For BiP blot, following EROS revelation, membrane was stripped and incubated with BiP antibody (Ab). Each panel represents different exposure time. Red square indicates the image used in the figures and arrow points to the band(s) of interest.

## Slide 3
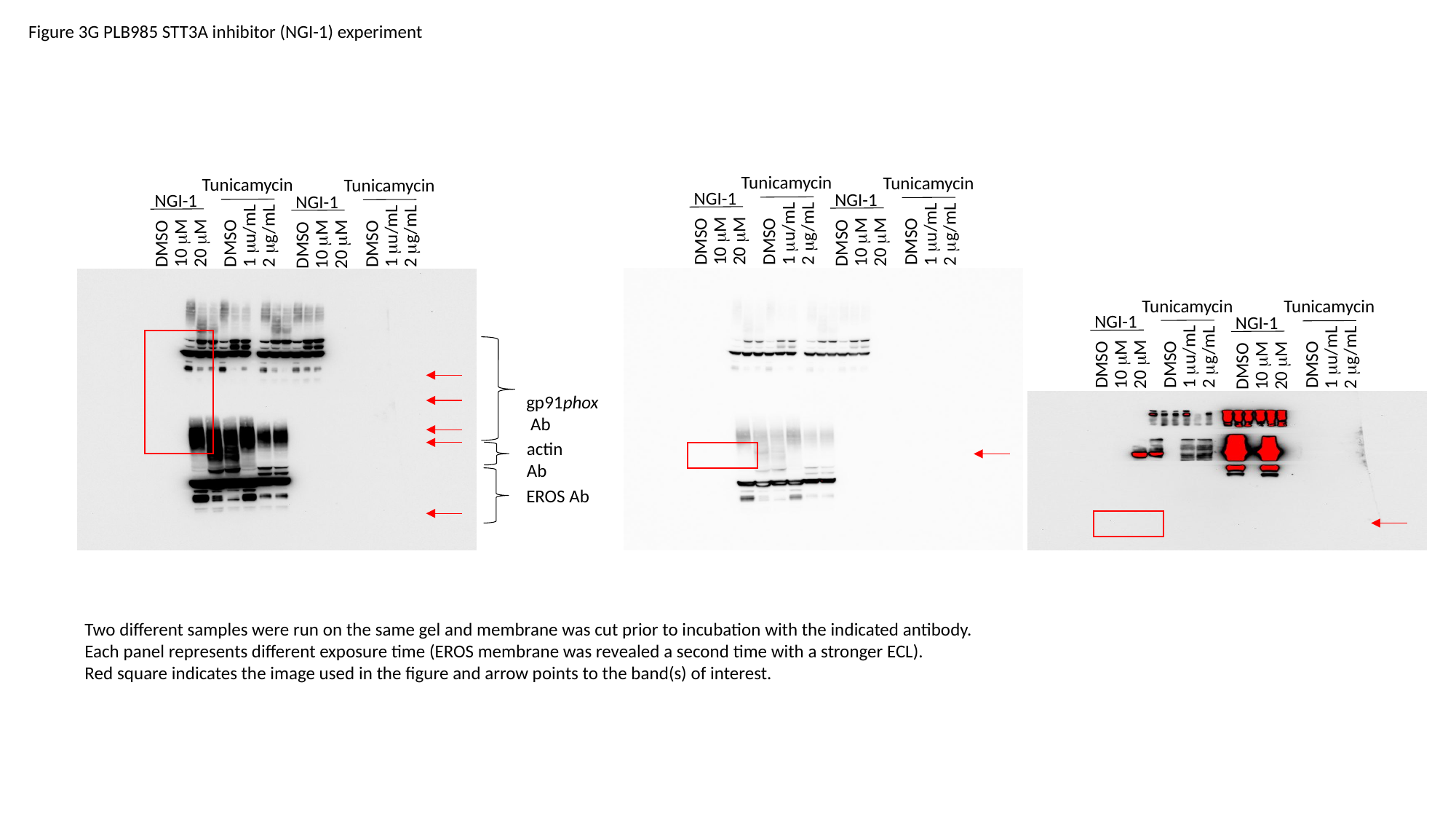

Figure 3G PLB985 STT3A inhibitor (NGI-1) experiment
Tunicamycin
Tunicamycin
Tunicamycin
Tunicamycin
NGI-1
NGI-1
NGI-1
NGI-1
1 u/mL
2 μg/mL
1 u/mL
2 μg/mL
1 u/mL
2 μg/mL
1 u/mL
2 μg/mL
10 μM
20 μM
DMSO
DMSO
DMSO
10 μM
20 μM
DMSO
10 μM
20 μM
DMSO
DMSO
DMSO
10 μM
20 μM
DMSO
Tunicamycin
Tunicamycin
NGI-1
NGI-1
1 u/mL
2 μg/mL
1 u/mL
2 μg/mL
10 μM
20 μM
DMSO
DMSO
DMSO
10 μM
20 μM
DMSO
gp91phox
 Ab
actin
Ab
EROS Ab
Two different samples were run on the same gel and membrane was cut prior to incubation with the indicated antibody.
Each panel represents different exposure time (EROS membrane was revealed a second time with a stronger ECL).
Red square indicates the image used in the figure and arrow points to the band(s) of interest.
