# Supplementary material for: EROS is a selective chaperone regulating the phagocyte NADPH oxidase and purinergic signalling
Source: eLife. 2022 Nov 24;11:e76387. doi: 10.7554/eLife.76387 (PMC9767466; doi:10.7554/eLife.76387)
Supplement: Figure 3—figure supplement 1—source data 2. [file elife-76387-fig3-figsupp1-data2.zip › Figure 3 figure supplement 1- source data 2.pptx]

## Slide 1
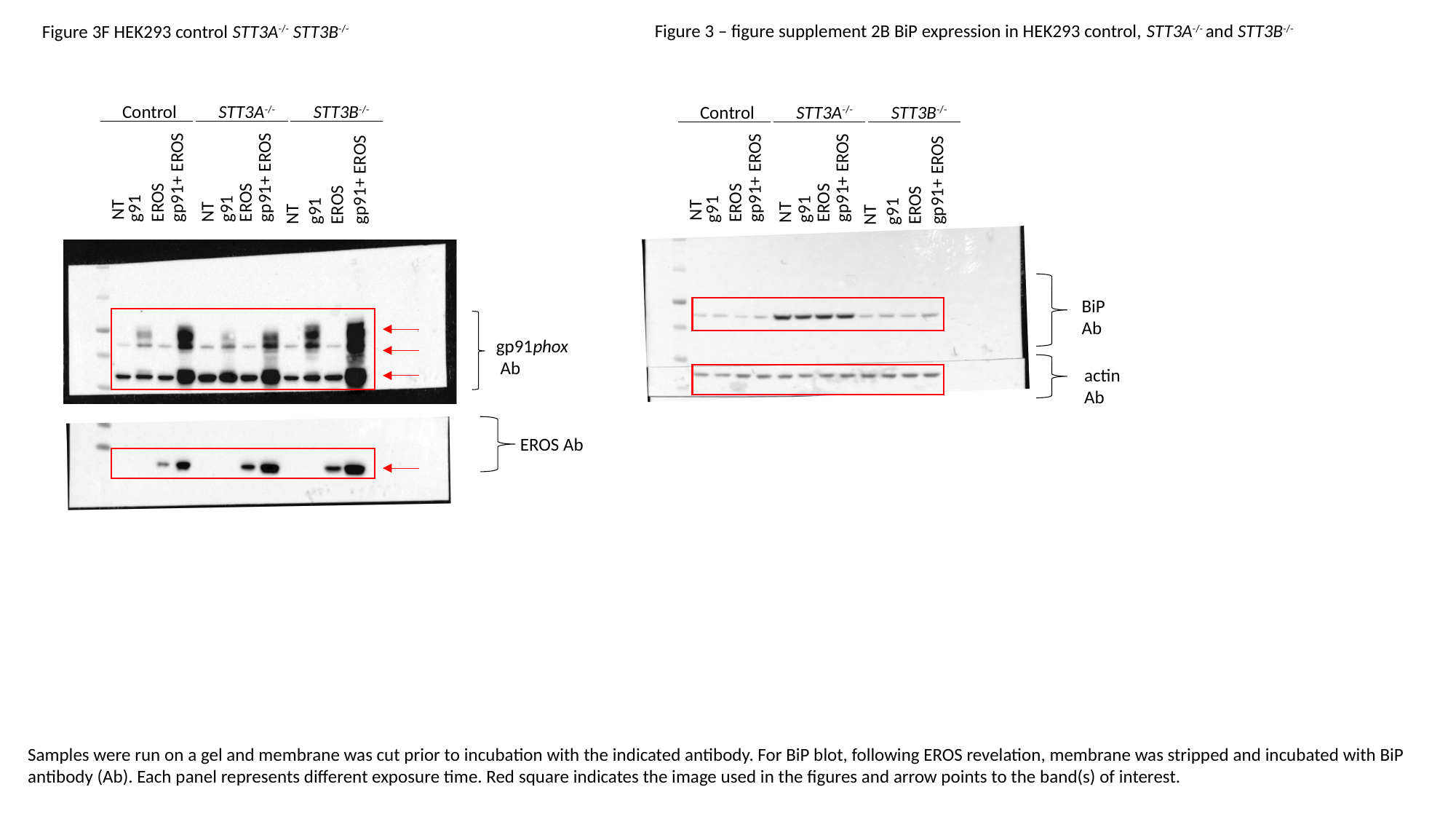

Figure 3 – figure supplement 2B BiP expression in HEK293 control, STT3A-/- and STT3B-/-
Figure 3F HEK293 control STT3A-/- STT3B-/-
Control
STT3A-/-
STT3B-/-
Control
STT3A-/-
STT3B-/-
gp91+ EROS
gp91+ EROS
gp91+ EROS
gp91+ EROS
gp91+ EROS
gp91+ EROS
EROS
EROS
EROS
EROS
EROS
EROS
g91
g91
g91
g91
NT
NT
g91
g91
NT
NT
NT
NT
BiP
Ab
gp91phox
 Ab
actin
Ab
EROS Ab
Samples were run on a gel and membrane was cut prior to incubation with the indicated antibody. For BiP blot, following EROS revelation, membrane was stripped and incubated with BiP antibody (Ab). Each panel represents different exposure time. Red square indicates the image used in the figures and arrow points to the band(s) of interest.

## Slide 2
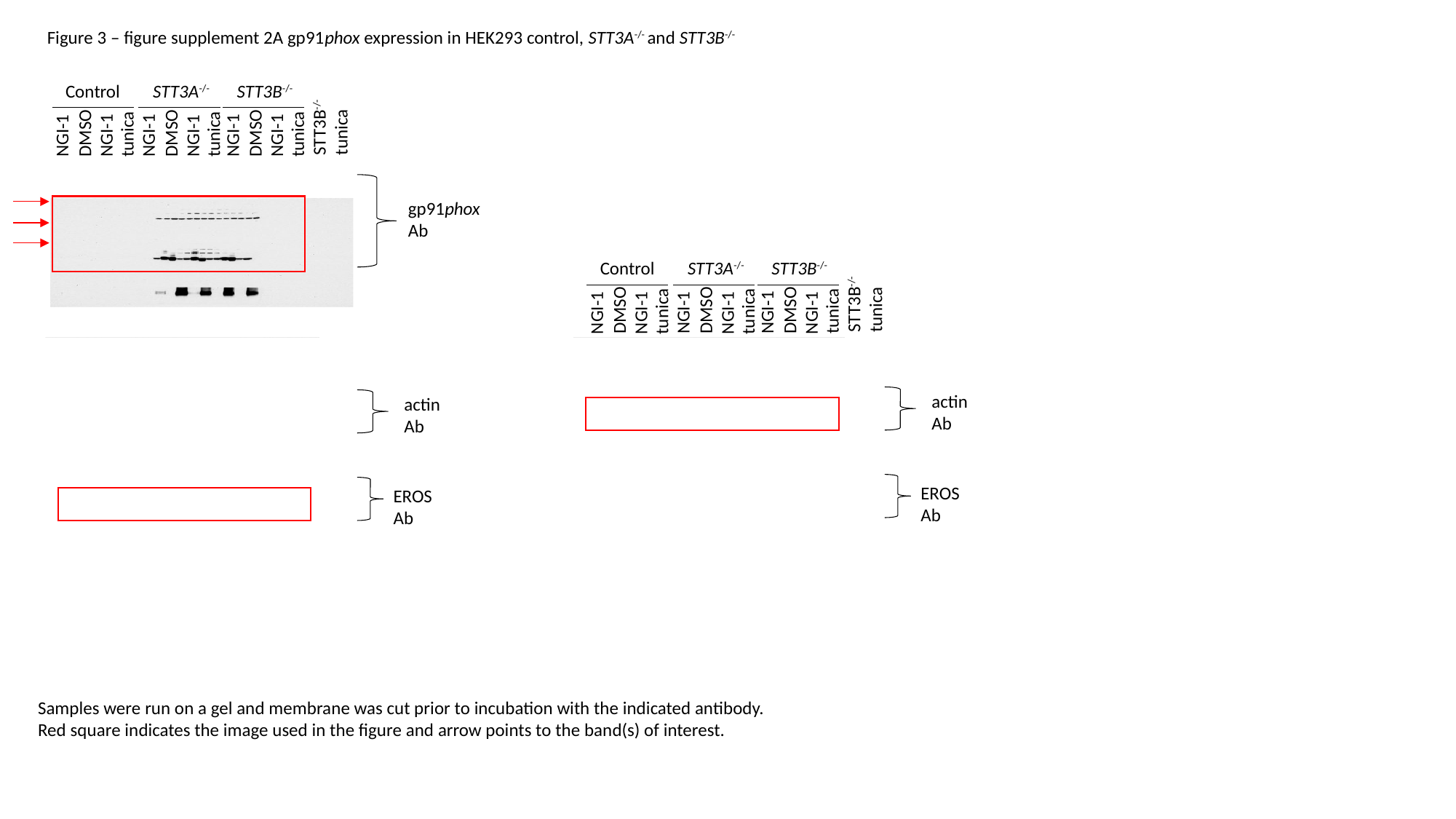

Figure 3 – figure supplement 2A gp91phox expression in HEK293 control, STT3A-/- and STT3B-/-
STT3B-/-
STT3A-/-
Control
STT3B-/-
tunica
DMSO
DMSO
DMSO
tunica
tunica
tunica
NGI-1
NGI-1
NGI-1
NGI-1
NGI-1
NGI-1
gp91phox
Ab
STT3B-/-
STT3A-/-
Control
STT3B-/-
tunica
DMSO
DMSO
DMSO
tunica
tunica
tunica
NGI-1
NGI-1
NGI-1
NGI-1
NGI-1
NGI-1
actin
Ab
actin
Ab
EROS
Ab
EROS
Ab
Samples were run on a gel and membrane was cut prior to incubation with the indicated antibody.
Red square indicates the image used in the figure and arrow points to the band(s) of interest.

## Slide 3
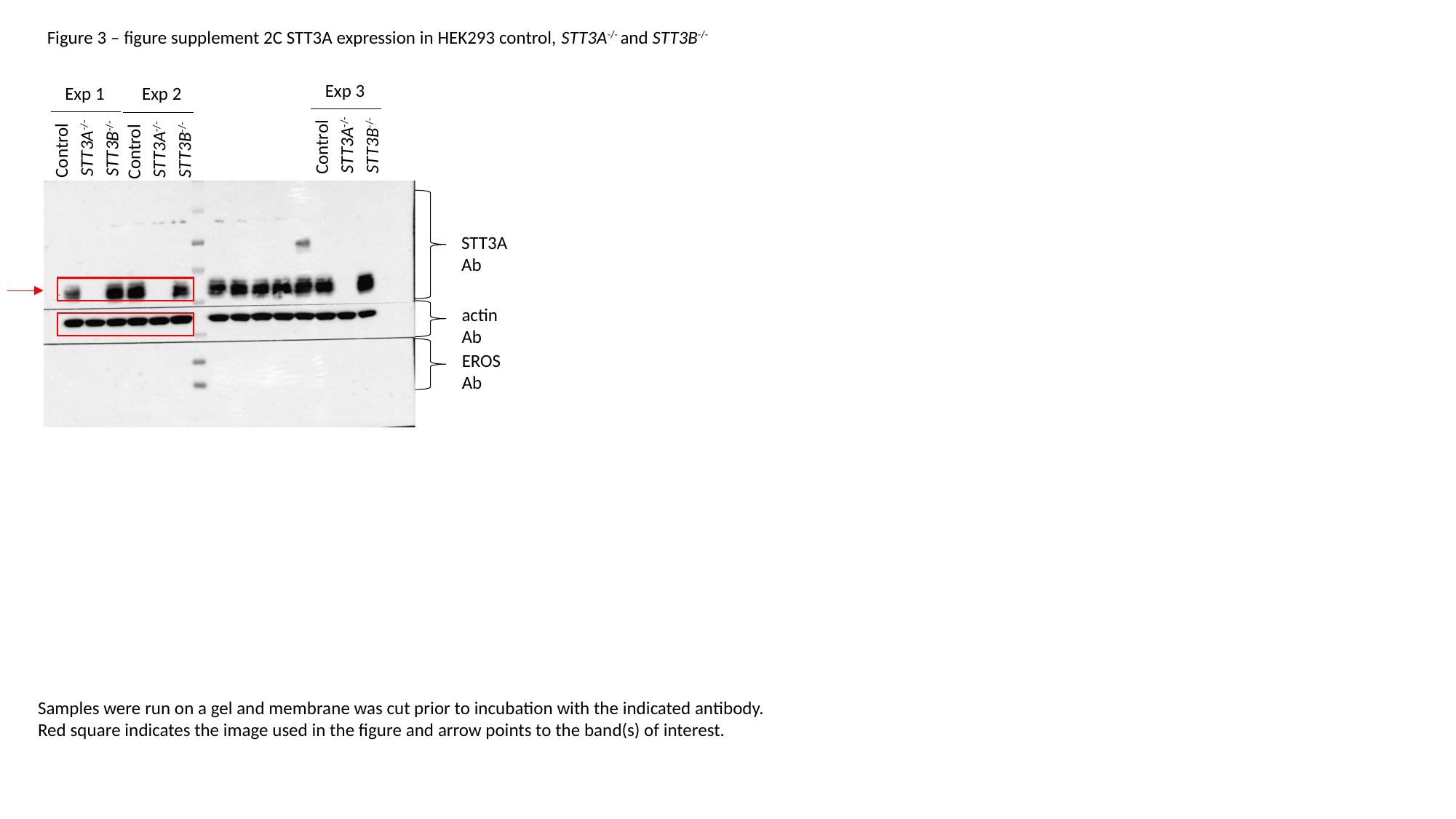

Figure 3 – figure supplement 2C STT3A expression in HEK293 control, STT3A-/- and STT3B-/-
Exp 3
Exp 1
Exp 2
STT3A-/-
STT3B-/-
Control
STT3A-/-
STT3B-/-
STT3A-/-
STT3B-/-
Control
Control
STT3A
Ab
actin
Ab
EROS
Ab
Samples were run on a gel and membrane was cut prior to incubation with the indicated antibody.
Red square indicates the image used in the figure and arrow points to the band(s) of interest.

## Slide 4
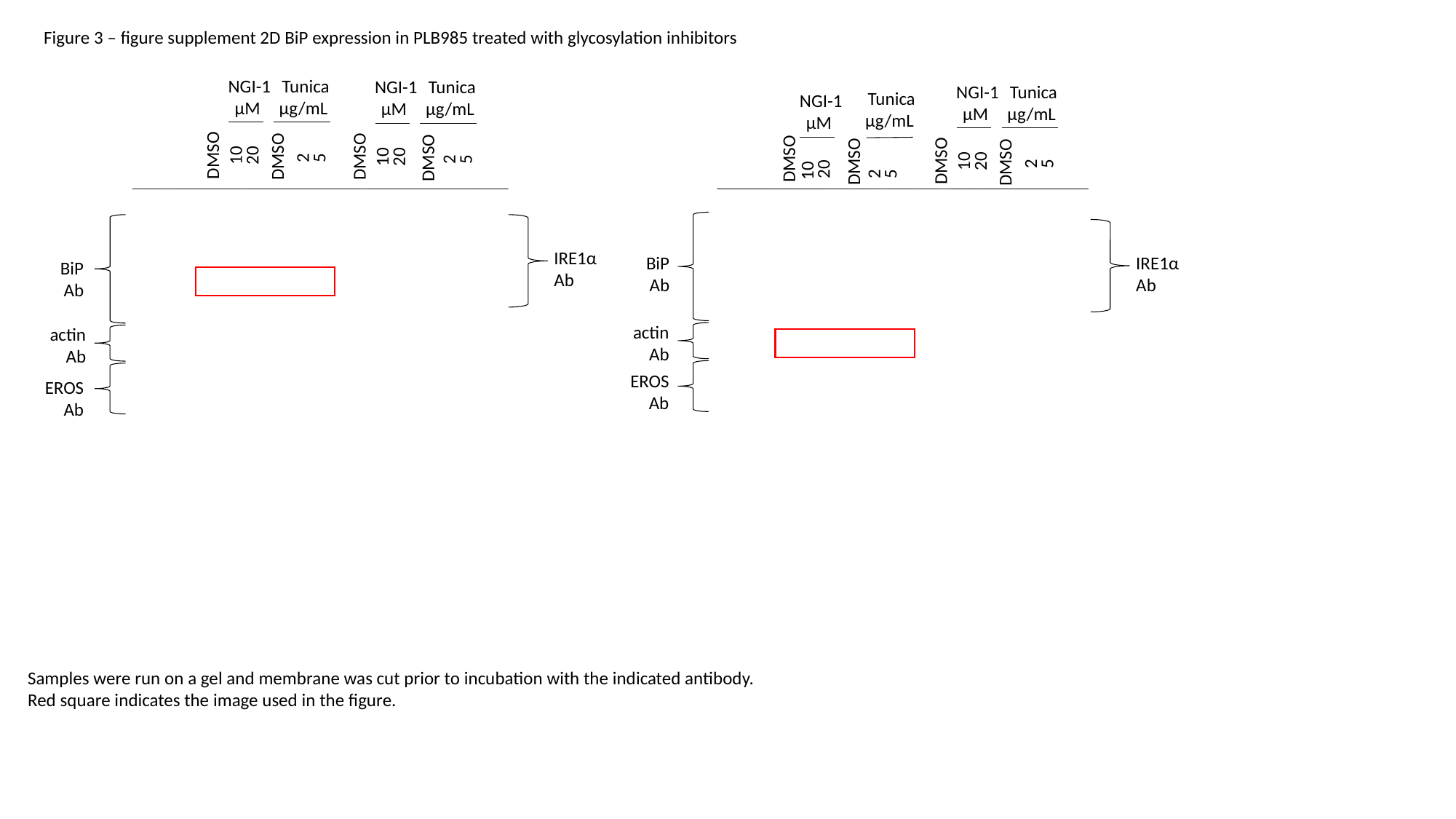

Figure 3 – figure supplement 2D BiP expression in PLB985 treated with glycosylation inhibitors
NGI-1
µM
Tunica
µg/mL
NGI-1
µM
Tunica
µg/mL
NGI-1
µM
Tunica
µg/mL
Tunica
µg/mL
NGI-1
µM
DMSO
10
20
DMSO
DMSO
10
20
DMSO
2
5
DMSO
2
5
DMSO
10
20
DMSO
DMSO
2
5
20
10
2
5
IRE1α
Ab
BiP
Ab
IRE1α
Ab
BiP
Ab
actin
Ab
actin
Ab
EROS
Ab
EROS
Ab
Samples were run on a gel and membrane was cut prior to incubation with the indicated antibody.
Red square indicates the image used in the figure.
