# Supplementary material for: EROS is a selective chaperone regulating the phagocyte NADPH oxidase and purinergic signalling
Source: eLife. 2022 Nov 24;11:e76387. doi: 10.7554/eLife.76387 (PMC9767466; doi:10.7554/eLife.76387)
Supplement: Figure 5—source data 4. [file elife-76387-fig5-data4.zip › Figure 5- source data 4.pptx]

## Slide 1
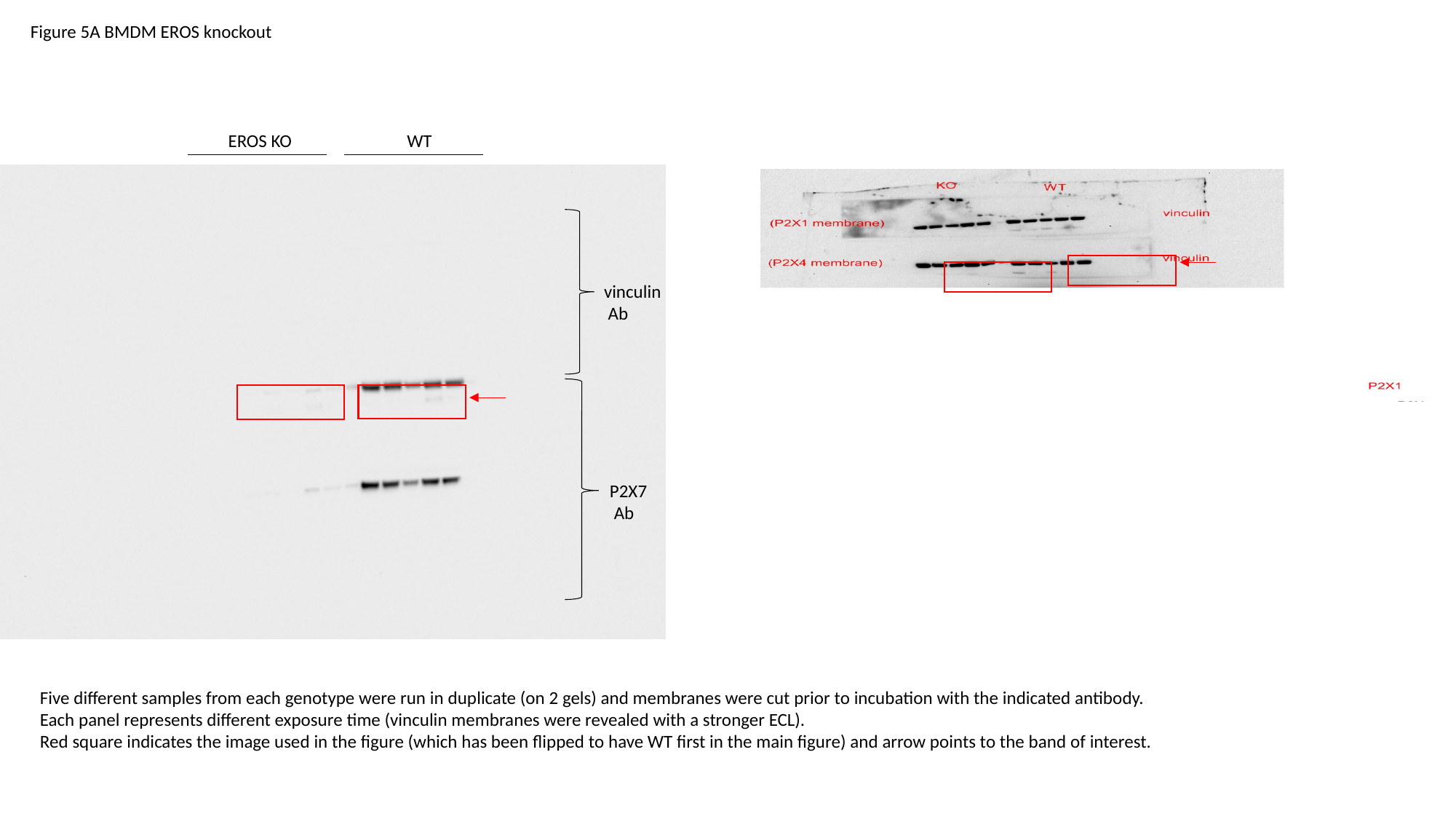

Figure 5A BMDM EROS knockout
EROS KO
WT
vinculin
 Ab
P2X7
 Ab
Five different samples from each genotype were run in duplicate (on 2 gels) and membranes were cut prior to incubation with the indicated antibody.
Each panel represents different exposure time (vinculin membranes were revealed with a stronger ECL).
Red square indicates the image used in the figure (which has been flipped to have WT first in the main figure) and arrow points to the band of interest.

## Slide 2
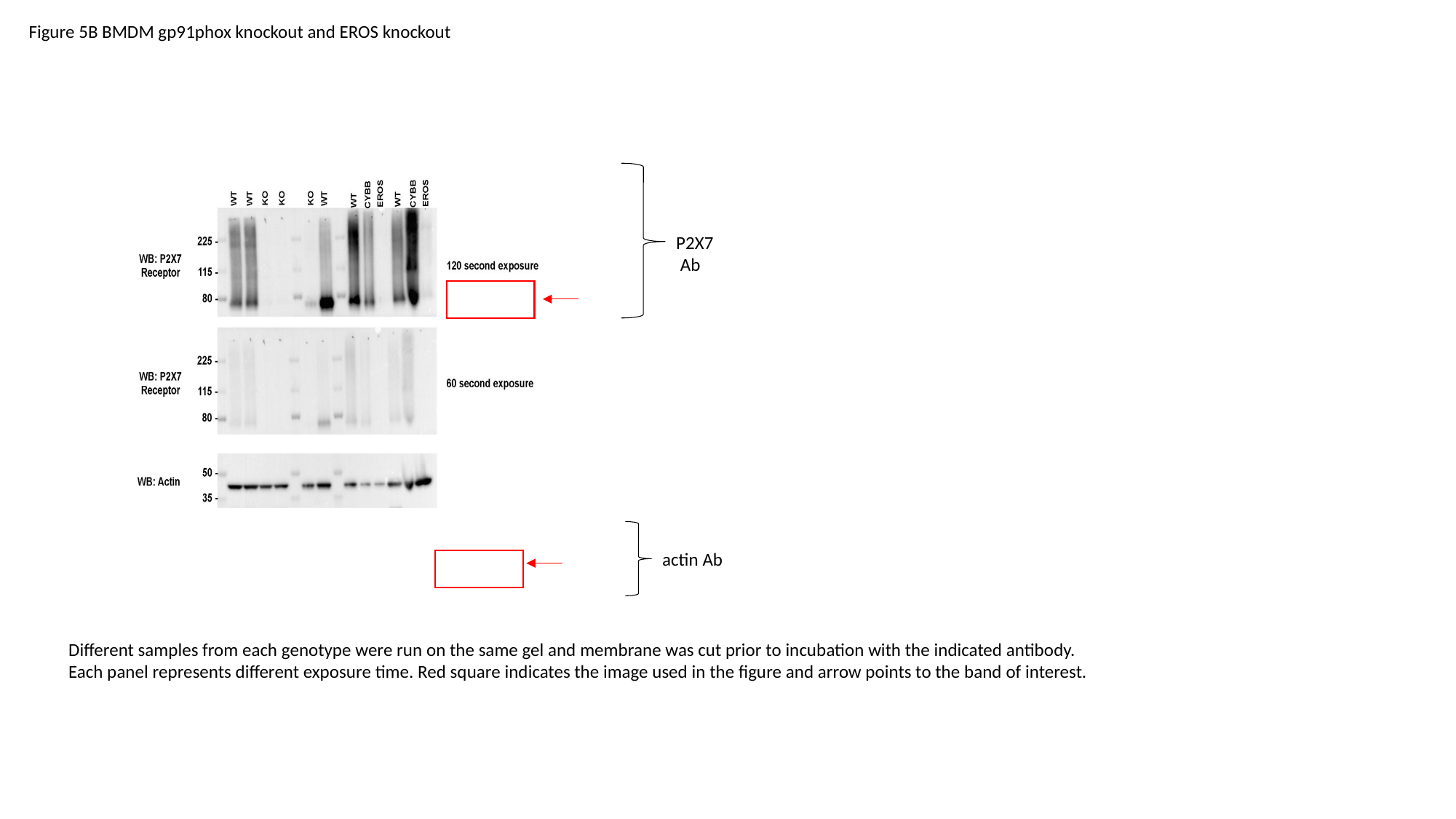

Figure 5B BMDM gp91phox knockout and EROS knockout
P2X7
 Ab
actin Ab
Different samples from each genotype were run on the same gel and membrane was cut prior to incubation with the indicated antibody.
Each panel represents different exposure time. Red square indicates the image used in the figure and arrow points to the band of interest.

## Slide 3
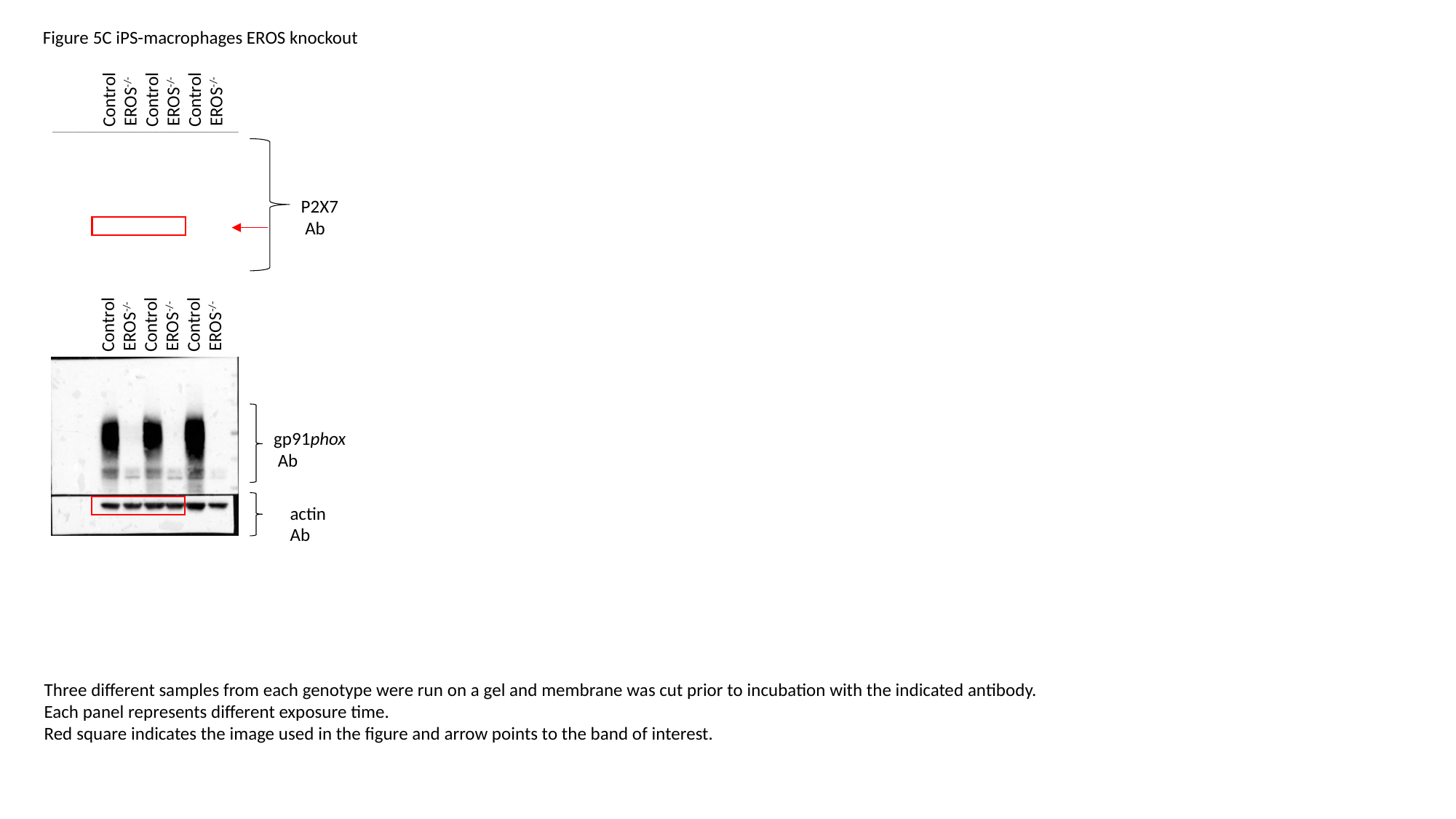

Figure 5C iPS-macrophages EROS knockout
Control
Control
Control
EROS-/-
EROS-/-
EROS-/-
P2X7
 Ab
Control
Control
Control
EROS-/-
EROS-/-
EROS-/-
gp91phox
 Ab
actin
Ab
Three different samples from each genotype were run on a gel and membrane was cut prior to incubation with the indicated antibody.
Each panel represents different exposure time.
Red square indicates the image used in the figure and arrow points to the band of interest.

## Slide 4
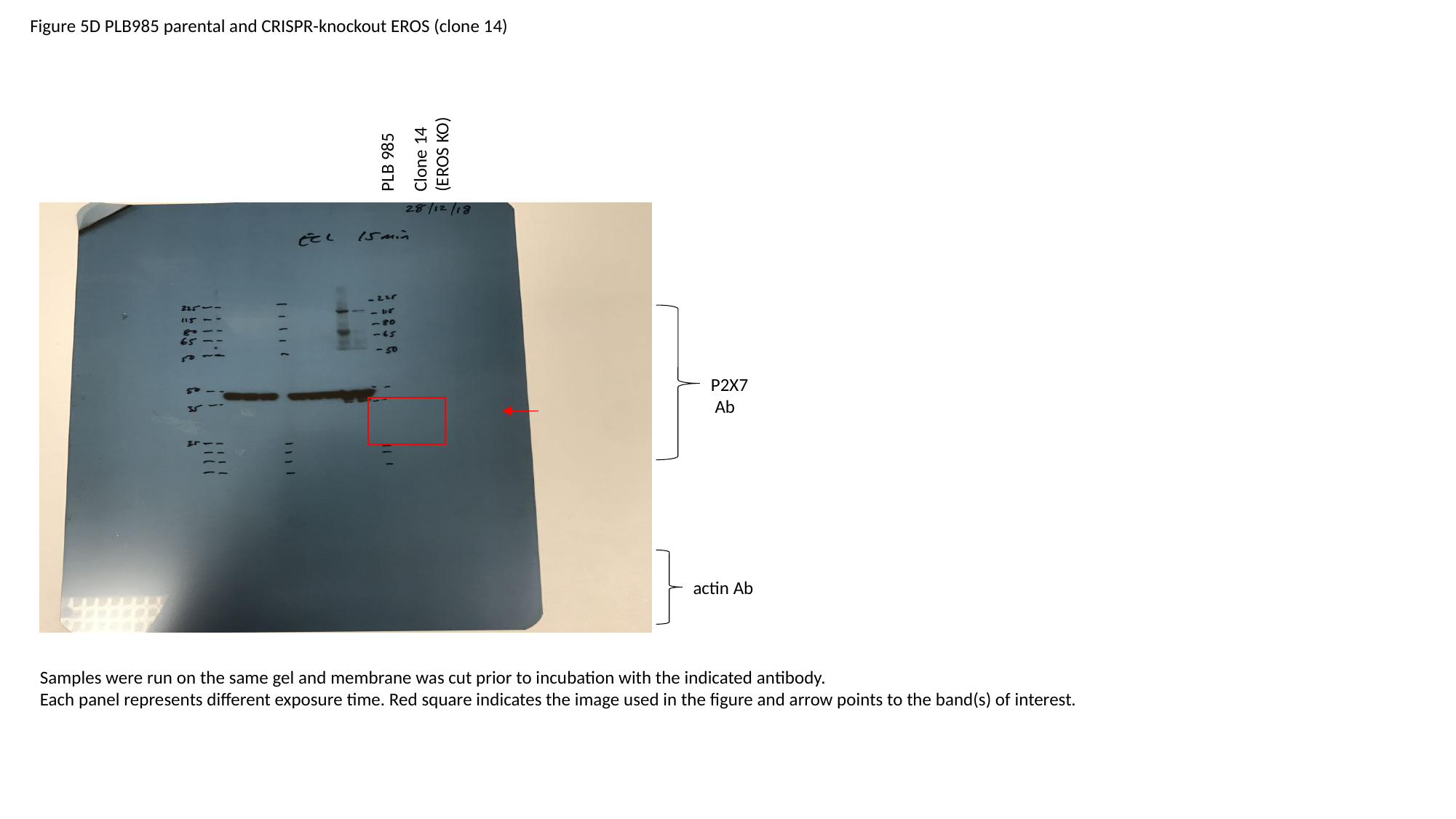

Figure 5D PLB985 parental and CRISPR-knockout EROS (clone 14)
Clone 14 (EROS KO)
PLB 985
P2X7
 Ab
actin Ab
Samples were run on the same gel and membrane was cut prior to incubation with the indicated antibody.
Each panel represents different exposure time. Red square indicates the image used in the figure and arrow points to the band(s) of interest.

## Slide 5
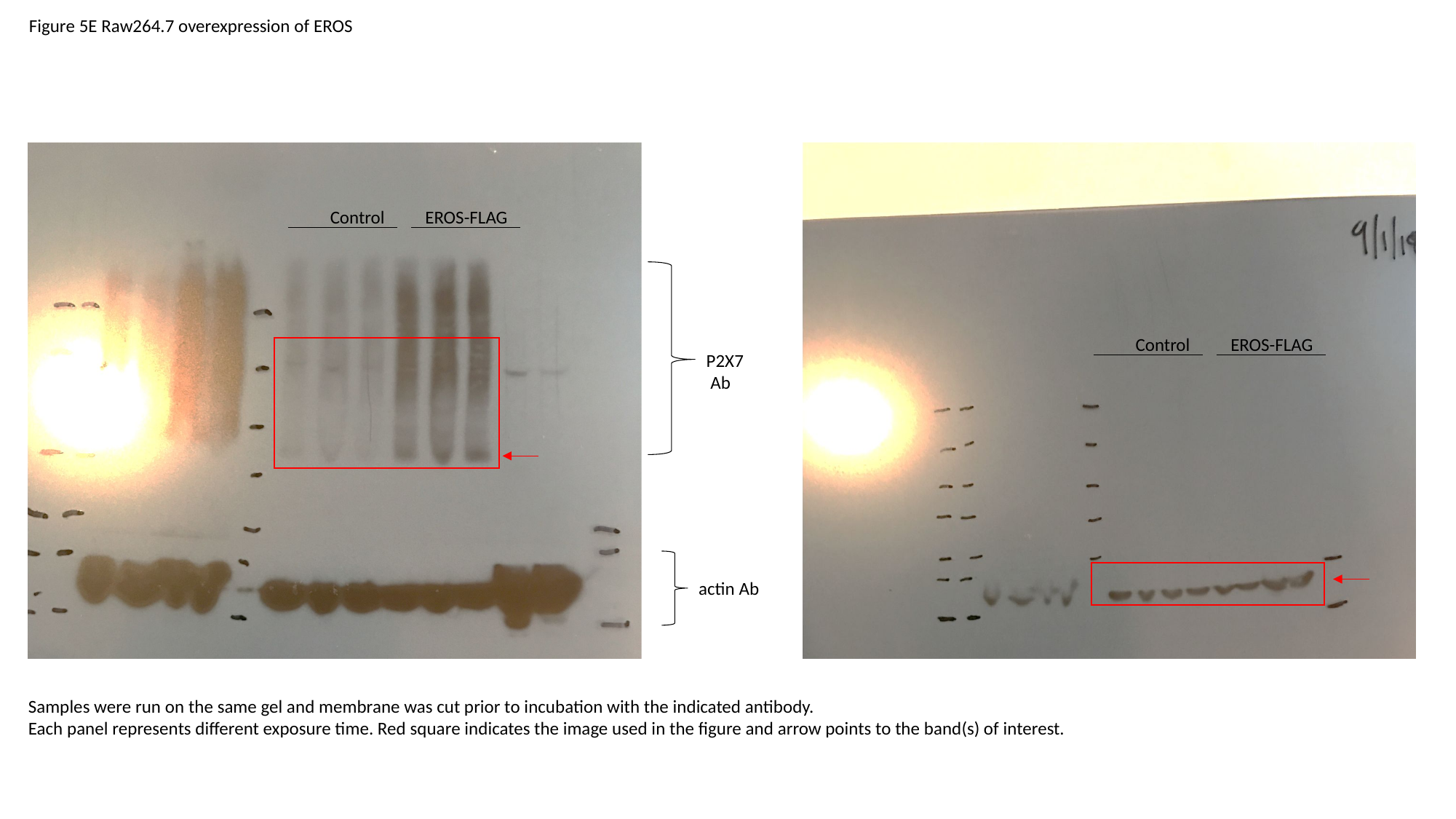

Figure 5E Raw264.7 overexpression of EROS
Control
EROS-FLAG
Control
EROS-FLAG
P2X7
 Ab
actin Ab
Samples were run on the same gel and membrane was cut prior to incubation with the indicated antibody.
Each panel represents different exposure time. Red square indicates the image used in the figure and arrow points to the band(s) of interest.

## Slide 6
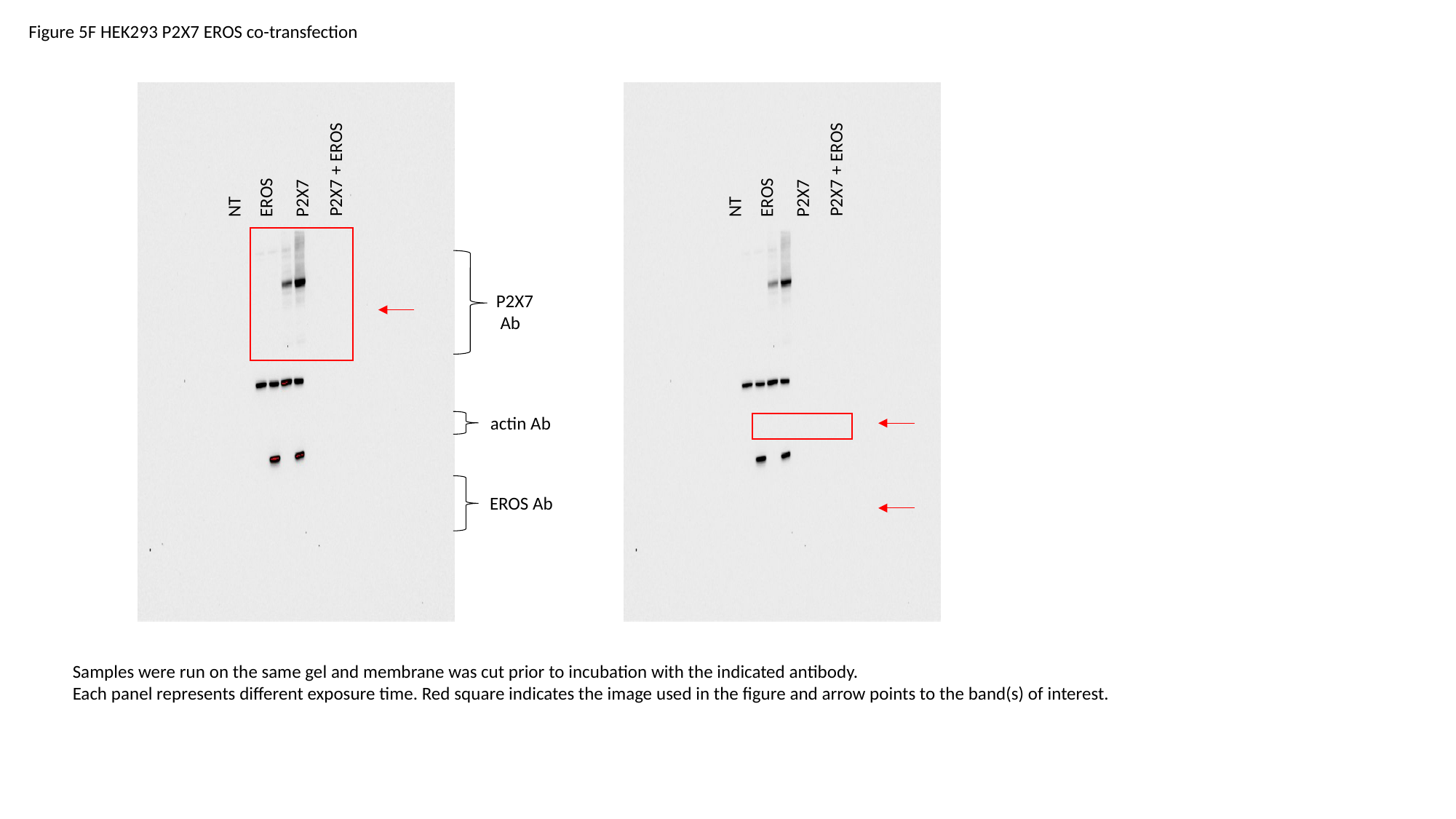

Figure 5F HEK293 P2X7 EROS co-transfection
P2X7 + EROS
P2X7 + EROS
EROS
EROS
P2X7
P2X7
NT
NT
P2X7
 Ab
actin Ab
EROS Ab
Samples were run on the same gel and membrane was cut prior to incubation with the indicated antibody.
Each panel represents different exposure time. Red square indicates the image used in the figure and arrow points to the band(s) of interest.

## Slide 7
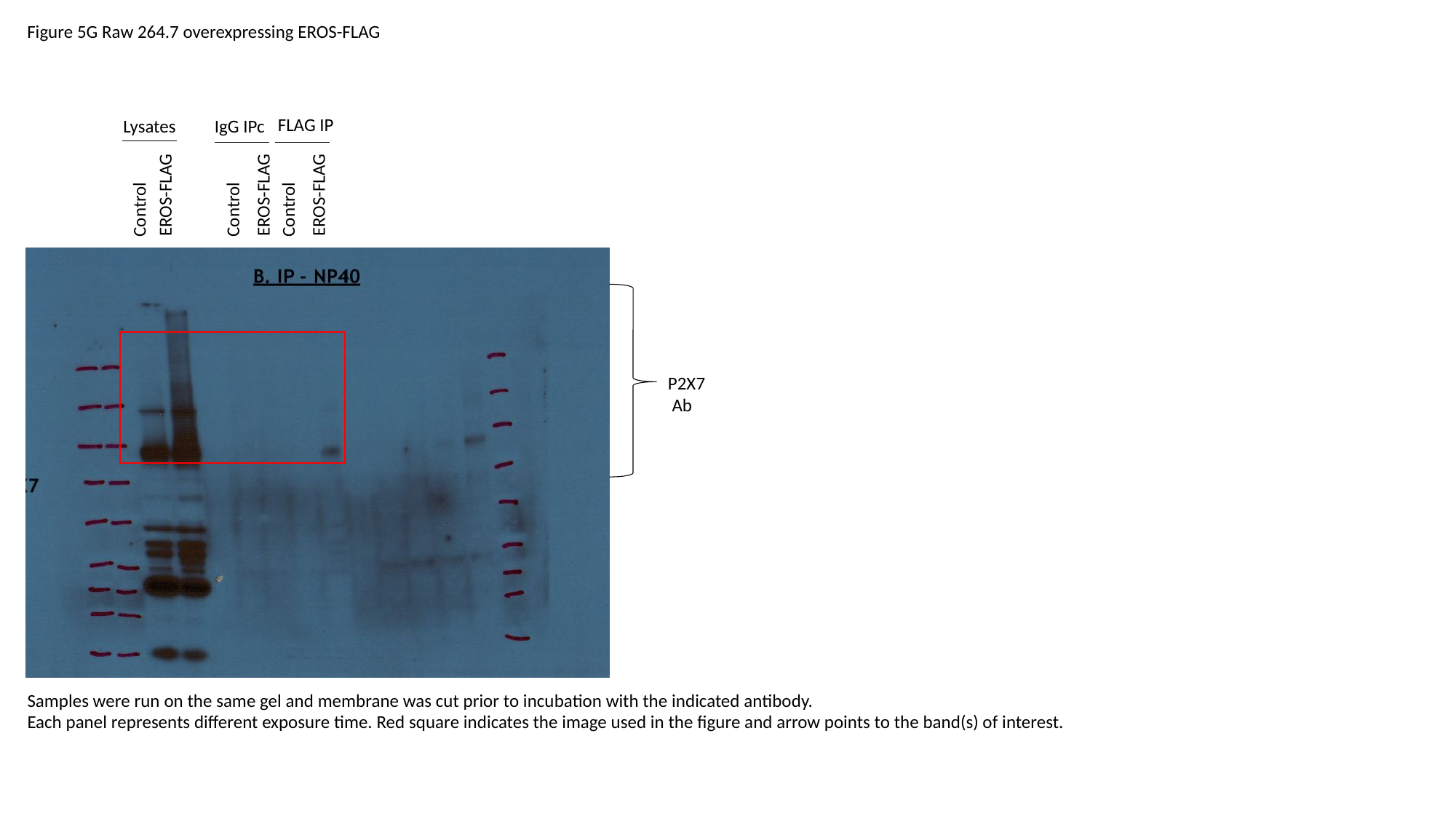

Figure 5G Raw 264.7 overexpressing EROS-FLAG
FLAG IP
Lysates
IgG IPc
EROS-FLAG
EROS-FLAG
EROS-FLAG
Control
Control
Control
P2X7
 Ab
Samples were run on the same gel and membrane was cut prior to incubation with the indicated antibody.
Each panel represents different exposure time. Red square indicates the image used in the figure and arrow points to the band(s) of interest.

## Slide 8
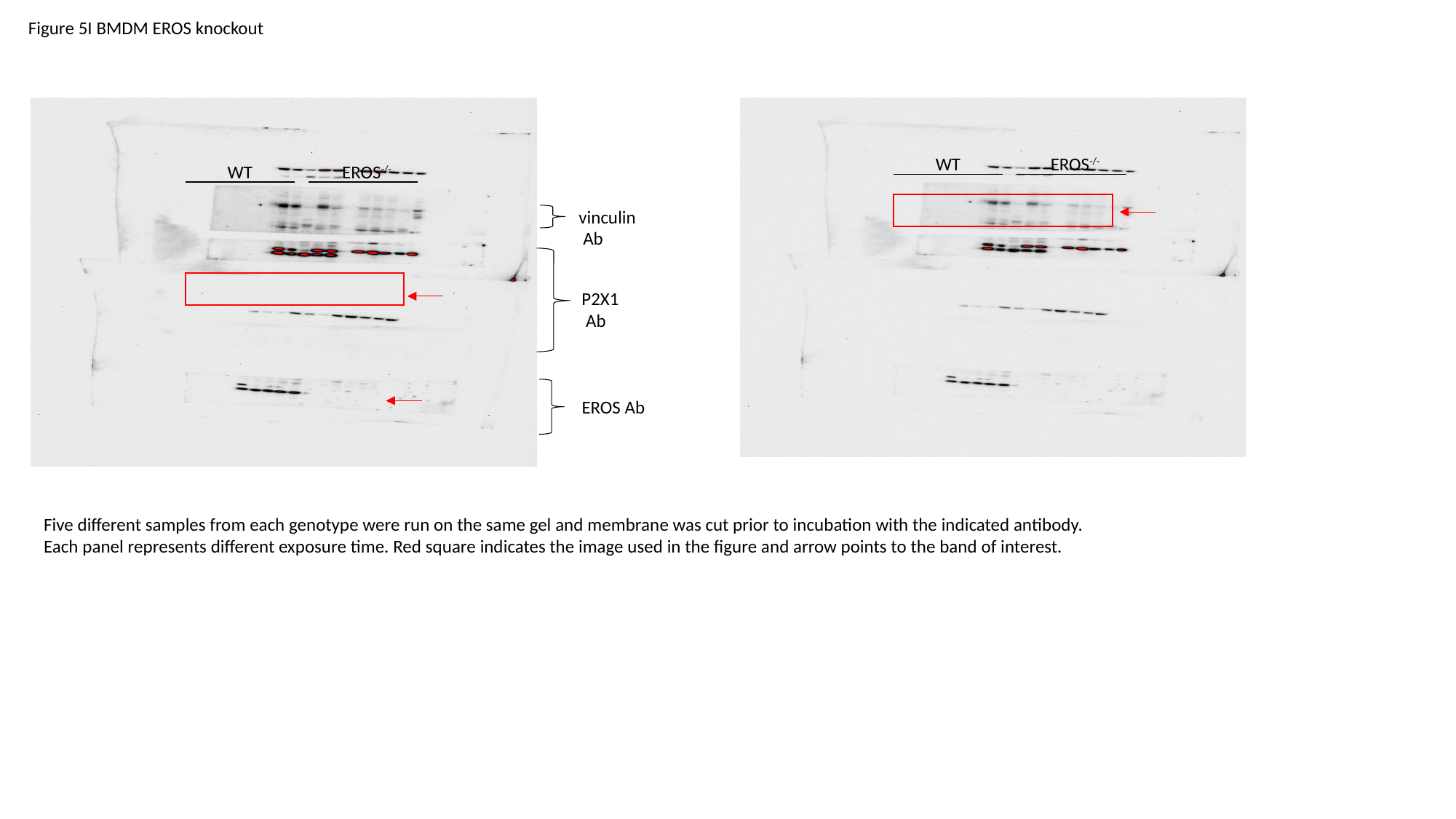

Figure 5I BMDM EROS knockout
WT
EROS-/-
WT
EROS-/-
vinculin
 Ab
P2X1
 Ab
EROS Ab
Five different samples from each genotype were run on the same gel and membrane was cut prior to incubation with the indicated antibody.
Each panel represents different exposure time. Red square indicates the image used in the figure and arrow points to the band of interest.

## Slide 9
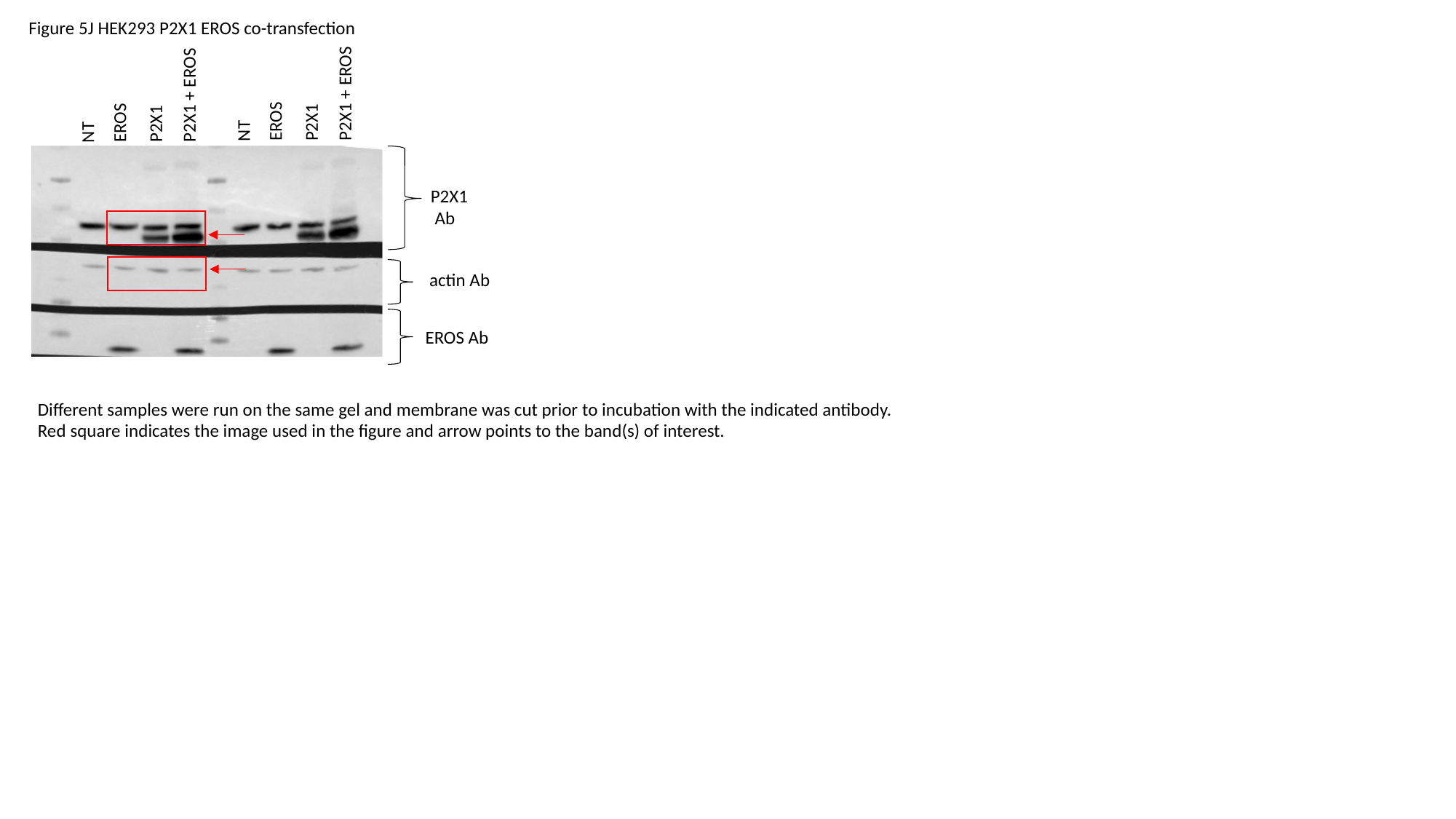

Figure 5J HEK293 P2X1 EROS co-transfection
P2X1 + EROS
P2X1 + EROS
EROS
P2X1
EROS
P2X1
NT
NT
P2X1
 Ab
actin Ab
EROS Ab
Different samples were run on the same gel and membrane was cut prior to incubation with the indicated antibody.
Red square indicates the image used in the figure and arrow points to the band(s) of interest.
