# Supplementary material for: EROS is a selective chaperone regulating the phagocyte NADPH oxidase and purinergic signalling
Source: eLife. 2022 Nov 24;11:e76387. doi: 10.7554/eLife.76387 (PMC9767466; doi:10.7554/eLife.76387)
Supplement: Figure 5—figure supplement 1—source data 2. [file elife-76387-fig5-figsupp1-data2.zip › Figure 5 figure supplement 1- source data 2.pptx]

## Slide 1
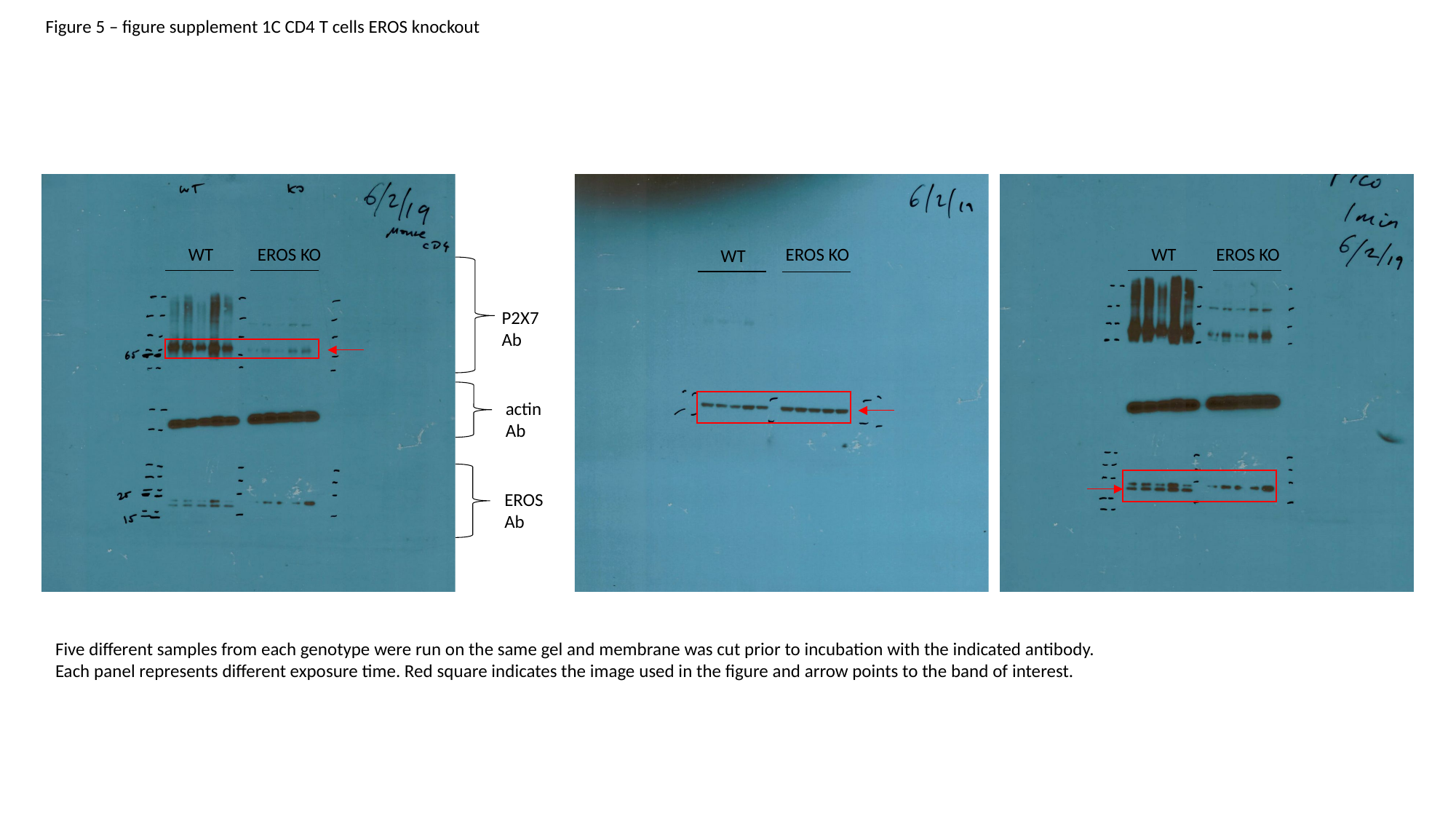

Figure 5 – figure supplement 1C CD4 T cells EROS knockout
WT
EROS KO
EROS KO
WT
EROS KO
WT
 P2X7
 Ab
actin
Ab
EROS Ab
Five different samples from each genotype were run on the same gel and membrane was cut prior to incubation with the indicated antibody.
Each panel represents different exposure time. Red square indicates the image used in the figure and arrow points to the band of interest.

## Slide 2
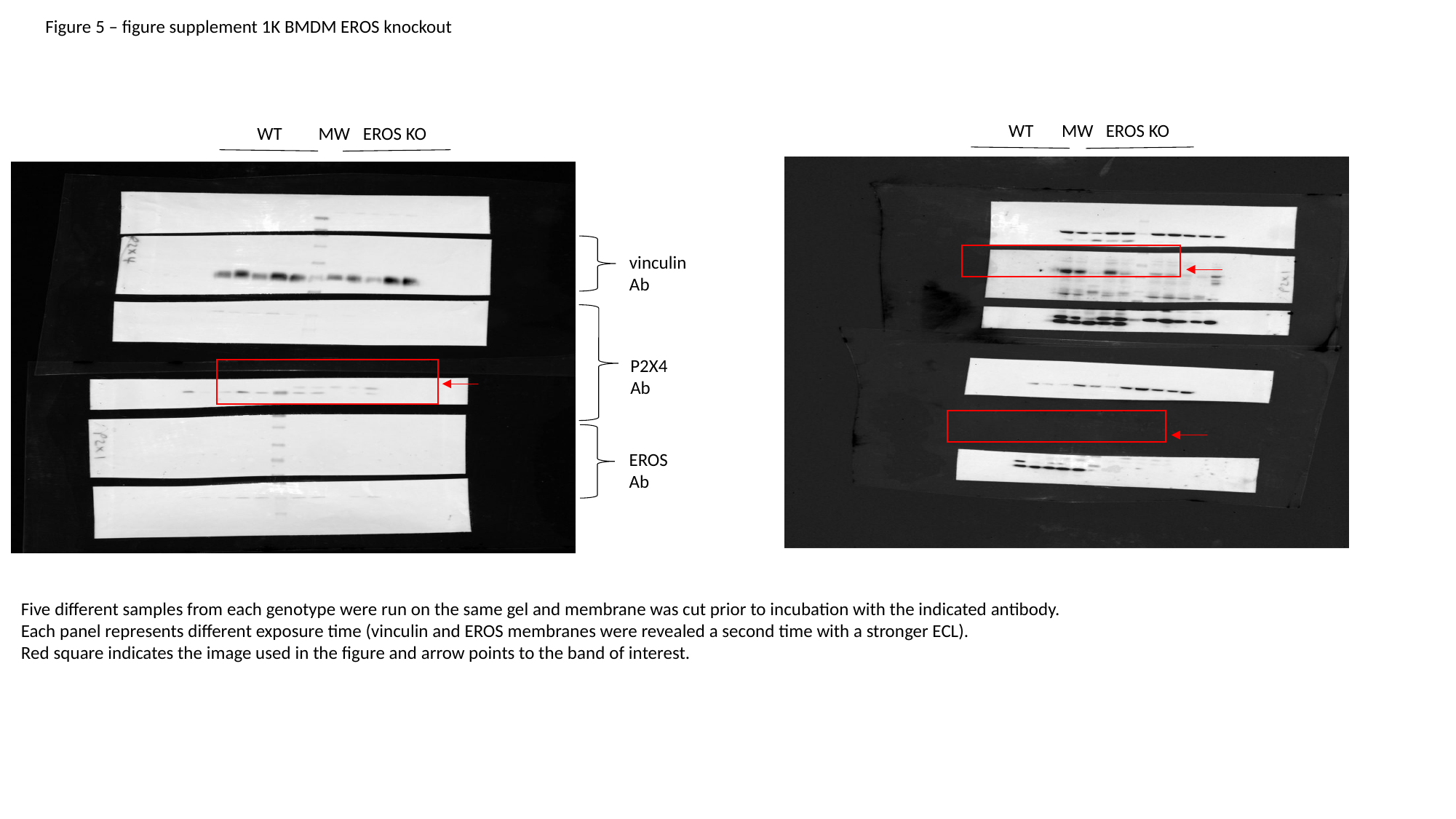

Figure 5 – figure supplement 1K BMDM EROS knockout
WT
MW
EROS KO
WT
MW
EROS KO
vinculin
Ab
 P2X4
 Ab
EROS Ab
Five different samples from each genotype were run on the same gel and membrane was cut prior to incubation with the indicated antibody.
Each panel represents different exposure time (vinculin and EROS membranes were revealed a second time with a stronger ECL).
Red square indicates the image used in the figure and arrow points to the band of interest.
