# Supplementary material for: EROS is a selective chaperone regulating the phagocyte NADPH oxidase and purinergic signalling
Source: eLife. 2022 Nov 24;11:e76387. doi: 10.7554/eLife.76387 (PMC9767466; doi:10.7554/eLife.76387)

**Supplementary File 1**

**A**

| **CRISPR guide** | GAGTCAGCAGCTTCTTGTAGAGG |
| --- | --- |
| ***CYBC1_* GF1** | CTTCATAGGAATCTTGTCGATTGGC |
| ***CYBC1*_ GR1** | GTCCACAAACTCATCTCCTCTCC |

**B**

*CYBC1* clone A07: CRISPR mutation resulting in 46bp frameshift


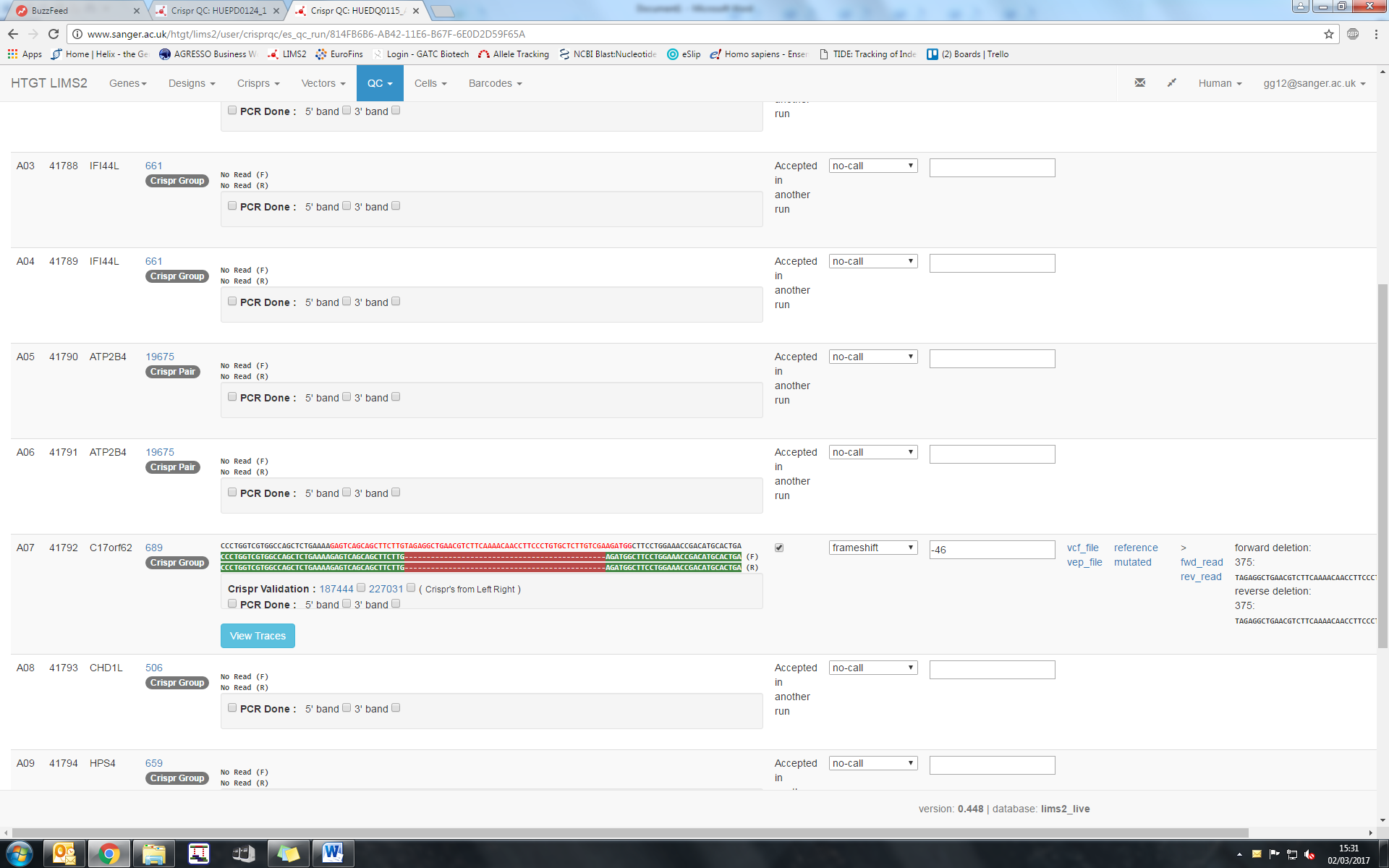

Supplement: Supplementary file 1. — (A) Sequences of the CRISPR guide RNA and the gene-specific genotyping primers (GF1-GR1). (B) Validation of EROS knockout (gene CYBC1) by Sanger sequencing. [file elife-76387-supp1.docx]
